# Heterobimetallic Janus Catalysts for the One‐Pot Synthesis of Polar‐Apolar Block Copolymers

**DOI:** 10.1002/anie.2602995

**Published:** 2026-08-17

**Authors:** Raju Chambenahalli, Gary S. Nichol, Jennifer A. Garden

**Affiliations:** ^1^ School of Chemistry The University of Edinburgh Edinburgh UK

**Keywords:** block copolymers, heterobimetallic catalysts, polar‐apolar polymers, polyester, polyolefin

## Abstract

The one‐pot synthesis of apolar‐polar block copolymers combining polyolefins with polyesters has been a long‐standing challenge in polymer chemistry. Despite broad‐reaching applications of apolar‐polar block copolymers in drug delivery, compatibilisation of polymer blends and biomedical materials, their synthesis often requires multi‐step protocols with multiple catalysts and several isolation and purification steps. This is a particular challenge when combining the polymerisation of non‐activated α‐olefins with lactones, as these two distinct polymerisation mechanisms are often incompatible. This work reports heterobimetallic Janus catalysts for orthogonal 1‐hexene and ε‐caprolactone polymerisation. Generated in situ, the active catalysts combine distinct Ti and Al metal centres connected by a Ti‐C(H)═C(R)CH_2_O‐Al linker (R = Me or Bn). This approach successfully delivered poly(1‐hexene)‐*block*‐poly(ε‐caprolactone) copolymers with well‐controlled linear block copolymer architectures, targeted molar compositions and tuneable molar mass (*M*_n_ values < 67.3 kDa, *Ð* = 1.47), in a one‐pot reaction, an approach that was successfully extended to poly(1‐octene)‐*block*‐poly(ε‐caprolactone) and poly(1‐decene)‐*block*‐poly(ε‐caprolactone) copolymers. Overall, this work is a notable addition to the toolbox for synthesising apolar‐polar block copolymers. This work demonstrates for the first time that titanium‐alkenyl complexes can efficiently initiate olefin polymerisations and that heterobimetallic complexes can act as efficient bifunctional initiators for distinct polymerisation mechanisms.

## Graphical abstract

Heterobimetallic Janus catalysts act as bifunctional initiators, combining two distinct polymerisation mechanisms in one pot to deliver well‐defined apolar‐polar block copolymers. Combining Earth abundant Ti and Al catalysts for respective α‐olefin and lactone polymerisation, the orthogonal activity is enabled by unprecedented initiation from a Ti‐C_alkenyl_ unit.

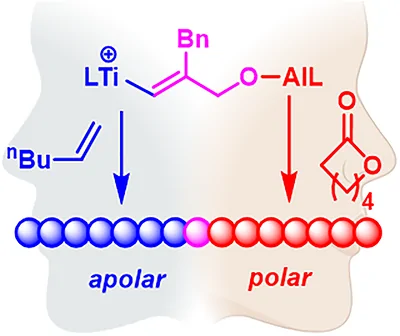

## 1 Introduction

One‐pot catalytic reactions are a powerful synthetic strategy for generating desirable small molecules and macromolecules. From fine chemicals, pharmaceuticals and agrochemicals through to copolymer synthesis, integrating multiple transformations within a single vessel maximises efficiency, improves atom economy, minimises purification steps and reduces chemical waste [1, 2]. This is particularly attractive from a Green Chemistry standpoint [3]. In polymer synthesis, one‐pot transformations include sequential monomer synthesis and polymerisation, tandem polymerisation and post‐functionalisation, and the synthesis of block copolymers (BCPs) [1]. However, one‐pot strategies to generate BCPs are not always straightforward, especially when using different polymerisation mechanisms with distinct, and sometimes incompatible, catalyst requirements.

Harnessing the beneficial properties of two or more homopolymers, BCPs have broad‐ranging applications spanning drug delivery, membranes and the compatibilisation of polymer blends [4]. The block copolymerisation of olefins with polar monomers remains a long‐standing and highly attractive target in polymer chemistry, with the potential to endow hydrophobic polyolefins with remarkably high adhesive, dyeing and moisture absorption properties. However, synthetic strategies to generate poly(olefin‐*block*‐ester)s are limited. Switching between olefin polymerisation and lactone ring‐opening polymerisation (ROP) mechanisms brings complex catalyst requirements and catalyst/monomer compatibility issues. Catalysts that can generate poly(olefin‐*block*‐ester)s are thus scarce [5, 6, 7]. Recently, several catalysts based on M^3+^ rare earth metals (Sc, Y) and lanthanides (Sm, Er, Yb, Lu) have been reported for the copolymerisation of olefins with ε‐caprolactone (CL), yet these studies have been almost exclusively limited to activated alkenes (*i.e*. conjugated 1,3‐dienes or styrene) [8, 9, 10, 11, 12, 13, 14, 15, 16].

The copolymerisation of non‐activated alkenes, such as 1‐hexene, is a greater challenge [17, 18, 19]. A single publication reports metal‐based catalysts for poly(1‐hexene)‐*block*‐poly(ε‐caprolactone) (PH‐*b*‐PCL), based on yttrium and samarium metallocene catalysts (Figure 1A) [17]. However, the 1‐hexene conversion was limited to 15 mol%. Changing the molar composition and block length of BCPs is an effective method of modifying the physical, chemical and material properties, enabling BCP structures to be tailored towards different applications. Alternative synthetic approaches to the related non‐activated alkene copolymer, poly(propene)‐*block*‐poly(ε‐caprolactone) (PP‐*b*‐PCL), have generated hydroxyl‐terminated macroinitiators through sequential polymerisation, oxidation and protonation steps with zirconium and rhodium catalysts [20], followed by activation with Sn(oct)_2_ for CL ROP (oct = octoate, Figure 1B) [21]. This multi‐step strategy not only requires three separate metal catalysts but also uses gaseous CO/H_2_ at high pressures, introducing additional complexity into the experimental set‐up.

**FIGURE 1:**
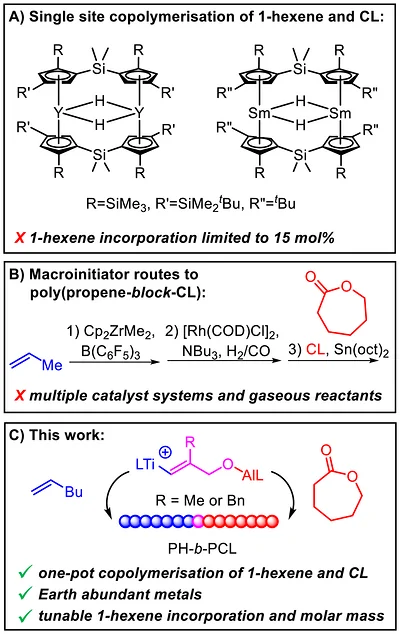
(A) Rare‐earth metal catalysts for the sequential copolymerisation of 1‐hexene and ε‐caprolactone [17]; (B) Synthesis of poly(propene)‐*block*‐poly(ε‐caprolactone) *via* multi‐step synthesis [21]; (C) This work: heterobimetallic Janus catalysts based on Earth abundant metals for the one‐pot synthesis of poly(1‐hexene)‐*block*‐poly(ε‐caprolactone).

Group IV metal catalysts are known to give excellent performance in olefin polymerisation as well as lactone polymerisation. However, there are no reports of Group IV metal catalysts for olefin/lactone block copolymerisations. This may be for several reasons. First, the realm of olefin polymerisation is based on cationic metal catalysts, whereas lactone polymerisation is dominated by neutral metal catalysts. While several studies have shown that cationic metal catalysts can polymerise lactones, this is generally proposed to occur *via* a cationic mechanism, that is, with polymerisation initiated by nucleophilic attack from a lactone monomer and not a cationic metal─O_alkoxide_ bond. This is attributed to the high Lewis acidity of cationic metal complexes compared to the neutral analogues, diminishing the nucleophilicity of the tightly bound metal‐O_alkoxide_ unit [22, 23]. As cationic lactone ROP leads to the formation of homopolyesters, this prevents the formation of poly(olefin‐*block*‐ester)s. Secondly, sequential lactone initiation from a metal‐C_polyolefin_ chain is made challenging by the potential for β‐hydride elimination reactions, which remove the polyolefin chain from the active metal centre to generate homopolyolefins. Thirdly, there are several reports of oxygenated monomers poisoning cationic metal catalysts [24, 25, 26, 27, 28]. These deactivation or chain termination pathways all increase the challenge in synthesising BCPs *via* sequential olefin and lactone addition.

Advances in catalyst design and understanding are essential to achieving the one‐pot synthesis of poly(olefin‐*block*‐ester)s. Here, we report the development of heterobimetallic Janus catalysts featuring two complementary Ti and Al catalytic centres that are specifically designed for olefin and lactone polymerisation, respectively (Figure 1C). Unlike the previously reported Y and Sm‐based catalysts (Figure 1A), our catalysts are based on Al and Ti as the most abundant, and seventh most abundant metals (by weight) in the Earth's crust, respectively [29]. Generated in situ, these heterobimetallic Janus catalysts deliver PH‐*b*‐PCL copolymers with excellent control over the block structure, block length and molar composition. This ultimately enables the synthesis of well‐defined PH‐*b*‐PCL with over 50 mol% of 1‐hexene incorporation through a straightforward, one‐pot synthetic strategy.

## 2 Results and Discussion

As precursors to the targeted heterobimetallic Janus catalysts, a series of neutral and cationic amine bis(phenolate) catalysts were synthesised following modified literature procedures (Figure 2A, see ESI Complex Synthesis for further details) [30, 31, 32]. Complexes LTiMe_2_ **1a**, LTiBn_2_ **1b**, [LTiMe]^+^[MeB(C_6_F_5_)_3_]ˉ **2a** and [LTiBn]^+^[B(C_6_F_5_)_4_]ˉ **2b** were selected because cationic amine bis(phenolate) titanium‐alkyl catalysts have been reported for the living polymerisation of 1‐hexene with efficient initiation at room temperature under neat conditions [30, 33], and where the polymerisation control was attributed to the *cis*‐orientation between the coordinated olefin and the propagating polymer chain. The Ti‐CH_3_ and Ti‐CH_2_Ph initiators were selected to prevent β‐hydride elimination reactions occurring prior to initiation. The co‐catalysts, B(C_6_F_5_)_3_ and [CPh_3_]^+^[B(C_6_F_5_)_4_]‾, were used to abstract an alkyl group to form cationic complexes, which are charge balanced by a spectator anion. The two different co‐catalysts were used due to solubility differences between cationic [LTiMe]^+^ and [LTiBn]^+^, to aid crystallisation, purification and characterisation of the active catalyst species. X‐ray diffraction studies of LTiMe_2_ **1a** confirmed a distorted octahedral geometry at titanium (Figure 1A, Tables S4, S5), with a *cis*‐orientation between the two methyl groups [C1–Ti–C2, 95.26(4)°] and a slightly distorted O─Ti─O axis [O1─Ti1─O2, 163.35(2)°]. Two distinct methyl resonances were observed in the ^1^H and ^13^C{H} NMR spectra (^1^H NMR, δ 1.54 and 1.29 ppm, see Figures S1 and S2), which shows that the solid‐state structure is maintained in C_6_D_6_ solution at room temperature. The benzyl analogue, LTiBn_2_ **1b**, has been characterised previously and adopts a similar structure with two *cis*‐benzyl units and a distorted O─Ti─O axis [C─Ti─C, 99.29(16)°; O─Ti─O, 162.14(11)°] [30, 34].

**FIGURE 2:**
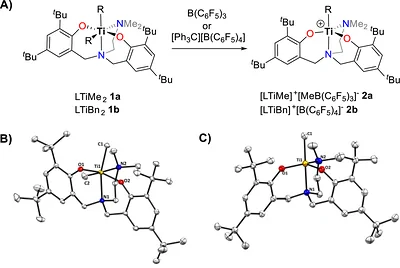
(A) Synthesis of [LTiMe]^+^[MeB(C_6_F_5_)_3_]ˉ **2a** (in pentane solvent) and [LTiBn]^+^[B(C_6_F_5_)_4_]ˉ **2b** (in toluene solvent); (B) Molecular structure of LTiMe_2_ **1a**; (C) Molecular structure of [LTiMe]^+^[MeB(C_6_F_5_)_3_]ˉ **2a**. Thermal ellipsoids are shown at 50% and hydrogen atoms are omitted for clarity. For [LTiMe]^+^[MeB(C_6_F_5_)_3_]ˉ **2a**, solvent and the anion in **2a** have also been omitted for clarity.

The cationic analogues, [LTiMe]^+^[MeB(C_6_F_5_)_3_]ˉ **2a** and [LTiBn]^+^[B(C_6_F_5_)_4_]ˉ **2b**, were derived through the addition of B(C_6_F_5_)_3_ to LTiMe_2_ **1a** and [Ph_3_C]^+^[B(C_6_F_5_)_3_]ˉ to LTiBn_2_ **1b**, respectively (Figure 2A). Both compounds were crystallised from pentane. The molecular structures of [LTiMe]^+^[MeB(C_6_F_5_)_3_]ˉ **2a** and [LTiBn]^+^[B(C_6_F_5_)_4_]ˉ **2b** represent the first crystallographic characterisations of any cationic amine bis(phenolate) Group IV metal complex. This is particularly notable as these complexes have been extensively studied for olefin polymerisation, both academically and industrially, over the past 25 years. The cationic component of [LTiMe]^+^[MeB(C_6_F_5_)_3_]ˉ **2a** has a distorted trigonal bipyramidal geometry at titanium. The unit cell consists of two molecules with slight differences in the bonding parameters, which have Ti─C bond lengths of 2.032(4) and 2.081(4) Å. While these Ti─C bond lengths are by no means the shortest Ti─C bonds reported to date (Ti─C = 1.988(10) Å in cationic [Cp*_2_TiMe(THF)]^+^[BPh_4_]ˉ) [35], they are significantly shorter than those of the neutral precursor LTiMe_2_ **1a** [Ti1─C1, 2.1119(8) Å; Ti1─C2, 2.1087(9) Å]. This contraction highlights the enhanced Lewis acidity of Ti in cationic [LTiMe]^+^[MeB(C_6_F_5_)_3_]ˉ **2a** compared to neutral LTiMe_2_ **1a**.

In the benzyl analogue, [LTiBn]^+^[B(C_6_F_5_)_4_]ˉ **2b** (Figure S86), the [LTiBn]^+^ cation adopts a similar structure to the methyl analogue of **2a**, with the phenyl ring situated above the O1‐Ti1‐O2 unit. This orientation hints at secondary interactions between the aryl ring and Ti. Accordingly, the Ti─C_ipso_ distance of 2.986 Å lies below the sum of the van der Waals radii (∼ 3.5 Å), and the Ti─C bond is extended compared to the methyl analogue [**2b**, 2.136(3) Å; **2a**, 2.082(4) and 2.032(4) Å]. The trigonal bipyramidal geometry of **2a** and **2b** potentially opens up olefin coordination sites at the least sterically congested O2─Ti1─N2 face, as the O2─Ti1─N2 bond angle is significantly wider than the O1─Ti1─O2 and O1─Ti1─N2 bond angles (128.81(13)° *vs* 115.78(12)° and 111.16(13)°, respectively) (Table S4). However, it is worth noting that most of the structurally characterised hexacoordinate Ti amine bis(phenolate) complex reported to date adopts a linear _phenolate_O─Ti─O_phenolate_ axis, which suggests that monomer coordination is most likely to occur between the two phenolic oxygen atoms [32, 36, 37, 38, 39].

Uniquely, the molecular structures of [LTiMe]^+^[MeB(C_6_F_5_)_3_]ˉ **2a** and [LTiBn]^+^[B(C_6_F_5_)_4_]ˉ **2b** feature unsaturated pentacoordinate Ti centres, with no interactions between Ti and the counteranion. Crystallographic characterisation of cationic titanium olefin polymerisation catalysts based on other ligands all show an interaction between the cationic metal centre and the counteranion or a Lewis base [40, 41, 42]. Here, Ti instead adopts a trigonal bipyramidal geometry in both **2a** and **2b**, which presumably stabilises the Ti centre. DOSY NMR analysis of [LTiMe]^+^[MeB(C_6_F_5_)_3_]ˉ **2a** and [LTiBn]^+^[B(C_6_F_5_)_4_]ˉ **2b** suggests that the monomeric solid‐state structure is maintained in C_6_D_6_ solution (see ESI Complex Synthesis for details). This differs from a previously reported cationic Ti‐Me complex based on a related amine bis(phenolate) ligand with a pyridine sidearm, where NMR spectroscopic analysis indicated that the complex dimerised in solution, to generate hexacoordinate Ti centres [43].

Understanding the coordination geometry of these cationic amine bis(phenolate) Ti complexes, instead of the well‐characterised neutral precursors, is important because the active catalyst species is cationic. Upon the loss of one methyl or benzyl anion, to generate cationic **1b** and **2b**, the Ti geometry changes from octahedral to distorted trigonal bipyramidal. Ultimately, it is an equatorial Me/Bn group that is displaced, leaving a vacant equatorial site available for olefin coordination. The two phenoxide rings are brought closer together in the trigonal bipyramidal geometry, and the O1─Ti1─O2 bond angle decreases from 163.35(2)° in **1a** to 115.78(12)° in **2a**, and from 162.14(11)° in **1b** [30, 36] to 119.56(8)° in **2b**. This coordination change is underpinned by the flexibility of the amine bis(phenolate) ligand, which enables Ti to transition between trigonal bipyramidal and octahedral geometries in the absence or presence of olefins. In the absence of olefins, the vacant coordination sites of cationic metal‐alkyl catalysts are typically occupied by the counter anion, Lewis basic solvent, catalyst dimerisation and/or interactions with β‐hydrides of the metal‐alkyl group, the latter of which can give elimination side‐reactions. To prevent β‐hydride elimination, some groups have used fluorinated ligands that interact with and stabilise the β‐hydrogens during propagation [44, 45, 46]. Here, this unprecedented structural characterisation of cationic Ti amine bis(phenolate) shows that the amine bis(phenolate) ligand instead stabilises vacant coordination sites through formation of a trigonal bipyramidal structure, which disfavours β‐hydride activation/elimination pathways. This provides insight into the origins of the living olefin polymerisations reported previously for cationic amine bis(phenolate) complexes [30, 31, 37].

Having gained key structural insights into the cationic titanium component of the targeted heterobimetallic Janus catalysts, the homopolymerisation of 1‐hexene was established. 1‐Hexene was targeted because catalytic routes to poly(1‐hexene‐*block*‐ester) copolymers are limited, yet other hydrophobic/hydrophilic BCPs can self‐assemble into tailored nanostructures, delivering enhanced tensile modulus, flexibility and dyeability compared to the respective homopolymers or their blends [47, 48, 49, 50]. Furthermore, 1‐hexene is relatively straightforward to handle compared to gaseous olefins such as ethene and propene, and so was selected for initial proof‐of‐concept. Our preliminary polymerisation studies verified that [LTiMe]^+^[MeB(C_6_F_5_)_3_]ˉ **2a** and [LTiBn]^+^[B(C_6_F_5_)_4_]ˉ **2b** successfully polymerised 1‐hexene, delivering PH with narrow dispersities (Table 1, entries 1–3), which corresponds with literature reports on related amine bis(phenolate)Ti catalyst systems [30, 36].

**TABLE 1:** Synthesis and characterisation of homo‐ and block copolymerisations of 1‐hexene and ε‐caprolactone to generate poly(1‐hexene) (PH), poly(ε‐caprolactone) (PCL), poly(1‐hexene)‐*block*‐poly(ε‐caprolactone) (PH‐*b*‐PCL), poly(1‐octene)‐*block*‐poly(ε‐caprolactone) (PO‐*b*‐PCL) and poly(1‐decene)‐*block*‐poly(ε‐caprolactone) (PD‐*b*‐PCL) using [LTiMe]^+^[MeB(C_6_F_5_)_3_]ˉ **2a** (R = Me), [LTiBn]^+^[B(C_6_F_5_)_4_]ˉ **2b** (R = Bn), and [LAlOCH_2_C≡CH] **3**.

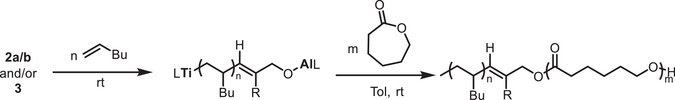												
Entry	Cat.	Polymer	CL (eq.) (% conv.)^a^	Time (h)		*M*_n, obs_ (kDa)^b^	*Ð* ^b^	Mol% Olefin ^c^	Mol% CL ^c^	*T*_g_ Polyolefin	*T*_g_ PCL	*T_m_* PCL
				Olefin	CL					(°C)^d^	(°C)^d^	(°C)^d^
1	**2a** ^e^	PH	—	1.0	—	25.0	1.26	100	—	—	—	—
2	**2b** ^e^	PH	—	0.5	—	16.3	1.21	100	—	—	—	—
3	**2b** ^e^	PH	—	1.0	—	22.5	1.36	100	—	—	—	—
4	**2a/3**	PH	—	0.5	—	11.9	1.15	100	—	—	—	—
5	**2a/3**	PH	—	1.0	—	21.5	1.39	100	—	—	—	—
6	**2b/3**	PH	—	0.5	—	13.2	1.23	100	—	—	—	—
7	**2b/3**	PH	—	1.0	—	20.0	1.40	100	—	−36.0	—	—
8^f^	**3**	PCL	100(96)	—	3.5	18.2	1.08	—	100	—	—	—
9^g^	**3**	PCL	200(>99)	—	15.0	44.5	1.15	—	100	—	−66.9	56.6
10	**2a/3**	PH‐*b*‐PCL	200(48)	0.5	15.0	37.9	1.65	30	70	—	—	—
11	**2a/3**	PH‐*b*‐PCL	200(52)	1.0	15.0	52.0	1.50	52	48	—	—	—
12	**2b/3**	PH‐*b*‐PCL	200(97)	0.5	15.0	60.0	1.35	32	68	−35.6	−63.1	57.9
13	**2b/3**	PH‐*b*‐PCL	200(97)	1.0	15.0	67.3	1.47	49	51	−33.4	−62.3	56.9
14^h^	**2b/3**	PH‐*b*‐PCL	200(>99)	0	15.0	57.7^j^	1.33^j^	—	100	—	—	—
15^i^	**2b/3**	PO	—	1.0	—	16.0^j^	1.53^j^	100	—	−68.6	—	—
16^i^	**2b/3**	PO‐*b*‐PCL	200(98)	1.0	15.0	63.2^j^	1.41^j^	40.4	59.6	—	−61.0	55.9
17^k^	**2b/3**	PD	—	1.0	—	14.5^j^	1.46^j^	100	—	—	—	—
18^k^	**2b/3**	PD‐*b*‐PCL	200(98)	1.0	15.0	53.9^j^	1.55^j^	27.4	72.6	—	−64.9	56.3

1‐Hexene polymerisations were performed in 1‐hexene (5 mL) with a 4000:1:1 monomer:catalyst:co‐catalyst ratio under an inert argon atmosphere

^a^ CL monomer conversion was determined by ^1^H NMR spectroscopy (see Figure S34 for an example ^1^H NMR spectrum).

^b^ Determined by SEC analysis using polystyrene standards in THF, values are uncorrected to enable comparison. The polymers were purified by precipitation from MeOH prior to analysis (see ESI Polymerisation Protocols for details).

^c^ Determined by ^1^H NMR of purified polymer samples (see Figures S53 and S54 for an example ^1^H NMR spectrum and calculation).

^d^ DSC data was collected from the second heating cycle at 5°C/min scan rates (see Figures S70–S76).

^e^ 1‐Hexene polymerisations were performed using in situ generated **2a** or **2b** (see ESI Polymerisation Protocols for details) under an inert argon atmosphere.

^f^ Reaction was carried out at 1 M concentration of CL in toluene.

^g^ Reaction was carried out at 0.4 M concentration of CL in toluene.

^h^ Control reaction performed with simultaneous addition of 1‐hexene and CL.

^i^ 1‐Octene polymerisations were performed in 1‐octene (5 mL) with a 3132:1:1 monomer:catalyst:co‐catalyst ratio under an inert argon atmosphere.

^j^ Determined by SEC analysis using polystyrene standards in CHCl_3_, values are uncorrected to enable comparison.

^k^ 1‐Decene polymerisations were performed in 1‐decene (5 mL) with a 2641:1:1 monomer:catalyst:co‐catalyst ratio under an inert argon atmosphere.

To combine 1‐hexene polymerisation with CL ROP, we designed an aluminium‐alkoxide complex based on the same amine bis(phenolate) ligand but featuring propynoxide units with both alkyne and alkoxide functionalities, LAlOCH_2_C≡CH **3** (Figure 3). This complex was designed to act as a ‘chemical connector’ between olefin polymerisation and lactone ROP, to combine two distinct polymerisation mechanisms. LAlOCH_2_C≡CH **3** features an olefinic unit for insertion into the Ti─C_Me/Ph_ bond of cationic **2a**/**2b**, as well as a ligated aluminium‐alkoxide unit designed to initiate CL polymerisation. The alkyne functionality was specifically selected due to the precedent for cationic group IV metal‐alkyl complexes to insert alkynes in a 1,2‐fashion [51, 52, 53], to generate products without β‐hydrides and thus prevent problematic elimination reactions (Figure 4). In general, 1,2‐insertion is sterically favoured by bulky ligands such as *tert*‐butyl substituted amine bis(phenolates) [54]. In addition to the steric bulk, the LAl fragment was selected because amine bis(phenolate) aluminium alkoxides can efficiently polymerise CL under mild conditions [55], and it reuses the same ligand scaffold as [LTiMe]^+^[MeB(C_6_F_5_)_3_]ˉ **2a**, thus aligning with Green Chemistry Principles. Prepared by combining the LAlMe precursor with 2‐propyn‐1‐ol in a 1:1 ratio (Figure 3A), LAlOCH_2_C≡CH **3** was crystallised from hexane and characterised by single crystal x‐ray diffraction (Figure 3B), multinuclear NMR spectroscopy and elemental analysis (refer to ESI for details).

**FIGURE 3:**
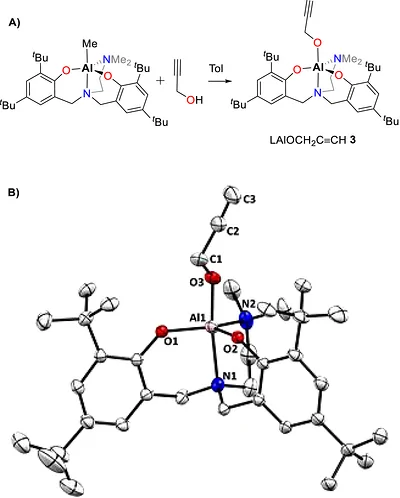
(A) Synthesis of [LAlOCH_2_C≡CH] **3**; (B) Molecular structure of LAlOCH_2_C≡CH **3**. Thermal ellipsoids shown at 30% probability. Hydrogen atoms are omitted for clarity.

**FIGURE 4:**
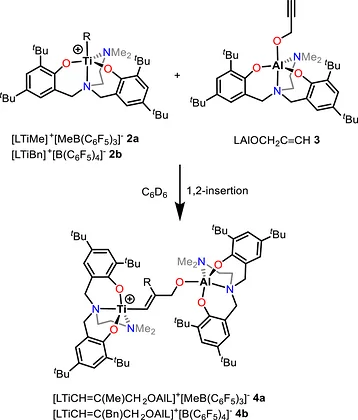
Combination of **2a** or **2b** (only the cationic fragment is shown) with **3** to generate heterobimetallic [LTiCH═C(Me)CH_2_OAlL]^+^[MeB(C_6_F_5_)_3_]ˉ **4a** or [LTiCH═C(Bn)CH_2_OAlL]^+^[B(C_6_F_5_)_4_]ˉ **4b** *via* in situ 1,2‐alkyne insertion into the Ti─Me bond of **2a** (mixture of species) or Ti─Bn bond of **2b** (one major product). Refer to ESI for additional details.

X‐ray diffraction analysis shows that the Al‐C_alkyne_ distances are 4.025(4) Å for Al1‐C2 and 5.135(3) Å for Al‐C3. This was deemed important to avoid steric congestion for two key reasons. First, propyne insertion into the cationic Ti─C bond of **2a** or **2b** requires alkyne coordination to Ti, which may be restricted when using sterically inaccessible alkynes. Secondly, CL ROP requires monomer coordination at Al, thus this metal centre must also be accessible. The short C_3_ alkyne chain was designed to balance preventing internal alkyne coordination to Al, keeping this functionality accessible for coordination‐insertion into the Ti─C bond of **2a** or **2b**, while also disfavouring 2,1‐insertion of the alkyne into the Ti‐C bond (vide supra).

Homopolymerisation studies showed that [LAlOCH_2_C≡CH] **3** was inactive towards 1‐hexene polymerisation and gave excellent control in CL ROP (Table 1, entry 8 and 9), with a narrow dispersity and good agreement between the observed and calculated molar masses, indicative of a single site reaction. While Table 1 contains uncorrected PCL *M*_n, obs_ values from SEC analysis, to enable comparison between homopolymers and copolymers, applying the appropriate PCL correction factor of 0.56 [56] shows a close match between the corrected and calculated *M*_n_ values (see Table S2). For example, in Table 1 entry 9, applying the 0.56 correction factor to *M*_n, obs_ = 44.5 kDa gives *M*_n, corr_ = 24.9 kDa, which is in good agreement with *M*_n, calc_ = 22.6 kDa). The corrected *M*_n_ values can be used to calculate the % active catalyst giving an average value of 99.2% (see Table S2), which indicates that **3** is a highly efficient initiator for CL ROP (see ESI Polymerisation Protocols for details). Interrogating the polymer end groups *via* MALDI‐ToF showed α‐propynoxy, ω‐hydroxy‐end capped PCL (Figure S59). This was corroborated by ^1^H NMR and DOSY analysis, as a distinctive terminal alkyne resonance was observed (─C≡CH, δ: 2.47 ppm in CDCl_3_, Figure S44) with the same diffusion coefficient as the PCL chain (Figure S46). These observations confirm that coordination‐insertion polymerisation occurred [57], initiated by the Al─O─CH_2_C≡CH unit of **3**.

Having verified the individual success of **2a**‐**2b** for hexene polymerisation and **3** for CL ROP, the production of PH‐*b*‐CL was investigated. First, the synthesis of bifunctional Ti‐C_alkenyl_/Al‐O_alkoxide_ catalysts was investigated by combining **2a** or **2b** with **3** in C_6_D_6_ solvent (Figure 4). ^1^H NMR spectroscopic studies showed that the reaction of [LTiMe]^+^[MeB(C_6_F_5_)_3_]ˉ **2a** with [LAlOCH_2_C≡CH] **3** gave a mixture of species, including the expected alkyne insertion product [LTiCH═C(Me)CH_2_OAlL]^+^[MeB(C_6_F_5_)_3_]ˉ **4a** (Figure 4) along with LAlMe and presumably [LTiOCH_2_C≡CH]^+^[MeB(C_6_F_5_)_3_]ˉ as the metathesis products (see Figure S23 and Scheme S1). In contrast, the combination of the benzyl analogue [LTiBn]^+^[B(C_6_F_5_)_4_]ˉ **2b** with LAlOCH_2_C≡CH **3** gave solely the alkyne insertion product [LTiCH═C(Bn)CH_2_OAlL]^+^[B(C_6_F_5_)_4_]ˉ **4b**, selectively generating a heterobimetallic species with dual Ti‐C_alkenyl_/Al‐O_alkoxide_ functionality (Figures S26–S31). The differences in reaction selectivity between the methyl and benzyl insertion are attributed to a combination of steric and electronic influences. Not only is the benzyl unit larger, it can also engage in metal‐pi or agostic interactions (Figure S86), weakening the Ti─C bond (vide supra) to facilitate alkyne insertion and potentially also stabilising the formation of the heterometallic Janus catalyst [58]. Importantly, the heterometallic Janus catalyst produced from **2b** and **3** was essentially stable in C_6_D_6_ solution for a period of 4 days at room temperature (Figure S32), confirming that the formation of the titanium‐alkenyl unit prevented elimination side‐reactions.

Following the in situ combination of **2a** or **2b** with **3** in benzene, 1‐hexene was added (see Table 1 and ESI for details). After 0.5 or 1 h, the reaction mixture was divided in two; half was quenched by the addition of benzyl alcohol, which gave a vivid colour change from red (active catalyst) to yellow, indicating termination of the olefin polymerisation. The resultant PH was characterised by ^1^H, ^13^C{^1^H} NMR, SEC and DOSY analysis, to enable comparisons to be drawn between the PH precursor and copolymer products (see Table 1, Figure 5 and ESI for details). To the other half of the reaction mixture, a toluene solution of CL was added (200 equivalents *vs* Ti). The gradual colour change from red to yellow indicated termination of the olefin polymerisation during CL ROP (see ESI for details). Indeed, the addition of polar monomers has been long reported to poison Group 4 metal catalysts towards polymerisation [59, 60]. Yet the heterometallic Janus catalyst displayed good activity towards CL ROP (Table 1), and the resultant polymers were quenched, purified and isolated as fibrous white materials (see ESI for details).

**FIGURE 5:**
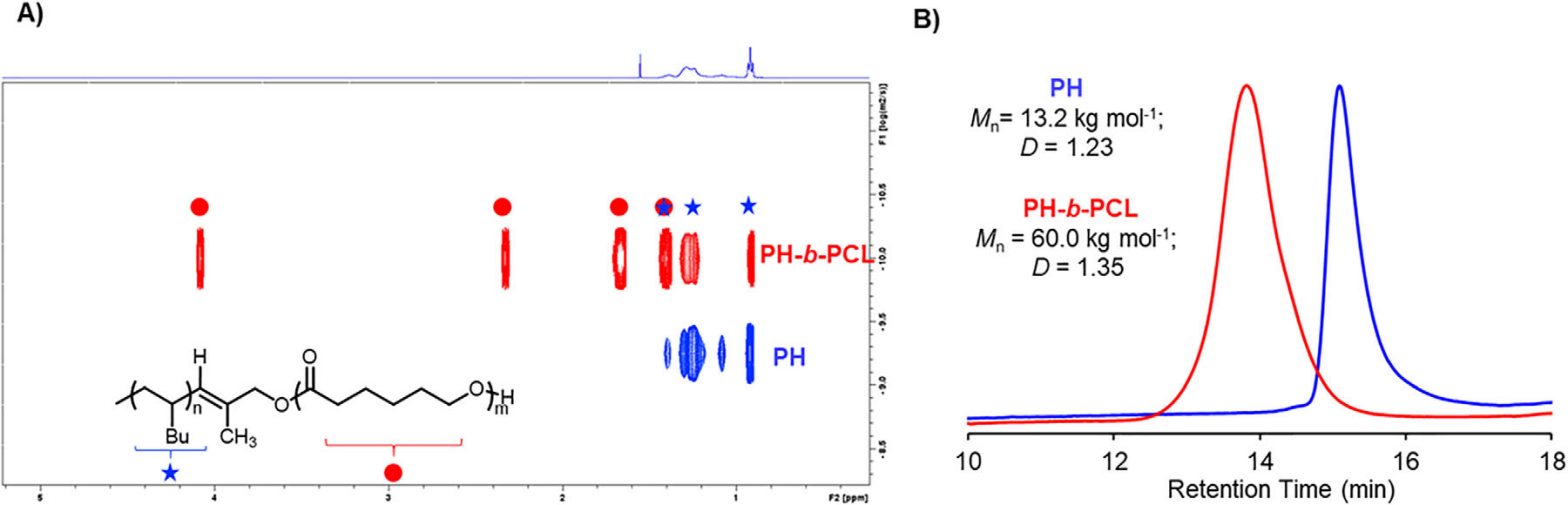
(A) Stacked DOSY of purified samples of PH‐*b*‐PCL (Table 1, entry 12) and PH (Table 1 entry 6), recorded in CDCl_3_; B) SEC traces of PH‐*b*‐PCL (Table 1, entry 12) and PH (Table 1, entry 6).

The formation of PH‐*b*‐PCL from the heterobimetallic Janus catalyst **2b**/**3** was evidenced by multinuclear NMR spectroscopy, SEC and DSC analysis. First, ^1^H NMR analysis revealed an almost equimolar ratio of PH (49%) and PCL (51%) in the copolymer structure (entry 13). DOSY NMR analysis shows that the ^1^H NMR resonances from the PH and PCL blocks have the same diffusion coefficient (Figure 5A). There is also a significant shift in the diffusion co‐efficient for PH‐*b*‐PCL compared to the PH precursor, confirming chain extension upon addition of CL. SEC analysis of purified PH‐*b*‐PCL also showed an increase in *M*_n, obs_, for example from 20.0 to 67.3 kDa from PH to PH‐*b*‐PCL (Table 1, entries 6 and 12). Both of the SEC traces are unimodal with a narrow dispersity (Figure 5B).

DSC analysis at 5°C/min scan rates showed two distinct glass‐transition (*T*_g_) values at ‐33.4°C and ‐62.3°C, corresponding to the PH and PCL blocks respectively, as well as a melting temperature (*T*_m_) at 56.9°C for the PCL block (Table 1, entry 13, Figure S72). These thermal transitions are in agreement with the values obtained for the homopolymers (Table 1, entries 7 and 9, Figures S70 and S71) and align with literature values for PH and PCL homopolymers [61, 62]. The observation of these discrete transitions confirms that the blocks remain phase separated and chemically distinct, further validating successful BCP formation. This is further corroborated by the *T*_m_ values for the semi‐crystalline PCL homopolymer and PH‐*b*‐PCL copolymer, which are almost identical (56.6°C versus 56.9°C, respectively, entries 9 and 13). Thermogravimetric analysis showed that the PH, PCL and PH‐*b*‐PCL polymers all have high thermal stability, with respective *T*_d,5%_ values of 367.2°C, 324.3°C and 295.0°C (Figures S77–S79). The PH‐*b*‐PCL copolymers display good solubility in organic solvents of varying polarity, including chloroform, THF, toluene and benzene, which makes them relatively facile to process, and are also colourless. These features are important for a multitude of different applications, for both homopolymers and copolymers.

The initiation of 1‐hexene polymerisation from the Ti–C_alkenyl_ unit and CL ROP from the Al–O_alkoxide_ group of the heterobimetallic Janus catalyst **2b**/**3** was subsequently investigated. Shorter chain copolymers were synthesised to facilitate observation of the bifunctional alkenyl/alkoxide initiator by NMR spectroscopy. ^1^H NMR analysis confirmed the presence of PH, PCL and the ─C(H)═C(Bn)CH_2_O─ linker group, which was identified by the diagnostic alkene and OCH_2_ resonances at 5.33 ppm and 4.38 ppm, respectively (Figures S49–S52). This provided evidence that **2b**/**3** truly acts as a Janus catalyst with each ‘face’ performing a different polymerisation, where 1‐hexene polymerisation was initiated by the LTi‐C_alkenyl_ group and CL polymerisation was initiated by the Al‐O_alkoxide_ unit. This was further supported by MALDI‐ToF analysis of PCL homopolymers generated from heterobimetallic **2b**/**3**, which gave the expected CH_2_═C(Bn)CH_2_O end group (Figure S60). ^1^H NMR end group analysis of PH homopolymer generated from heterobimetallic **2b**/**3** showed diagnostic resonances from the titanium‐alkenyl Ti–CH═C(CH_2_Ph)CH_2_OAl catalyst (*e.g*. δ 5.32, ─C*H*═C(CH_2_Ph)CH_2_OH; 3.95 ─CH═C(CH_2_Ph)C*H*_2_OH; 3.51 ─CH═C(C*H*_2_Ph)CH_2_OH, see Figure S40). The presence of a 2‐benzylpropenol end group was further supported by COSY, HSQC and HMBC analysis (Figures S41–S43). Comparison of the crude and purified SEC traces shows that a very small portion of PH is formed, which can be easily removed *via* precipitation and washing with hexane (Figure S67). This is an advantage compared to the previously reported rare earth metal catalysts (Figure 1A), as while the SEC traces for PH‐*b*‐PCL were not reported, the synthesis of poly(1‐hexene)‐*block*‐poly(methylmethacrylate) (PH‐*b*‐PMMA) gave a bimodal SEC trace with equivalent‐sized peaks for the PH‐*b*‐PMMA copolymer and PH homopolymer. This indicates poor selectivity for the copolymers, which corresponds with the lower‐than‐expected PH incorporation into the PH‐*b*‐PCL copolymer reported (<15 mol%) [17]. Together with the aforementioned DOSY, SEC and DSC studies, these end group analyses show that the Janus catalyst **2b**/**3** efficiently acts as a bifunctional initiator to copolymerise 1‐hexene and CL, to generate linear PH‐*b*‐PCL block copolymer architectures across different copolymer length scales (Table 1).

The heterobimetallic Janus catalyst system was specifically designed with a titanium alkenyl unit (Figure 4), which enhances the catalyst stability by preventing β‐hydride elimination prior to polymerisation. This work demonstrates, for the first time, that metal‐alkenyl units can efficiently initiate olefin polymerisation through a coordination‐insertion pathway [63]. This builds on previous reports of hafnium‐phenyl initiators [64, 65], alkyne insertion into Ti─C_alkyl_ and Zr─C_aryl_ bonds [51, 66], ethylene oligomerisation from a Ga‐CH═CH_2_ intermediate and cationic zirconium vinylidene initiators featuring a cationic carbon [67, 68, 69]. Our work not only shows that initiation from a Ti‐C_alkenyl_ is possible, but also that this is a useful strategy to prevent both α‐ and β‐hydride elimination reactions occurring prior to polymerisation. This key catalyst feature helps to stabilise the heterobimetallic Janus catalyst, and ultimately, to generate PH‐*b*‐PCL copolymers.

Comparing the performance of **2a**/**3** with **2b**/**3** in copolymer synthesis shows that CL ROP is much slower with the methyl analogue **2a**/**3**, reaching only 48% conversion instead of 97% CL conversion under analogous conditions (Table 1, entries 10 and 12). This is attributed to competing metathesis side‐reactions reducing the concentration of active catalyst with **2a**/**3**, whereas **2b**/**3** selectively forms the desired heterometallic Janus catalyst (Schemes S1 and S2). In spite of the reduced activity of **2a**/**3**, analysis of the resultant polymers *via* DOSY NMR and SEC analysis still indicates the formation of PH‐*b*‐PCL copolymers (Figures S55 and S64).

Control reactions with [LTiMe]^+^[MeB(C_6_F_5_)_3_]ˉ **2a**, in the absence of LAlOCH_2_C≡CH **3**, showed that cationic **2a** is capable of the sequential polymerisation of 1‐hexene at room temperature and CL at elevated temperatures, but delivered two separate homopolymers (refer to ESI Polymerisation Protocols for details). Once 1‐hexene is polymerised, the propagating [LTi‐C_polymer_]^+^[MeB(C_6_F_5_)_3_]‾ chain may homopolymerise CL by initiation from a cationic LTi‐H species formed *via* β‐hydride transfer, or through a cationic mechanism [23]. The simultaneous addition of 1‐hexene and CL to **2b**/**3** generated only PCL, providing further evidence that polar CL poisons the cationic Ti centre towards 1‐hexene polymerisation (Table 1, entry 14). This underscores the need to include **3** in order to generate the heterobimetallic Janus catalyst as a bifunctional initiator, and to deliver BCPs through sequential monomer addition, with a cationic Ti‐C unit for 1‐hexene polymerisation and an Al‐O_alkoxide_ unit for CL polymerisation.

To investigate the substrate scope, two additional copolymers were synthesised using the optimum heterometallic Janus catalyst system, **2b**/**3**, through the sequential addition of 1‐octene or 1‐decene followed by CL. This generated the target BCPs, poly(1‐octene‐*block*‐ε‐caprolactone) (PO‐*b*‐PCL, Table 1, entry 16) and poly(1‐decene‐*block*‐ε‐caprolactone) (PD‐*b*‐PCL, entry 18). The copolymers were characterised *via* ^1^H NMR spectroscopy, which confirmed the polyolefin and PCL. The extension of the polyolefin upon the subsequent addition of CL was evidenced by DOSY NMR (Figures S57 and S58), along with SEC analysis (Figures S68 and S69). DSC analysis of PO‐*b*‐PCL and PD‐*b*‐PCL (Figures S75 and S76) showed *T*_g_ and *T*_m_ values for PCL that are essentially identical to the PCL homopolymer (entries 16, 18 and 9). To the best of our knowledge, this is the first report of well‐defined PO‐*b*‐PCL and PD‐*b*‐PCL block copolymers.

To probe the potential of the PH‐*b*‐PCL copolymers to act as compatibilisers, polymer blends of PH:PCL were prepared *via* solvent casting from chloroform solvent, both in the absence and presence of PH‐*b*‐PCL (using PH, PCL and PH‐*b*‐PCL from entries 7, 9 and 13, respectively). Without PH‐*b*‐PCL, the 50:50 PH:PCL blend showed clear phase separation between PH and PCL (Figure 6A). In the presence of 2 wt% of PH‐*b*‐PCL, the PCL domain size was visibly reduced, and the PH/PCL interfaces are less well‐defined; both are features of successful compatibilisation (Figure 6B). The potential for PH‐*b*‐PCL to compatibilise polyolefin/polyester interfaces is interesting for several reasons. First, the compatibilisation of polyolefin/polyester blends is challenging due to the structural and polarity differences between these two polymer classes. Secondly, PH is a liquid whilst PCL is a solid at room temperature, and the compatibilised blend is a solid which forms a film upon solvent casting. In contrast, film formation was unsuccessful without the PH‐*b*‐PCL compatibiliser. This shows that PH‐*b*‐PCL can compatibilise polymers with different states of matter and generates solid polymers even when using PH as the matrix phase.

**FIGURE 6:**
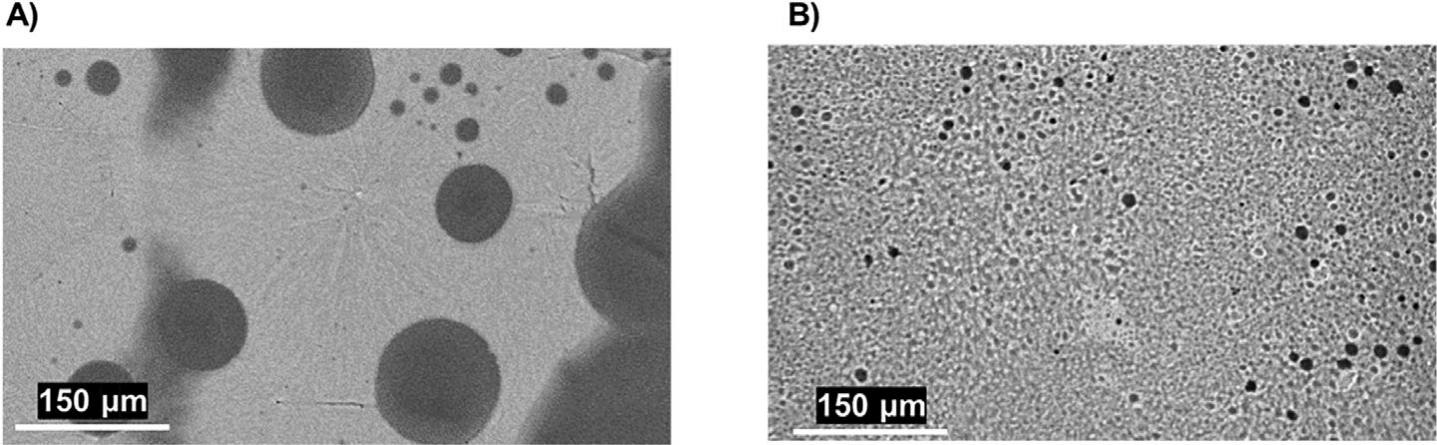
(A) SEM image of a 50:50 PH:PCL polymer blend (dark grey = PH fraction, light grey = PCL fraction, showing PH oil dispersed in a solid PCL matrix); (B) SEM of a 50:50 PH:PCL blend with 2 wt% of PH‐*b*‐PCL added as a compatibiliser (see Figures S82 and S83 for additional details).

## 3 Conclusion

This work describes the synthesis of apolar‐polar poly(1‐hexene‐*block*‐ε‐caprolactone), poly(1‐octene‐*block*‐ε‐caprolactone) and poly(1‐decene‐*block*‐ε‐caprolactone) copolymers using heterobimetallic Janus catalysts based on Earth abundant Ti and Al. To the best of our knowledge, these are the first bifunctional initiators based on heterobimetallic complexes to be reported for olefin/lactone copolymerisation. This work demonstrates that heterometallic complexes can show unique reactivity in the formation of poly(olefin‐*block*‐ester)s, producing well‐defined block copolymers where the individual components generate only homopolymers. The heterobimetallic Janus catalyst was generated in situ *via* the selective 1,2‐insertion of an alkynyl‐functionalised aluminium alkoxide into a Ti─C_Bn_ bond. Applying this approach to generate a Ti–C_alkenyl_ species, without β‐hydrides, stabilised the formation of the heterobimetallic Janus catalyst by preventing elimination reactions. The resultant Ti‐C_alkenyl_ centre successfully initiates 1‐hexene polymerisation. This is the first initiation of olefin polymerisation from a metal‐alkenyl unit, opening the door to using metal‐alkenyl complexes as efficient polymerisation initiators. Subsequent addition of ε‐caprolactone activates the Al–O_alkoxide_ bond towards lactone ROP, generating BCPs with well‐controlled chain lengths and molar compositions. Overall, this work not only provides a one‐pot method to generate poly(1‐hexene‐*block*‐ε‐caprolactone) but also paves the way for other olefin/ester copolymerisations, including other monomer combinations as well as the synthesis of diblock, triblock and multiblock structures. SEM analysis indicates that PH‐*b*‐PCL copolymers can compatibilise polymer blends combining PH as a liquid, apolar polymer with PCL as a solid, polar polymer, which will be the subject of follow‐on studies in our laboratory.

## Author Contributions

**Raju Chambenahalli**: investigation, writing – original draft, methodology, formal analysis, funding acquisition. **Gary S. Nichol**: formal analysis. **Jennifer A. Garden**: writing – review and editing, conceptualization, funding acquisition, methodology, supervision.

## Conflicts of Interest

The authors declare no conflicts of interest.

## Supporting information



Deposition Numbers 2559078–2559081 contain the supplementary crystallographic data for this paper. These data can be obtained free of charge via the joint Cambridge Crystallographic Data Centre (CCDC) and Fachinformationszentrum Karlsruhe Access Structures service.
**Supporting File**: anie74082‐sup‐0001‐SuppMat.pdf.

## Data Availability

The data that support the findings of this study are available from the corresponding author upon reasonable request.
