# Supplementary material for: Heterobimetallic Janus Catalysts for the One‐Pot Synthesis of Polar‐Apolar Block Copolymers
Source: Angew Chem Int Ed Engl. 2026 Aug 17;65(41):e2602995. doi: 10.1002/anie.2602995 (PMC13638117; doi:10.1002/anie.2602995)
Supplement: Supplementary file 1 [file ANIE-65-e2602995-s001.pdf]

Heterobimetallic Janus Catalysts for the One-Pot Synthesis of Polar-Apolar Block Copolymers

Raju Chambenahalli, Gary S. Nichol, Jennifer A. Garden*

Electronic Supporting Information

Table of Contents

General experimental details 3

NMR spectroscopy 3

SEC analysis 4

DSC analysis 4

TGA analysis 4

SEM analysis 4

Complex synthesis and characterisation protocols 5

Synthesis of $LTiMe_2$ **1a** 5

Synthesis of $[LTiMe][MeB(C_6F_5)_3]$ **2a** 5

Synthesis of $[LTiBn][B(C_6F_5)_4]$ **2b** 6

Synthesis of $LAlOCH_2C{\equiv}CH$ **3** 7

NMR scale reaction for the *in situ* generation of $[LTiCH{=}C(Me)CH_2OAlL][MeB(C_6F_5)_3]$ **4a** 7

NMR scale reaction for the *in situ* generation of $[LTiCH{=}C(Bn)CH_2OAlL][B(C_6F_5)_4]$ **4b** 8

Polymerisation protocols 9

Homopolymerisation of 1-hexene with compound **2a** 9

Homopolymerisation of 1-hexene with compound **2b** 9

Attempted homopolymerisation of 1-hexene with compound **3** 9

Homopolymerisation of 1-hexene with compound **4a** and **4b** 9

Homopolymerisation of ε-caprolactone with compound **3** 10

Kinetic study of the homopolymerisation of ε-caprolactone with compound **3** 10

Homopolymerisation of 1-hexene and ε-caprolactone with $[ABPTiMe][MeB(C_6F_5)_3]$ **2a** via sequential monomer addition 12

Attempted copolymerisation of 1-hexene and ε-caprolactone with [LTi-CH=C(Bn)CH_2OAlL][B(C_6F_5)$_4$] **4b** via simultaneous monomer addition ... 12
Copolymerisation of ε-caprolactone and 1-hexene, 1-octene or 1-decene with [LTi-CH=C(Me)CH_2OAlL][MeB(C_6F_5)$_3$] **4a** via sequential monomer addition ... 12
Synthesis of short chain PH-*b*-PCL copolymers with *in situ* generated [LTi-CH=C(Bn)CH_2OAlL][B(C_6F_5)$_4$] **4b** ... 13
Complex characterisation: NMR spectroscopy ... 14
Polymer characterisation ... 36
Crystallography data ... 69
References ... 75

General experimental details

All manipulations involving air- or moisture-sensitive compounds were performed either in a glovebox or using standard Schlenk techniques under an argon atmosphere. Amine bis(phenolate) ligand LH_2,[1] and the associated titanium and aluminium precursor complexes $LTiCl_2$,[2] $LTiBn_2$ **1b**[3] and LAlMe[4] were synthesised using previously reported procedures. All reagents and solvents were obtained from Sigma-Aldrich, Fischer Scientific, Honeywell, Strem or Acros Organics and used as received unless stated otherwise. Dry toluene solvent was collected from a solvent purification system (Innovative Technologies). Anhydrous deuterated solvents ($CDCl_3$ and C_6D_6) used for NMR spectroscopic analysis of air- and moisture-sensitive compounds were dried overnight over CaH_2, degassed three times by freeze-pump-thaw and vacuum transferred under reduced pressure. All dry solvents were stored in the presence of activated 4 Å molecular sieves under an argon atmosphere. 1-Hexene, 1-octene, and 1-decene were degassed three times by freeze-pump-thaw cycles and stored in the presence of activated 4 Å molecular sieves under an argon atmosphere for a period of minimum three days before use. ε-Caprolactone (CL) was dried over CaH_2 and distilled under reduced pressure prior to use.

NMR spectroscopy

1D NMR (^{1}H, ^{13}C{^{1}H}, ^{11}B, ^{19}F) and 2D NMR (DOSY, COSY, HSQC, HMBC, NOESY) spectra were recorded on a Bruker AVA500 or AVA600 spectrometer at 300 K. Chemical shifts (δ) are given in parts per million (ppm), and coupling constants (*J*) are reported in Hertz (Hz). ^{1}H and ^{13}C NMR spectra were referenced with respect to the residual peaks of deuterated solvents (C_6D_6: 7.16 ppm, 128.06 ppm; $CDCl_3$: 7.27 ppm, 77.00 ppm).

Diffusion-Ordered Spectroscopy (DOSY) NMR experiments were performed at 300 K on a Bruker NEO 2 channel (H/X) instrument operating at a frequency of 500 MHz for proton resonance under TopSpin (version 4.5, Bruker Biospin, Karlsruhe) and equipped with a z-gradient BBO/5mm tuneable "CryoProbe"™ probe providing a maximum gradient output of 50.7 G/cm (5.07G/cmA). Diffusion ordered NMR data was acquired using the Bruker pulse program dstebpgp3s with a spectral width of 10330 Hz (centred on 6.175 ppm) and 32768 data points.[5] A relaxation delay of 2 s was employed along with a diffusion time (Δ) of 100 ms and a longitudinal eddy current delay (LED) of 5 ms. Bipolar gradients pulses (δ/2) of 1.5 ms and homospoil gradient pulses of 0.6 ms were used. The gradient strengths of the 3 homospoil pulses were -13.17%, -17.13%, -15.37%. 16 experiments were collected with the bipolar gradient strength, initially at 5% (1st experiment), linearly increased to 95% (16th experiment). All gradient pulses were smooth-square shaped (SMSQ10.100) and after each application a recovery delay of 200 µs used. The experiment was run with 16 scans per increment, employing one stimulated echo with two spoil gradients. DOSY NMR spectra were formatted in

TopSpin and Dynamic centre. Parameters were optimised empirically to find the best quality of data for presentation purposes. The reported DOSY masses (to the nearest whole number) and aggregation states were determined by comparison to a calibration plot made with a range of standards (adamantane, 2-phenylpyridine, pyrene, tri(*o*-tolyl)-phosphine, tetraphenylphthaline and 2,2′-bis(diphenylphosphino)-1,1′-binaphthyl (BINAP)) with molar masses varying from 136.24 to 622.67 g mol^{-1} in C_6D_6.

SEC analysis

Polymer samples (2 – 10 mg) were dissolved in GPC grade THF (1 mL) and filtered using a 0.2 µm PTFE syringe filter. SEC analyses of the filtered polymer samples were carried out in GPC grade THF at a flow rate of 1 mL min^{-1} at 35 °C on a 1260 Infinity II GPC/SEC single detector system with mixed bed C PL gel columns (300 x 7.5 mm). The RI detector was calibrated using narrow molecular weight polystyrene standards.

DSC analysis

DSC experiments were performed using a DSC (DSC 8000, Perkin Elmer) with an intracooler. In all experiments, the DSC cell was purged with nitrogen at 50 mL/min. Indium was used to calibrate the cell constant. Materials were sealed in Tzero aluminium pans with a closed lid. Runs were performed at a ramp rate of 5°C/min from -80 °C to 100 °C.

TGA analysis

TGA experiments were performed using a simultaneous thermal analyser (NETZSCH STA 449 F1 Jupiter with platinum furnace). Runs were performed at a ramp rate of 10°C/min from 25 °C to 600 °C under nitrogen atmosphere.

SEM analysis

A 100 mg sample of the polymer was dissolved in 0.5 mL of chloroform and stirred until completely dissolved. The resulting solution was drop-cast onto a glass slab and allowed to evaporate slowly in air for two days at room temperature. The dried polymer thin films were carefully peeled off the glass slabs. The samples were then analysed for surface morphology using the Phenom G6 Pure SEM.

Complex synthesis and characterisation protocols

Air- and moisture-sensitive Ti and Al complexes were prepared and manipulated using standard Schlenk and glovebox techniques, under an argon atmosphere.

Synthesis of $LTiMe_2$ 1a

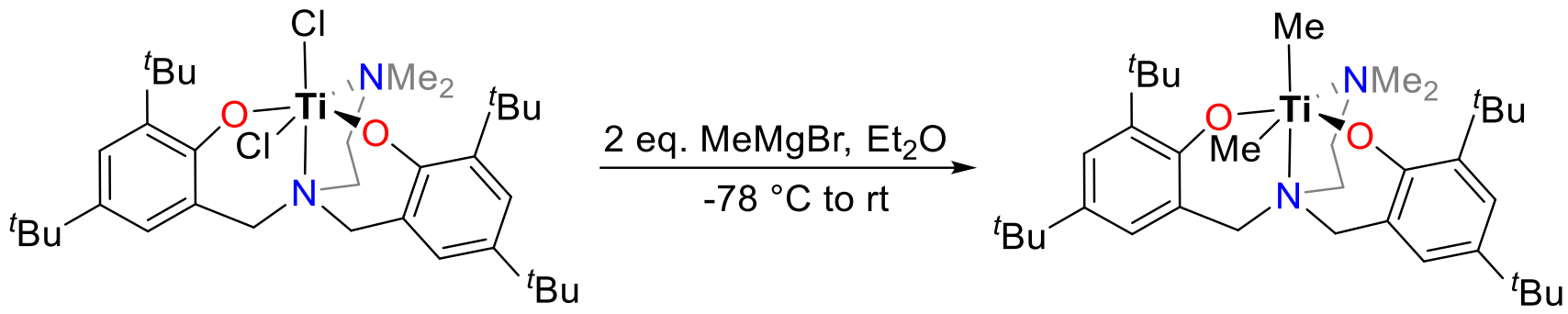

$LTiMe_2$ **1a**

Compound $LTiCl_2$ was prepared according to literature procedures,[2] then 0.93 mmol (600 mg) was added to a 50 mL Schlenk flask and dissolved in 10 mL diethyl ether. Subsequently, 1.9 mmol of a MeMgBr solution in Et_2O (0.63 mL, 3 M solution) was added over a period of approximately 5 min with constant stirring at -78 °C. The reaction mixture was then stirred for 1 h, before it was allowed to gradually warm to room temperature. The volatiles were subsequently removed under vacuum at room temperature. The residue was re-dissolved in hexane followed by filtration. The filtrate was dried under vacuum at room temperature to obtain an orange residue as a crude product of $LTiMe_2$ **1a**. Orange-red crystals of $LTiMe_2$ **1a** suitable for single-crystal X-ray diffraction (SC-XRD) were obtained from a concentrated solution in hexanes held at -30 °C. Isolated yield: 87% (486 mg).

^{1}H NMR (500 MHz, C_6D_6, ppm): δ 7.69 (d, 2H, $^4J_{HH}$ = 2.5 Hz, *p*-C*H*-Ar), 6.98 (d, 2H, $^4J_{HH}$ = 2.5 Hz, *o*-C*H*-Ar), 3.48 (d, $^2J_{HH}$ = 13.5 Hz, 2H, NC*H*$_2$Ar), 2.58 (d, $^2J_{HH}$ = 13.5 Hz, 2H, NC*H*$_2$Ar), 2.01 (m, 2H, NC*H*$_2$), 1.97 (s, 18H, C(C*H*$_3$)$_3$), 1.54 (s, 3H, TiC*H*$_3$), 1.50 (s, 6H, N(C*H*$_3$)$_2$), 1.44 (s, 18H, C(C*H*$_3$)$_3$), 1.29 (s, 3H, TiC*H*$_3$).

^{13}C{^{1}H} NMR (126 MHz, C_6D_6, ppm): δ 160.6 (C_{ipso}-Ar), 140.8, 136.1, 126.5, 124.5, 124.5 (all *C*-Ar), 65.1 (N*C*H$_2$Ar), 61.6 and 61.4 (Ti*C*H$_3$), 59.7 and 52.2 (N*C*H$_2$), 47.5 (N*C*H$_3$), 35.9 and 34.5 (*C*(CH$_3$)$_3$), 32.1 and 30.9 (C(*C*H$_3$)$_3$).

EA calculated for $C_{36}H_{60}N_2O_2Ti$: C, 71.98; H, 10.07; N, 4.66. Found: C, 71.47; H, 9.94; N, 4.63.

Synthesis of [LTiMe][MeB(C_6F_5)$_3$] 2a

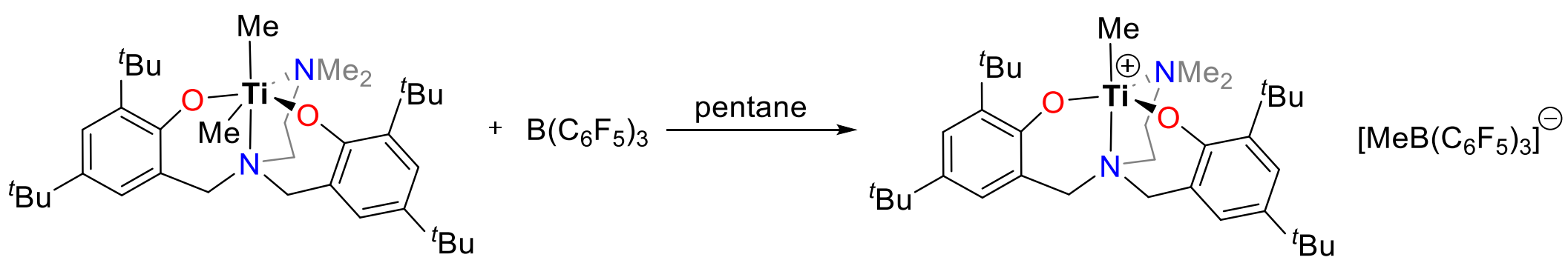

1a [LTiMe][MeB(C_6F_5)$_3$] **2a**

In a glovebox under argon atmosphere, compound $LTiMe_2$ **1a** (24.0 mg, 0.04 mmol) was dissolved in 2 mL of pentane and then added to a pentane (2 mL) solution of $B(C_6F_5)_3$ (20.5 mg, 0.04 mmol) in a Supelco reaction vial. A thick red oil settled in the bottom of the vial after the solution was allowed to stand for about thirty minutes at room temperature. The product, $[LTiMe][MeB(C_6F_5)_3]$ **2a**, was crystallised from the red oil in pentane as red-orange needles over a period of three days at -30 °C, which were suitable for SC-XRD. Yield: 89% (40 mg).

^{1}H NMR (500 MHz, C_6D_6, ppm): δ 7.42 (d, 2H, $^4J_{HH}$ = 2.5 Hz, *p*-C*H*-Ar), 6.99 (d, 2H, $^4J_{HH}$ = 2.5 Hz, *o*-C*H*-Ar), 3.16 (d, $^2J_{HH}$ = 13.5 Hz, 2H, NC*H*$_2$Ar), 2.95 (d, $^2J_{HH}$ = 13.5 Hz, 2H, NC*H*$_2$Ar), 2.38 (m, 2H, NC*H*$_2$), 2.15 (m, 2H, NC*H*$_2$), 1.73 (s, 6H, N(C*H*$_3$)$_2$), 1.70 (s, 3H, TiC*H*$_3$), 1.40 (s, 18H, C(C*H*$_3$)$_3$), 1.29 (s, 18H, C(C*H*$_3$)$_3$).

^{13}C{^{1}H} NMR (126 MHz, C_6D_6, ppm): δ 160.7 (C_{ipso}-Ar), 150.2, 148.3, 148.0, 139.2, 138.2, 137.3 136.1 135.1, 125.8, 125.0, 124.2 (all *C*-Ar), 74.5 (Ti*C*H$_3$), 62.6 (N*C*H$_2$), 58.4 (N*C*H$_2$Ar), 49.6(N*C*H$_2$), 48.6 (N(*C*H$_3$)$_2$), 35.1 and 34.8 (*C*(CH$_3$)$_3$), 31.4 and 29.8 (C(*C*H$_3$)$_3$).

^{11}B NMR (128 MHz, C_6D_6, ppm): -14.19.

^{19}F NMR (471 MHz, C_6D_6, ppm): −131.7 (d, *o*-C*F*), −163.9 (t, *p*-C*F*), −166.4 (t, *m*-C*F*).

EA calculated for $[LTiMe][MeB(C_6F_5)_3]$·pentane, $C_{59}H_{72}BF_{15}N_2O_2Ti$: C, 59.81; H, 6.13; N, 2.36. Found: C, 59.79; H, 6.22; N, 2.23.

Synthesis of $[LTiBn][B(C_6F_5)_4]$ 2b

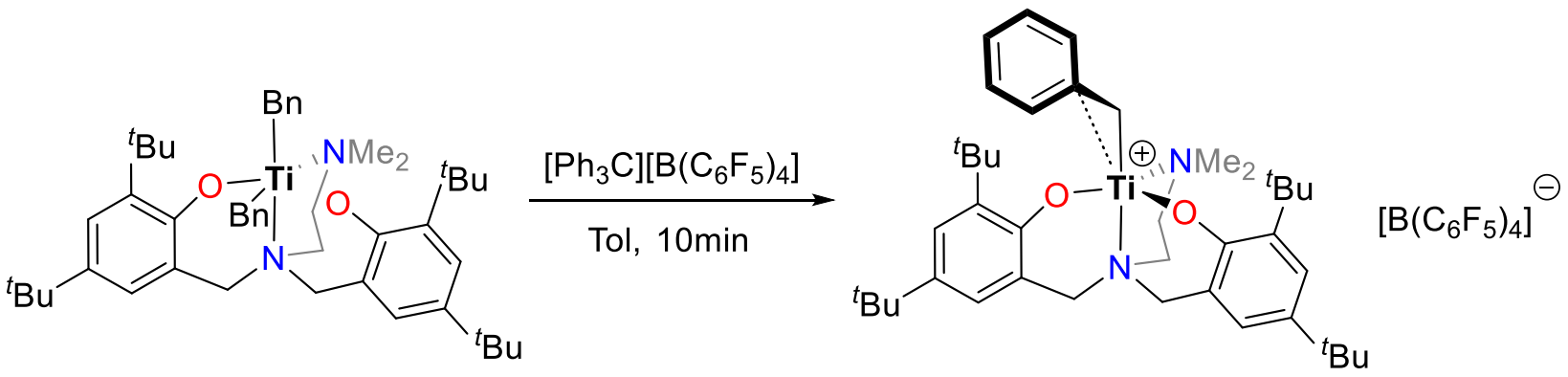

1b [LTiBn][B(C6F5)4] **2b**

A solution of $LTiBn_2$ **1b** (75.3.0 mg, 0.10 mmol) in toluene (5 mL) was added to a solution of $[Ph_3C][B(C_6F_5)_4]$ (92.2 mg, 0.10 mmol) in toluene (5 mL) in a Schlenk flask under an argon atmosphere, with constant stirring. The reaction mixture was stirred for a further 10 minutes at room temperature, after which the volatiles were removed under reduced pressure. The resulting residue was washed with pentane (15 mL x 2) and dried *in vacuo* to yield the desired product, $[LTiBn][B(C_6F_5)_4]$ **2b**, as a brown powder. Crystals of **2b** suitable for SC-XRD were obtained from the pentane washings after standing for 30 minutes at room temperature. Yield: 73% (98 mg).

^{1}H NMR (500 MHz, C_6D_6, ppm): δ 7.42 (d, 2H, $^4J_{HH}$ = 2.2 Hz, *p*-C*H*-Ar), 7.22 (br, 4H, TiCH$_2$*Ph*), 6.99 (m, 1H, TiCH$_2$*Ph*), 6.95 (d, 2H, $^4J_{HH}$ = 2.0 Hz, *o*-C*H*-Ar), 3.42 (s, 2H, TiC*H*$_2$Ph), 3.24 (d, $^2J_{HH}$ = 14.2 Hz, 2H,

NCH_2Ar), 2.99 (d, $^2J_{HH}$ = 14.2 Hz, 2H, NCH_2Ar), 2.28 (m, 2H, NCH_2), 2.24 (m, 2H, NCH_2), 1.79 (s, 6H, $N(CH_3)_2$), 1.35 (s, 18H, $C(CH_3)_3$), 1.27 (s, 18H, $C(CH_3)_3$).

$^{13}C\{^1H\}$ NMR (126 MHz, C_6D_6, ppm): δ 161.0 (C_{ipso}-Ar), 150.09, 148.2, 148.1, 145.0, 139.9, 138.0, 136.1, 135.4, 129.5, 128.5, 126.6, 125.5 125.2, 124.5 (all *C*-Ar), 101.9 ($TiCH_2Ph$), 62.6 (NCH_2), 59.3 (NCH_2Ar), 51.4 (NCH_2), 48.3 ($N(CH_3)_2$), 35.1 and 34.8 ($C(CH_3)_3$), 31.3 and 30.1 ($C(CH_3)_3$).

^{11}B NMR (128 MHz, C_6D_6, ppm): -15.92.

^{19}F NMR (471 MHz, C_6D_6, ppm): −131.7 (br, *o*-C*F*), −162.1 (br, *p*-C*F*), −165. (br, *m*-C*F*).

EA calculated for $C_{65}H_{61}BF_{20}N_2O_2Ti$: C, 58.22; H, 4.59; N, 2.09. Found: C, 57.91; H, 4.70; N, 1.99.

Synthesis of LAlOCH₂C≡CH 3

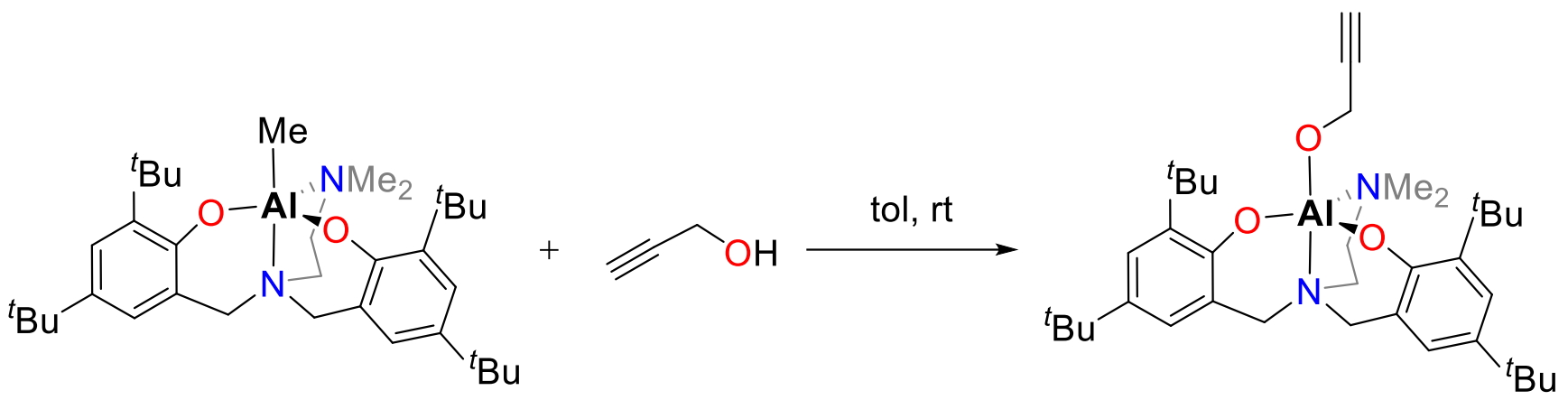

LAlOCH$_2$C≡CH **3**

Compound [LAlMe] (1.00 g, 1.77 mmol) was added to a 50 mL Schlenk flask along with 10 mL of toluene. Following this, 2-propyn-1-ol (103 µL, 1.77 mmol) was added over a period of approximately 5 min, with constant stirring at room temperature. The reaction mixture was then stirred for an additional hour. All the volatiles were subsequently removed under vacuum at room temperature to obtain LAlOCH$_2$C≡CH **3** as colourless solid. A portion of the residue (100 mg) was re-dissolved in 5 mL pentane and the solution was kept for crystallisation at -30 °C to obtain colourless crystals suitable for SC-XRD. Yield: 91% (980 mg).

^1H NMR (500 MHz, C_6D_6, ppm): δ 7.58 (d, 2H, $^4J_{HH}$ = 2.5 Hz, *p*-C*H*-Ar), 6.77 (s, br, 2H, *o*-C*H*-Ar), 5.35 (d, 2H, $^3J_{HH}$ = 2.5 Hz, OCH_2), 3.13 (br, 4H,CH_2Ar), 2.22 (t, 1H, $^4J_{HH}$ = 2.5 Hz, CC*H*), 2.15 (s, 6H, $N(CH_3)_2$), 1.85 (s, br, 2H, NCH_2CH_2N), 1.74 (s, 18H, $C(CH_3)_3$), 1.61 (s, br, 2H, NCH_2CH_2N), 1.42 (s, 18H, $C(CH_3)_3$).

$^{13}C\{^1H\}$ NMR (126 MHz, C_6D_6, ppm): δ 156.1 (C_{ipso}-Ar), 139.0, 138.7, 124.3, 123.8, 121.5 (all *C*-Ar), 89.2 (*C*CH), 69.3 (C*C*H), 59.1 (CH_2-Ar), 54.9 (NCH_2CH_2N), 52.7 (OCH_2) 49.3 (NCH_2CH_2N), 48.7 (NCH_3), 35.5 ($C(CH_3)_3$), 34.3 ($C(CH_3)_3$), 32.2($C(CH_3)_3$), 30.0 ($C(CH_3)_3$).

EA calculated for $C_{37}H_{57}N_2O_3Al$: C, 73.47; H, 9.50; N, 4.60. Found: C, 74.76; H, 9.40; N, 4.56.

NMR scale reaction for the *in situ* generation of [LTiCH=C(Me)CH₂OAlL][MeB(C₆F₅)₃] 4a

In a Supelco reaction vial in a glovebox under an argon atmosphere, [LTiMe][MeB(C_6F_5)$_3$] **2a** (11.1 mg, 0.01 mmol) was dissolved in C_6D_6 (0.6 mL). 1 equivalent of LAlOCH$_2$C≡CH **3** (6.05 mg 0.01 mmol) was

subsequently added into the same vial. The solution was then transferred into a J Young's NMR tube and allowed to react for one hour. This solution was subjected to multinuclear and 2D NMR spectroscopy, which showed the formation of **4a**, **4a'** and [LAlMe] as depicted in Scheme S1. See Figures S23-S25 for assignment of key reaction products.

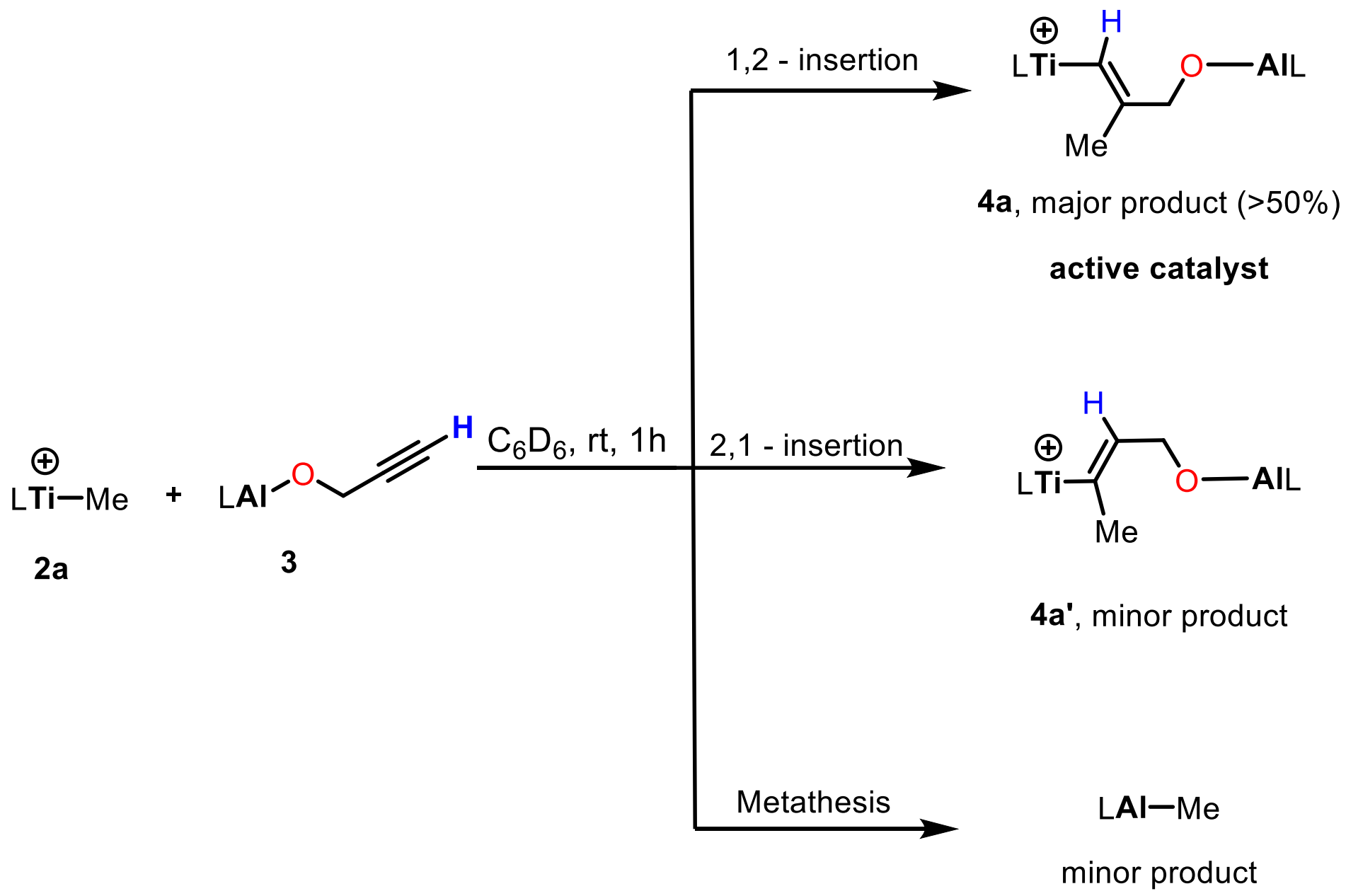

*Scheme S1: Schematic representation of reaction products obtained from the reaction of **2a** and **3** (see Figure S23).*

NMR scale reaction for the *in situ* generation of [LTiCH=C(Bn)CH_2OAlL][B(C_6F_5)$_4$] 4b

In a glovebox, [LTiBn][B(C_6F_5)$_4$] **2b** (13.4 mg, 0.01 mmol) was dissolved in C_6D_6 (0.6 mL) in a Supelco reaction vial. Subsequently, 1 equivalent of LAlOCH_2C≡CH **3** (6.05 mg, 0.01 mmol) was added into the same vial. The solution was then transferred into a J Young's NMR tube and allowed to react for one hour. This solution was subjected to multinuclear and 2D NMR spectroscopy to confirm the formation of **4b** (see Figures S25-S30).

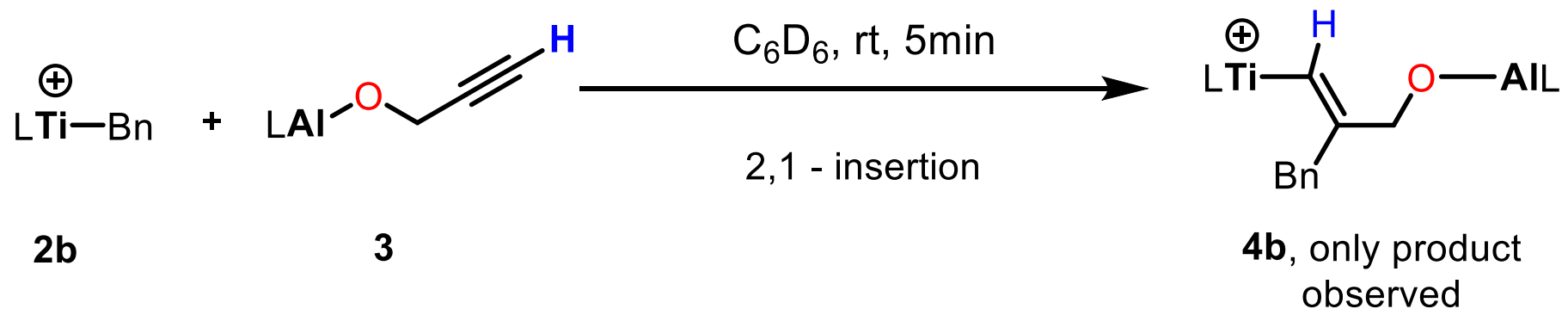

*Scheme S2: Schematic representation for the synthesis of **4b** from the reaction of **2b** and **3**.*

Polymerisation protocols

Homopolymerisation of 1-hexene with compound 2a

In a glovebox, $LTiMe_2$ **1a** (6 mg, 0.01 mmol) in 0.5 mL of benzene was added into a Supelco reaction vial equipped with a magnetic stirrer. 1.1 equivalents of $B(C_6F_5)_3$ (5.53 mg, 0.011 mmol) was added into the same vial. The colour of the solution turned red, indicating that the active species, $[LTiMe][MeB(C_6F_5)_3]$ **2a**, was generated. 1-Hexene (5 mL) was added to the above mixture, which caused a subtle change in the red colour of the solution, and the vial was capped with a PTFE-lined screw cap. The solution was stirred at room temperature for the specified length of time. The polymerisation was then quenched and purified with excess acidified cold methanol (3 x 20 mL). The obtained yellow oily polymer was dried under vacuum until constant weight was achieved. Note: In some cases, extensive PH purification was performed through dissolution in hexane followed by precipitation from methanol. This helped to remove the yellow colour, which was attributed to residual titanium species, generating a colourless oily polymer.

Homopolymerisation of 1-hexene with compound 2b

In a glovebox, $[LTiBn][B(C_6F_5)_4]$ **2b** (13.4 mg, 0.01 mmol) in 0.5 mL of benzene was added into a Supelco reaction vial equipped with a magnetic stirrer. 1-Hexene (5 mL) was added to the above mixture and the vial was capped with a PTFE-lined screw cap. The solution was stirred at room temperature for the specified length of time. The polymerisation was then quenched and purified with excess acidified cold methanol (3 x 20 mL), and the obtained yellow oily polymer was dried under vacuum until constant weight was achieved.

Attempted homopolymerisation of 1-hexene with compound 3

In a glovebox, $LAlOCH_2C{\equiv}CH$ **3** (6.05 mg, 0.01 mmol) in 0.5 mL of benzene was added into a Supelco reaction vial equipped with a magnetic stirrer. 1-Hexene (5 mL) was added to the above mixture and the vial was capped with a PTFE-lined screw cap. The solution was stirred at room temperature for 24 h. An ^1H NMR spectrum of the crude sample was recorded in $CDCl_3$. No polymerisation of 1-hexene was observed.

Homopolymerisation of 1-hexene with compound 4a and 4b

Example 1-hexene homopolymerisation procedure given for **4a**: In a glovebox under argon atmosphere, $[LTiMe][MeB(C_6F_5)_3]$ **2a** (11.13 mg, 0.01 mmol) was dissolved in benzene (1.0 mL) in a Supelco reaction vial equipped with a magnetic stirrer. 1 equivalent of $LAlOCH_2C{\equiv}CH$ **3** (6.05 mg 0.01 mmol), with respect to $[LTiMe][MeB(C_6F_5)_3]$ **2a**, was added into the same reaction vial. The reaction mixture was stirred at room temperature for 30 minutes to generate heterometallic **4a** *in situ*, along with other species present as identified by NMR spectroscopy (see Scheme S1). 1-Hexene

(5 mL) was added to the above mixture and the vial was capped with a PTFE-lined screw cap. The solution was stirred at room temperature for the specified length of time. The polymerisation was quenched and purified with excess acidified cold methanol (3 x 20 mL), and the resultant yellow oily polymer was dried under vacuum until constant weight was achieved.

A similar procedure was followed for the homopolymerisation of 1-hexene with compound **4b**, by instead combining [LTiBn][B(C_6F_5)$_4$] **2b** with LAlOCH$_2$C≡CH **3**.

Homopolymerisation of ε-caprolactone with compound 3

In a glovebox, LAlOCH$_2$C≡CH **3** (6.05 mg, 0.01 mmol) was added into a Supelco reaction vial equipped with a magnetic stirrer. Toluene (5 mL) was added to the reaction vial, followed by the addition of 200 equivalents of CL (228 mg, 2.0 mmol) with respect to compound **3**. The vial was capped with a PTFE-lined screw cap. The solution was stirred at room temperature for 15 h. The polymerisation was quenched and washed with excess of acidified cold methanol (3 x 50 mL) and the obtained polymer, which was a white solid, was dried under vacuum until constant weight was achieved.

Kinetic study of the homopolymerisation of ε-caprolactone with compound 3

In a glovebox, LAlOCH$_2$C≡CH **3** (6.05 mg, 0.01 mmol) was added into a Supelco reaction vial equipped with a magnetic stirrer. Toluene (1 mL) was added to the reaction vial, followed by 100 equivalents of CL (114.0 mg, 1.0 mmol) with respect to compound **3**. The vial was capped with a PTFE-lined screw cap and the solution was stirred at room temperature. At regular intervals, a sample (20.0 µL) was removed from the reaction mixture, quenched with $CDCl_3$ (0.5 mL) and subjected to ^{1}H NMR and SEC analysis to determine the monomer conversion along with the polymer M_n and dispersity $Đ$ (Table S1).

*Table S1: Homopolymerisation of CL with isolated LAlOCH$_2$C≡CH **3** at room temperature.*

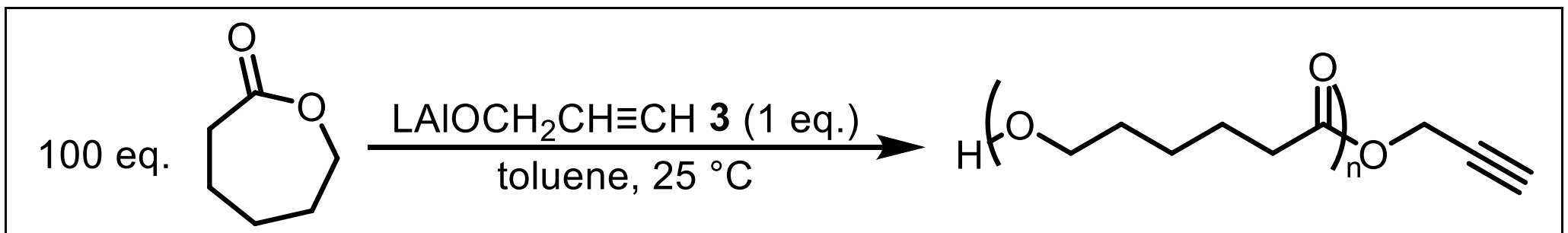

Entry	**Time (h)**	**Conversion (%)**	**Temp (°C)**	**$M_{n,calc}$ (kDa)**	**$M_{n,obs}$ (kDa)**	**$Đ$**
1	0.5	16	25	1.83	2.28	1.14
2	1.0	40	25	4.56	4.65	1.13
3	1.5	61	25	6.96	6.97	1.10
4	2.0	79	25	9.01	8.69	1.09
5	3.0	94	25	10.79	10.57	1.07
6	3.5	96	25	10.95	10.78	1.07

*Table S2: Comparison of calculated, observed, and corrected SEC values for PCL homopolymer generated using [LAlOCH$_2$C≡CH] **3**. This data corresponds to Table 1, entries 8 and 9.*

Table 1 Entry	Cat.	Polymer	CL (eq.) (% conv)[a]	Time (h) CL	$M_{n,calc}$[b] (kDa)	$M_{n,obs}$[c] (kDa)	$M_{n,corr}$[d] (kDa)	*Đ*[c]	% Active Catalyst[e]
**entry 8**	**3**	PCL	100(96)	3.5	11.0	18.2	10.2	1.08	**107.5**
**entry 9**	**3**	PCL	200(>99)	15.0	22.6	44.5	24.9	1.15	**90.9**
						Average % Active Catalyst =			**99.2**

[a] CL conversion was determined by ^{1}H NMR spectroscopy (see Figure S34 for an example ^{1}H NMR spectrum). [b] $M_{n,calc}$ calculated from monomer conversion; $M_{n,calc}$ = ([CL]$_0$/[Cat]$_0$) × (% conversion of CL) × 114.14, assuming 1 polymer chain per catalyst centre. [c] Determined by SEC analysis using polystyrene standards in THF. [d] Calculated by applying the 0.56 correction factor for PCL. [e] Note that there is a significant error in these calculations, which are based on the $M_{n,corr}$ values from SEC data.

Calculation of the % active catalyst for **3** in CL ROP was performed using the corrected SEC values from Table 1, entries 8 and 9 (see Table S2 for full details).

Equation 1: $\boldsymbol{M_{n,obs}} = \frac{\textbf{Conversion} \times \textbf{MW of εCL} \times \textbf{Loading}}{\textbf{N}}$, thus Loading = $\frac{\textbf{N} \times \boldsymbol{M_{n,obs}}}{\textbf{Conversion} \times \textbf{MW of εCL}}$

$M_{n,obs}$ is the M_n of the polymer obtained using SEC analysis (g mol^{-1}); Conversion = $\frac{[\text{εCL}]_t}{[\text{εCL}]_0}$; MW of ε-CL = 114.14 g mol^{-1}; N = no. of initiating groups (1 for complex **3**).

Equation 2: $\mathbf{n_{CL}} = \mathbf{n_{cat}} \times \textbf{Loading}$, thus $\mathbf{n_{cat}} = \frac{\mathbf{n_{εCL}}}{\textbf{Loading}}$;

$n_{εCL}$ = no. of mmol of ε-CL used; n_{cat} = no. of mmol of active catalyst, Loading = value calculated using Equation S1.

Equation 3: $\%\ \textbf{active catalyst} = \frac{\mathbf{n_{cat}}}{\mathbf{n_{cat0}}}$, where n_{cat} = value calculated using Equation 3; n_{cat0} = no. of mmol of catalyst added.

Homopolymerisation of 1-hexene and ε-caprolactone with [ABPTiMe][MeB(C_6F_5)$_3$] 2a via sequential monomer addition

In a glovebox, LTiMe$_2$ **1a** (6 mg, 0.01 mmol) was added into a Supelco reaction vial equipped with a magnetic stirrer. 1.1 equivalents of B(C_6F_5)$_3$ (5.53 mg 0.011 mmol) with respect to **1a** was added into the same vial, followed by 1-hexene (5 mL), and the vial was then capped with a PTFE-lined screw cap. This generated a red solution, indicating that [ABPTiMe][MeB(C_6F_5)$_3$] **2a** was generated. The solution was stirred at room temperature for 1 h. A toluene solution of CL (200 eq. with respect to LTiMe$_2$ **1a**, 228.0 mg, 2.0 mmol in 5 mL toluene) was added. The colour of the reaction mixture turned from red to yellow indicating the arrest of olefin polymerisation. This solution was further allowed to stir at 110 °C for another 15 h, to allow CL ROP. The conversion of CL to PCL was determined from the crude reaction mixture through analysing a reaction aliquot by ^{1}H NMR spectroscopy. Subsequently, the reaction mixture was quenched and precipitated by addition to an excess of acidified cold methanol (100 mL). The crude product obtained was then washed with cold methanol (3 x 100 mL) to remove trace metal impurities. The obtained product yielded a mixture of two homopolymers PH and PCL, as evidenced by DOSY NMR and SEC analysis.

Attempted copolymerisation of 1-hexene and ε-caprolactone with [LTi-CH=C(Bn)CH_2OAlL][B(C_6F_5)$_4$] 4b via simultaneous monomer addition

In a glovebox, [LTiBn][B(C_6F_5)$_4$] **2b** (13.40 mg, 0.01 mmol) was dissolved in benzene (1.0 mL) in a Supelco reaction vial equipped with a magnetic stirrer. 1 equivalent of LAlOCH_2C≡CH **3** (6.05 mg 0.01 mmol) with respect to compound **2b** was added into the same vial. The reaction mixture was stirred at room temperature for 30 minutes to generate compound **4b**. A mixture of 1-hexene (5 mL) and a toluene solution of CL (228 mg, 2.0 mmol in 5 mL) was added. The vial was capped with a PTFE-lined screw cap. This solution was allowed to stir at room temperature for 15 h. The percentage conversion of CL to PCL was analysed from the crude reaction mixture through ^{1}H NMR spectroscopy. Subsequently, the reaction mixture was quenched and precipitated by addition into an excess of acidified cold methanol (100 mL). The obtained crude product was then washed with cold methanol (2 x 100 mL) to remove trace metal impurities. The obtained polymer, which was a white solid, was dried under vacuum until constant weight was achieved, and the ^{1}H NMR spectrum revealed the presence of only PCL homopolymer.

Copolymerisation of ε-caprolactone and 1-hexene, 1-octene or 1-decene with [LTi-CH=C(Me)CH_2OAlL][MeB(C_6F_5)$_3$] 4a via sequential monomer addition

Example copolymerisation procedure given for **4a**: In a glovebox, [LTiMe][MeB(C_6F_5)$_3$] **2a** (22.26 mg, 0.02 mmol) was dissolved in benzene (2.0 mL) in a Supelco reaction vial equipped with a magnetic stirrer. 1 equivalent of LAlOCH_2C≡CH **3** (12.10 mg 0.02 mmol) with respect to compound **2a** was added into the same vial. The reaction mixture was stirred at room temperature for 30 minutes to generate

compound **4a**. 1-Hexene (10 mL) was added to the above mixture and the vial was capped with a PTFE-lined screw cap. The solution was stirred at room temperature for the specified amount of time. Half of the reaction mixture (6 mL) was removed *via* syringe, quenched with MeOH, and analysed by ^{1}H NMR spectroscopy and SEC. To the remaining active polymerisation, a toluene solution of CL (228 mg, 2.0 mmol in 5 mL) was added. The colour of the reaction mixture turned from red to yellow indicating the arrest of olefin polymerisation. This solution was allowed to stir at room temperature for another 15 h. The percentage conversion of CL to PCL was analysed from the crude reaction mixture through ^{1}H NMR spectroscopy. Subsequently, the reaction mixture was quenched and precipitated by addition into an excess of acidified cold methanol (100 mL). The obtained crude product was then washed with cold methanol (2x100 mL) to remove trace metal impurities, followed by *n*-hexane (3x100 mL) to remove polyhexene homopolymer. The block copolymer PH-*b*-PCL was obtained as a colourless fibrous material which was dried under vacuum until constant weight was achieved.

The same procedure was followed for the copolymerisation of ε-caprolactone with 1-hexene, 1-octene or 1-decene (5 mL in each case) with [LTi-CH=C(Bn)CH_2OAlL][B$(C_6F_5)_4$] **4b**, which was generated *in situ* by combining [LTiBn][B$(C_6F_5)_4$] **2b** with LAlOCH_2C≡CH **3**.

Synthesis of short chain PH-*b*-PCL copolymers with *in situ* generated [LTi-CH=C(Bn)CH_2OAlL][B$(C_6F_5)_4$] 4b

Short chain PH-*b*-PCL copolymers were generated for NMR analysis, to investigate the presence of PH, PCL and the CH=C(Bn)CH_2O linker unit within the same molecule. In a glovebox, [LTiBn][B$(C_6F_5)_4$] **2b** (13.40 mg, 0.01 mmol) was dissolved in toluene (1.0 mL) in a Supelco reaction vial equipped with a magnetic stirrer. 1 equivalent of LAlOCH_2C≡CH **3** (6.05 mg, 0.01 mmol) with respect to compound **2b** was added into the same vial. The reaction mixture was stirred at room temperature for 5 minutes to generate **4b** *in situ*. 1-Hexene (0.124 mL, 1.0 mmol) was added to the above solution and the vial was capped with a PTFE-lined screw cap. The solution was stirred at room temperature for 30 minutes followed by the addition of 100 equivalents of CL (114.0 mg, 1.0 mmol). This solution was then stirred at room temperature for an additional hour. Subsequently, the reaction mixture was quenched and precipitated by addition into an excess of acidified cold methanol (20 mL). The obtained crude product was then washed with cold methanol (2x20 mL) to remove trace metal impurities, followed by *n*-hexane (3x20 mL) to remove polyhexene homopolymer. The block copolymer PH-*b*-PCL obtained as a pale-yellow solid which was dried under vacuum until constant weight was achieved and was subjected to NMR spectroscopy for the end group analysis.

Complex characterisation: NMR spectroscopy

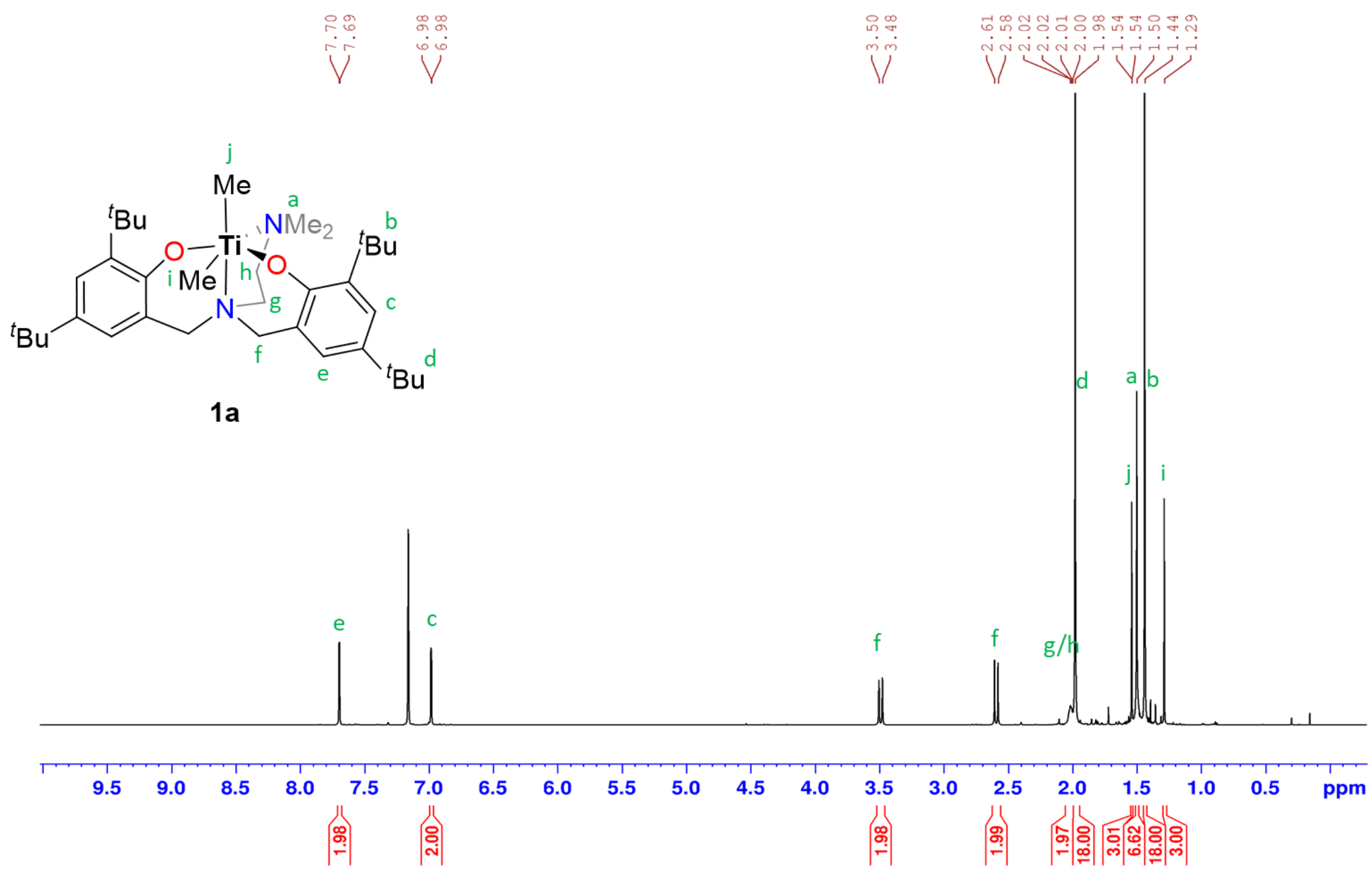

*Figure S1: ^{1}H NMR spectrum of LTiMe$_2$ **1a** in C$_6$D$_6$ (300 K).*

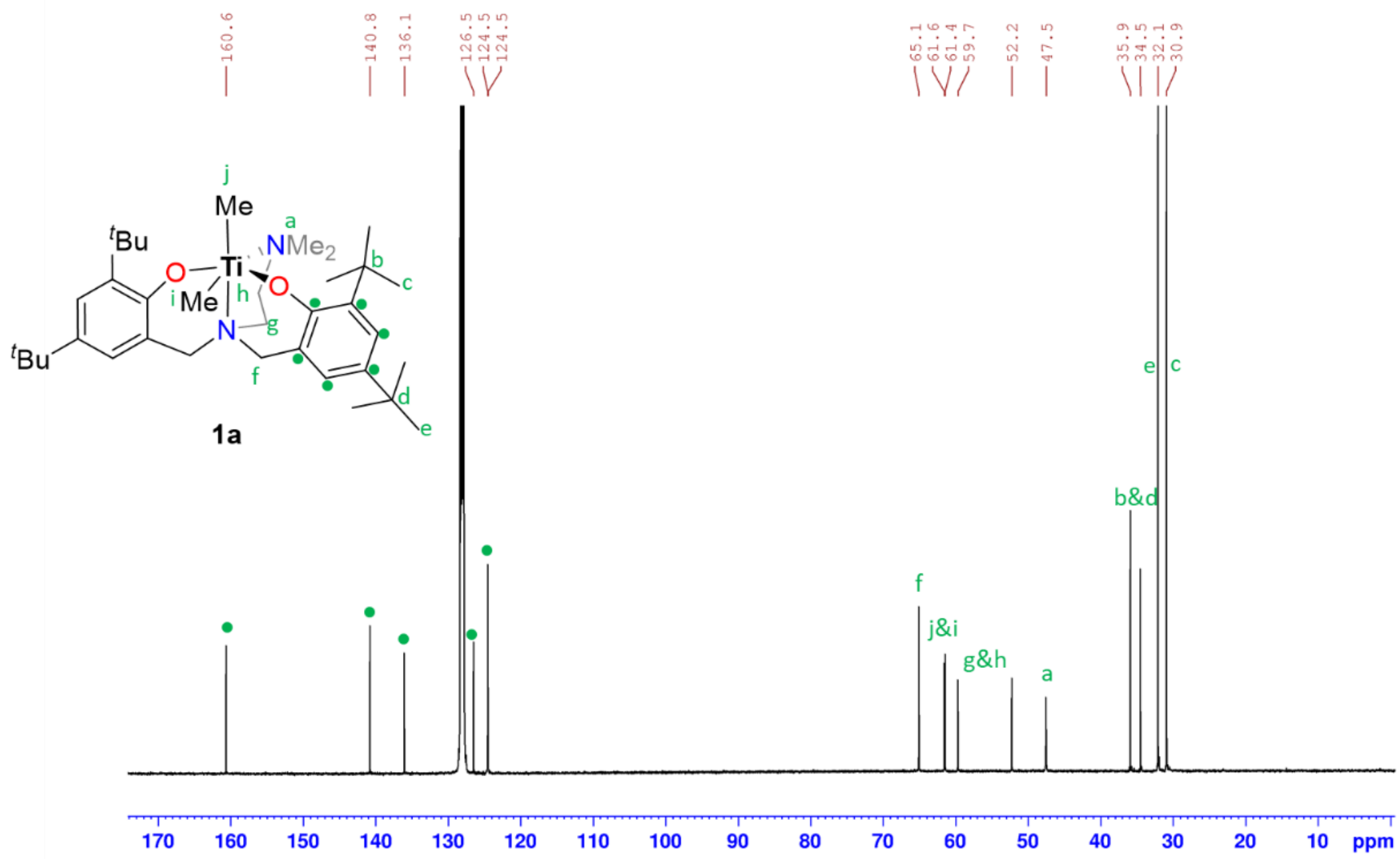

*Figure S2: ^{13}C NMR spectrum of LTiMe$_2$ **1a** in C$_6$D$_6$ (300 K).*

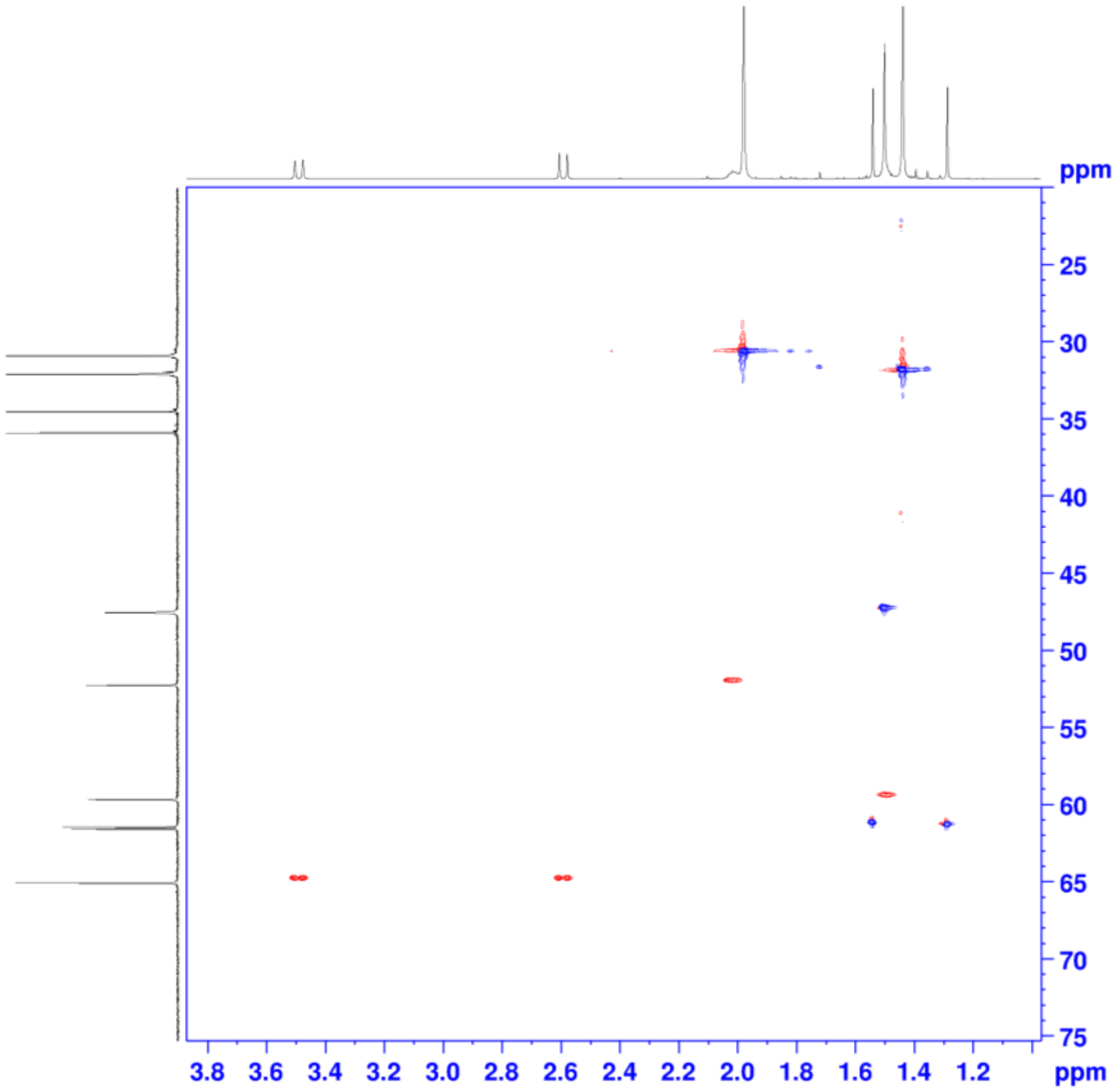

*Figure S3: HSQC NMR spectrum of LTiMe$_2$ **1a** in C_6D_6 (300 K).*

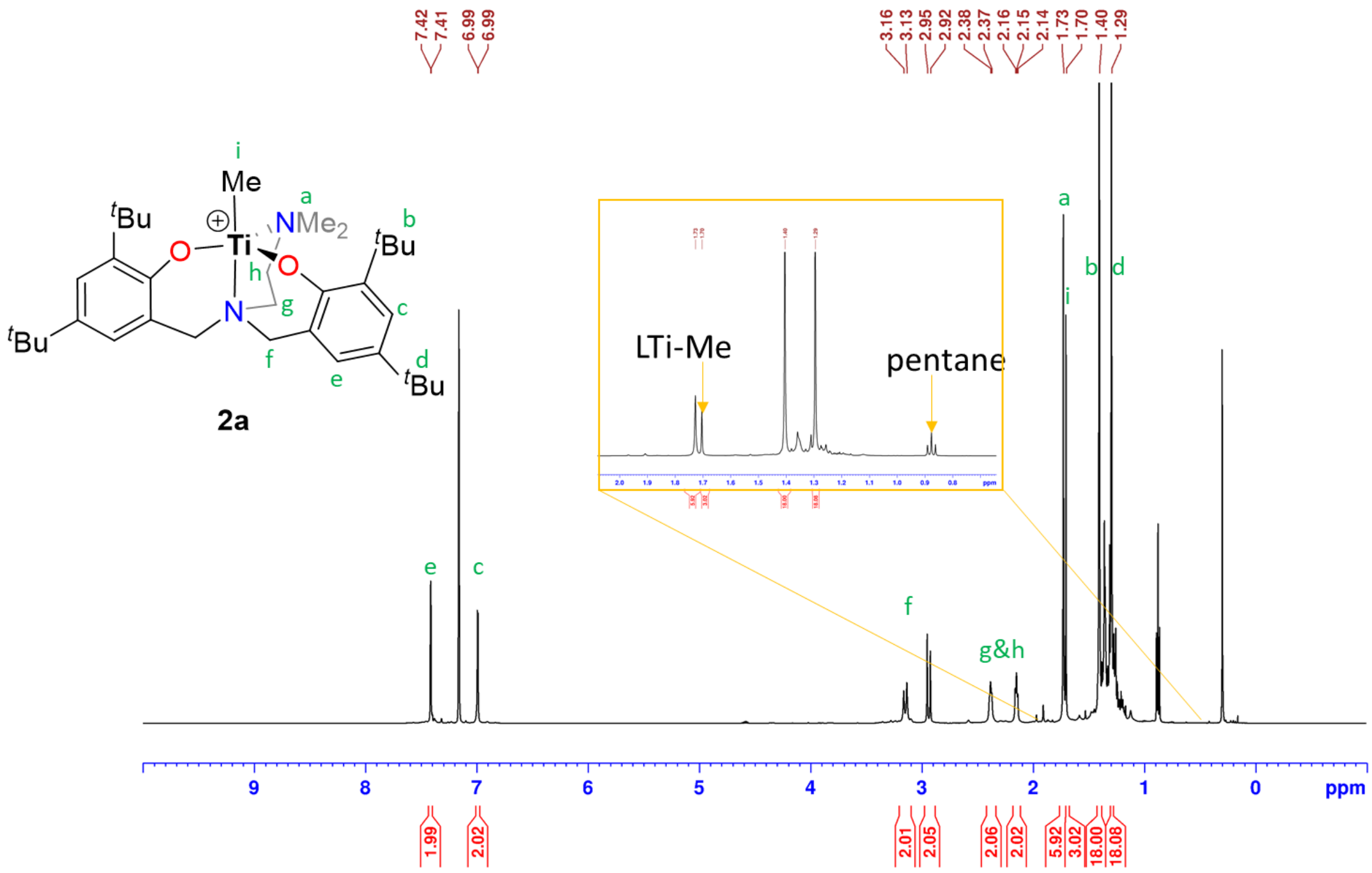

*Figure S4: ^{1}H NMR spectrum of [LTiMe][MeB(C_6F_5)$_3$] **2a** in C_6D_6 (300 K).*

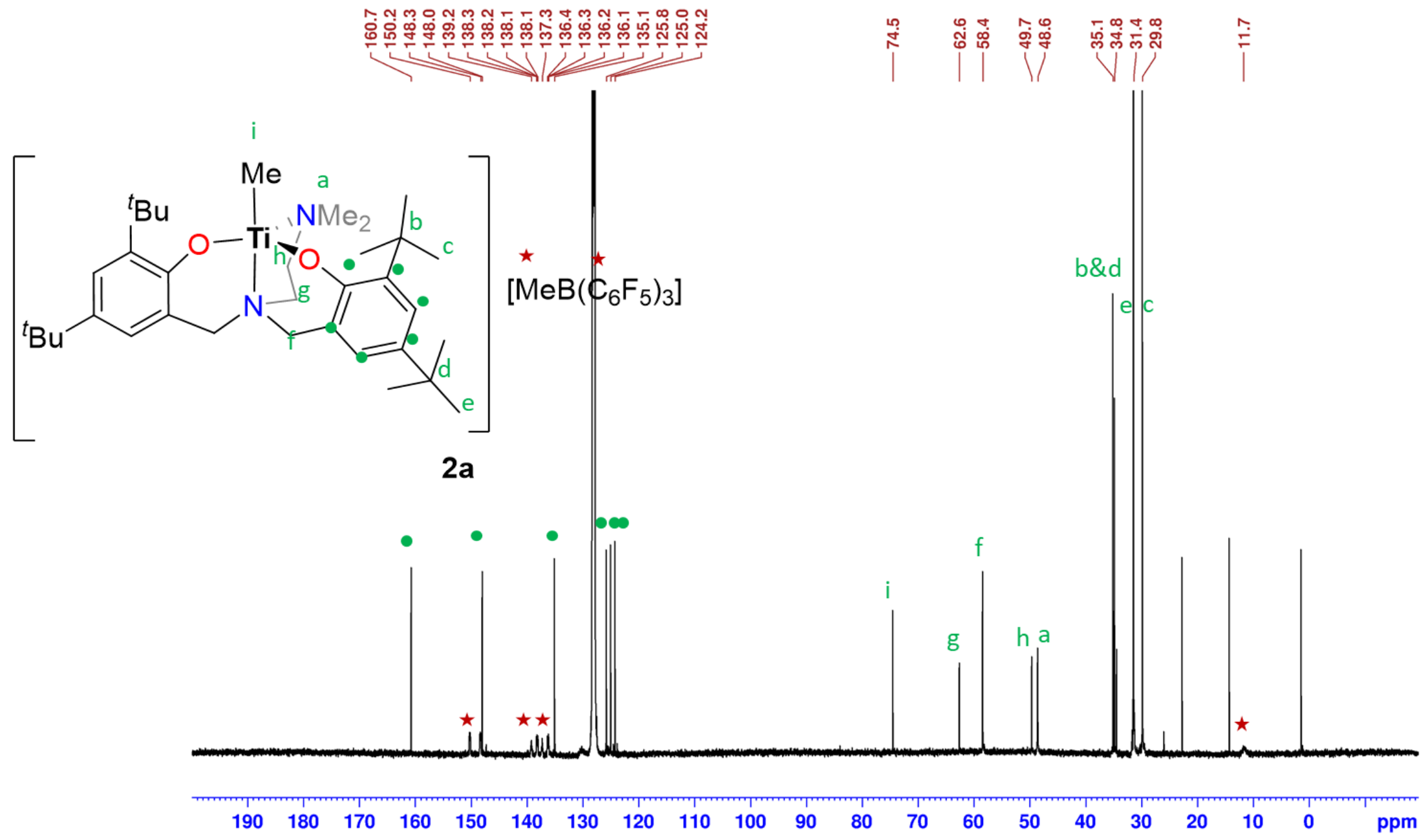

*Figure S5: ^{13}C NMR spectrum of [LTiMe][MeB(C$_6$F$_5$)$_3$] **2a** in C$_6$D$_6$ (300 K).*

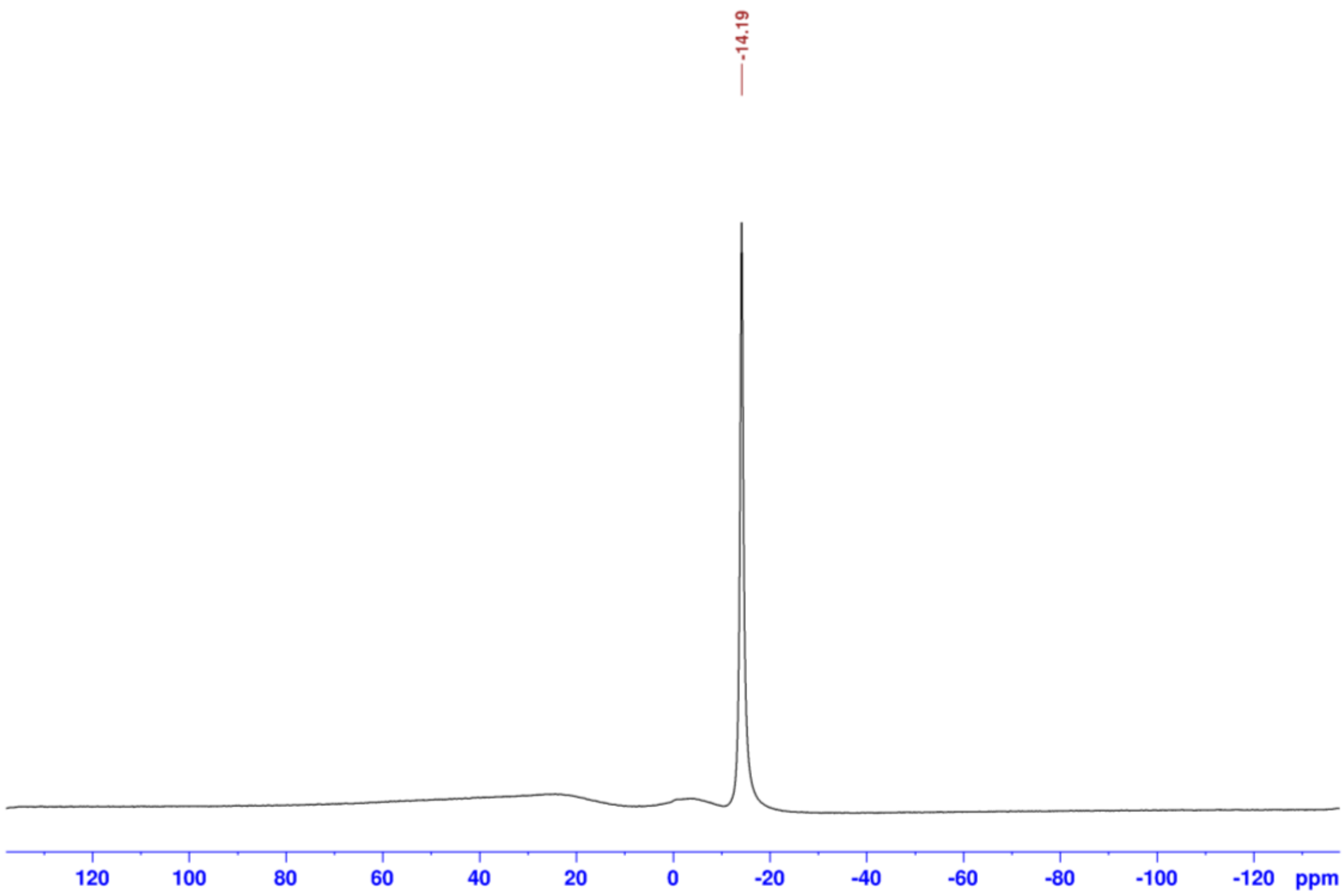

*Figure S6: ^{11}B NMR spectrum of [LTiMe][MeB(C$_6$F$_5$)$_3$] **2a** in C$_6$D$_6$ (300 K).*

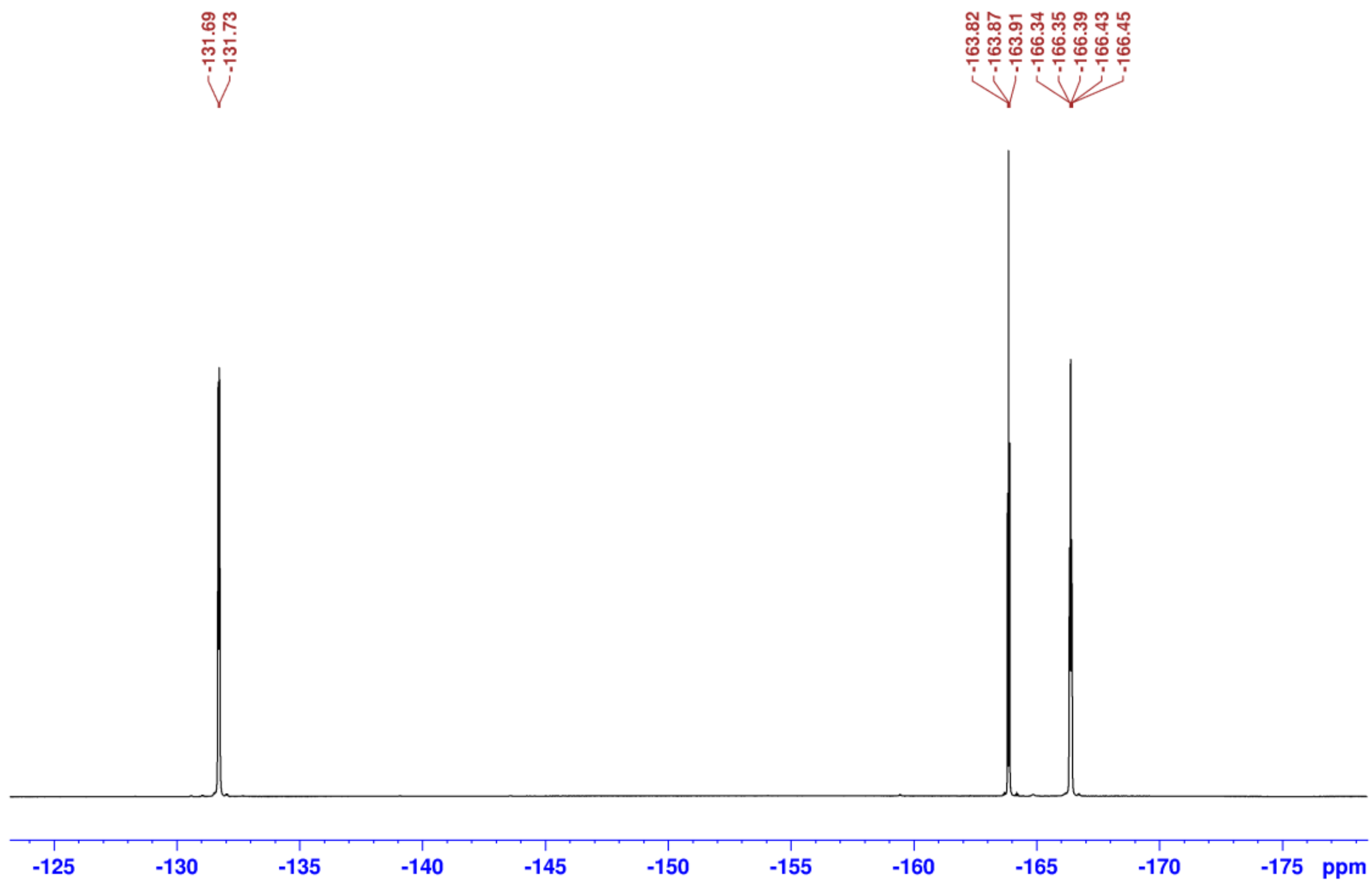

*Figure S7: ^{19}F NMR spectrum of [LTiMe][MeB(C_6F_5)$_3$] **2a** in C_6D_6 (300 K).*

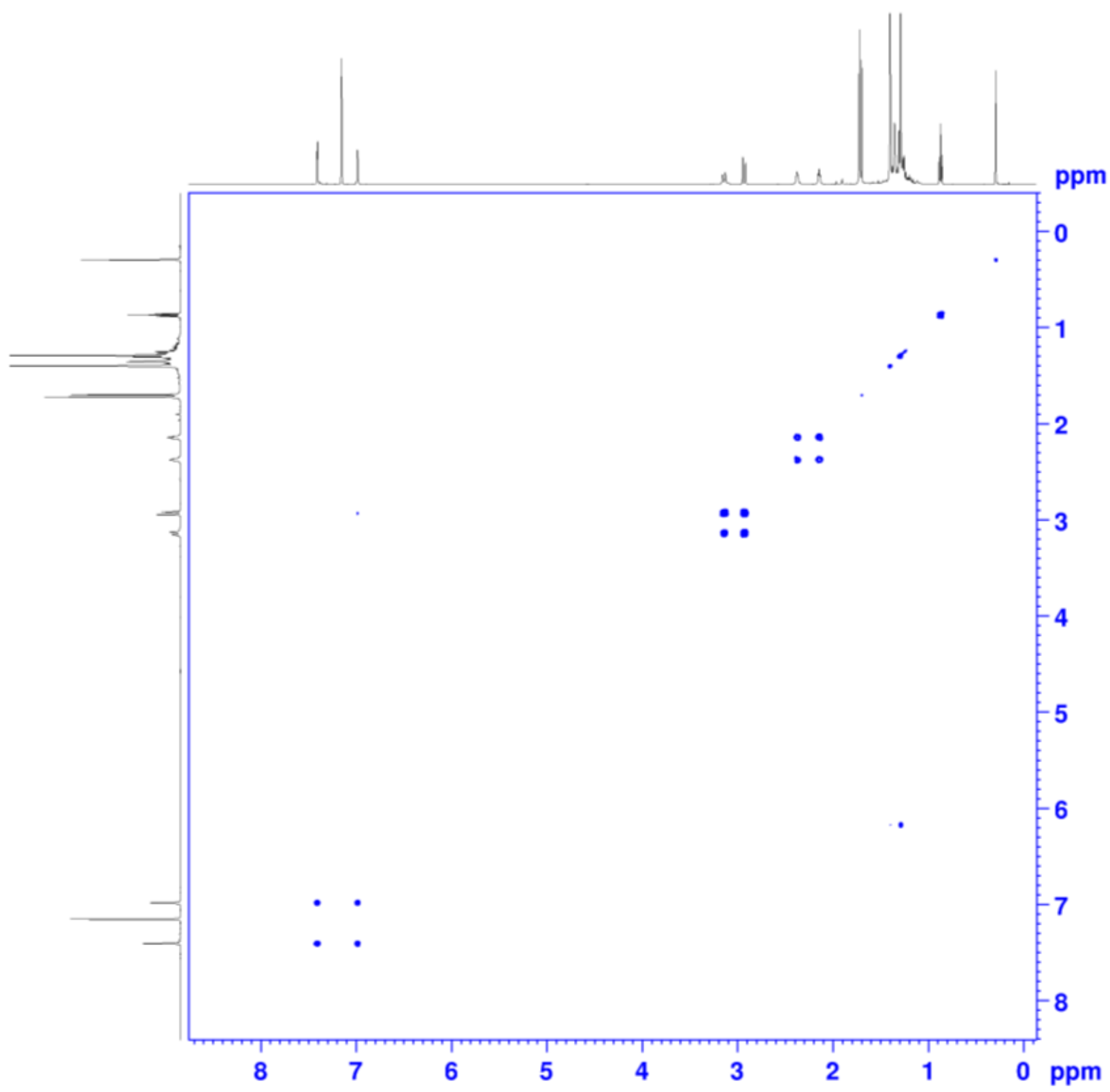

*Figure S8: COSY NMR spectrum of [LTiMe][MeB(C_6F_5)$_3$] **2a** in C_6D_6 (300 K).*

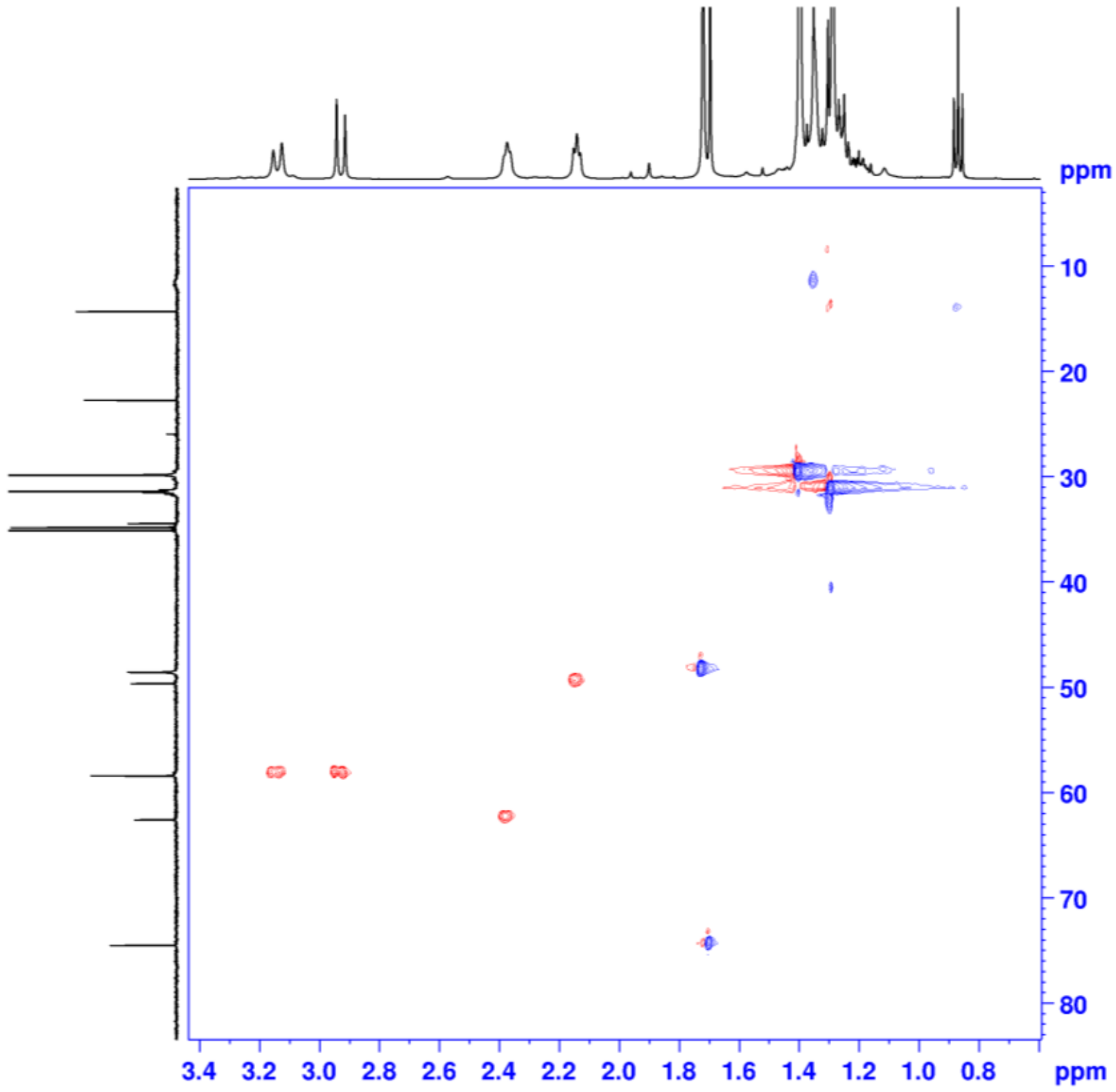

*Figure S9: HSQC NMR spectrum of [LTiMe][MeB(C_6F_5)$_3$] **2a** in C_6D_6 (300 K).*

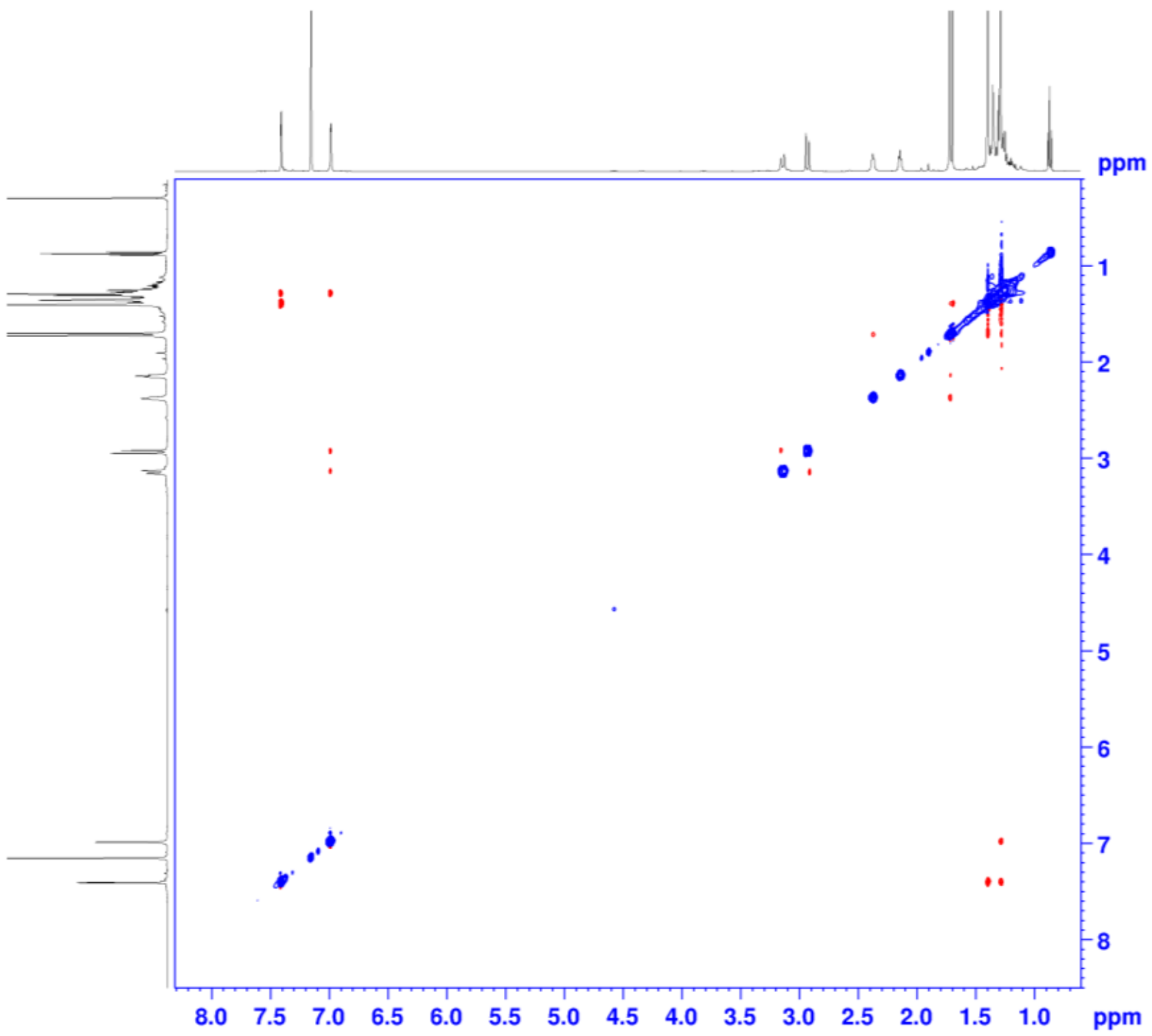

*Figure S10: NOESY NMR spectrum of [LTiMe][MeB(C_6F_5)$_3$] **2a** in C_6D_6 (300 K).*

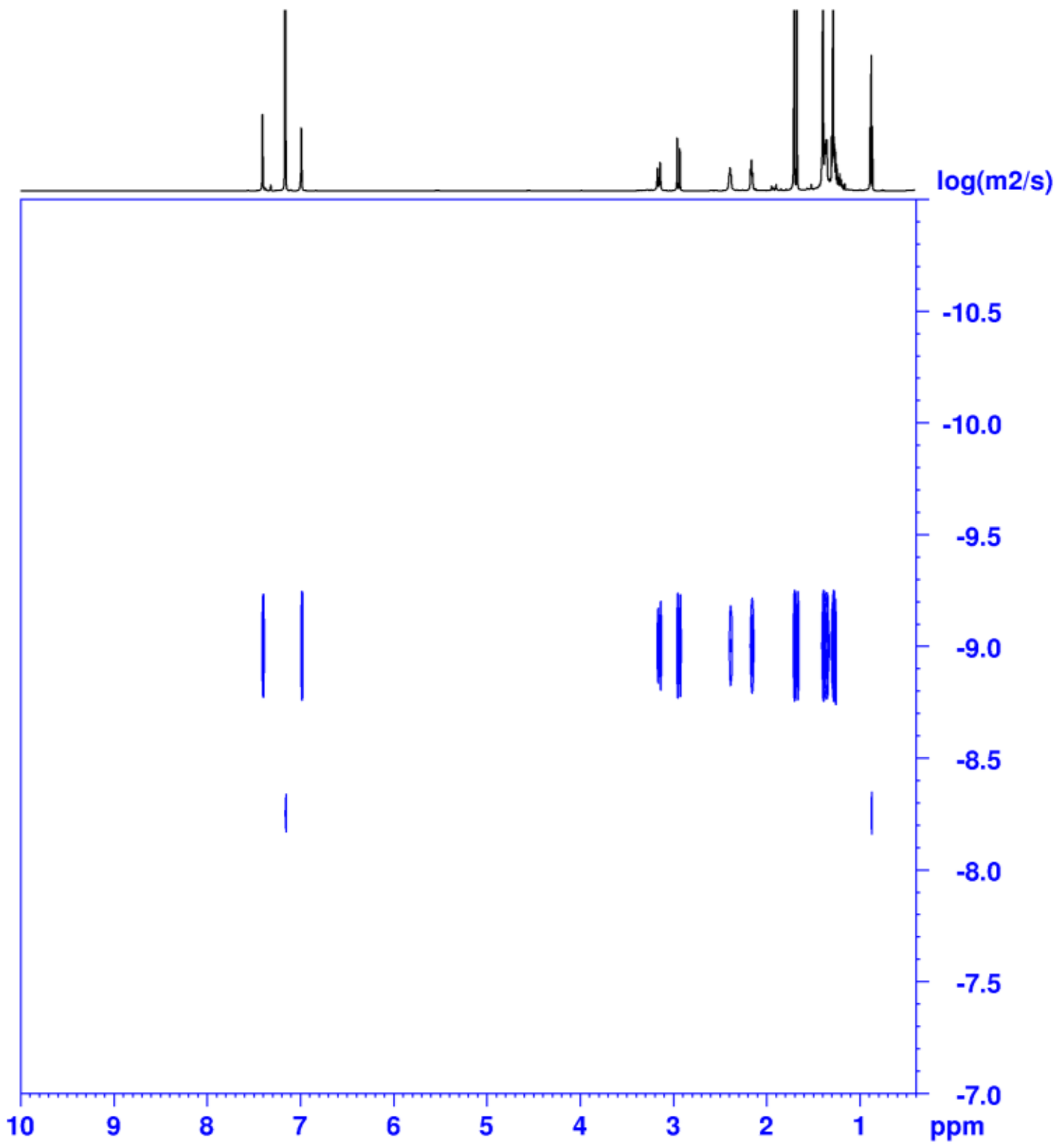

Figure S11: DOSY NMR spectrum of [LTiMe][MeB(C_6F_5)$_3$] ***2a*** *in C_6D_6 (300 K). The calculated mass is 1113 g mol^{-1} and the experimental mass is 930 g mol^{-1}.*

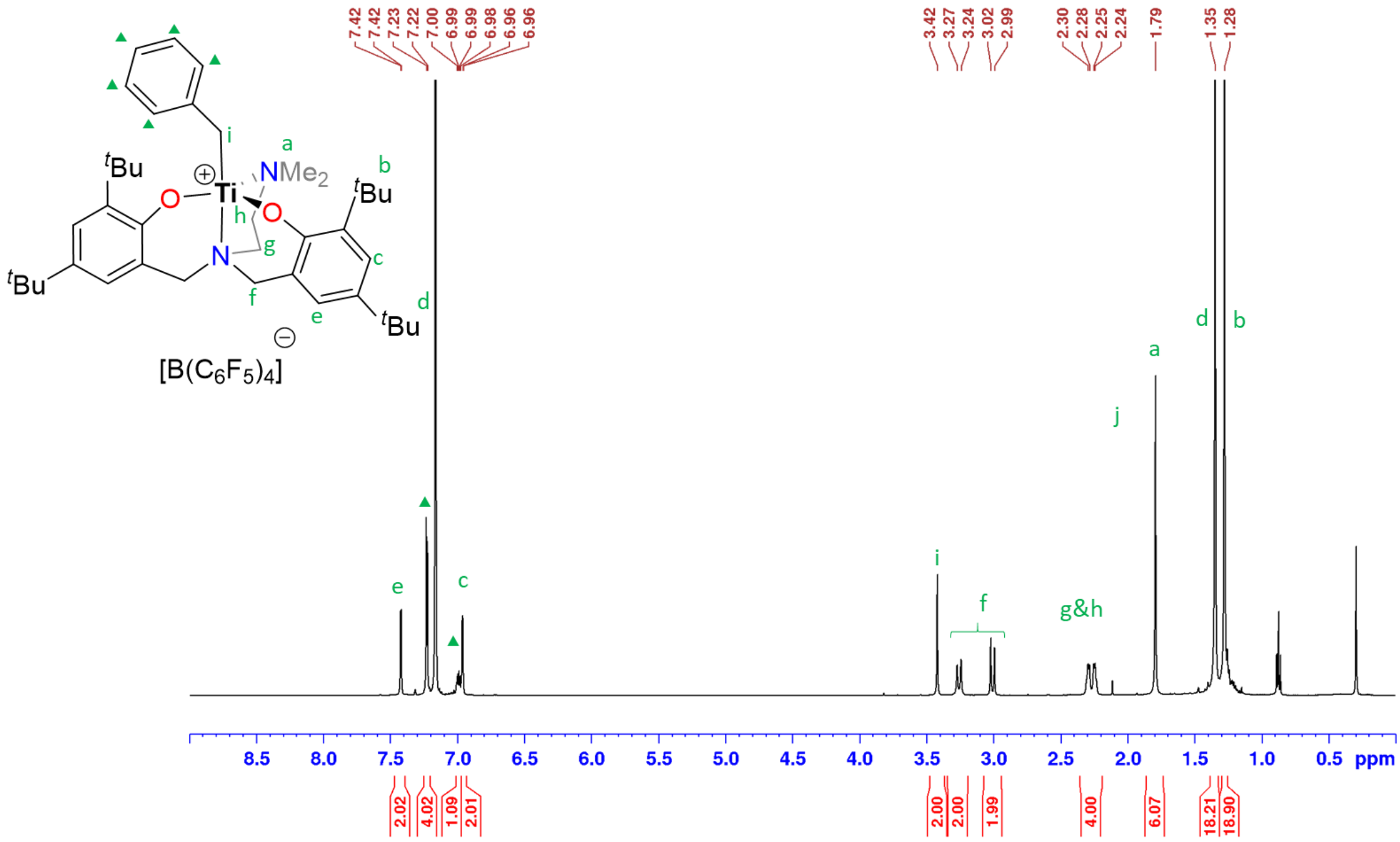

Figure S12: ^{1}H NMR spectrum of [LTiBn][B(C_6F_5)$_4$] ***2b*** *in C_6D_6 (300 K).*

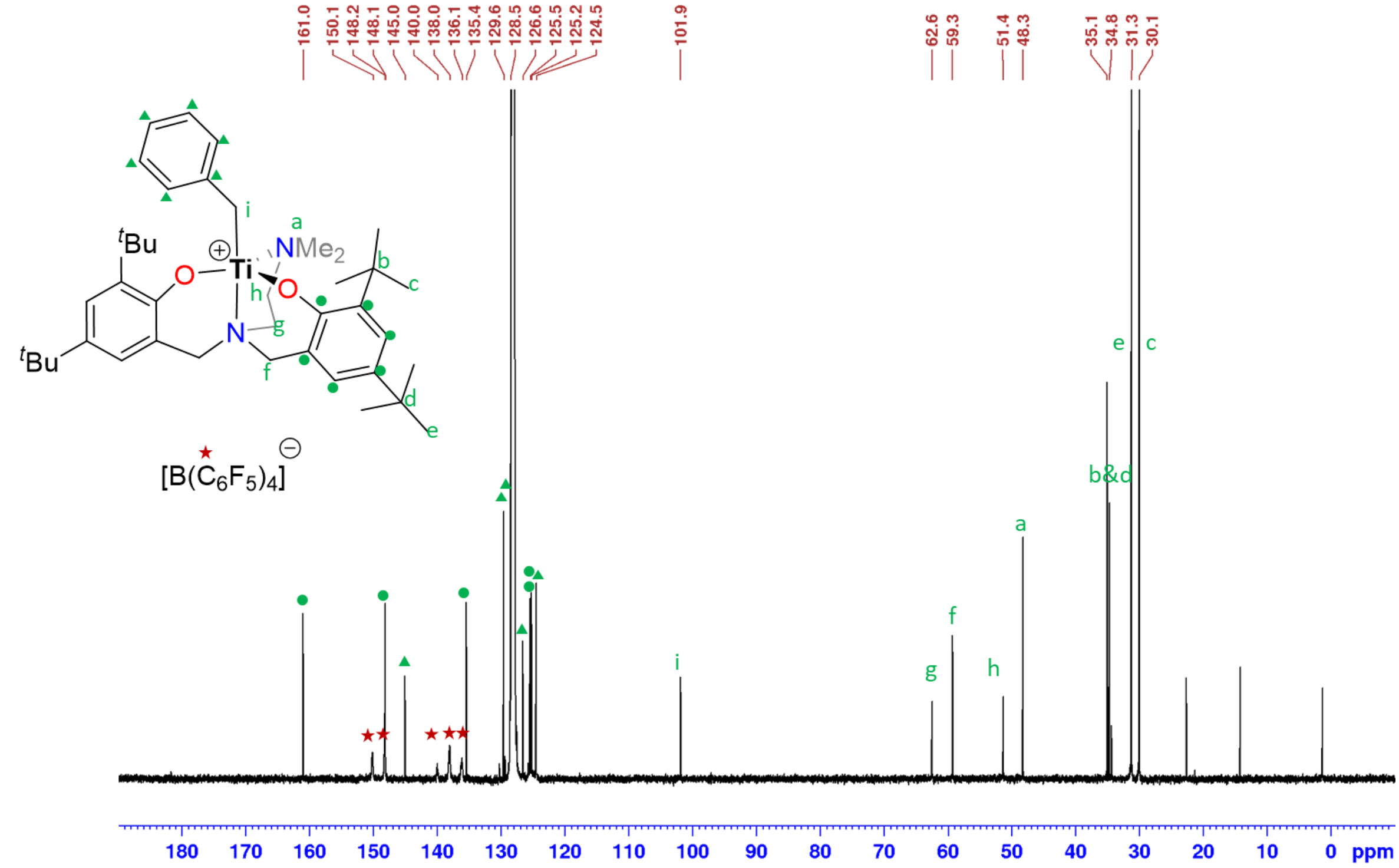

*Figure S13: ^{13}C NMR spectrum of [LTiBn][B(C_6F_5)$_4$] **2b** in C_6D_6 (300 K).*

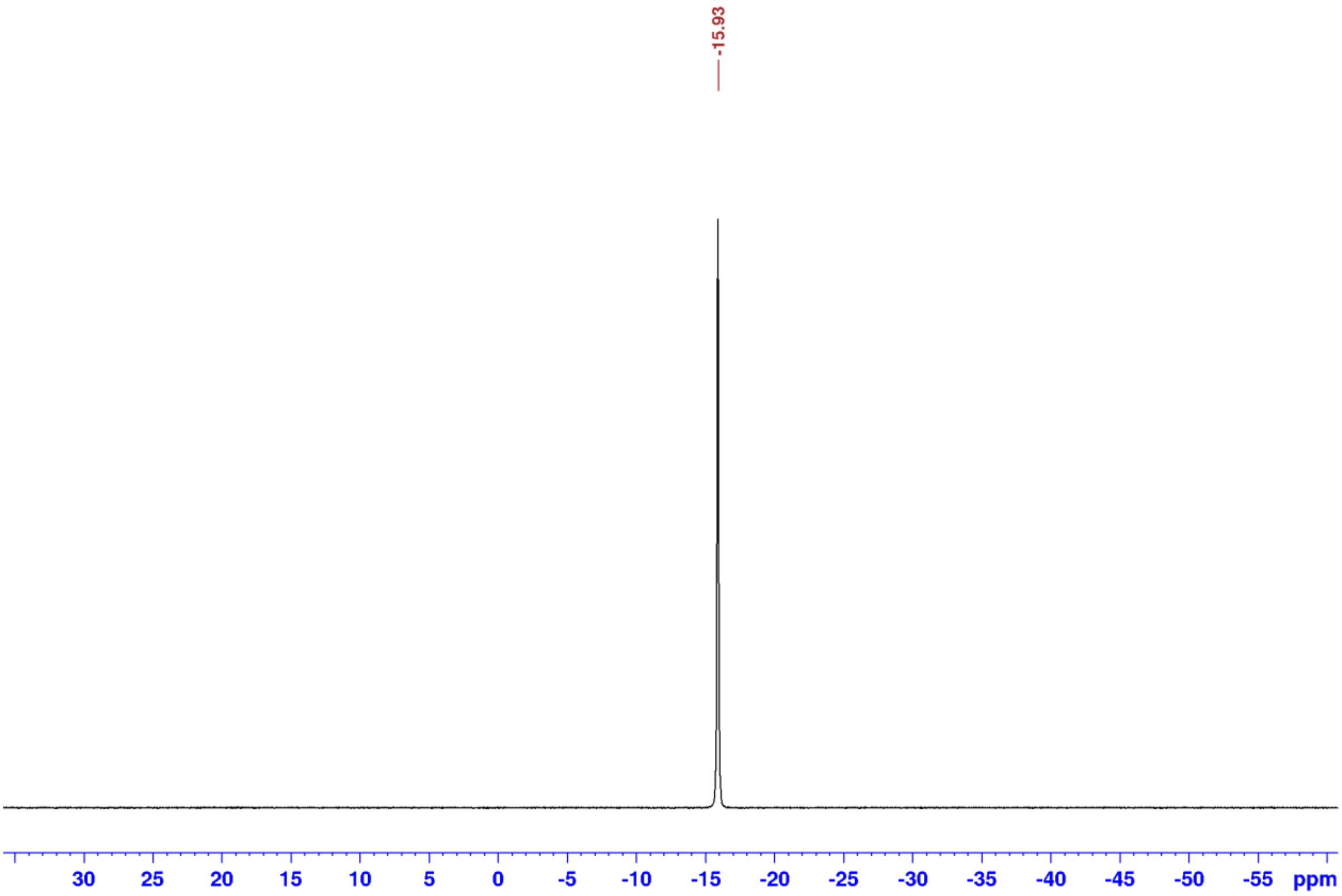

*Figure S14: ^{11}B NMR spectrum of [LTiBn][B(C_6F_5)$_4$] **2b** in C_6D_6 (300 K).*

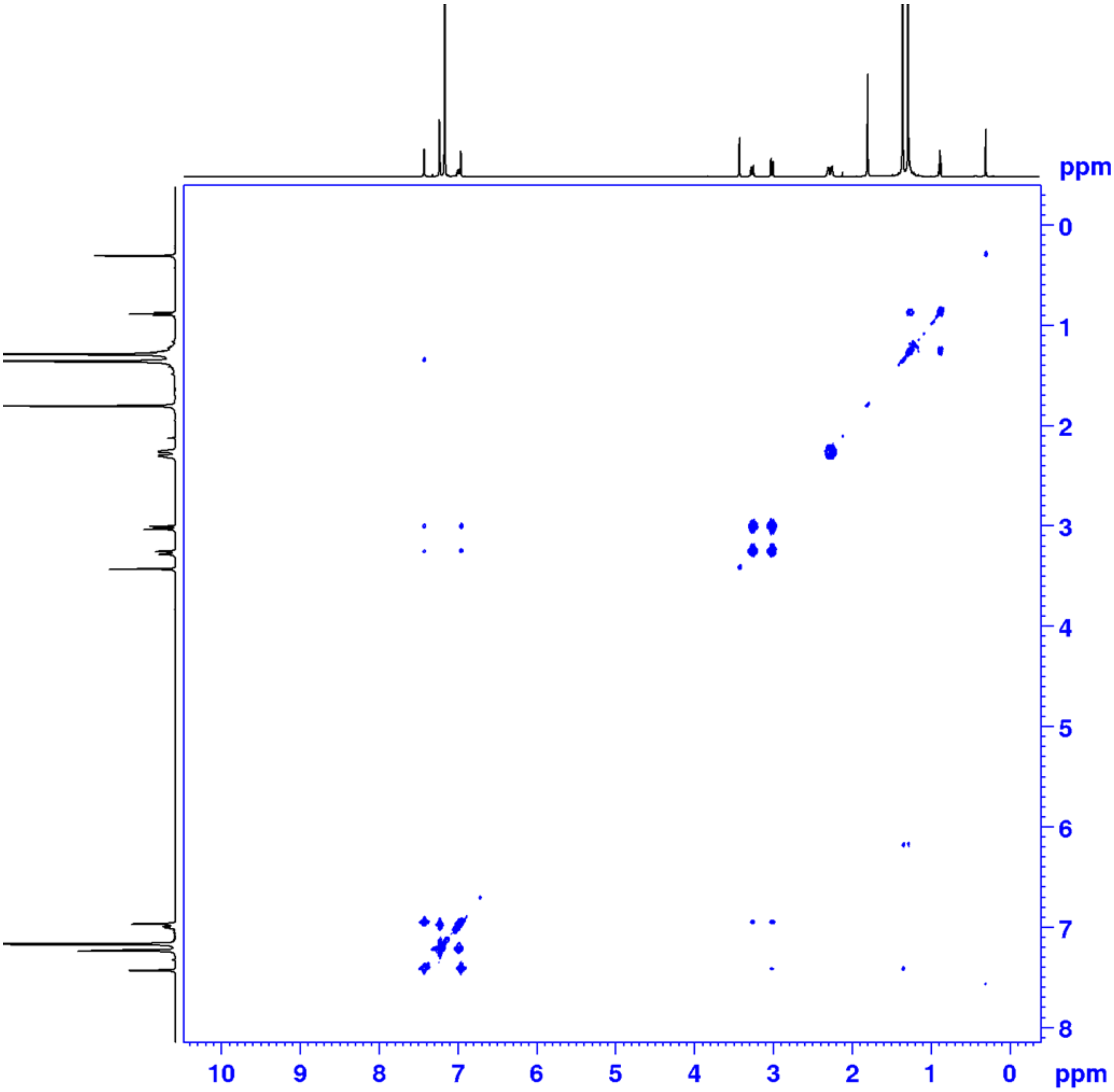

Figure S15: COSY NMR spectrum of [LTiBn][B(C_6F_5)$_4$] ***2b*** *in C_6D_6 (300 K).*

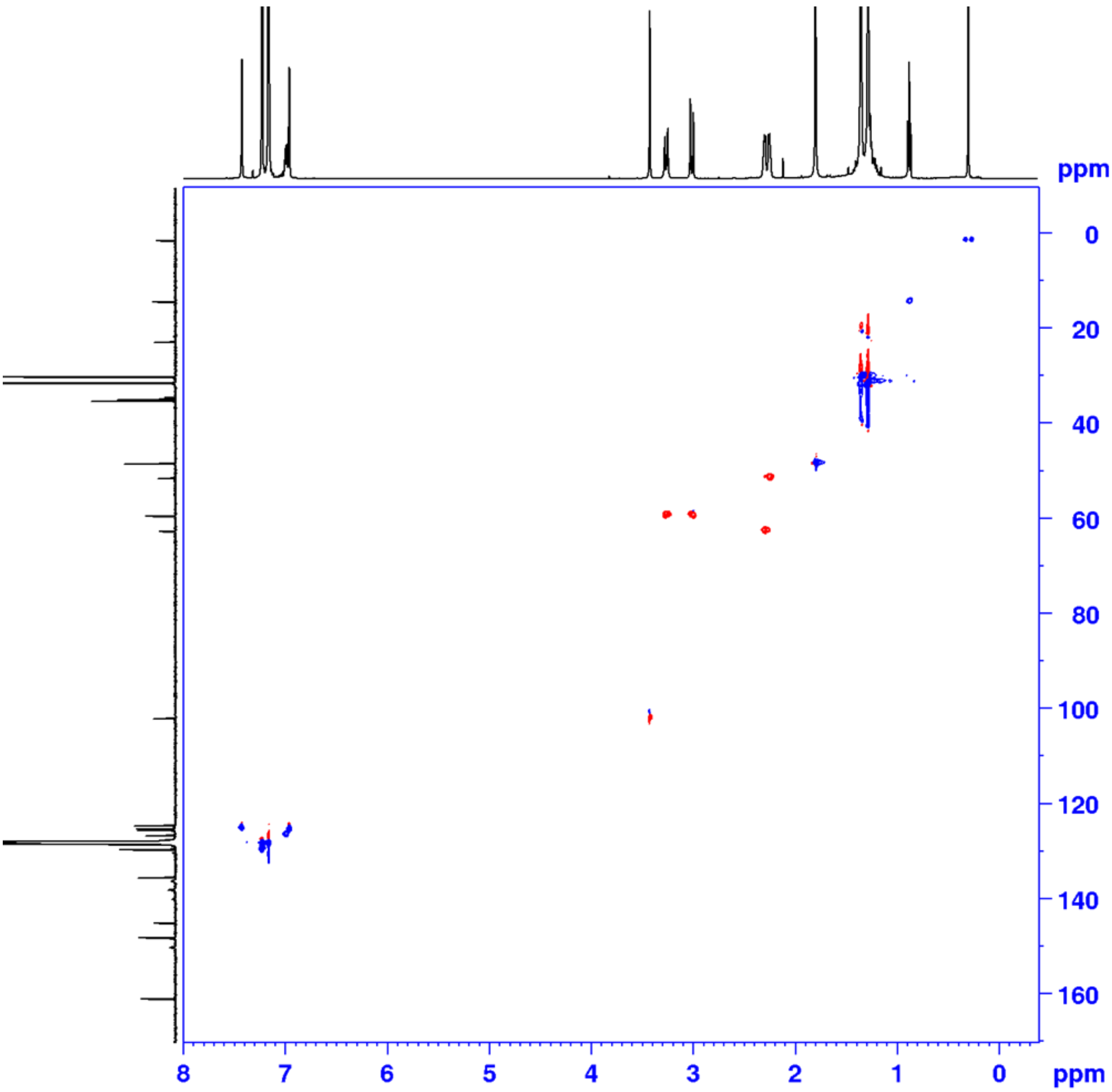

*Figure S16: HSQC NMR spectrum of [LTiBn][B(C_6F_5)$_4$] **2b** in C_6D_6 (300 K).*

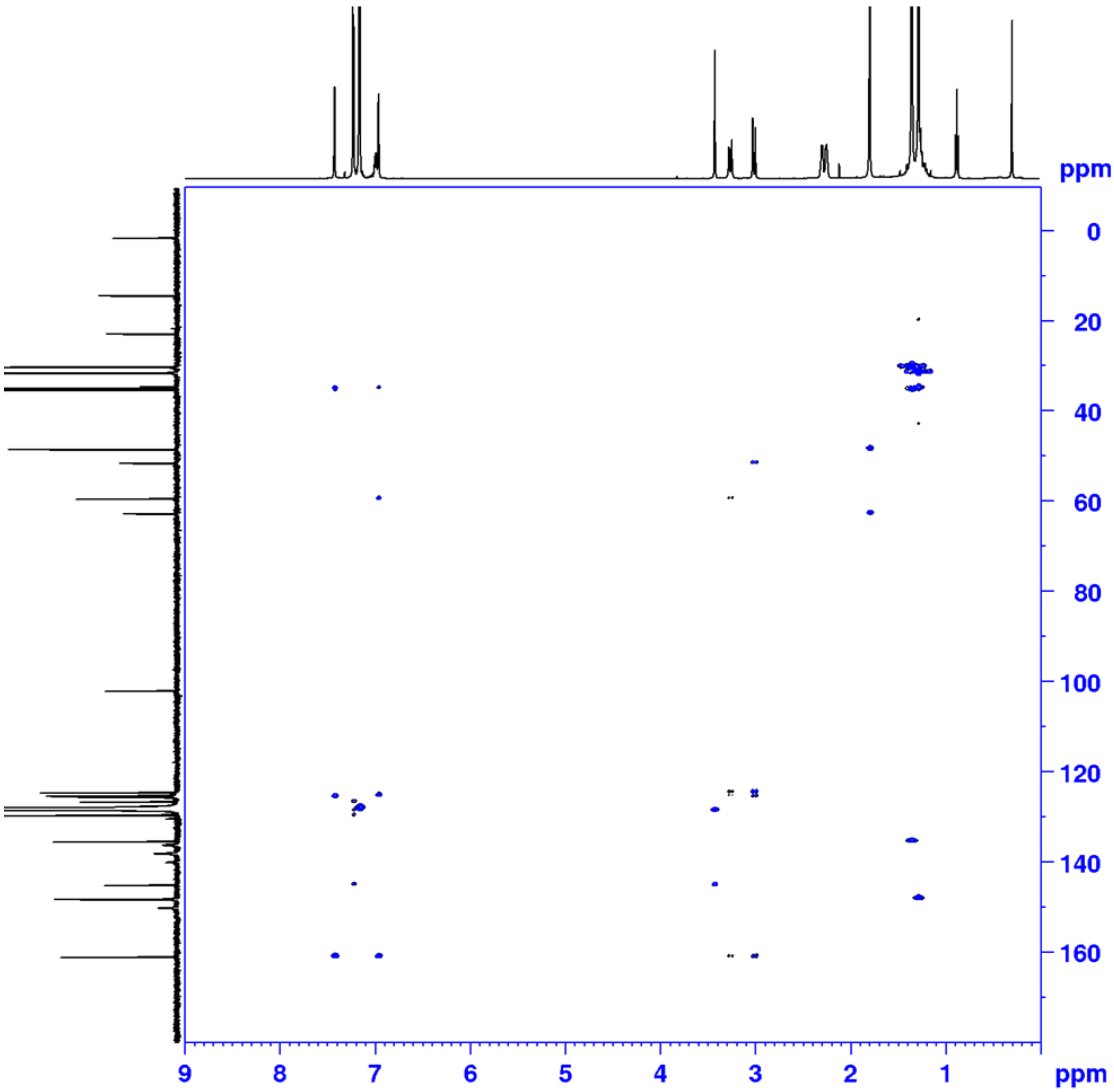

Figure S17: HMBC NMR spectrum of [LTiBn][B(C_6F_5)$_4$] ***2b*** *in C_6D_6 (300 K).*

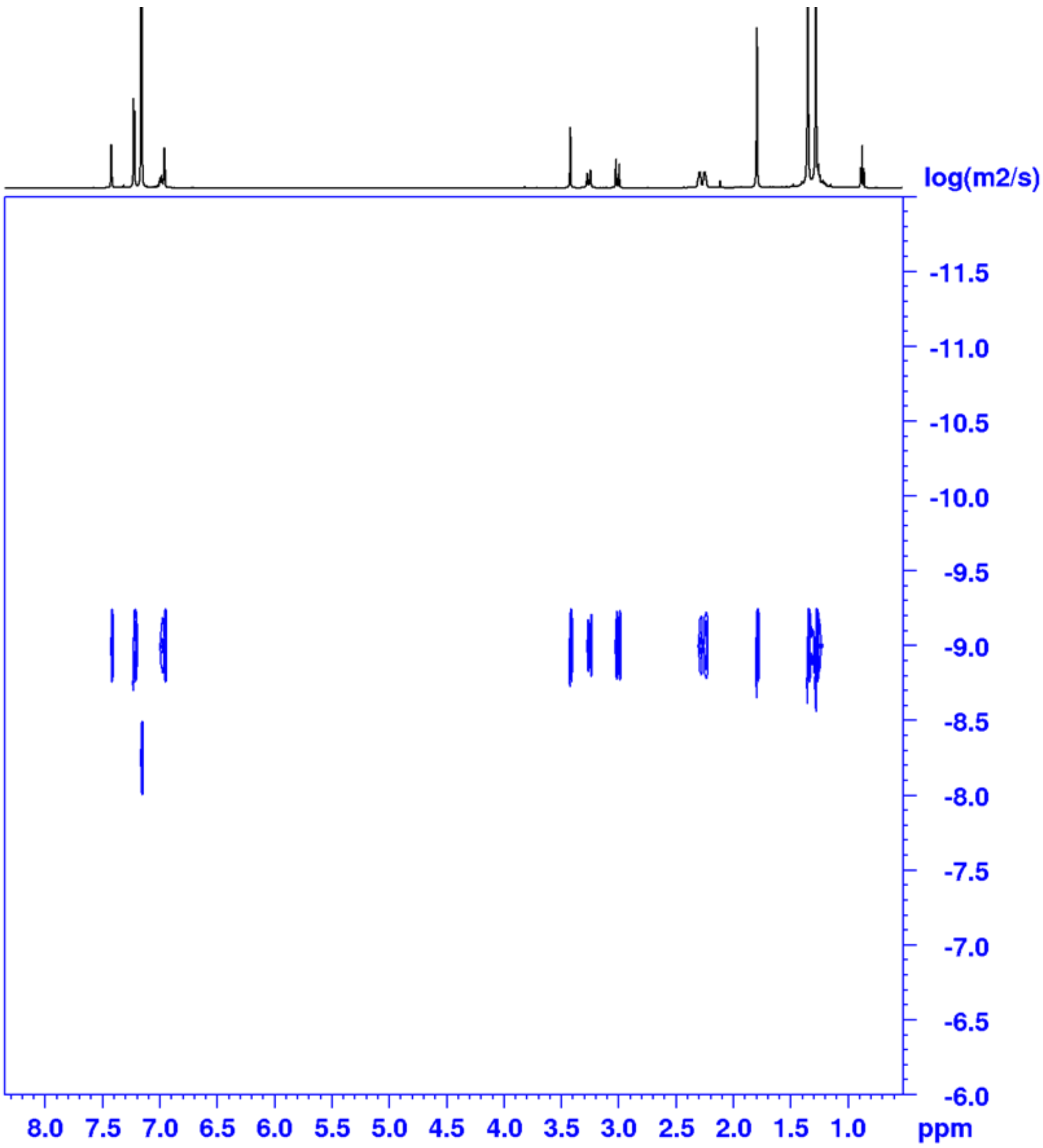

Figure S18: DOSY NMR spectrum of [LTiBn][B(C_6F_5)$_4$] ***2b*** *in C_6D_6 (300 K). Calculated mass is 1340.86 and the experimental mass is 1225.*

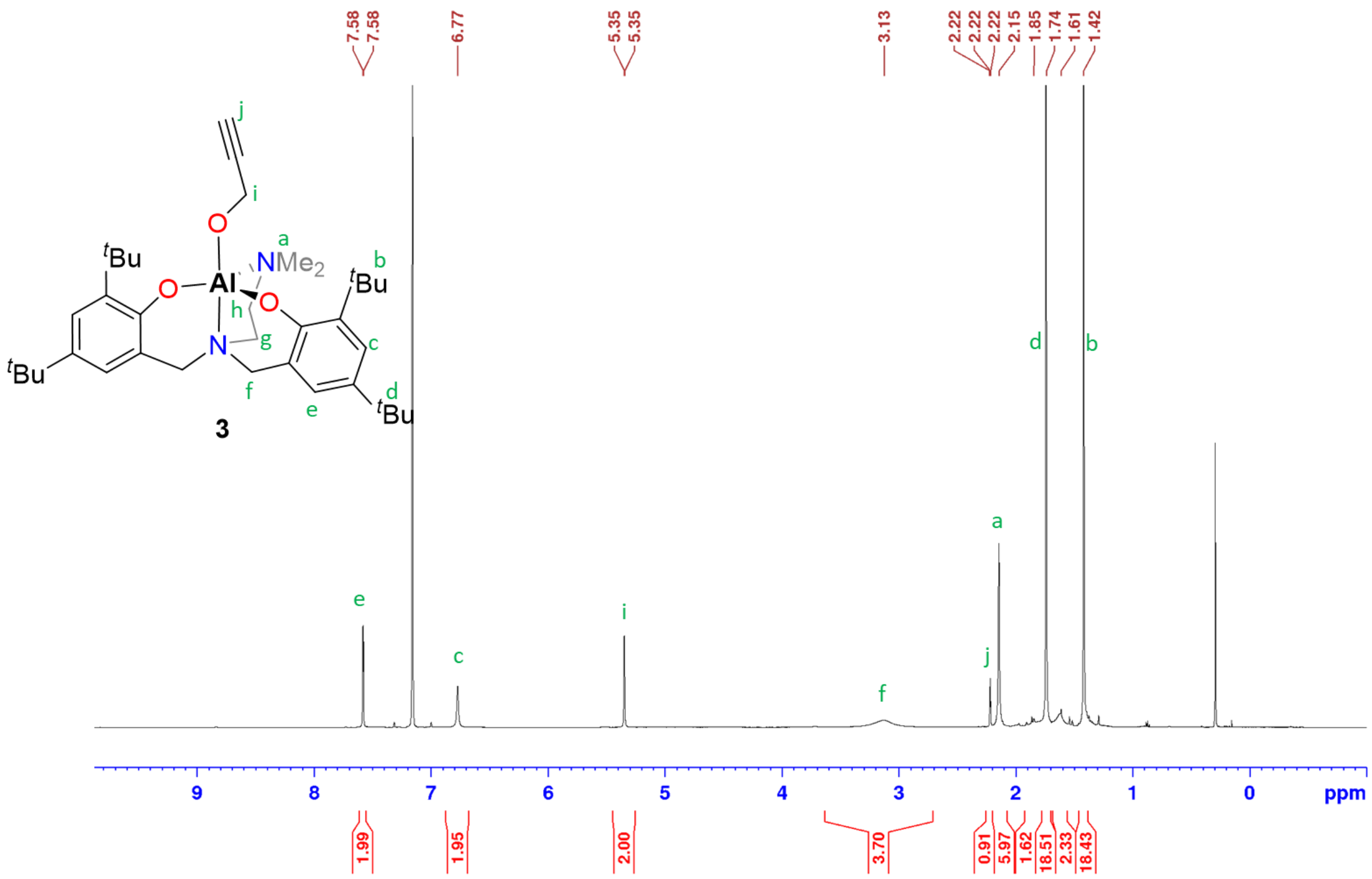

*Figure S19: ^{1}H NMR spectrum of $LAlOCH_2C{\equiv}CH$ **3** in C_6D_6 (300 K).*

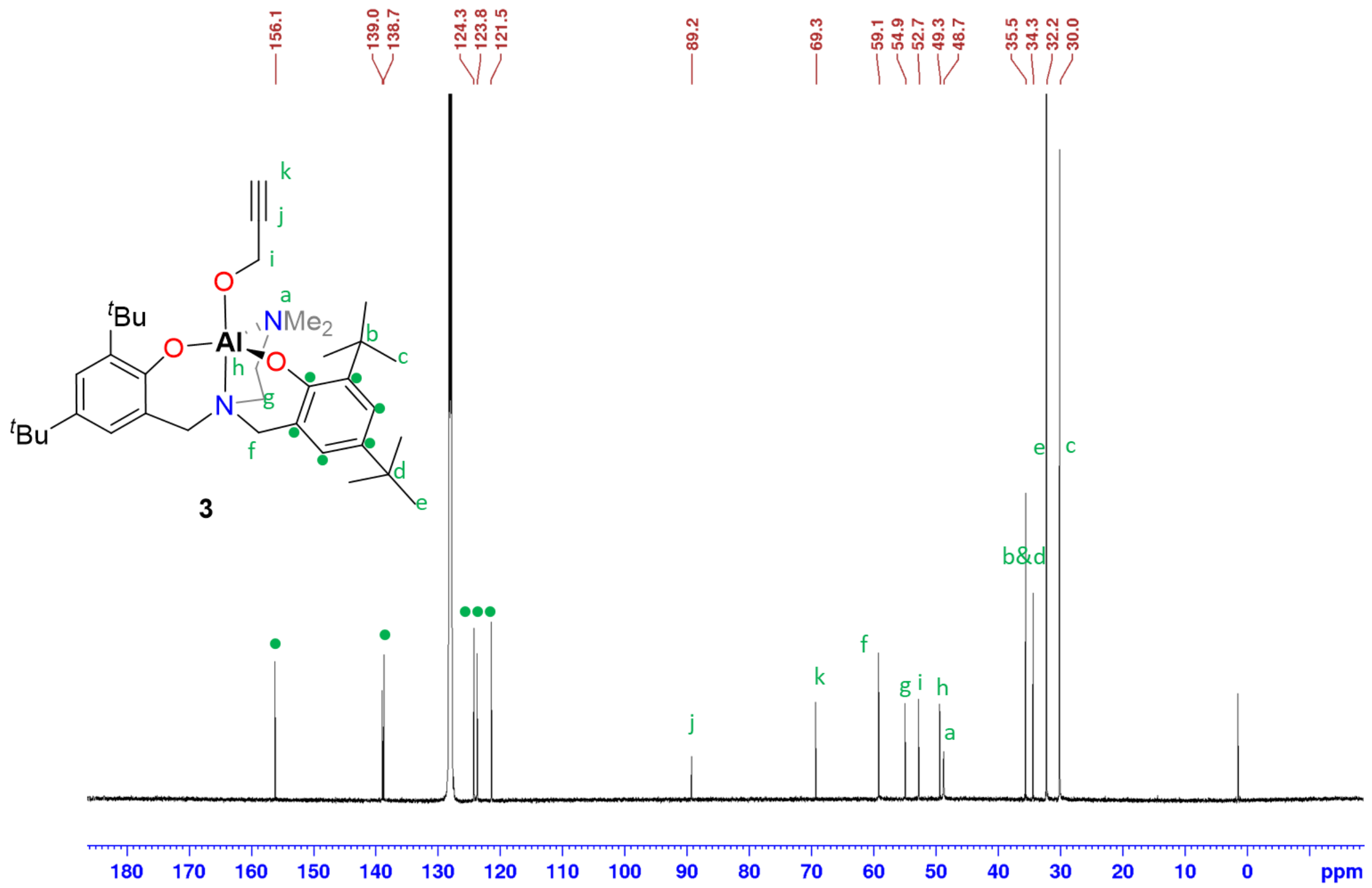

*Figure S20: ^{13}C NMR spectrum of $LAlOCH_2C{\equiv}CH$ **3** in C_6D_6 (300 K).*

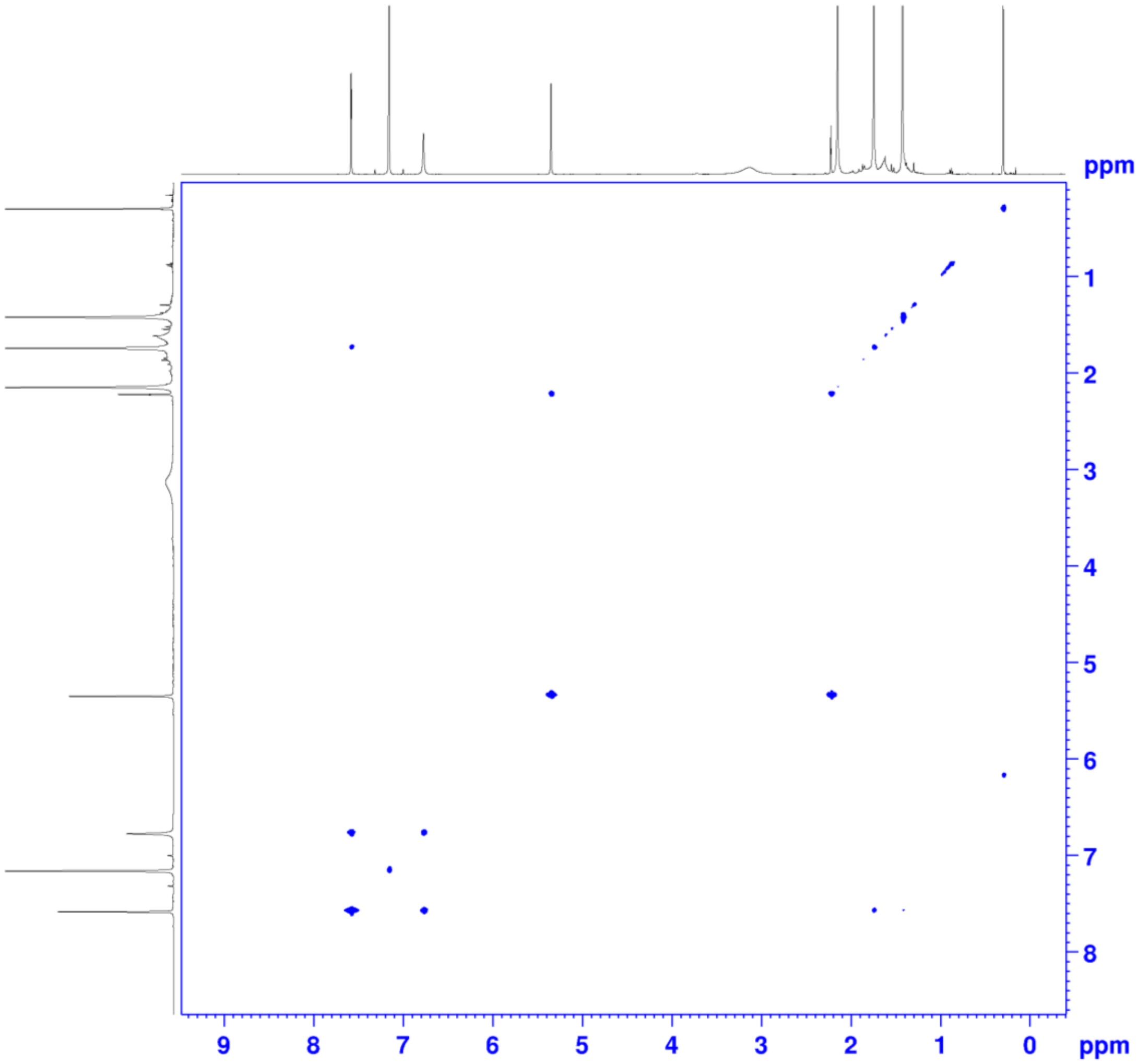

*Figure S21: COSY NMR spectrum of LAlOCH$_2$C≡CH **3** in C_6D_6 (300 K).*

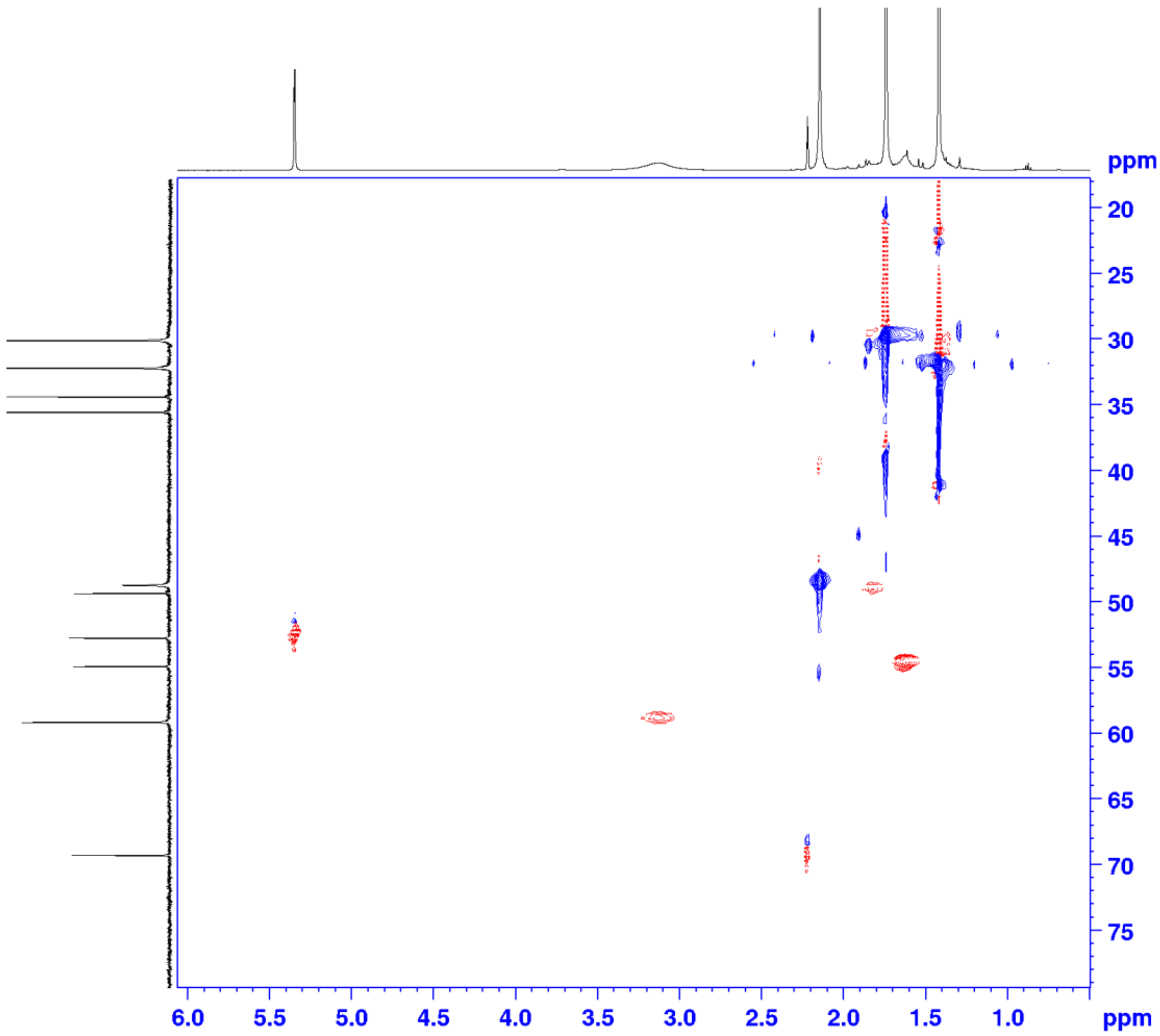

*Figure S22: HSQC NMR spectrum of LAlOCH$_2$C≡CH **3** in C$_6$D$_6$ (300 K).*

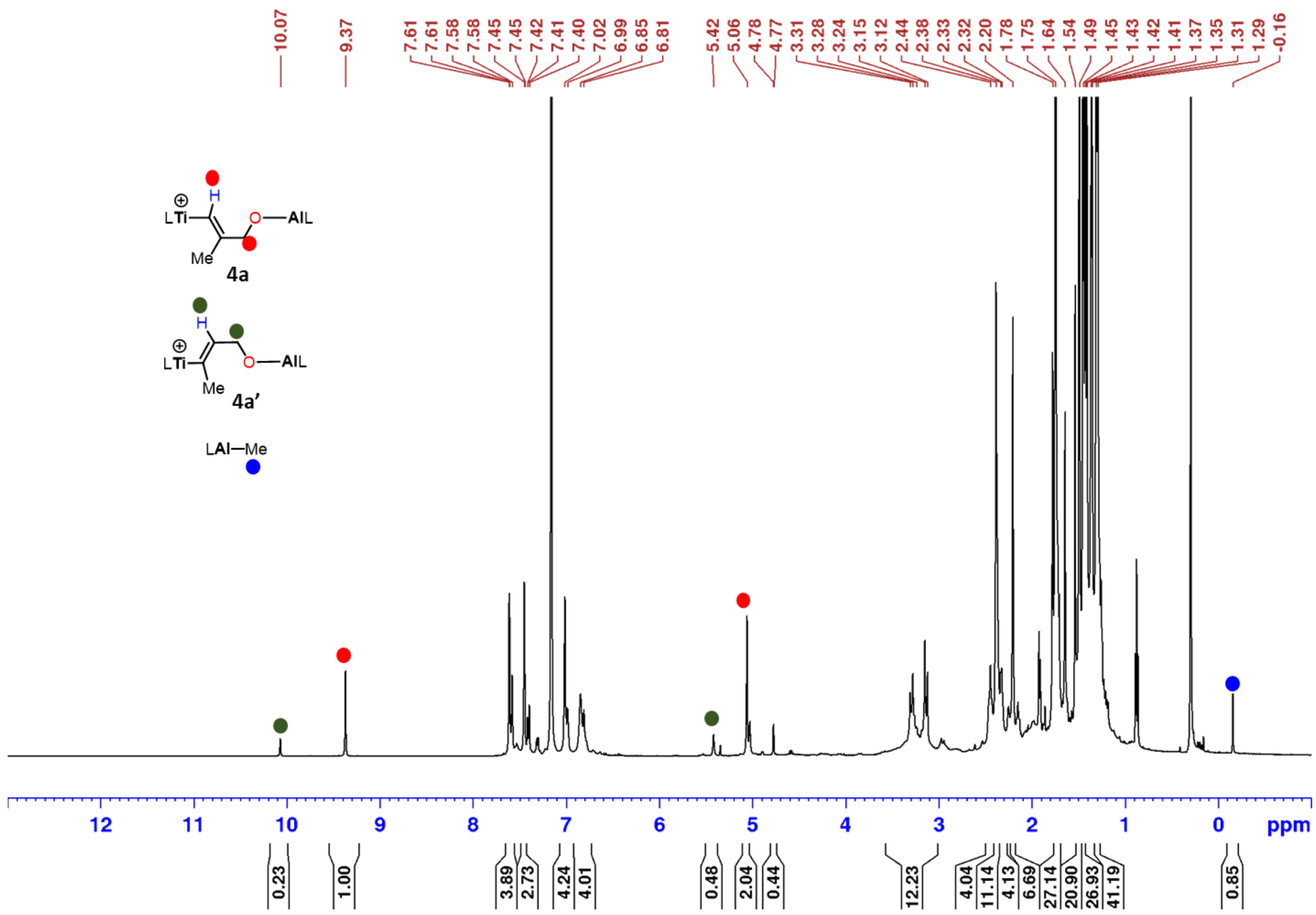

*Figure S23: ¹H NMR spectrum of equimolar [LTiMe][MeB(C_6F_5)$_3$] **2a** and LAlOCH$_2$C≡CH **3** combined in C_6D_6 (300 K) (see Scheme S1 for additional details). The characteristic peaks for **4a** and **4a'** were assigned based on the ¹H, ¹³C, COSY and HSQC spectra, and by comparison to the heavier group 4 zirconium alkenyl complexes reported in literature.[6] LAlMe was synthesised and characterised following literature protocols,[4] and used as the precursor to LAlOCH$_2$C≡CH **3**. The combination of **2a** and **3** regenerated key resonances for LAlMe, indicating that metathesis occurred between LAlOCH$_2$C≡CH **3** and [LTiMe] to generate LAlMe.*

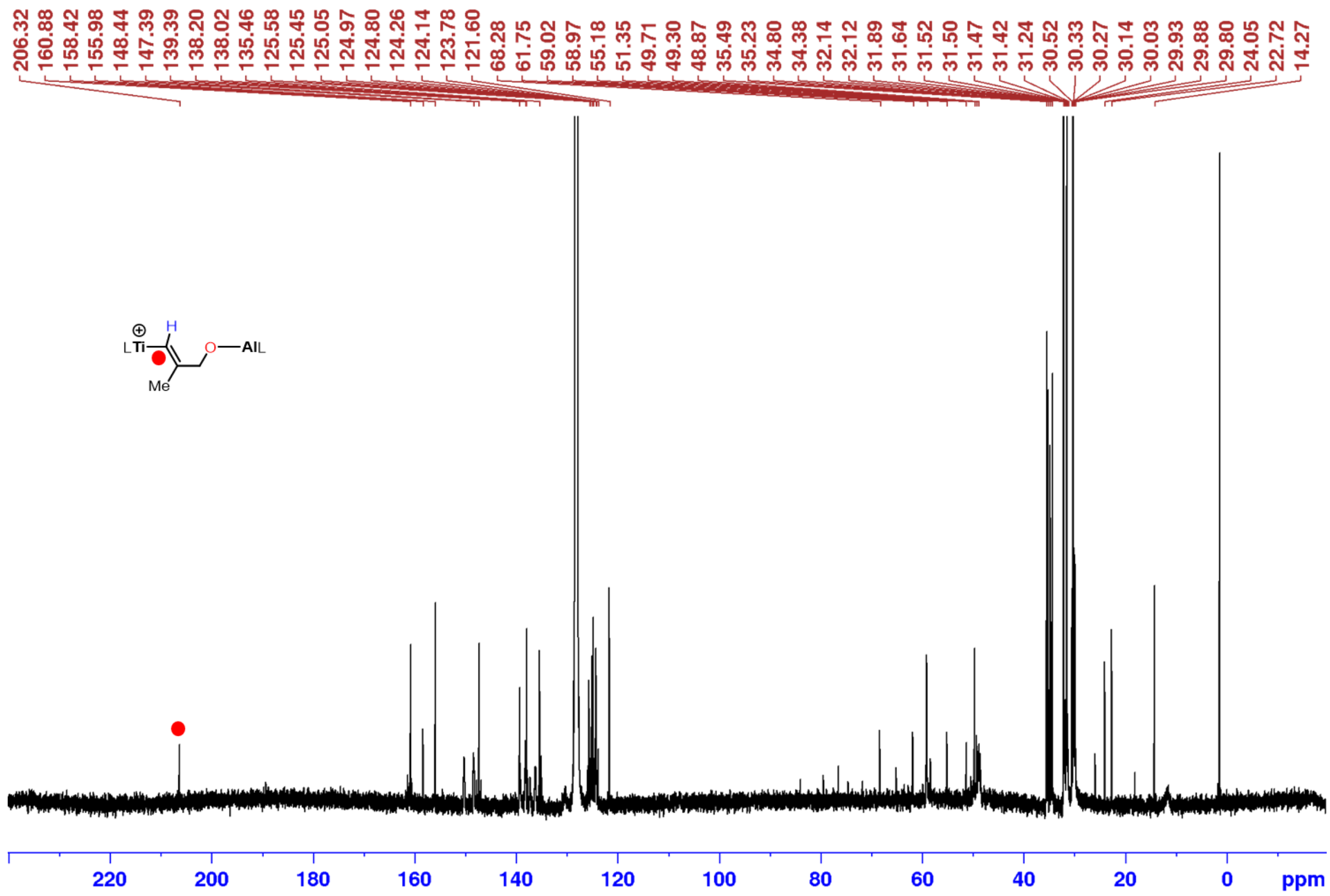

*Figure S24: ^{13}C NMR spectrum of equimolar [LTiMe][MeB(C_6F_5)$_3$] **2a** and LAlOCH$_2$C≡CH **3** in C_6D_6 (300 K). The characteristic Ti-CH$_{alkene}$ peak was assigned based on the HSQC spectrum and literature precedence for similar compounds.[6]*

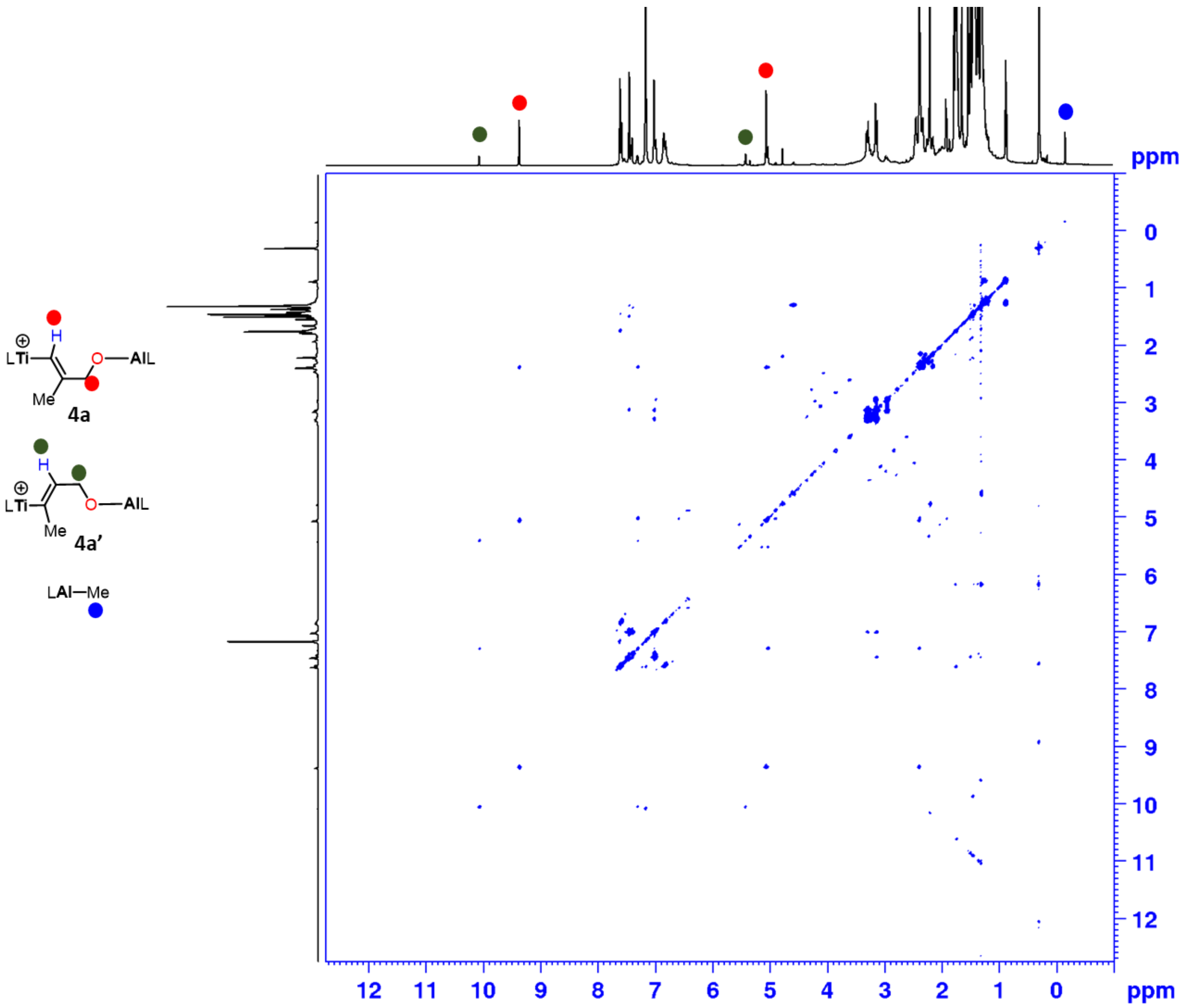

*Figure S25: COSY NMR spectrum of equimolar [LTiMe][MeB(C_6F_5)$_3$] **2a** and LAlOCH$_2$C≡CH **3** combined in C_6D_6 (300 K).*

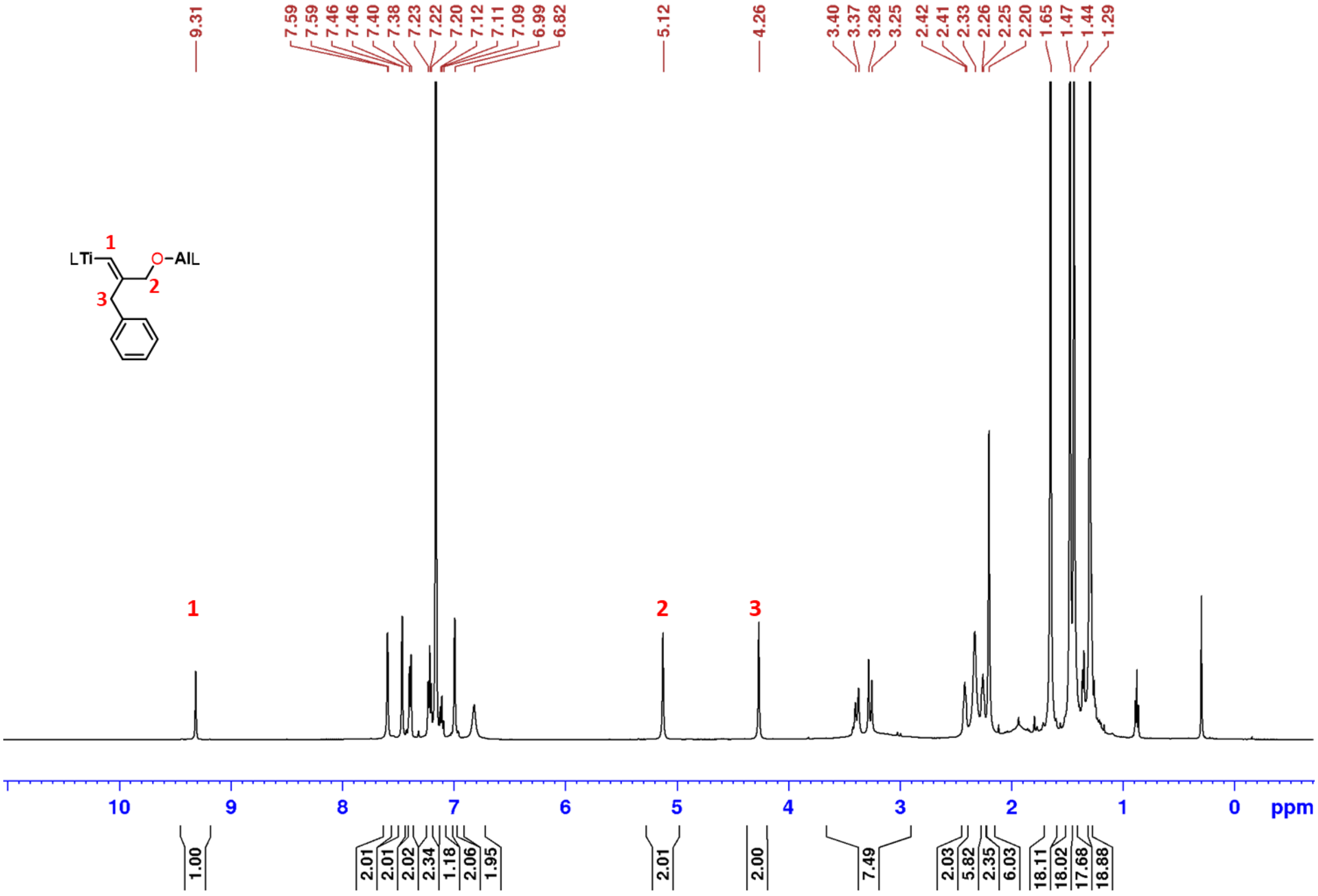

*Figure S26: ^{1}H NMR spectrum of [LTiCH=CBnCH$_2$OAlL][B(C$_6$F$_5$)$_4$] **4b** (generated in situ from equimolar [LTiBn][B(C$_6$F$_5$)$_4$] **2b** and LAlOCH$_2$C≡CH **3**) in C$_6$D$_6$ (300 K).*

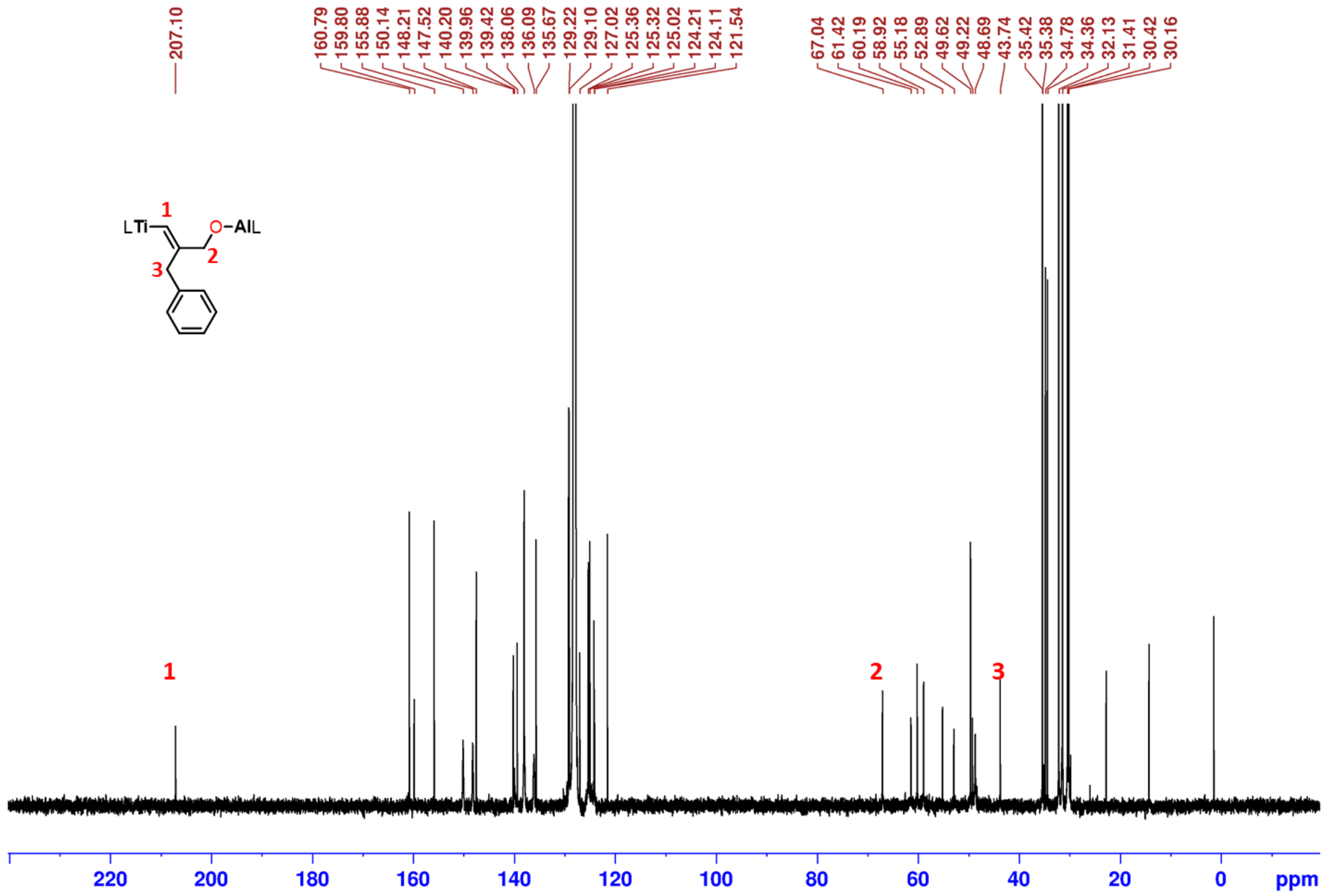

*Figure S27: ^{13}C NMR spectrum of [LTiCH=CBnCH$_2$OAlL][B(C$_6$F$_5$)$_4$] **4b** (generated in situ from equimolar [LTiBn][B(C$_6$F$_5$)$_4$] **2b** and LAlOCH$_2$C≡CH **3**) in C$_6$D$_6$ (300 K).*

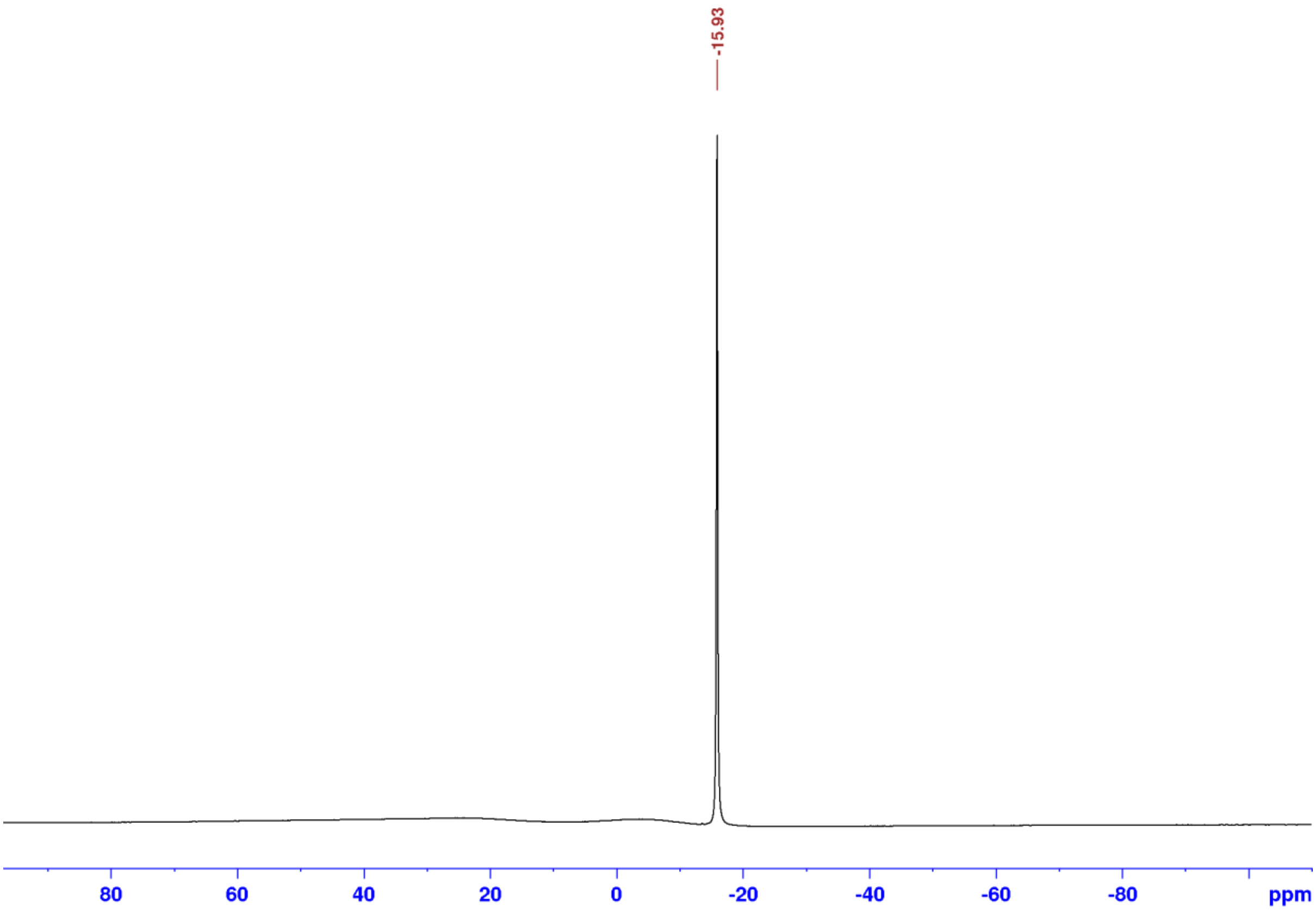

*Figure S28: ^{11}B NMR spectrum of [LTiCH=CBnCH$_2$OAlL][B(C$_6$F$_5$)$_4$] **4b** (generated in situ from equimolar [LTiBn][B(C$_6$F$_5$)$_4$] **2b** and LAlOCH$_2$C≡CH **3**) in C$_6$D$_6$ (300 K).*

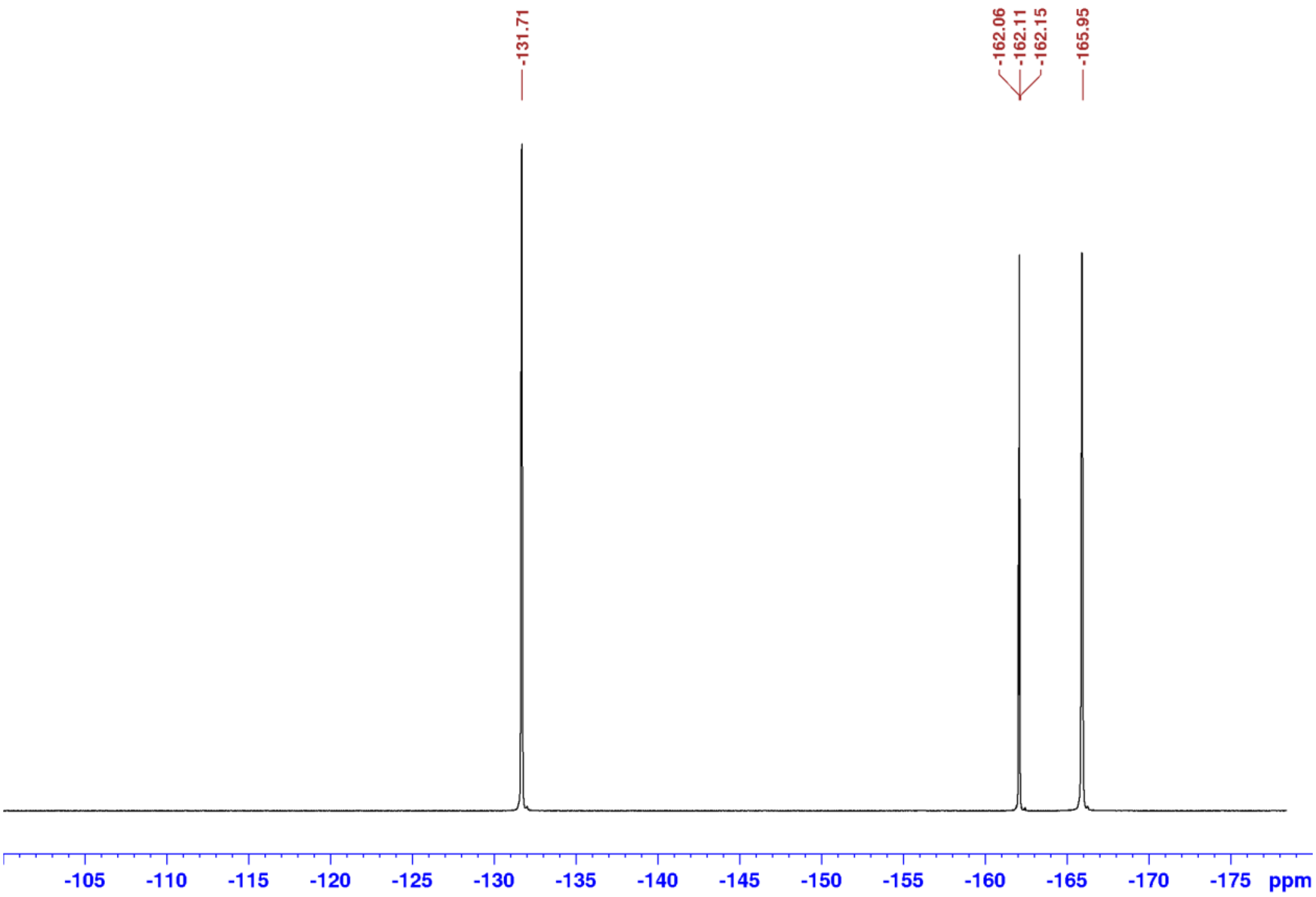

*Figure S29: ^{19}F NMR spectrum of [LTiCH=CBnCH$_2$OAlL][B(C$_6$F$_5$)$_4$] **4b** (generated in situ from equimolar [LTiBn][B(C$_6$F$_5$)$_4$] **2b** and LAlOCH$_2$C≡CH **3**) in C$_6$D$_6$ (300 K).*

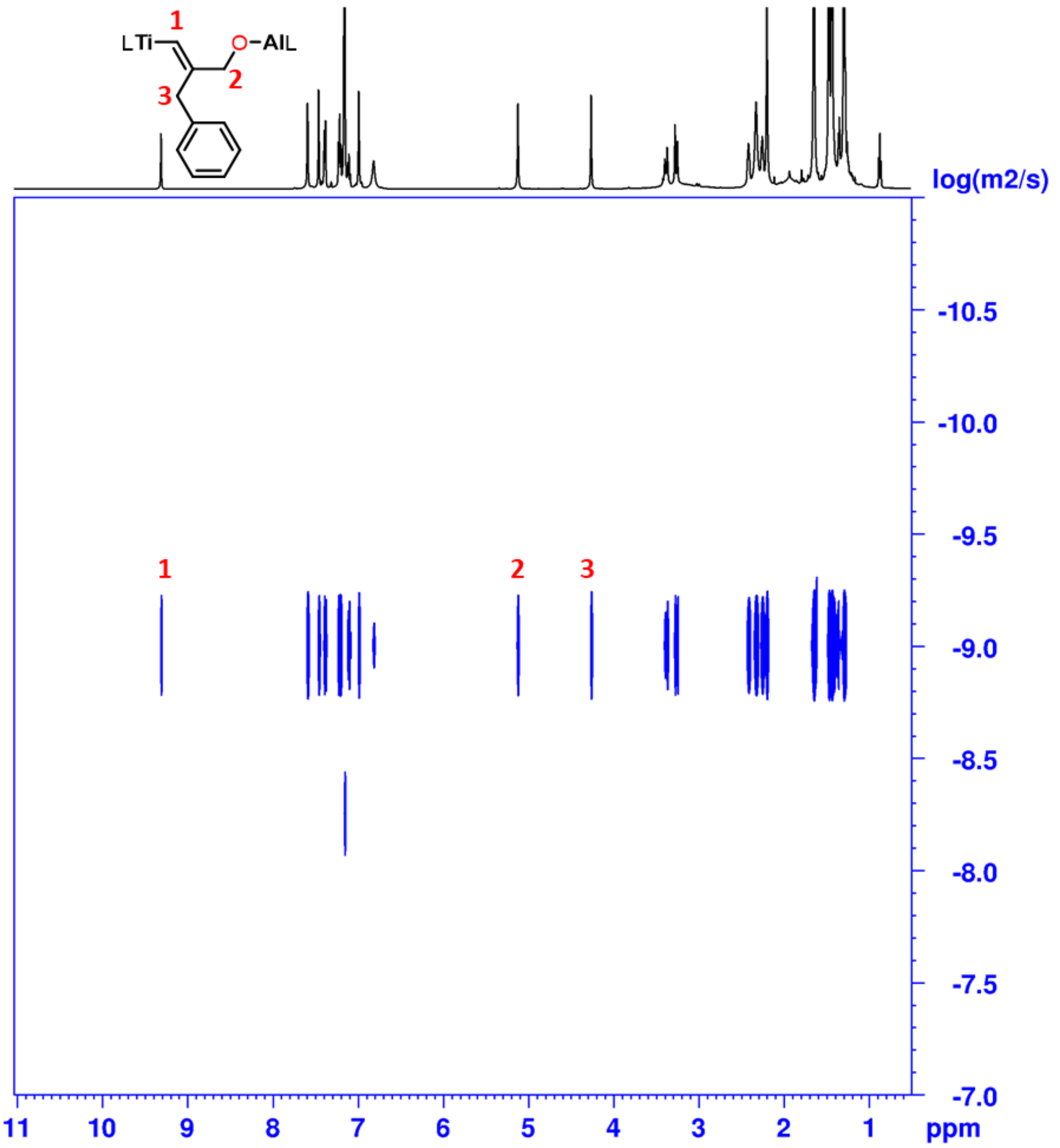

Figure S30: DOSY NMR spectrum of [LTiCH=CBnCH$_2$OAlL][B(C$_6$F$_5$)$_4$] ***4b*** *(generated in situ from equimolar [LTiBn][B(C$_6$F$_5$)$_4$]* ***2b*** *and LAlOCH$_2$C≡CH* ***3****) in C$_6$D$_6$ (300 K).*

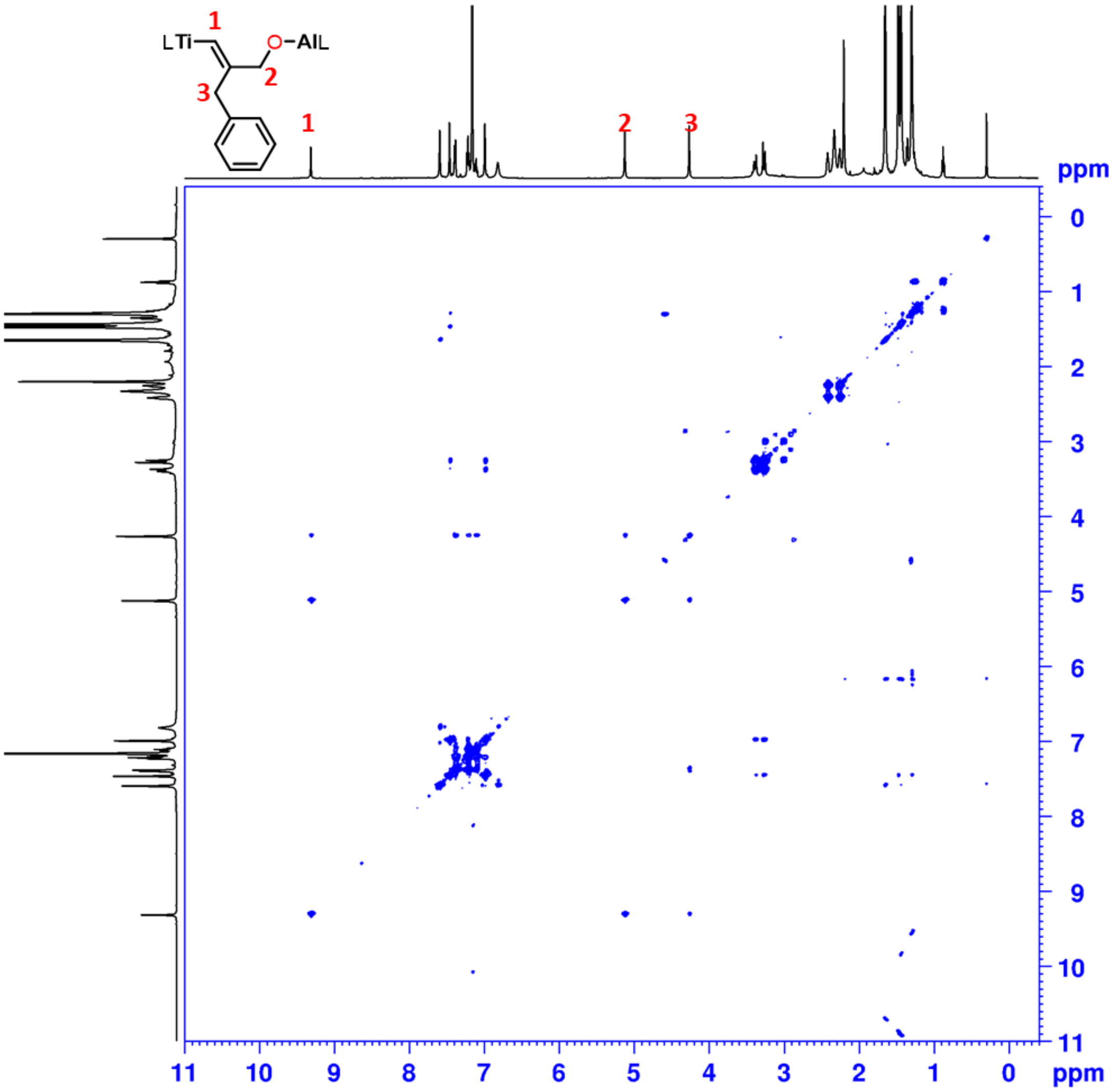

Figure S31: COSY NMR spectrum of [LTiCH=CBnCH$_2$OAlL][B(C_6F_5)$_4$] ***4b*** *(generated in situ from equimolar [LTiBn][B(C_6F_5)$_4$]* ***2b*** *and LAlOCH$_2$C≡CH* ***3****) in C_6D_6 (300 K).*

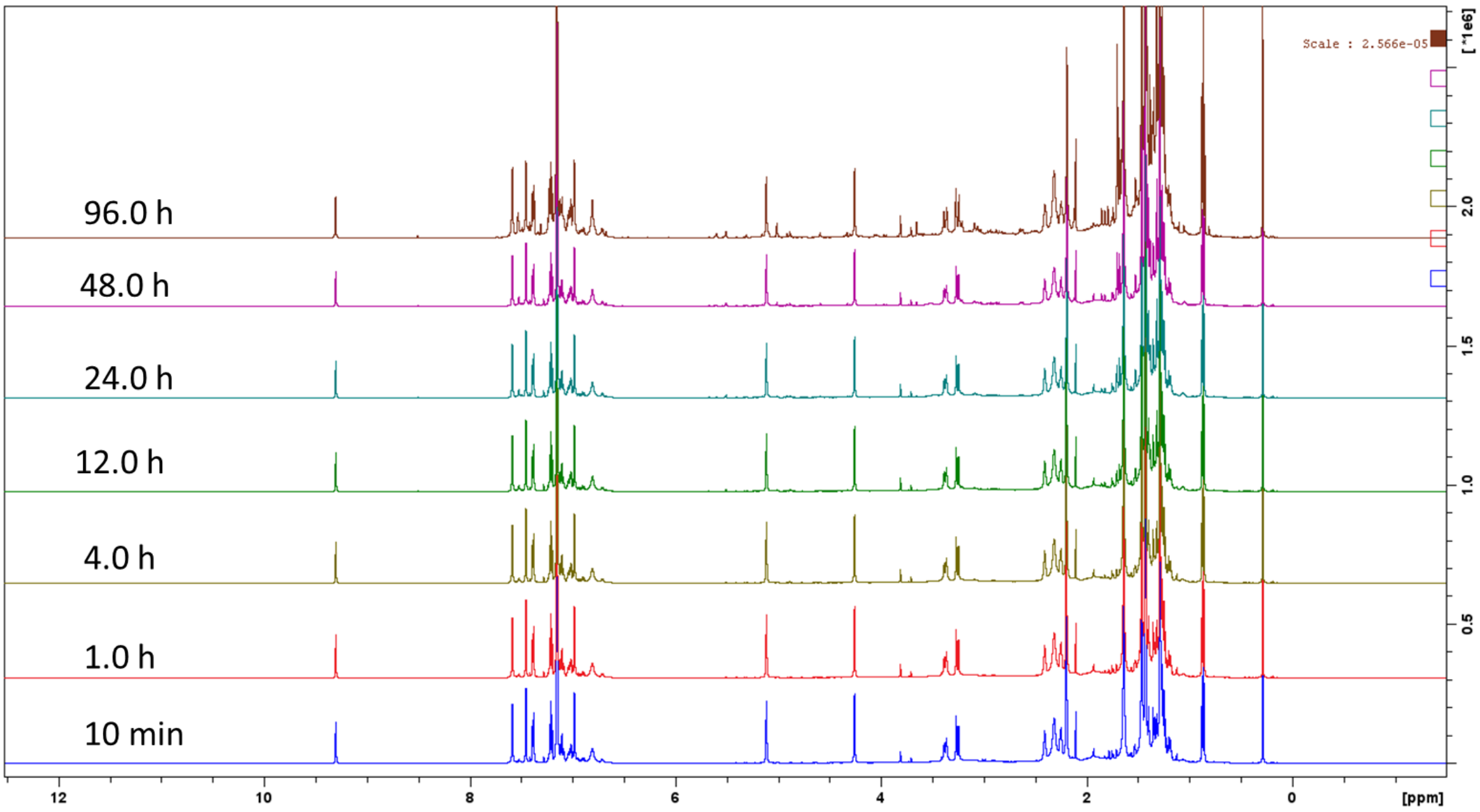

Figure S32: Stacked ¹H NMR spectra of [LTiCH=CBnCH$_2$OAlL][B(C$_6$F$_5$)$_4$] ***4b*** *(generated in situ from equimolar [LTiBn][B(C$_6$F$_5$)$_4$]* ***2b*** *and LAlOCH$_2$C≡CH* ***3****) in C$_6$D$_6$ (300 K) recorded over a period of 4 days.*

Polymer characterisation

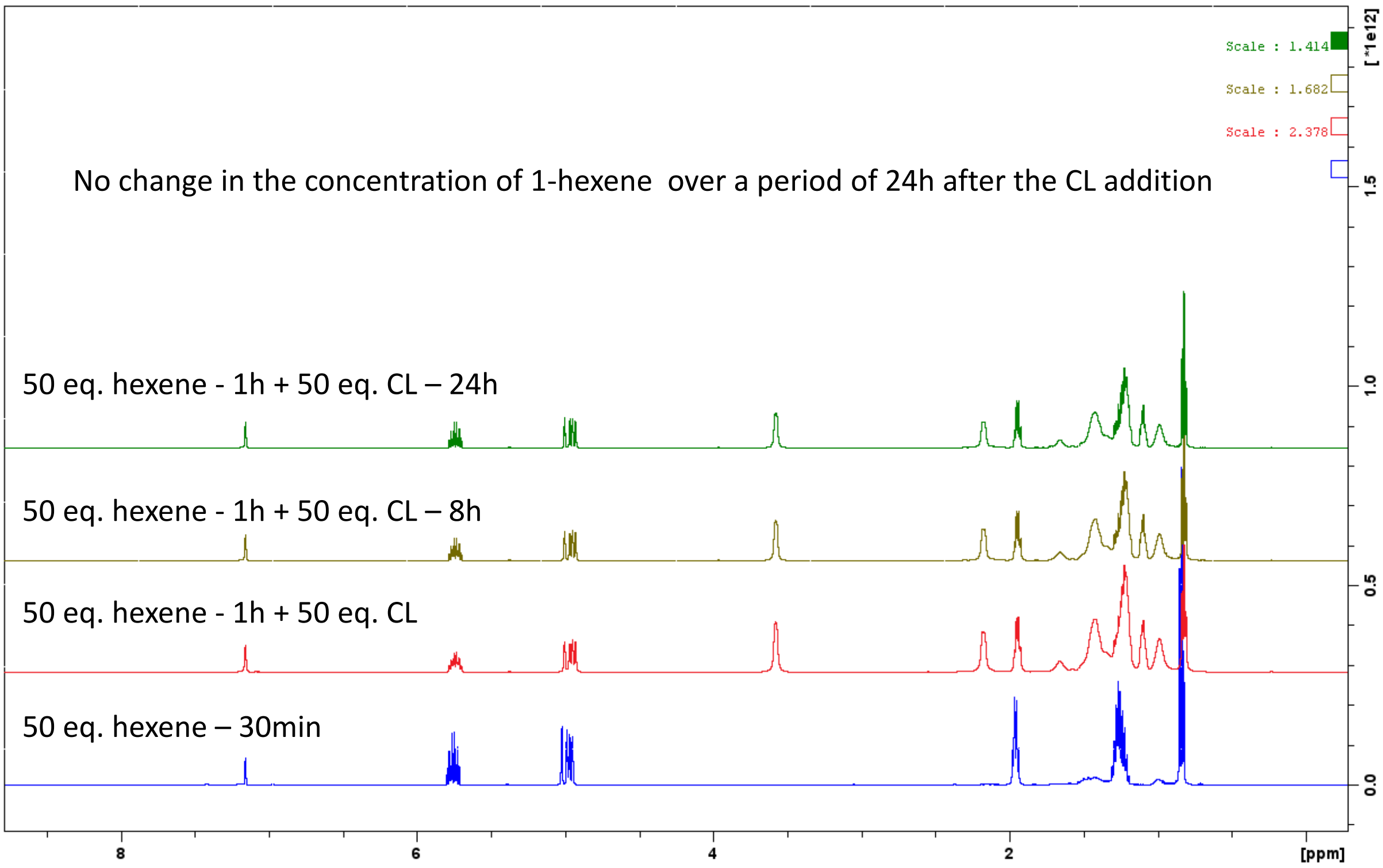

*Figure S33: Stacked ^{1}H NMR spectra of attempted block copolymerisation with [LTiBn][B(C_6F_5)$_4$] **2b** via sequential monomer addition of 1-hexene followed by CL in C_6D_6 (300 K) recorded over a period of 24 hours. No change in the concentration of 1-hexene concentration upon CL addition suggesting the arrest of 1-hexene polymerisation.*

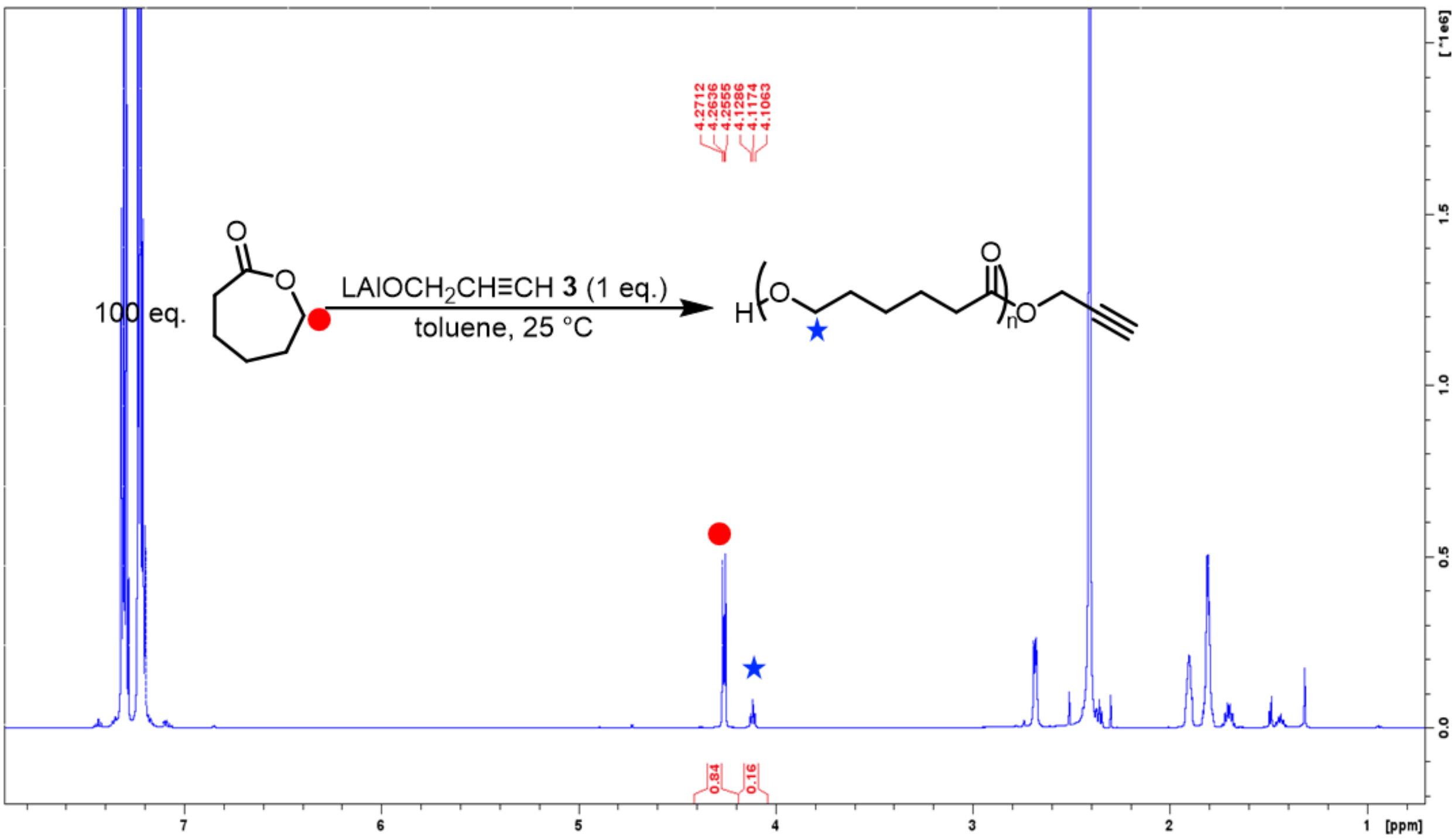

*Figure S34: An example NMR spectrum to show the monomer (CL) conversion to polymer (PCL) by LAlOCH$_2$C≡CH **3** (Table S1, entry 1) in $CDCl_3$ (300 K).*

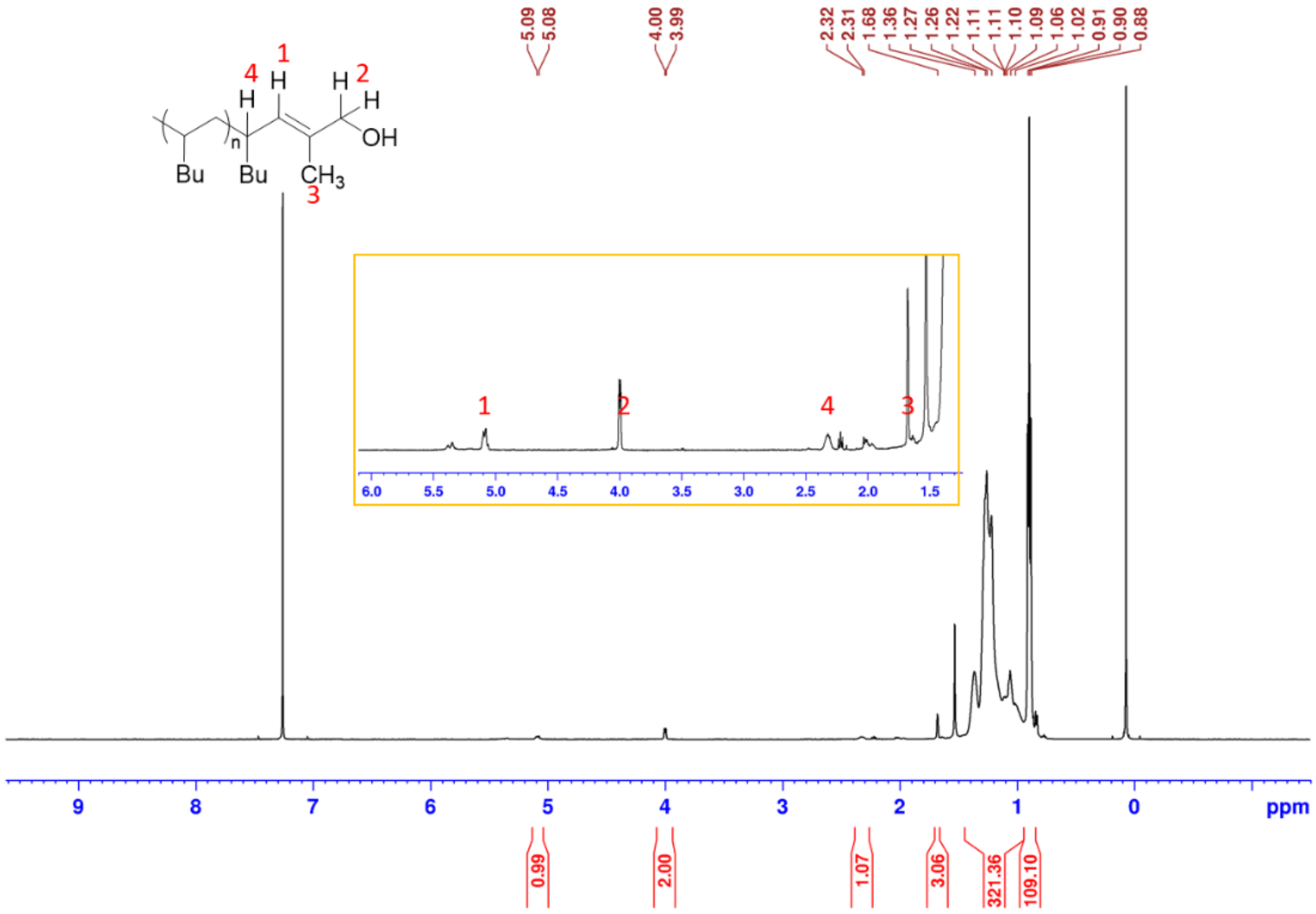

*Figure S35: ^{1}H NMR spectrum of PH produced from in situ generated [LTiCH=C(Me)CH$_2$OAlL][MeB(C$_6$F$_5$)$_3$] **4a**, recorded in CDCl$_3$ (300 K). 2-methylpropenol end groups are observed.*

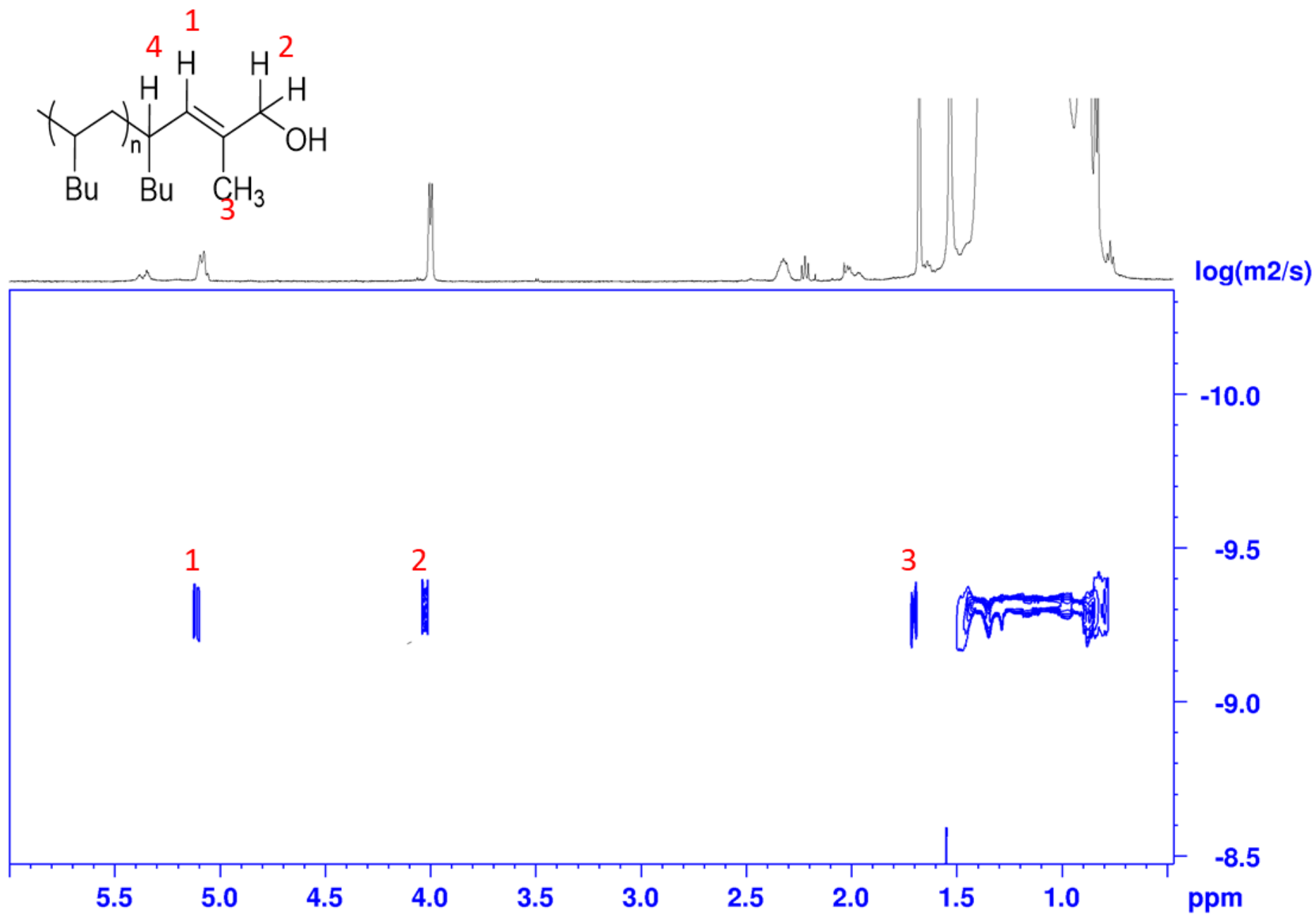

*Figure S36: DOSY NMR spectrum of PH produced from in situ generated [LTiCH=C(Me)CH$_2$OAlL][MeB(C$_6$F$_5$)$_3$] **4a**, with 2-methylpropenol end group in CDCl$_3$ (300 K). Diagnostic resonances for the 2-methylpropenol end groups were observed at the same diffusion coefficient as that of PH.*

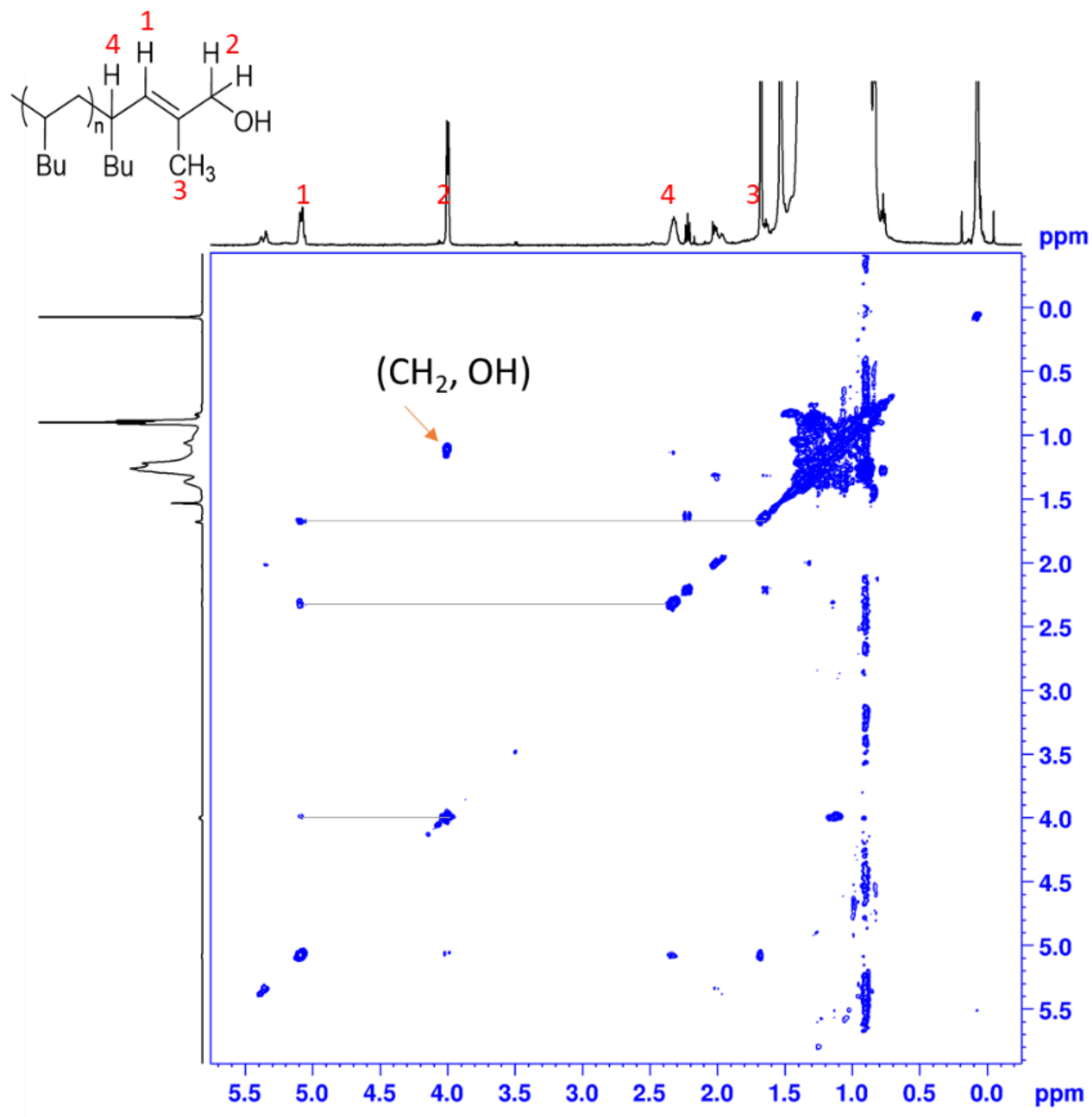

Figure S37: COSY NMR spectrum of PH produced from in situ generated [LTiCH=C(Me)CH$_2$OAlL][MeB(C$_6$F$_5$)$_3$] ***4a****, with 2-methylpropenol end group in CDCl$_3$ (300 K). Diagnostic cross peaks for the 2-methylpropenol end group including (CH$_2$, OH) were observed.*

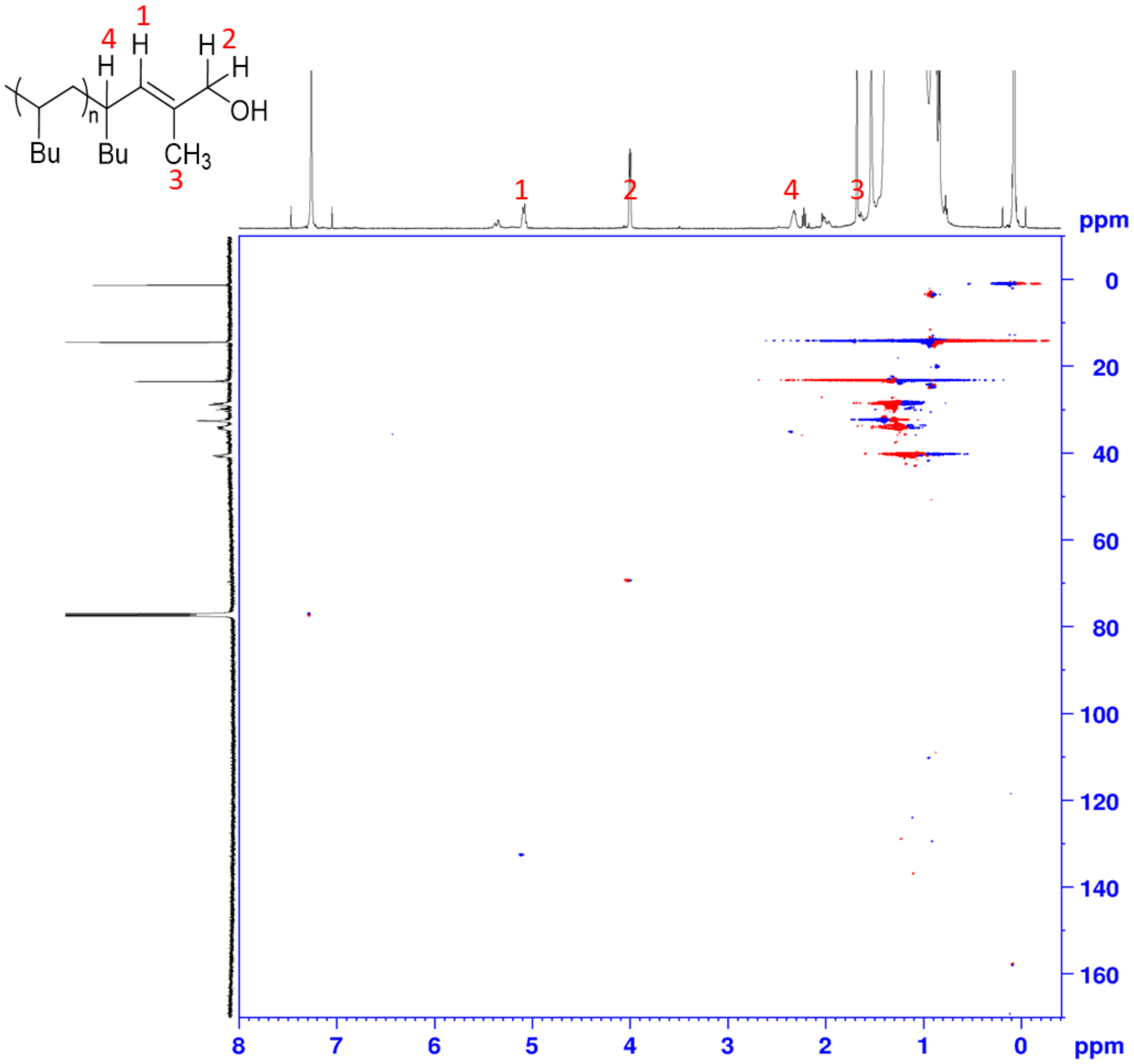

Figure S38: HSQC NMR spectrum of PH produced from in situ generated [LTiCH=C(Me)CH_2OAlL][MeB(C_6F_5)$_3$] ***4a****, with 2-methylpropenol end group in* $CDCl_3$ *(300 K).*

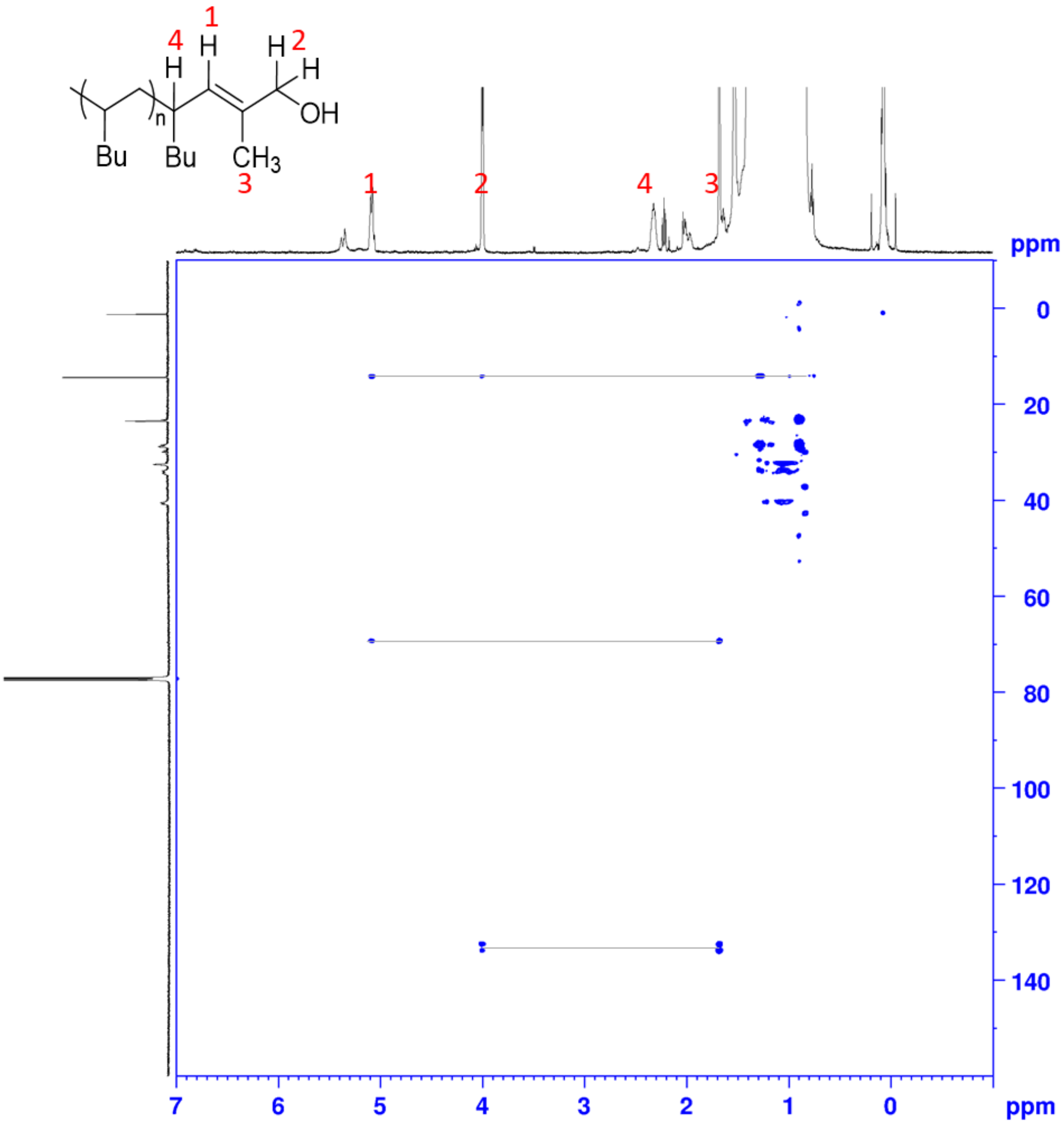

*Figure S39: HMBC NMR spectrum of PH produced from in situ generated [LTiCH=C(Me)CH$_2$OAlL][MeB(C$_6$F$_5$)$_3$] **4a**, with 2-methylpropenol end group in CDCl$_3$ (300 K).*

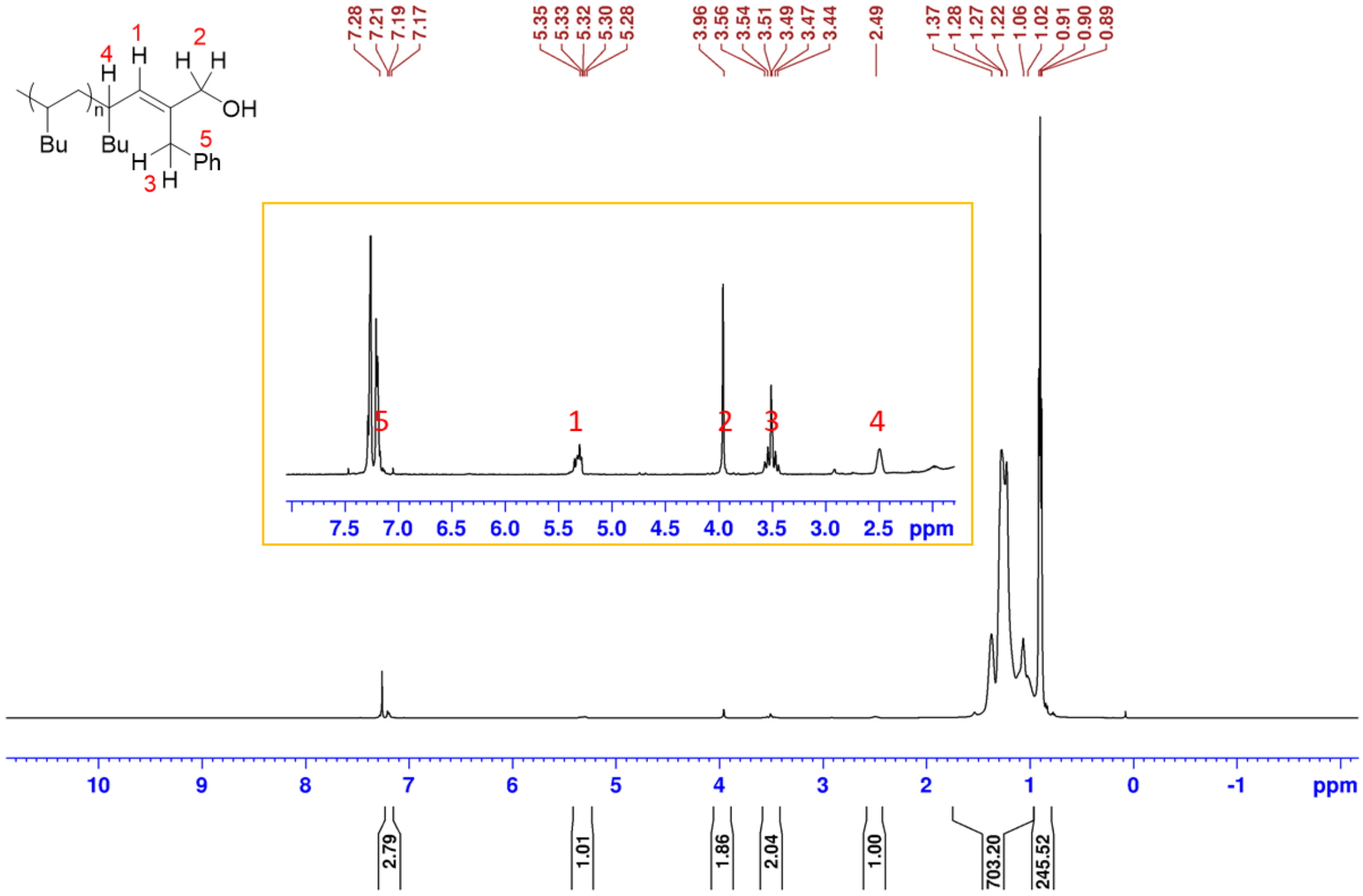

*Figure S40: ¹H NMR spectrum of PH produced from in situ generated [LTiCH=C(Bn)CH$_2$OAlL][B(C$_6$F$_5$)$_4$] **4b**, recorded in CDCl$_3$ (300 K). 2-benzylpropenol end groups are observed.*

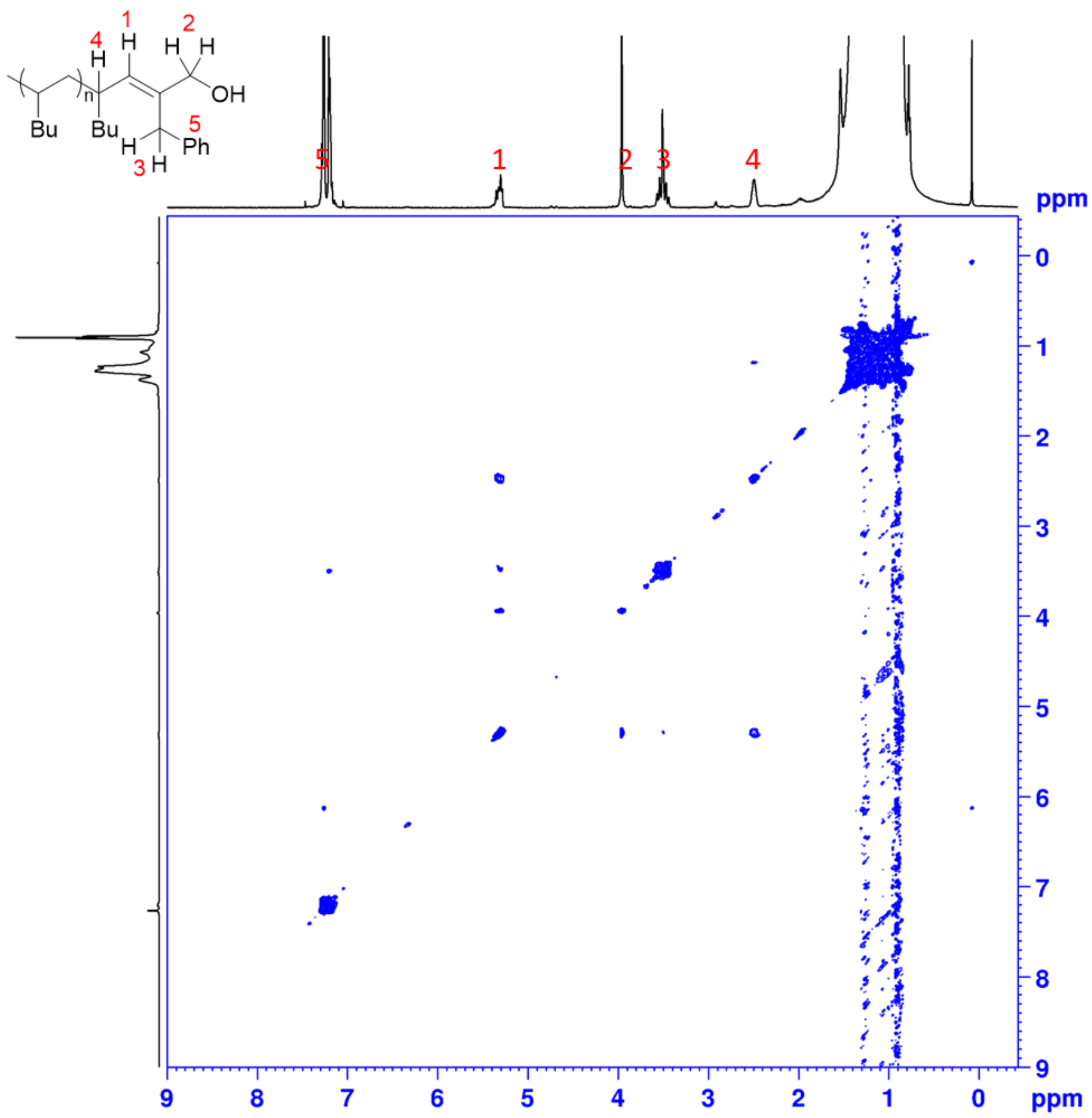

Figure S41: COSY NMR spectrum of PH produced from in situ generated [LTiCH=C(Bn)CH$_2$OAlL][B(C$_6$F$_5$)$_4$] ***4b****, with 2-benzylpropenol end group in CDCl$_3$ (300 K). Diagnostic cross peaks for the 2-benzylpropenol end group were observed.*

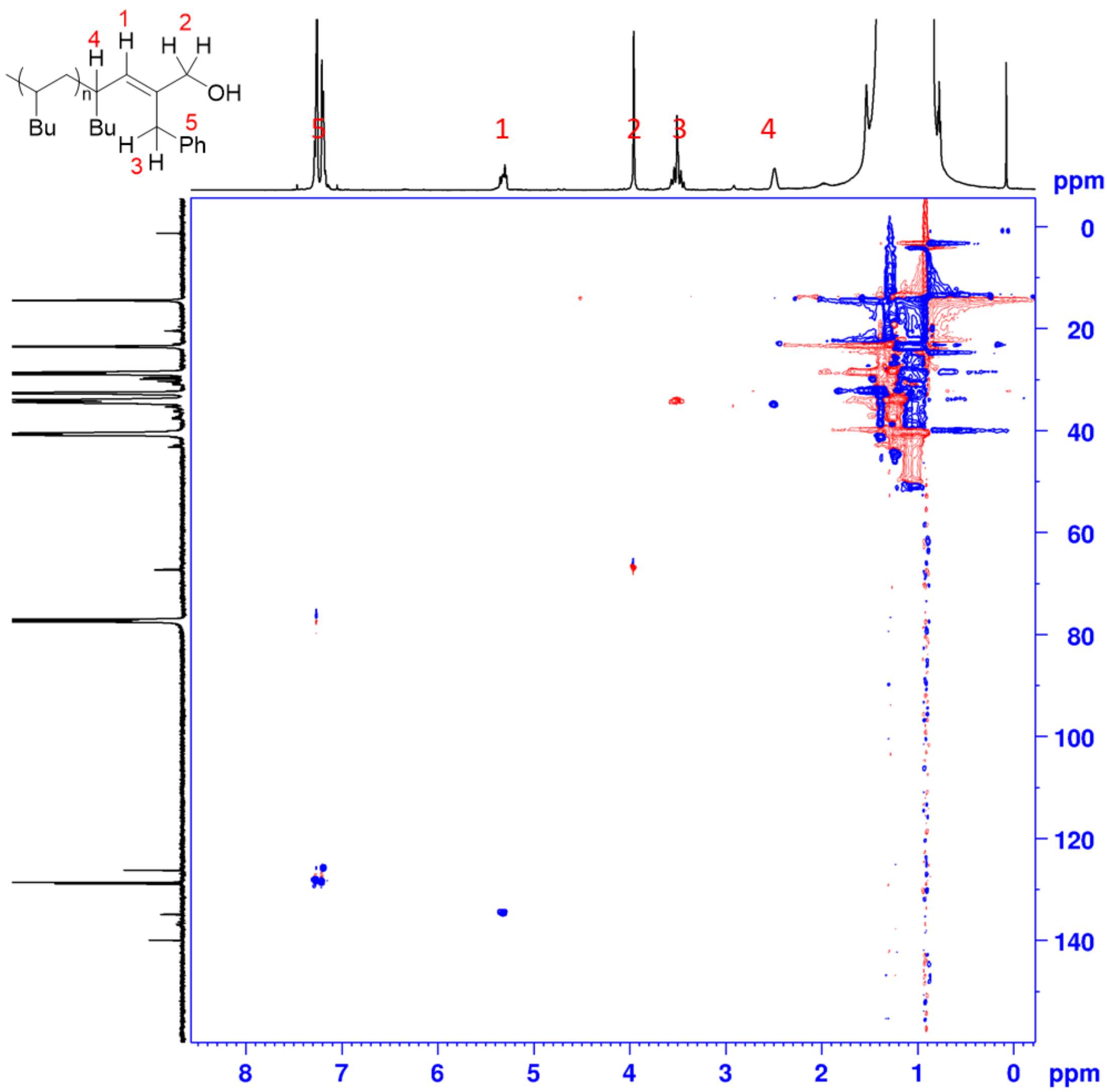

*Figure S42: HSQC NMR spectrum of PH produced from in situ generated [LTiCH=C(Bn)CH$_2$OAlL][B(C$_6$F$_5$)$_4$] **4b**, with 2-benzylpropenol end group in CDCl$_3$ (300 K).*

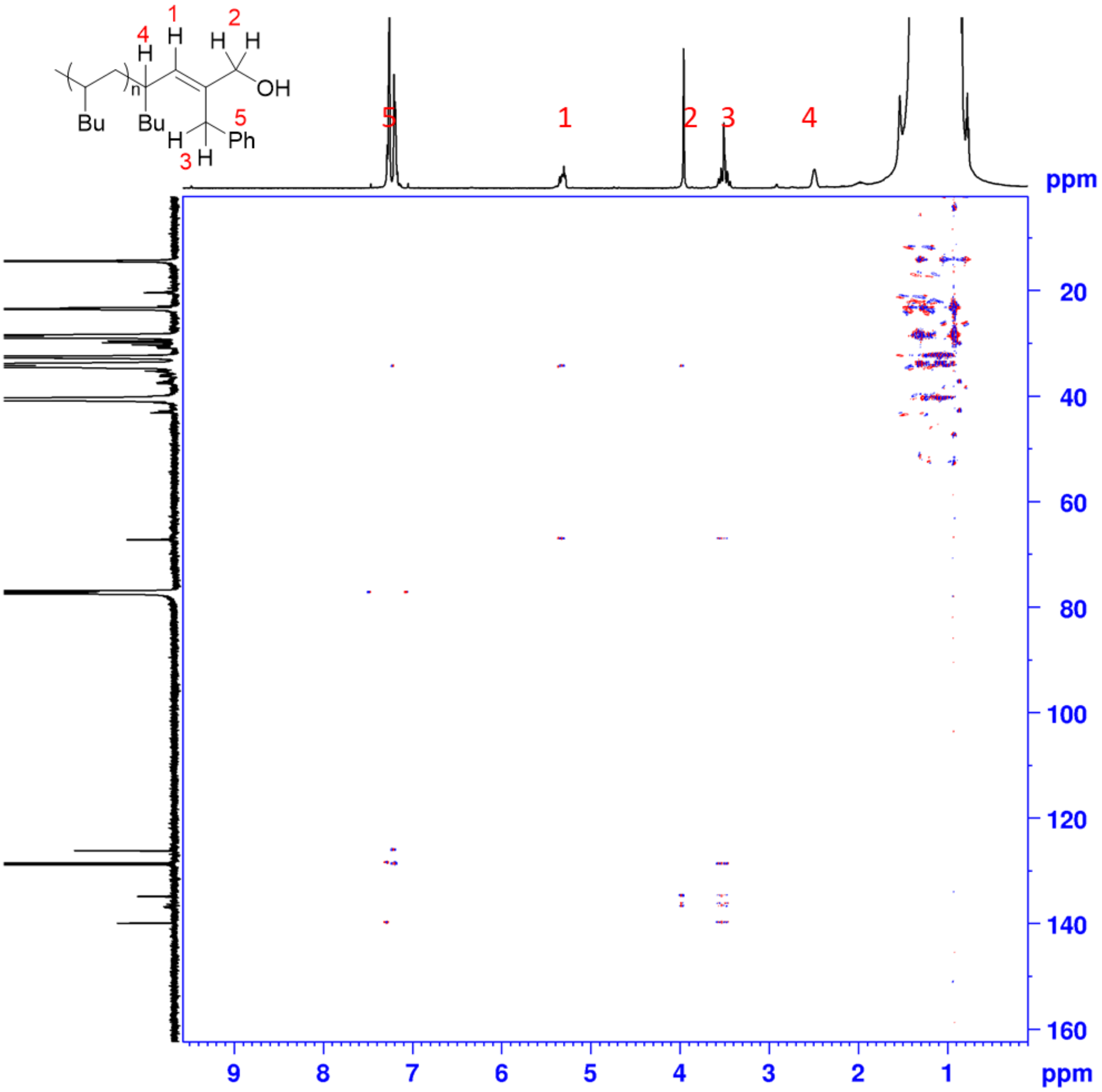

Figure S43: HMBC NMR spectrum of PH produced from in situ generated [LTiCH=C(Bn)CH$_2$OAlL][B(C$_6$F$_5$)$_4$] ***4b****, with 2-benzylpropenol end group in CDCl$_3$ (300 K).*

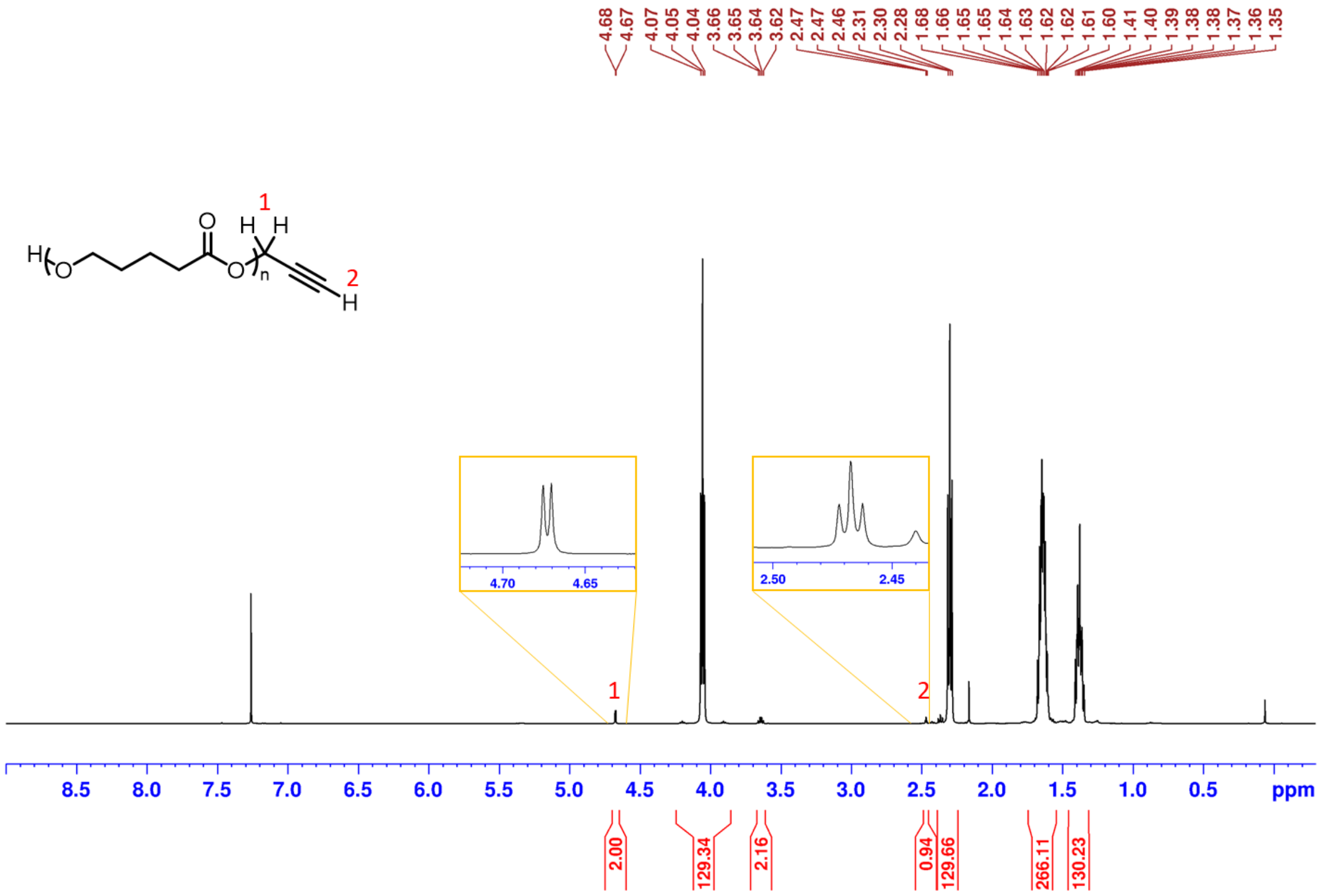

*Figure S44: ^{1}H NMR spectrum of PCL generated with LAlOCH$_2$C≡CH **3** in CDCl$_3$ (300 K), including the diagnostic alkyne C≡CH (δ 2.47 ppm) and OCH$_2$ (δ 4.68 ppm) resonances of the propynoxy group.*

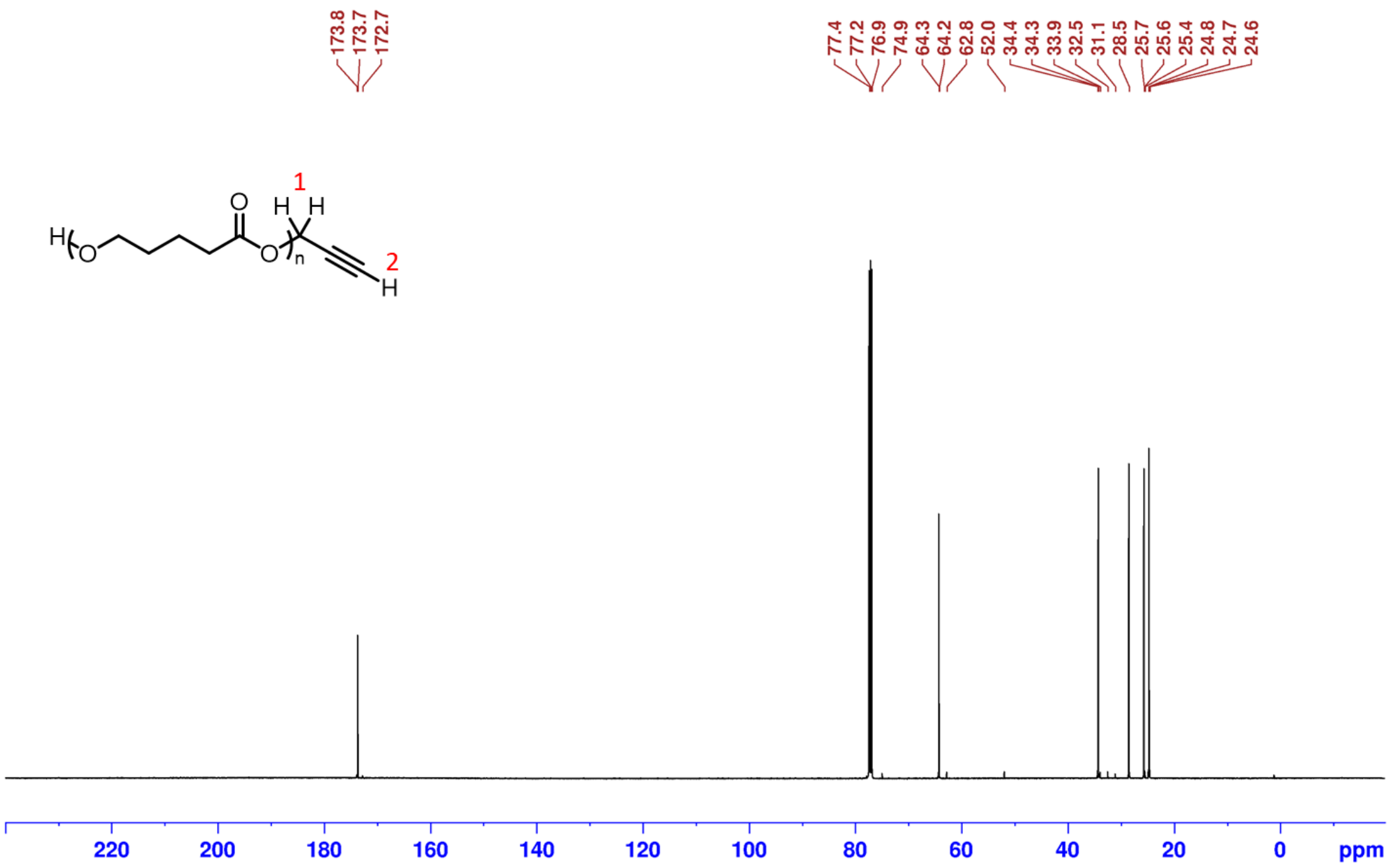

*Figure S45: ^{13}C NMR spectrum of PCL generated with LAlOCH$_2$C≡CH **3** with propynoxy end groups in CDCl$_3$ (300 K).*

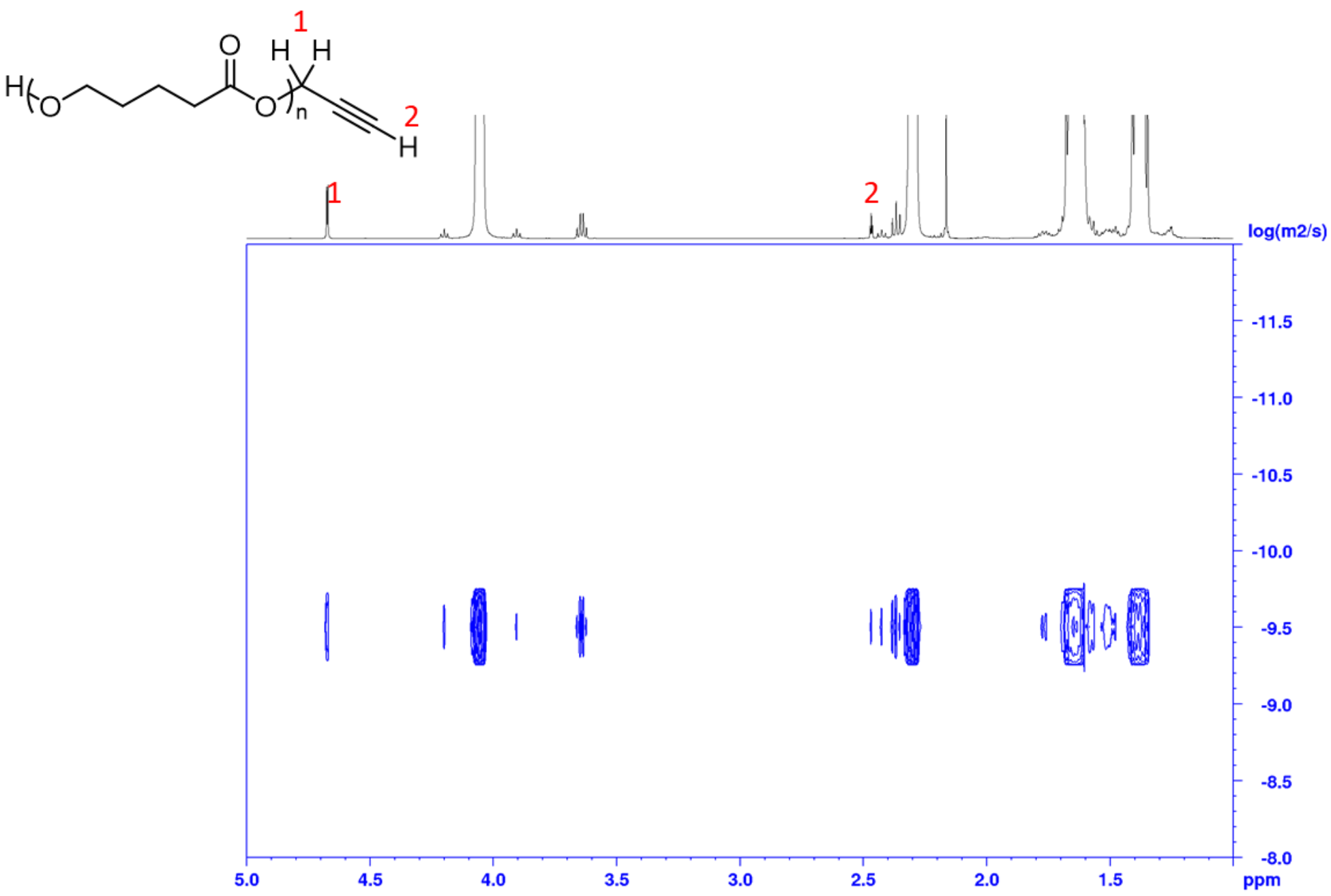

*Figure S46: DOSY NMR spectrum of PCL generated with LAlOCH$_2$C≡CH **3**, with propynoxy end groups in CDCl$_3$ (300 K). Diagnostic resonances for the with propynoxy end groups were observed at the same diffusion coefficient as that of PCL.*

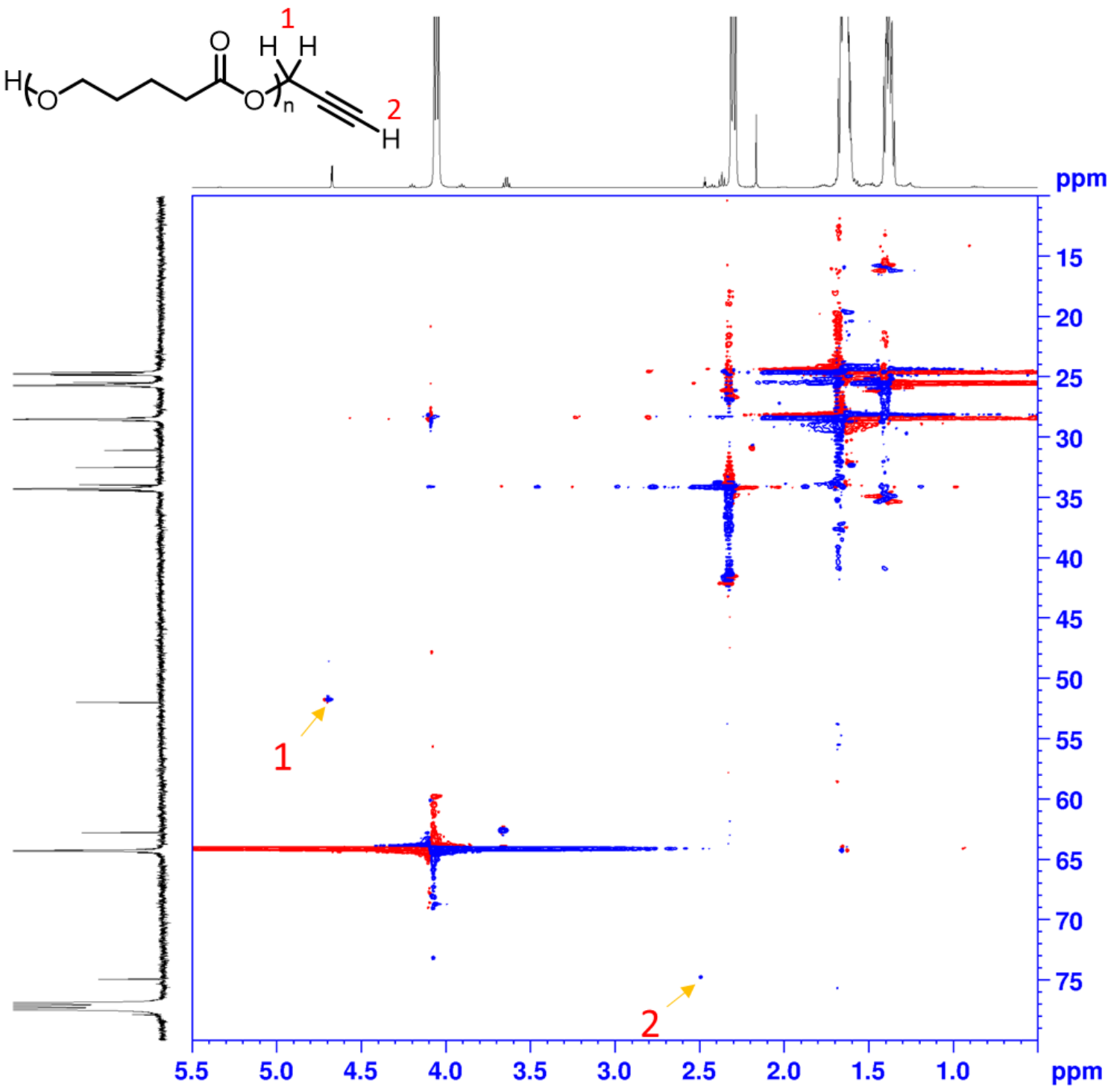

*Figure S47: HSQC NMR spectrum of PCL generated with LAlOCH$_2$C≡CH **3**, with propynoxy end groups in CDCl$_3$ (300 K).*

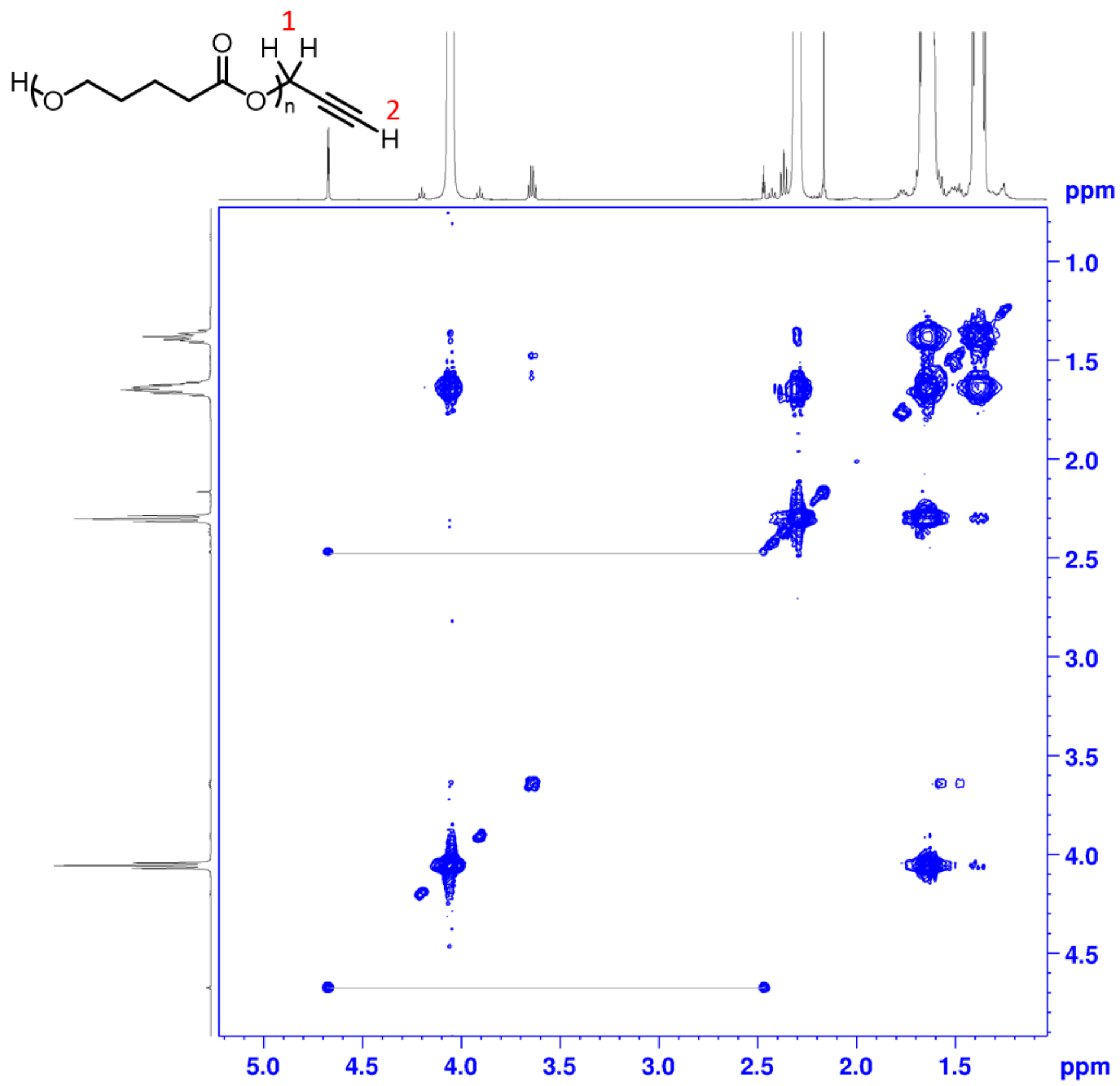

*Figure S48: COSY NMR spectrum of PCL generated with LAlOCH$_2$C≡CH **3**, with propynoxy end groups in $CDCl_3$ (300 K).*

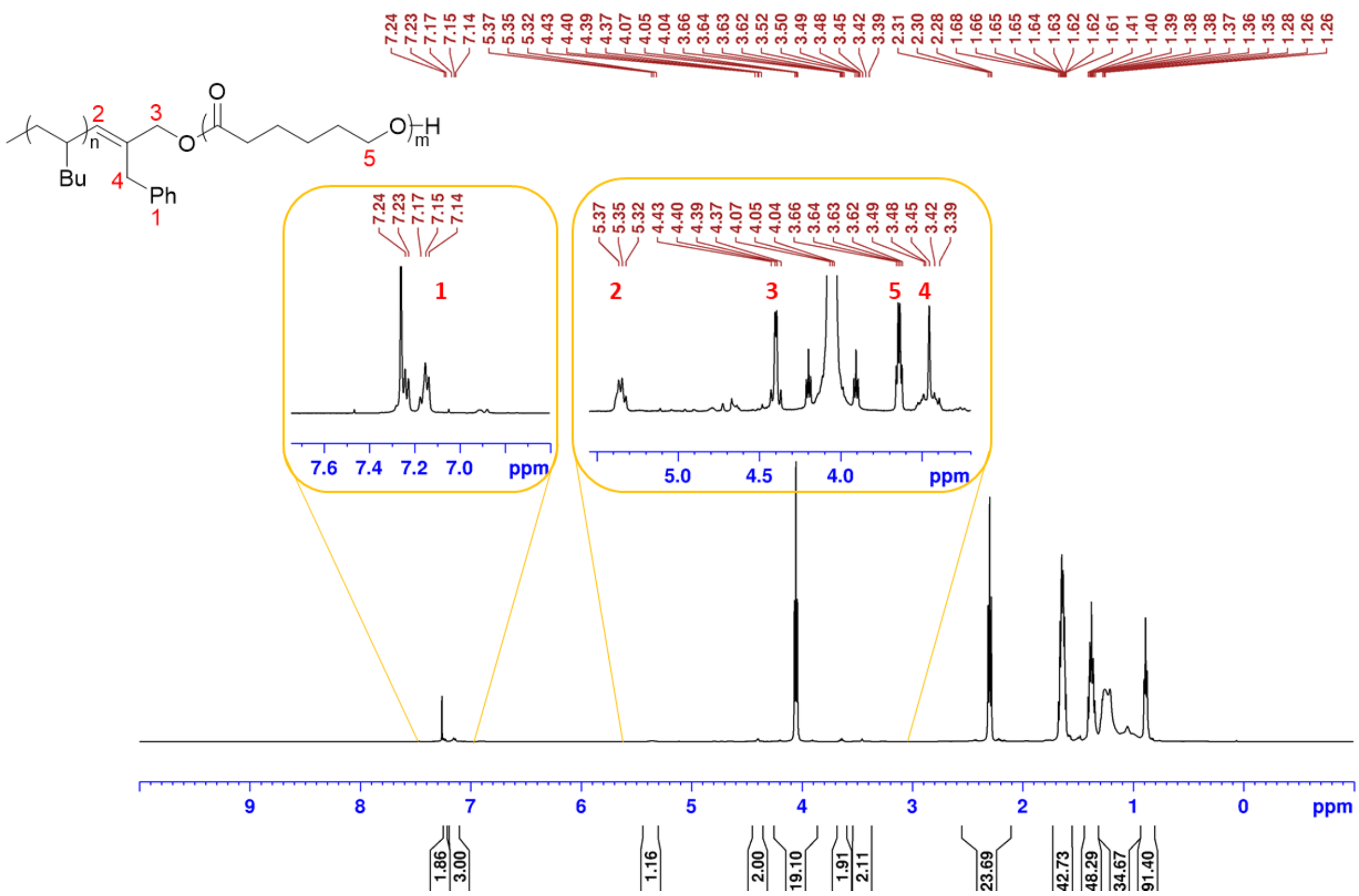

*Figure S49: ^{1}H NMR spectrum of PH-b-PCL generated with [LTiCH=C(Bn)CH$_2$OAlL][B(C$_6$F$_5$)$_4$] **4b**, recorded in $CDCl_3$ (300 K).*

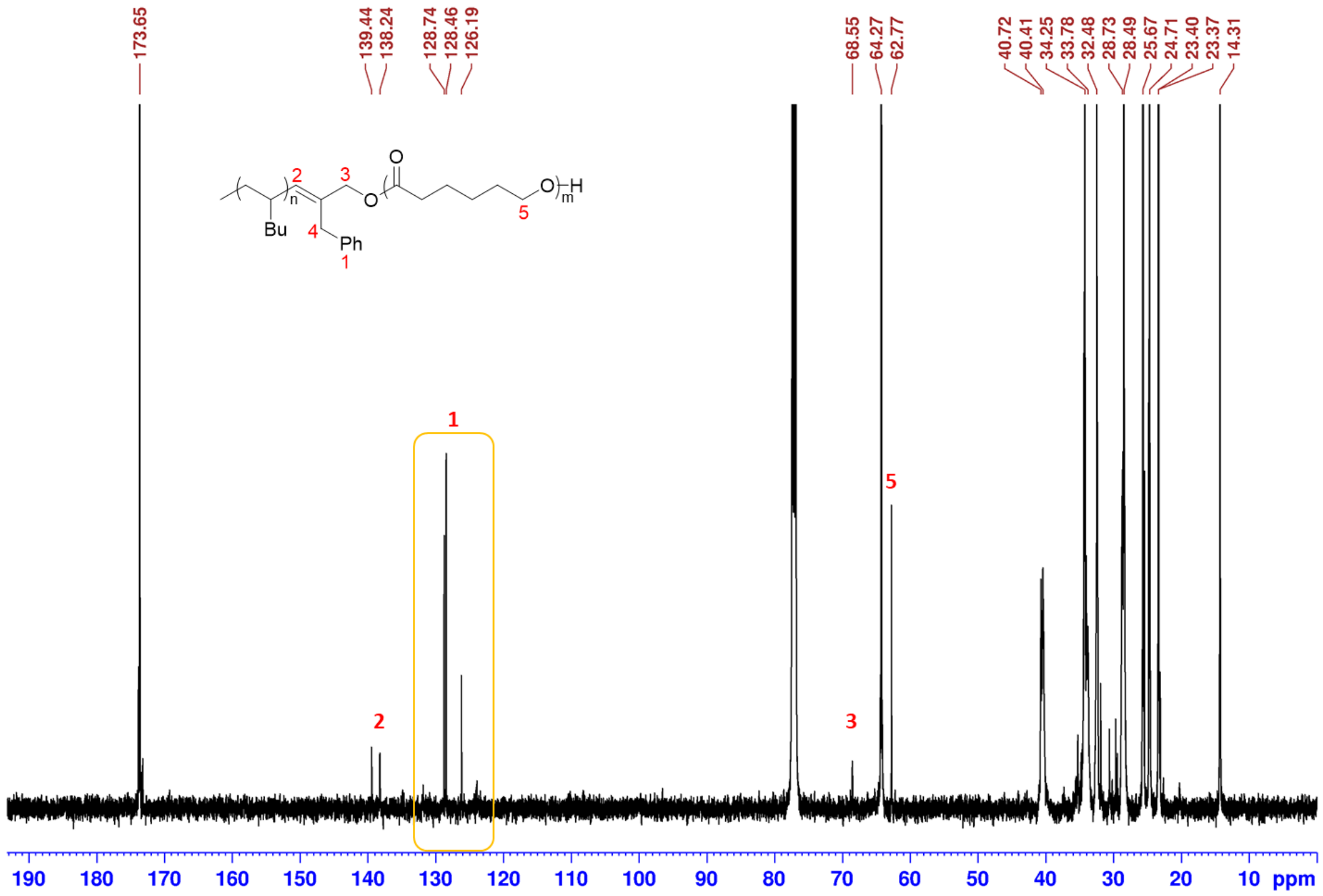

*Figure S50: ^{13}C NMR spectrum of PH-b-PCL produced from in situ generated [LTiCH=C(Bn)CH_2OAlL][B(C_6F_5)$_4$] **4b**, recorded in $CDCl_3$ (300 K).*

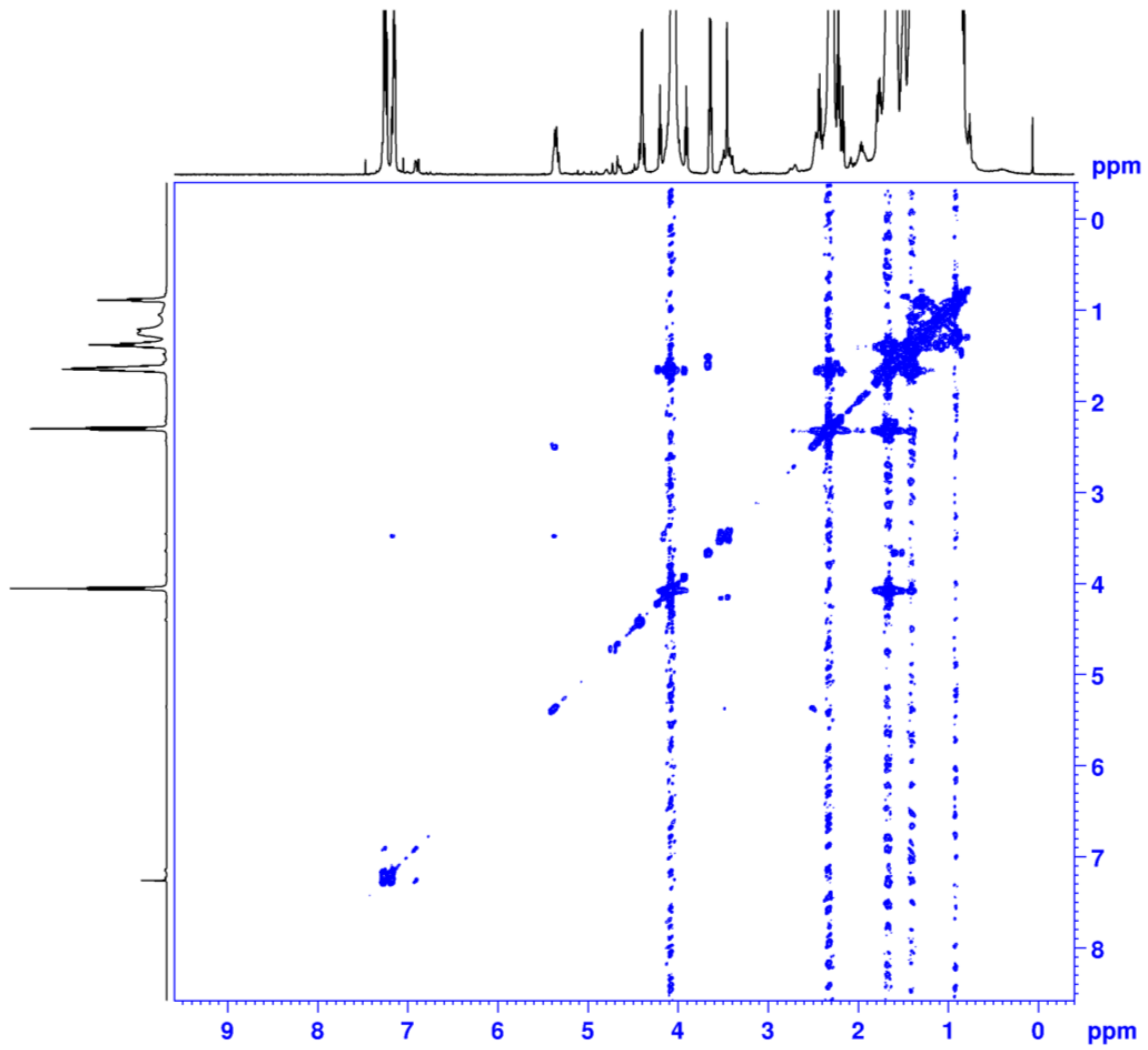

*Figure S51: COSY NMR spectrum of PH-b-PCL produced from in situ generated [LTiCH=C(Bn)CH$_2$OAlL][B(C$_6$F$_5$)$_4$] **4b**, recorded in CDCl$_3$ (300 K).*

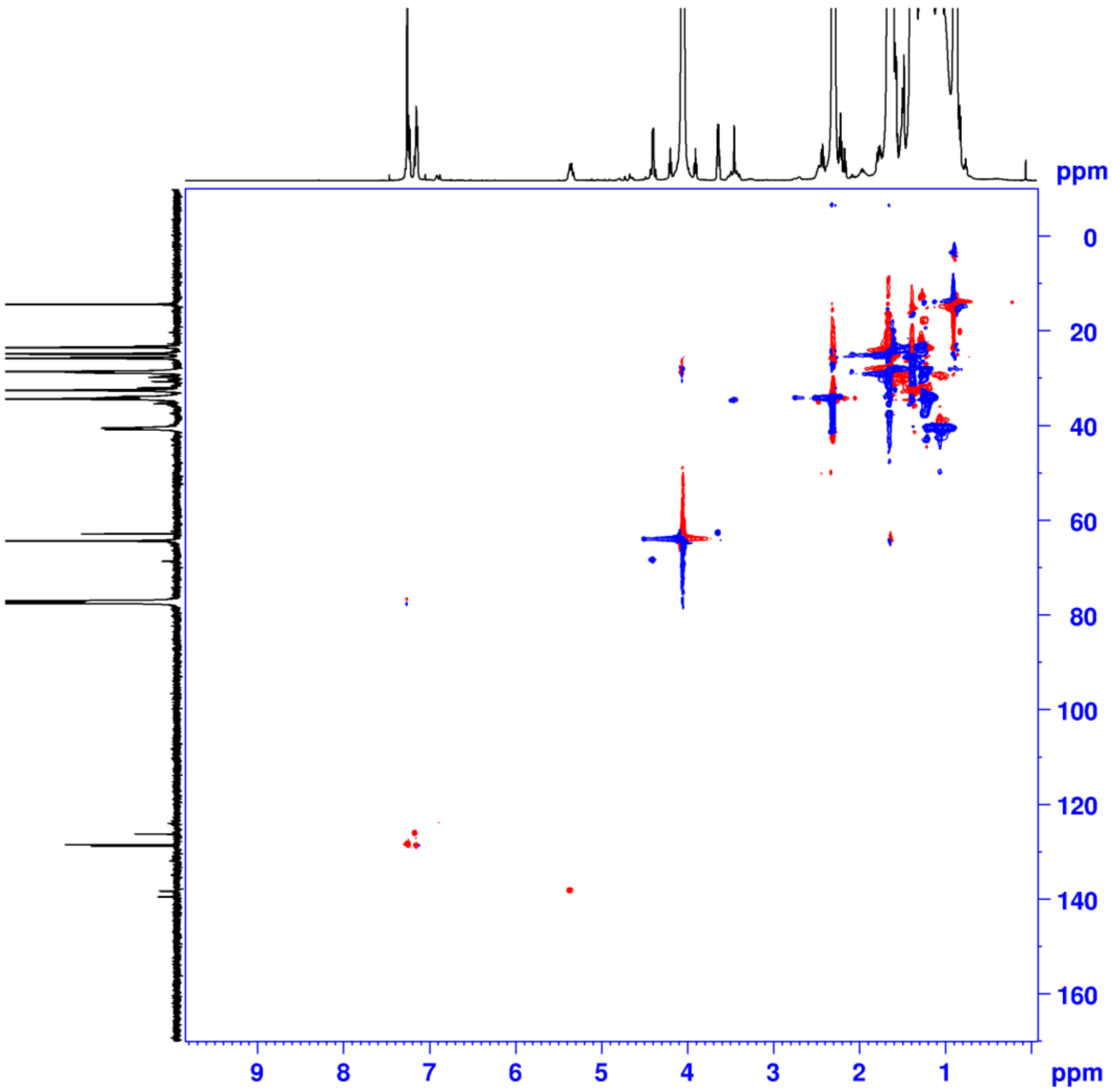

*Figure S52: HSQC NMR spectrum of PH-b-PCL produced from in situ generated [LTiCH=C(Bn)CH$_2$OAlL][B(C$_6$F$_5$)$_4$] **4b**, recorded in CDCl$_3$ (300 K).*

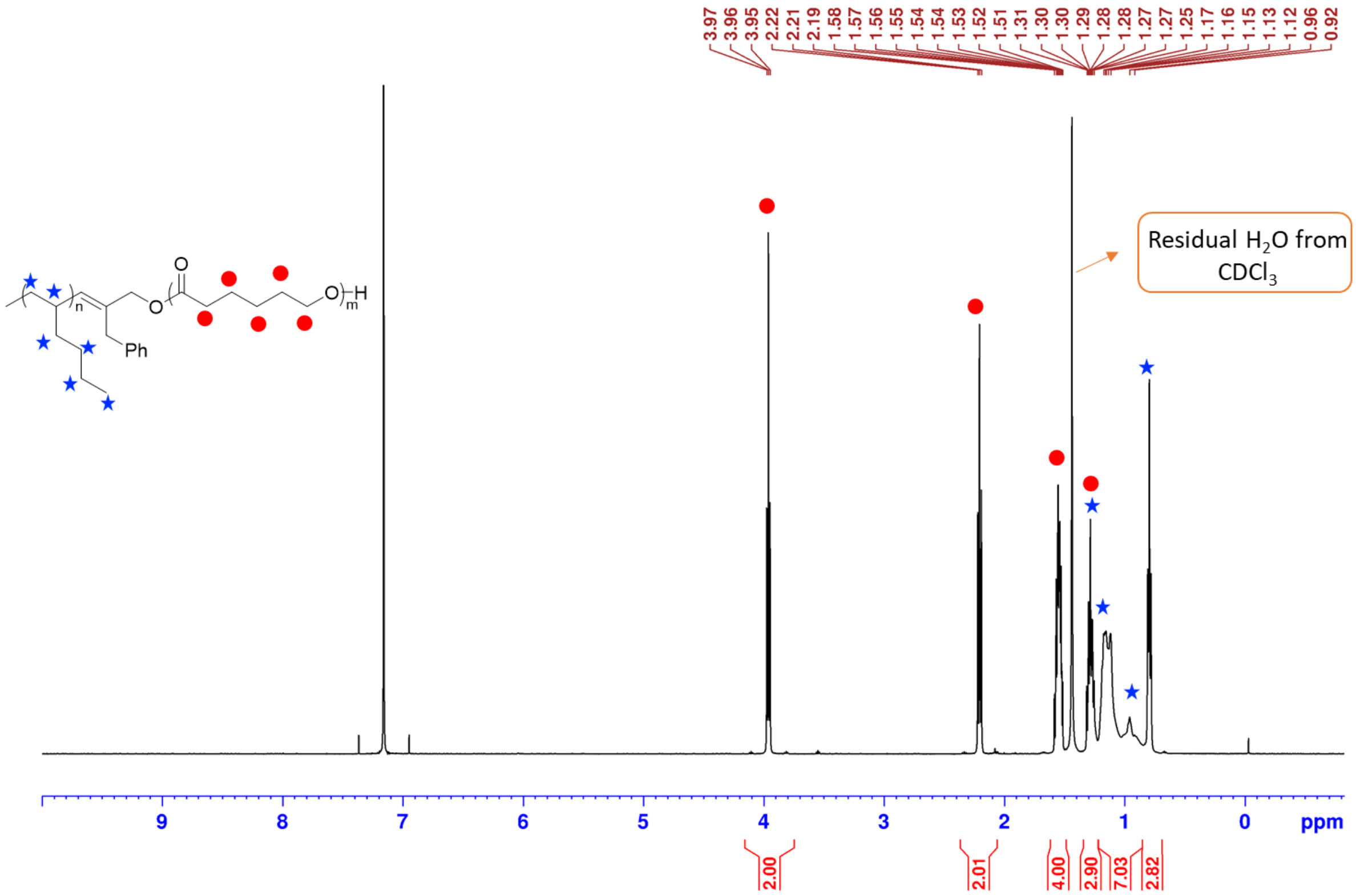

*Figure S53: ^{1}H NMR spectrum of PH-b-PCL generated with [LTiCH=C(Bn)CH$_2$OAlL][B(C$_6$F$_5$)$_4$] **4b** (Table 1, entry 13), recorded in CDCl$_3$ (300 K).*

$$Mol\%_{PCL} = \frac{\frac{I_{PCL}}{2}}{\frac{I_{PCL}}{2} + \frac{I_{PH}}{3}} \times 100$$

I_{PCL} = ^{1}H NMR integration value of the methylene group next to the ester oxygen (-CH$_2$O-, 3.96 ppm in CDCl$_3$) of PCL block

I_{PH} = ^{1}H NMR integration value of the methyl group at the end of side chain (-CH$_3$, 0.96 ppm in CDCl$_3$) of PH block

Figure S54: Formula used for the calculation of comonomer content in PH-b-PCL block copolymers based on ^{1}H NMR chemical shifts recorded in CDCl$_3$. The same formula is used for the calculation of comonomer content in the PO-b-PCL and PD-b-PCL block copolymers.

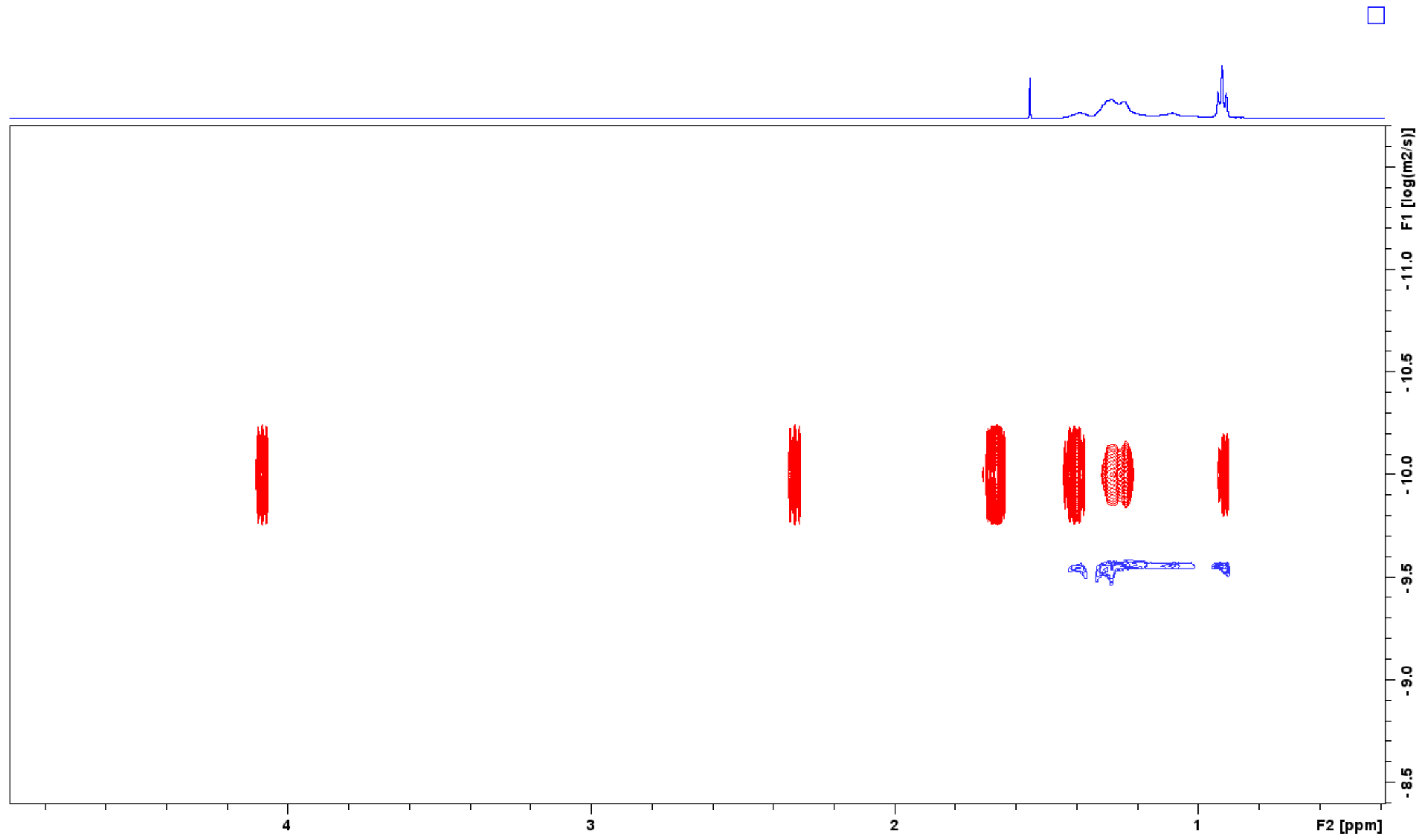

*Figure S55: Stacked DOSY spectra of PH homopolymer (blue) and PH-b-PCL copolymer (red) produced from in situ generated [LTiCH=C(Me)CH_2OAlL][MeB$(C_6F_5)_3$] **4a** (Table 1, entry 4 and 10).*

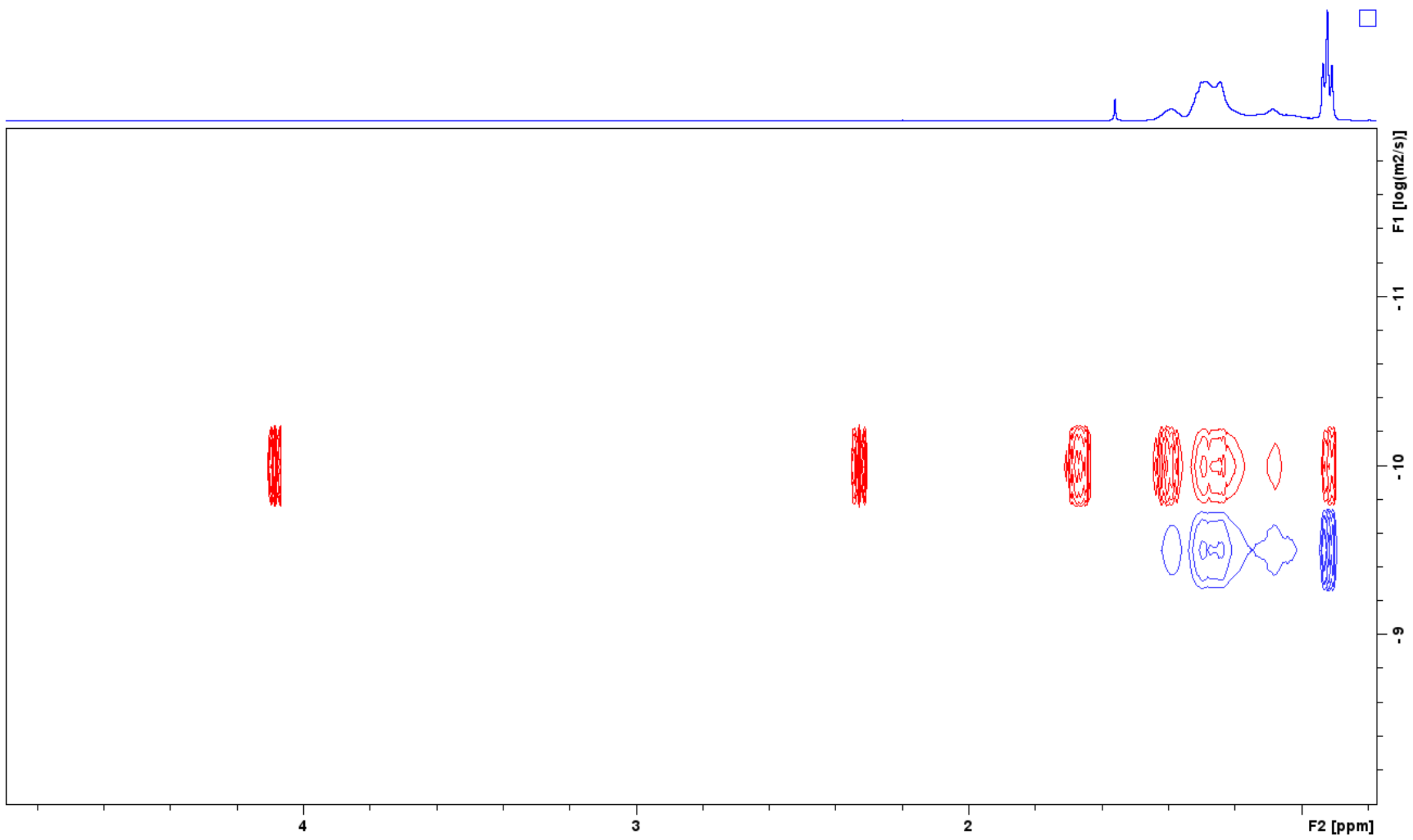

*Figure S56: Stacked DOSY spectra of PH homopolymer (blue) and PH-b-PCL copolymer (red) produced from in situ generated [LTiCH=C(Me)CH_2OAlL][MeB$(C_6F_5)_3$] **4a** (Table 1, entry 5 and 11).*

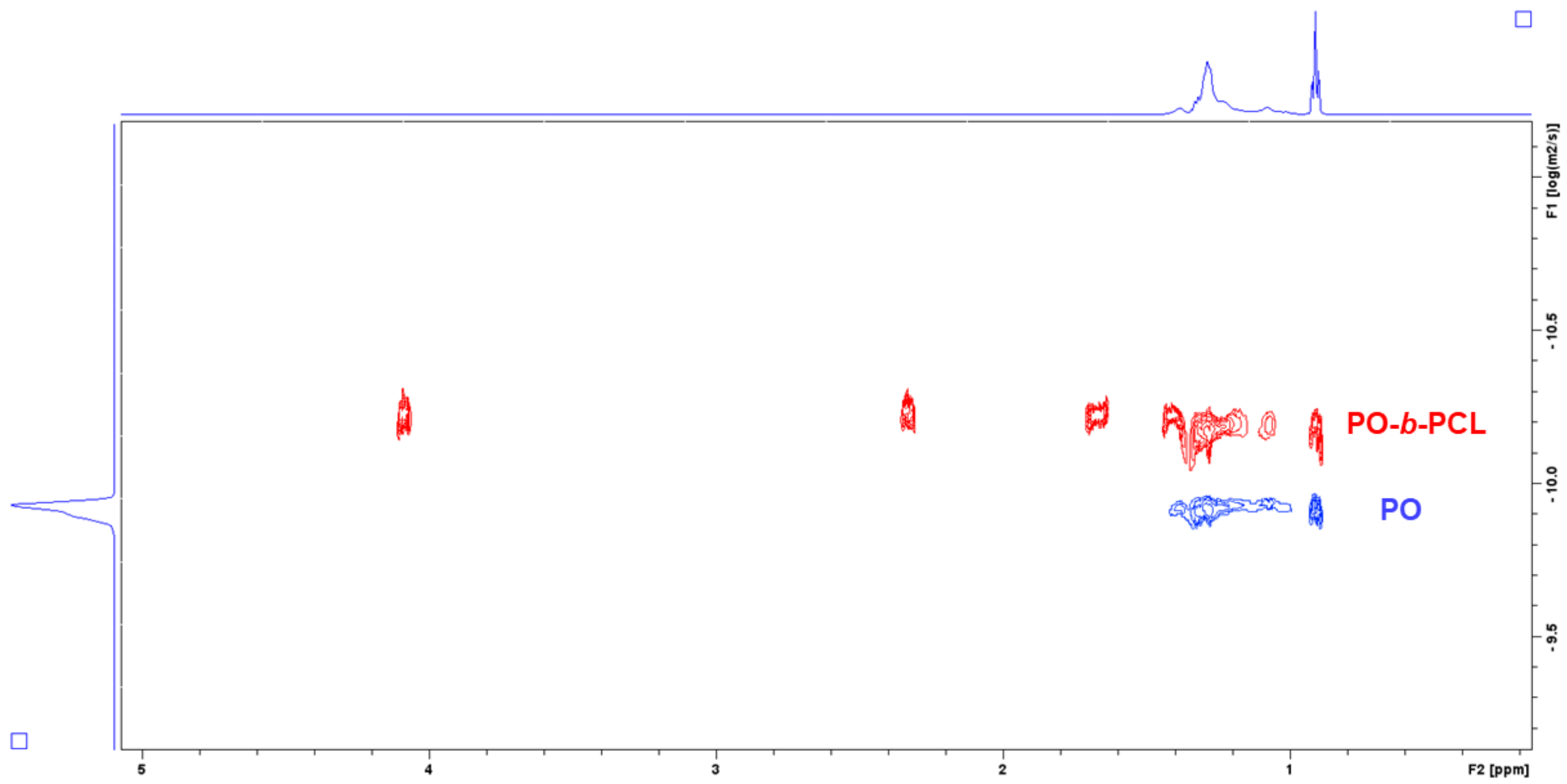

Figure S57: Stacked DOSY spectra of PO homopolymer (blue) and PO-b-PCL copolymer (red) produced from in situ generated [LTiCH=C(Bn)CH_2OAlL][B(C_6F_5)$_4$] ***4b*** *(Table 1, entry 15 and 16).*

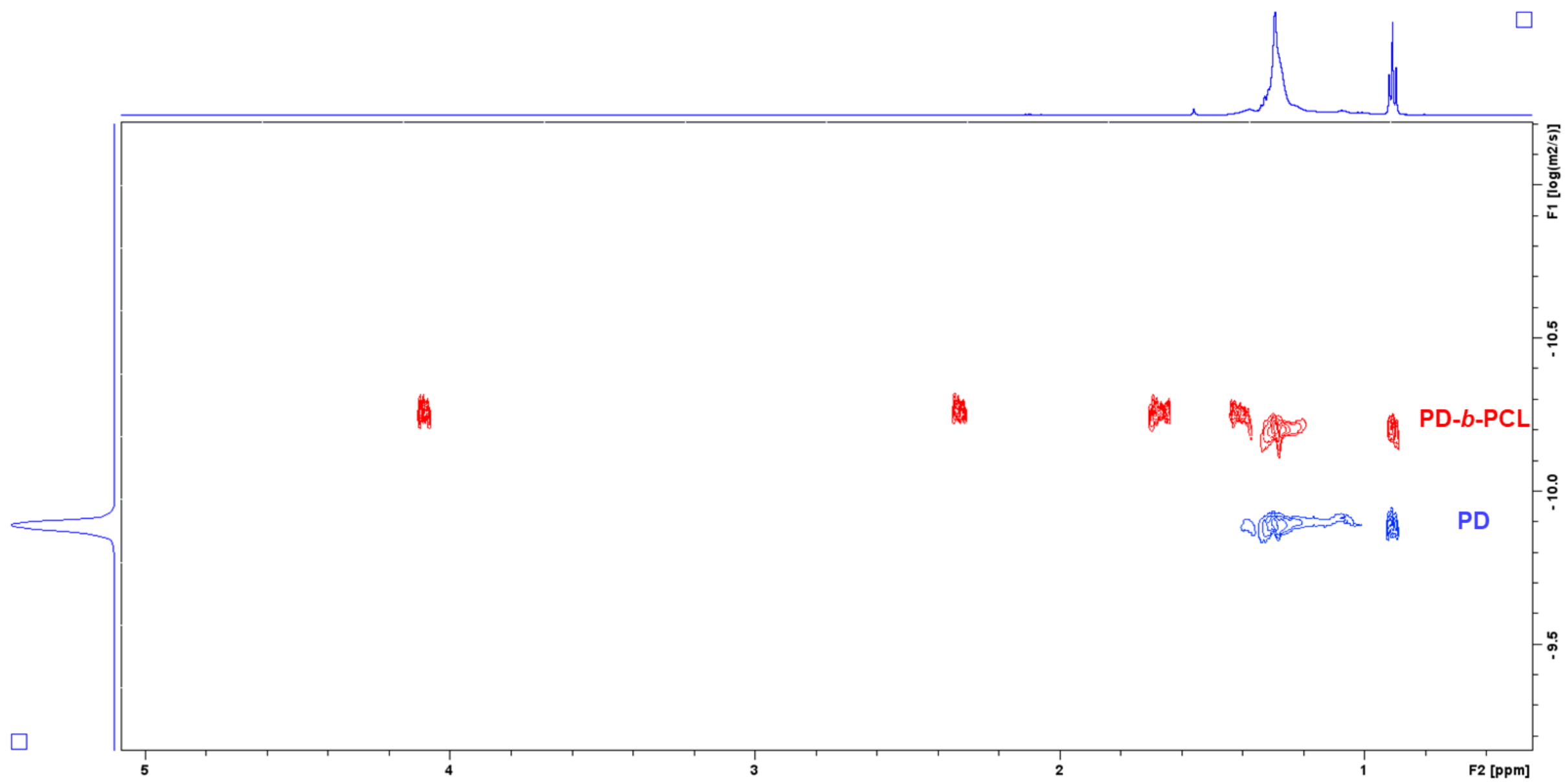

Figure S58: Stacked DOSY spectra of PD homopolymer (blue) and PD-b-PCL copolymer (red) produced from in situ generated [LTiCH=C(Bn)CH_2OAlL][B(C_6F_5)$_4$] ***4b*** *(Table 1, entry 17 and 18).*

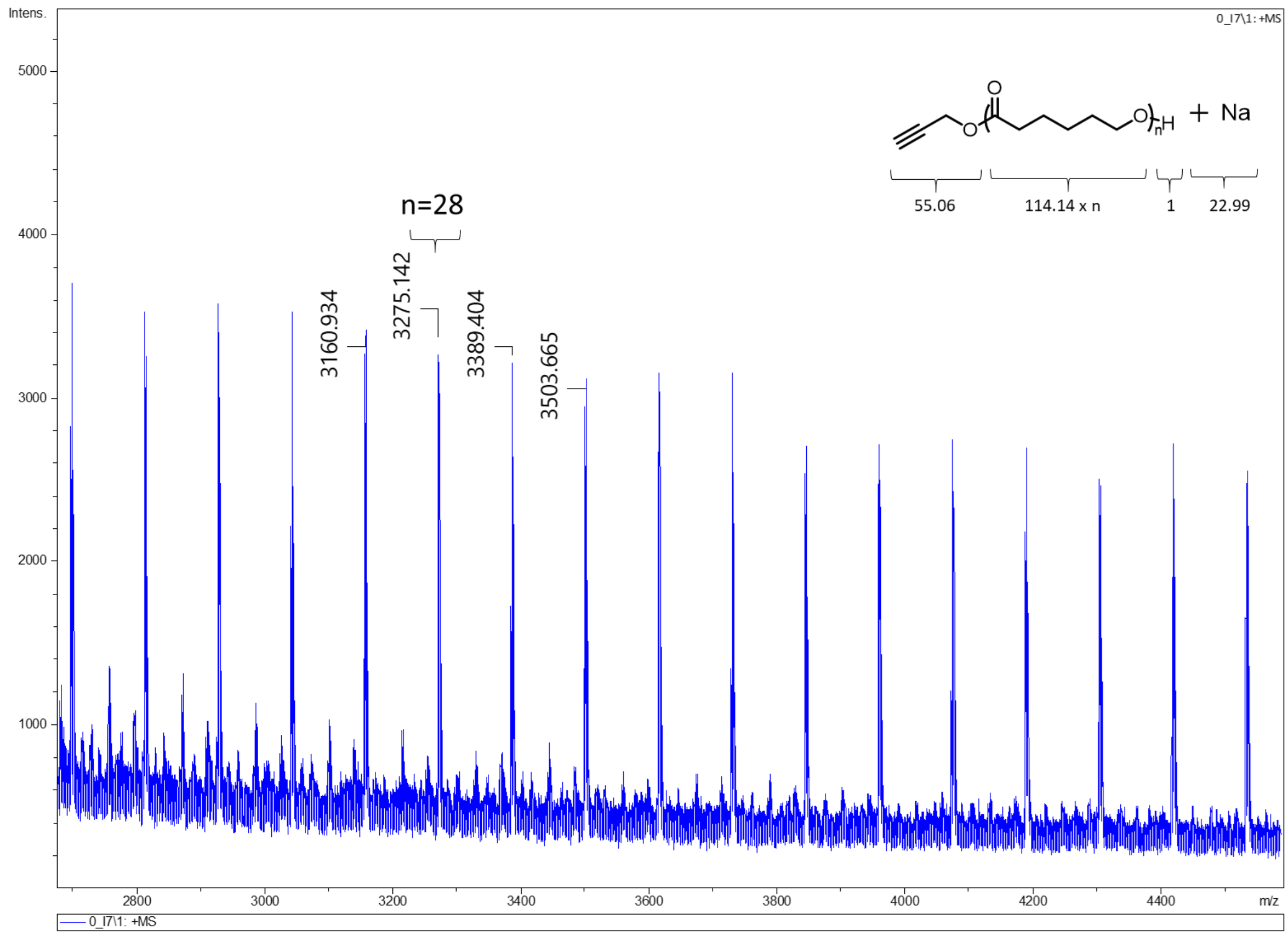

*Figure S59: MALDI-ToF spectrum of PCL (Table S1, entry 2) generated with $LAlOCH_2C{\equiv}CH$ **3**, confirming the presence of propynoxide end groups.*

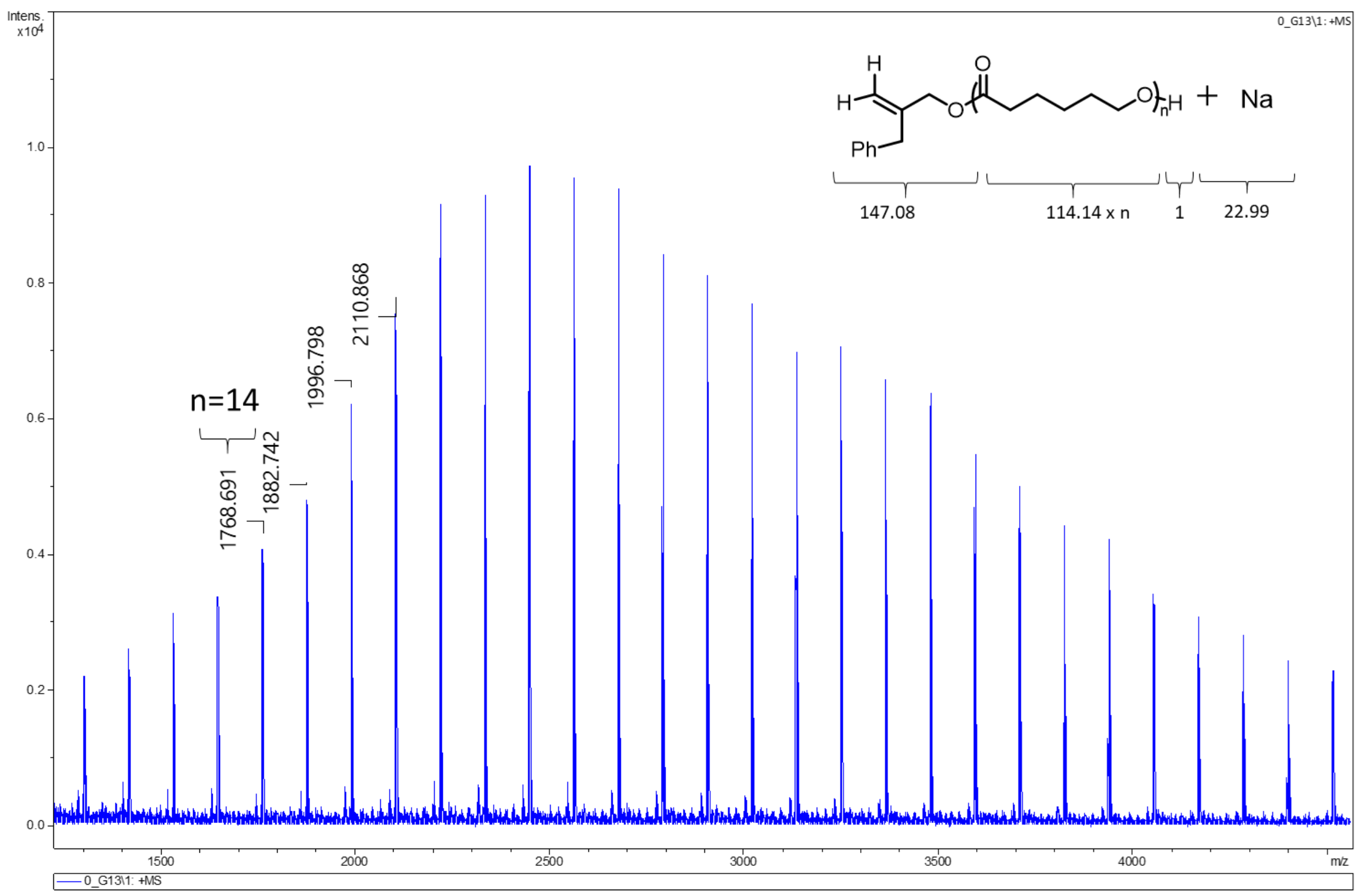

*Figure S60: MALDI-ToF spectrum of PCL produced from in situ generated $[LTi\text{-}CH{=}C(Bn)CH_2O\text{-}AlL][B(C_6F_5)_4]$ **4b**, confirming the presence of alkenoxide end groups.*

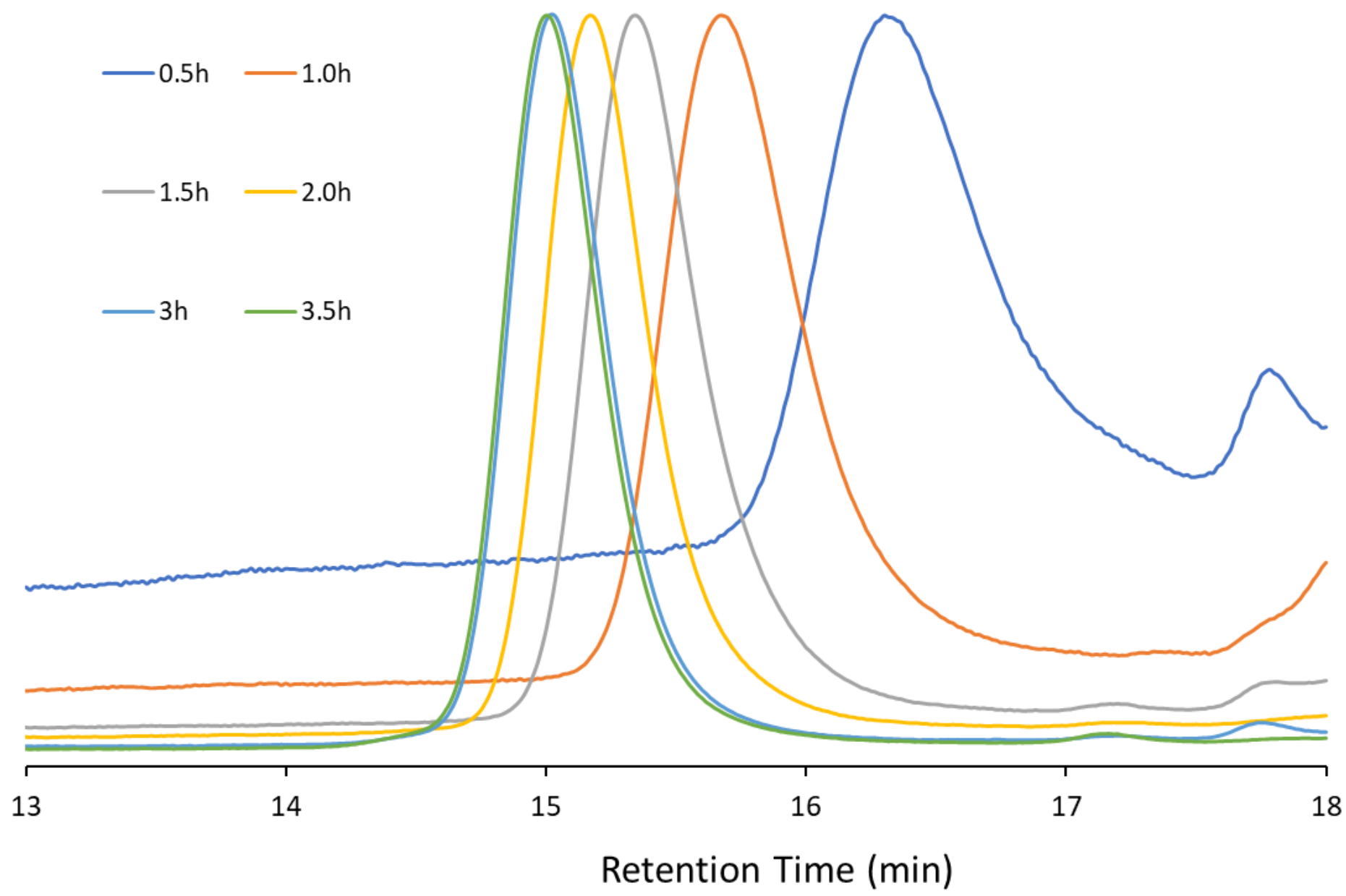

*Figure S61: SEC traces of PCL homopolymers (See Table S1 for details) generated with LAlOCH$_2$C≡CH **3**.*

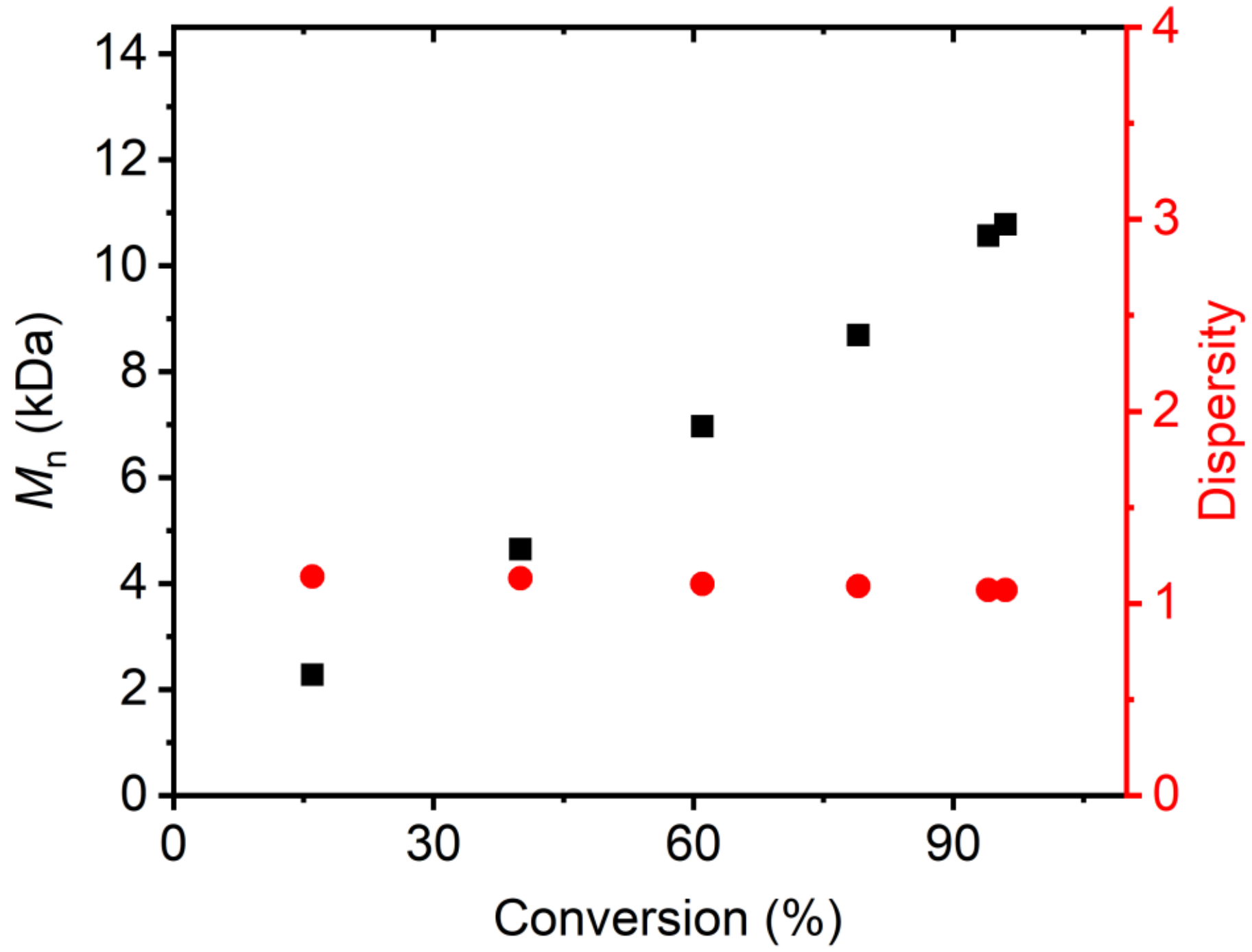

*Figure S62: A plot of $M_{n,obs}$ and dispersity of PCL homopolymers with respect to conversion (See Table S1 for details) generated with LAlOCH$_2$C≡CH **3**.*

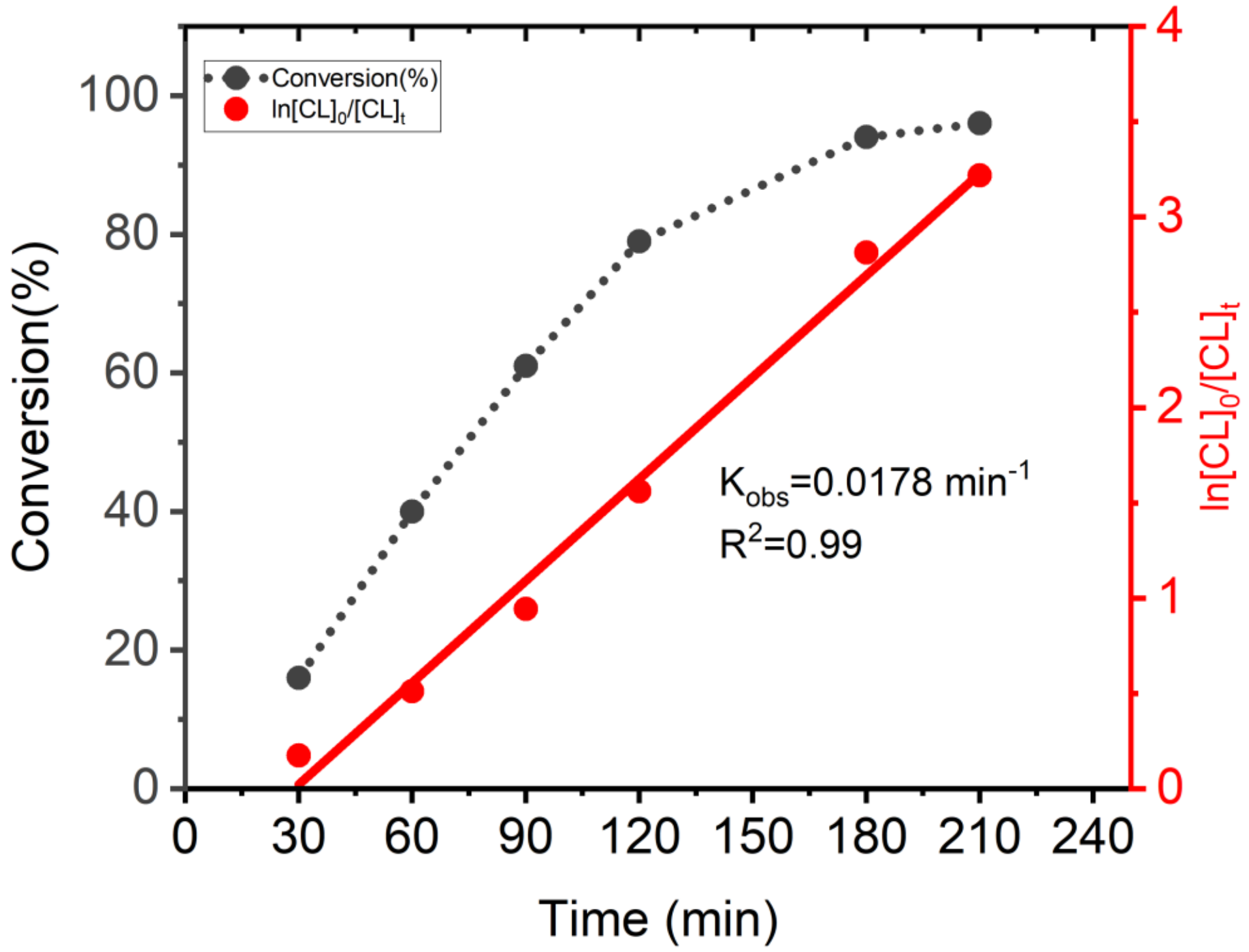

*Figure S63: A plot of conversion and ln$[CL]_0/[CL]_t$ with respect to time for CL homopolymerisation (See Table S1 for details) with LAlO$CH_2C{\equiv}CH$ **3** at room temperature.*

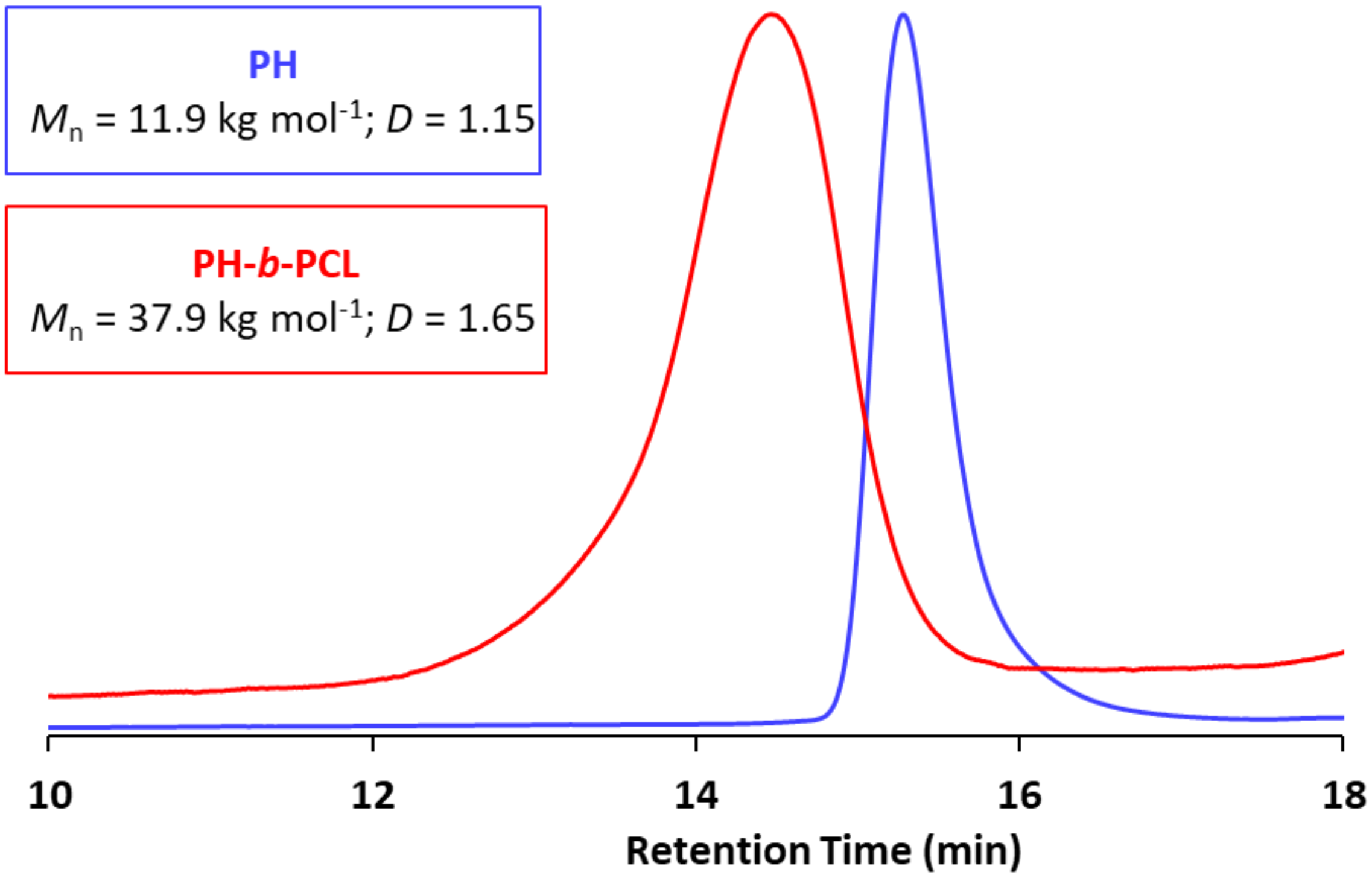

Figure S64: SEC traces of PH homopolymer and PH-b-PCL copolymer produced from in situ generated [LTiCH=C(Me)CH$_2$OAlL][MeB(C$_6$F$_5$)$_3$] ***4a*** *(Table 1, entry 4 and 10).*

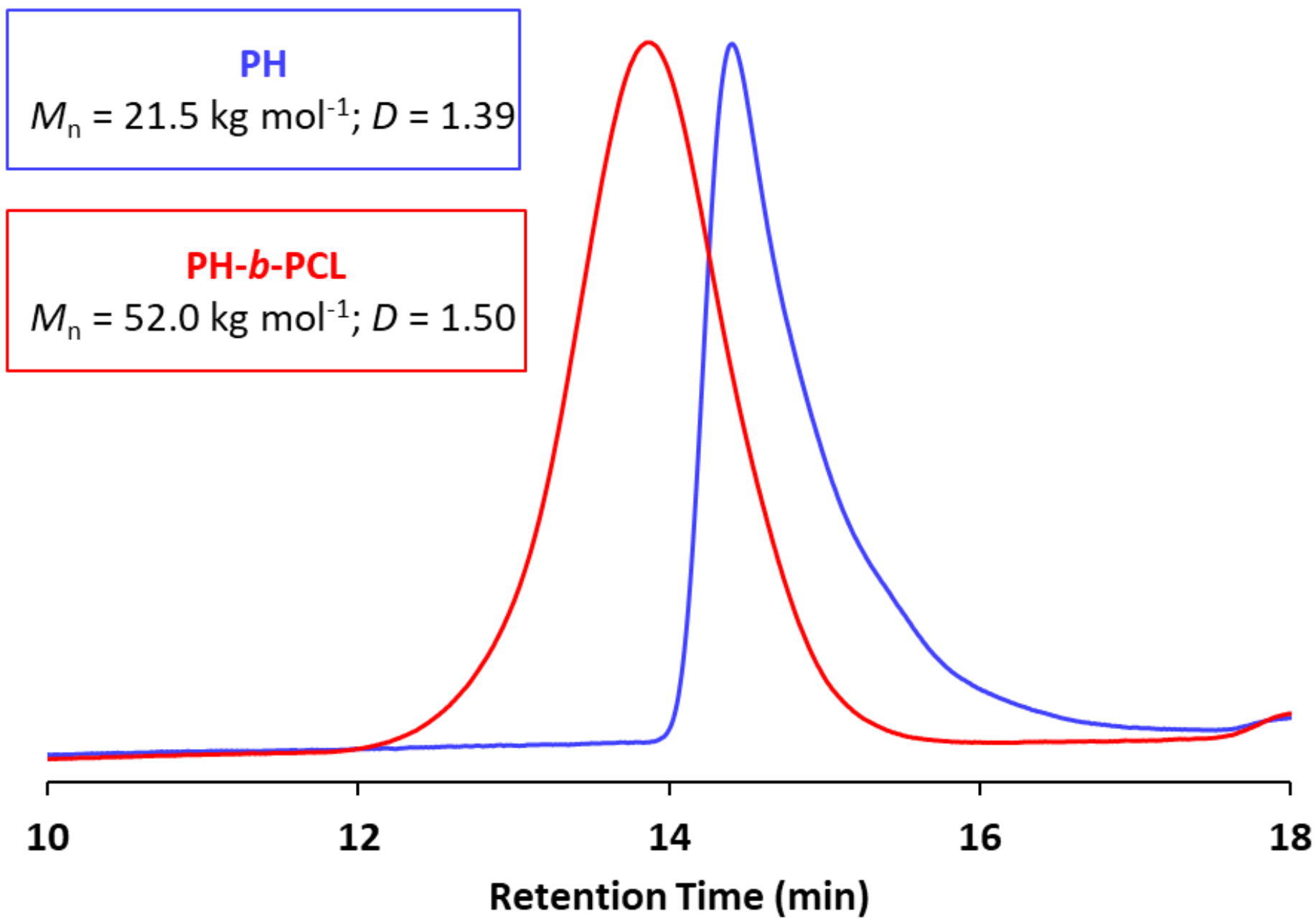

Figure S65: SEC traces of PH homopolymer and PH-b-PCL copolymer produced from in situ generated [LTiCH=C(Me)CH$_2$OAlL][MeB(C$_6$F$_5$)$_3$] ***4a*** *(Table 1, entry 5 and 11).*

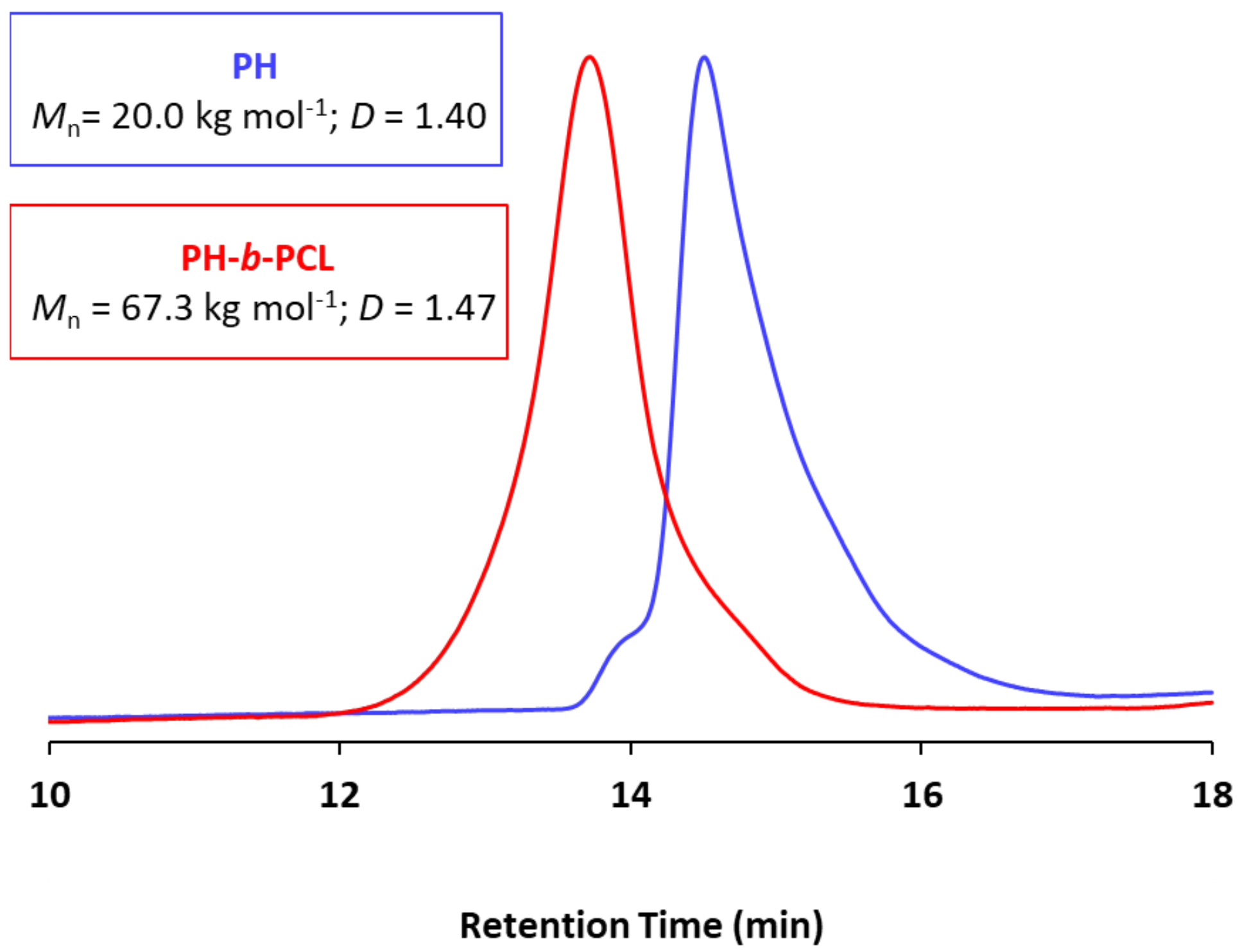

Figure S66: SEC traces of PH homopolymer and PH-b-PCL copolymer catalysed by [LTiCH=C(Bn)CH$_2$OAlL][B(C$_6$F$_5$)$_4$] ***4b*** *(Table 1, entry 7 and 13).*

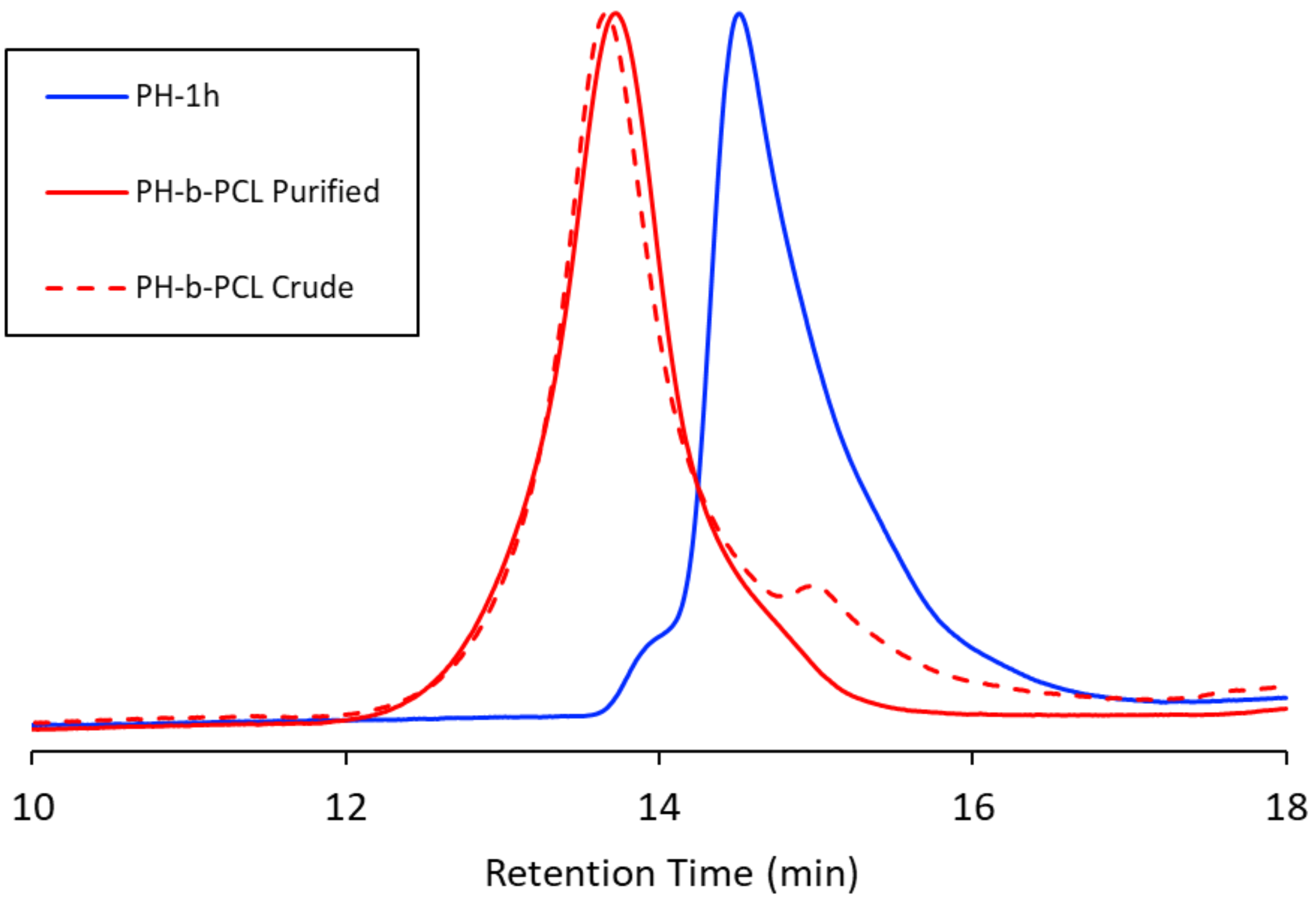

Figure S67: SEC traces of PH (Table 1, entry 7) homopolymer, PH-b-PCL (Table 1, entry 13) copolymer and the crude PH-b-PCL catalysed by [LTiCH=C(Bn)CH_2OAlL][B(C_6F_5)$_4$] ***4b*** *(Table 1, entry 7 and 13).*

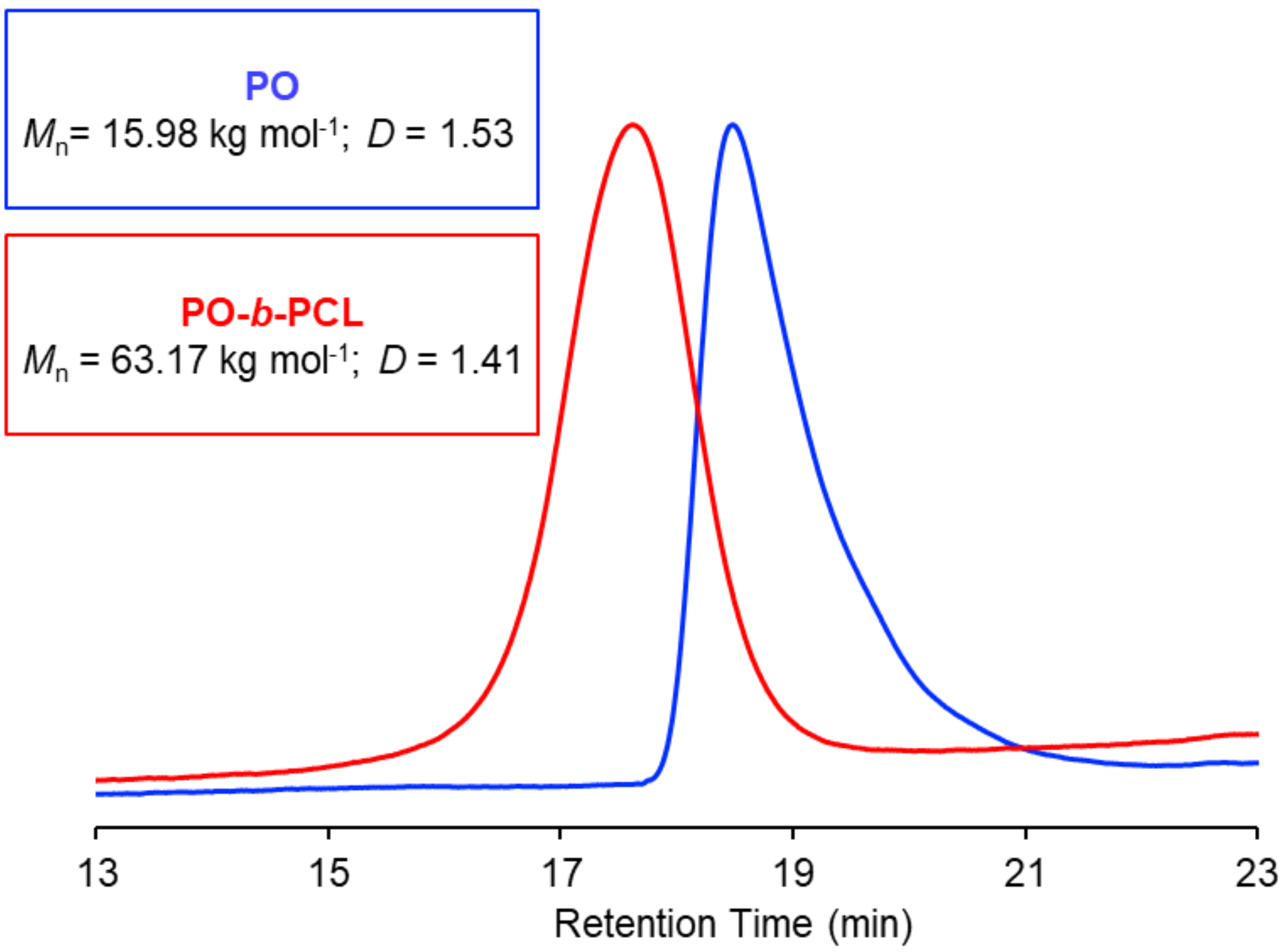

Figure S68: SEC traces of PO homopolymer and PO-b-PCL copolymer catalysed by [LTiCH=C(Bn)CH$_2$OAlL][B(C$_6$F$_5$)$_4$] ***4b*** *(Table 1, entry 15 and 16).*

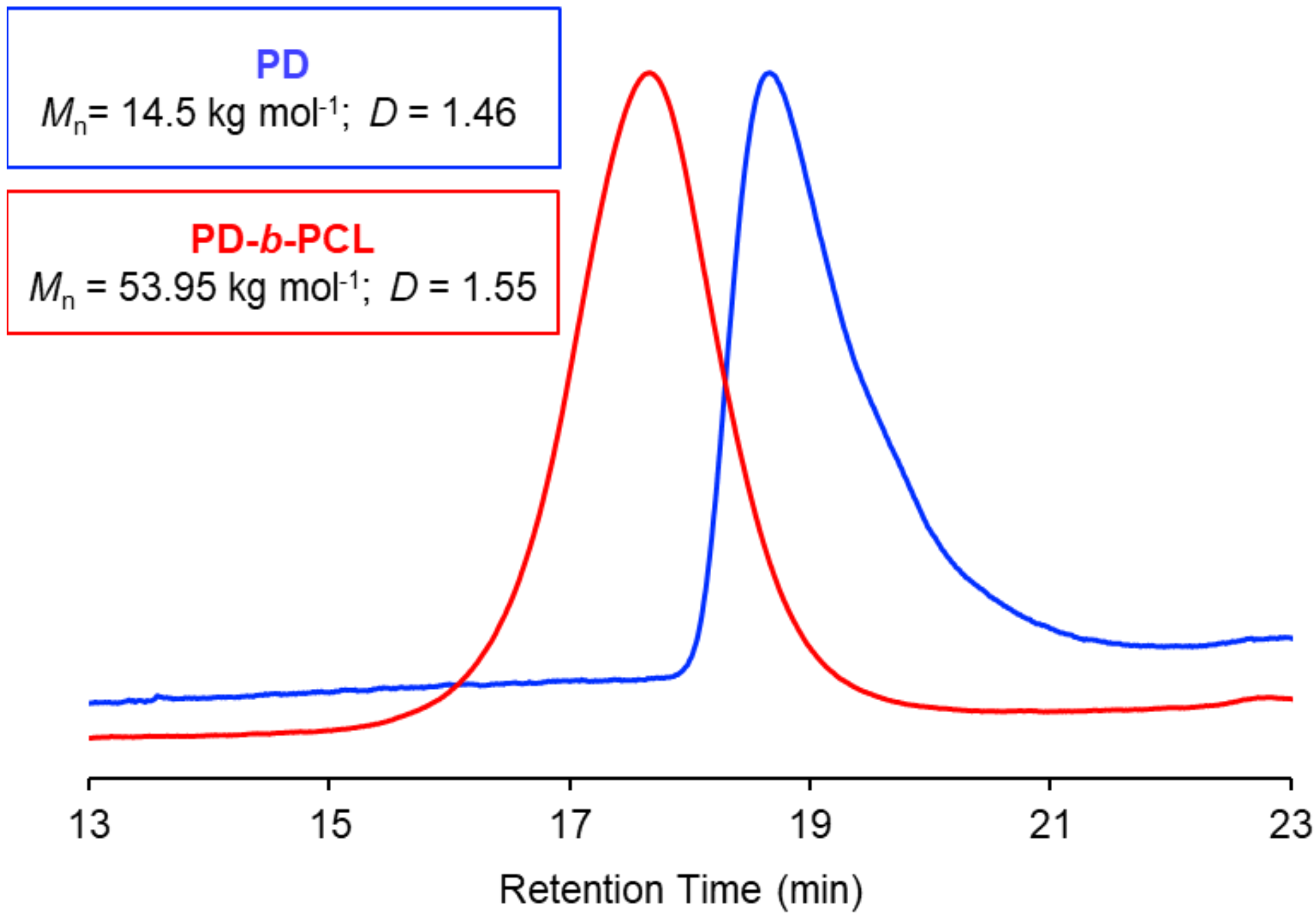

Figure S69: SEC traces of PD homopolymer and PD-b-PCL copolymer catalysed by [LTiCH=C(Bn)CH$_2$OAlL][B(C$_6$F$_5$)$_4$] ***4b*** *(Table 1, entry 17 and 18).*

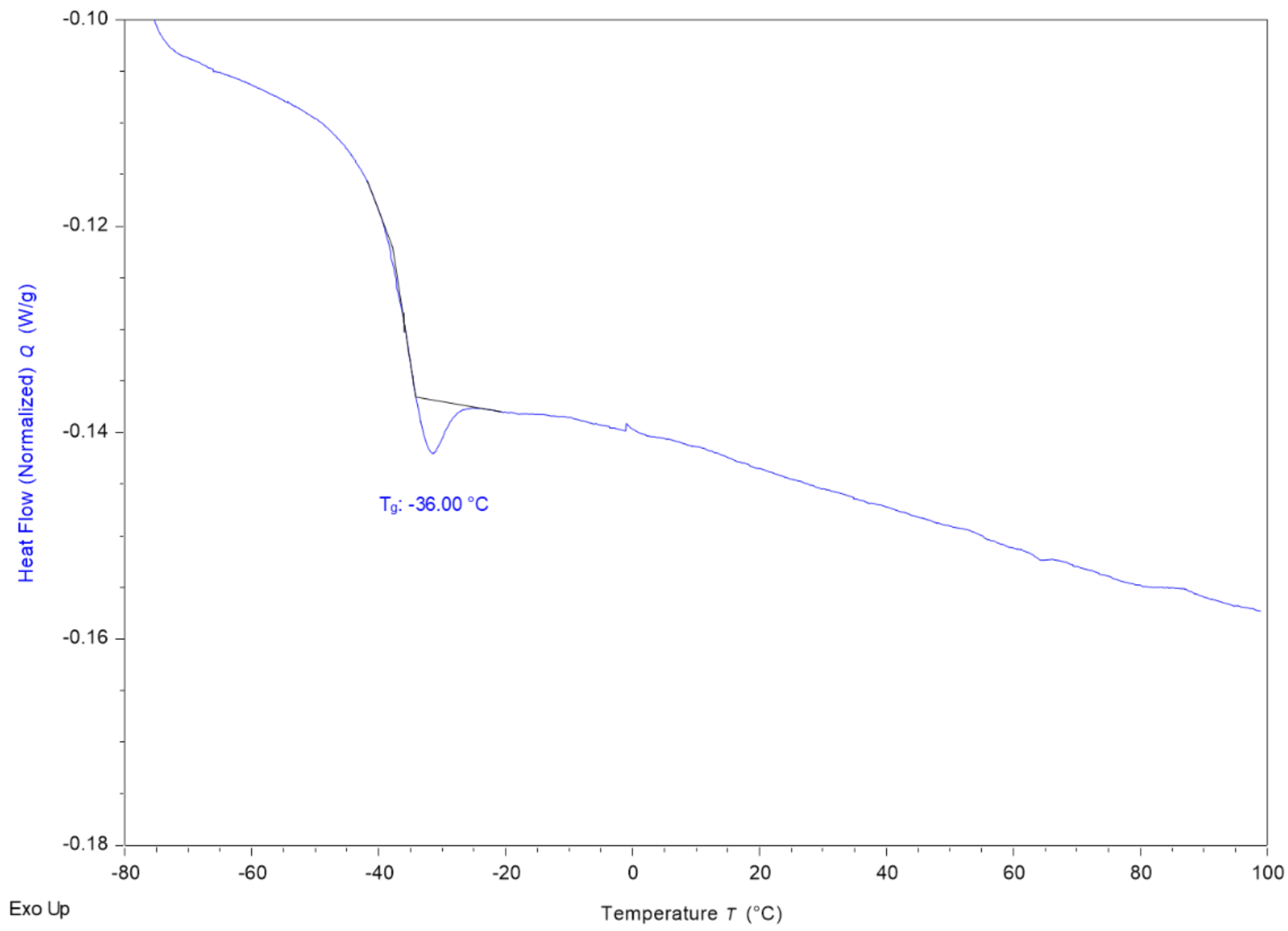

*Figure S70: DSC thermogram (second heating cycle) of PH (Table 1, entry 7) produced from in situ generated [LTiCH=C(Bn)CH_2OAlL][B$(C_6F_5)_4$] **4b**.*

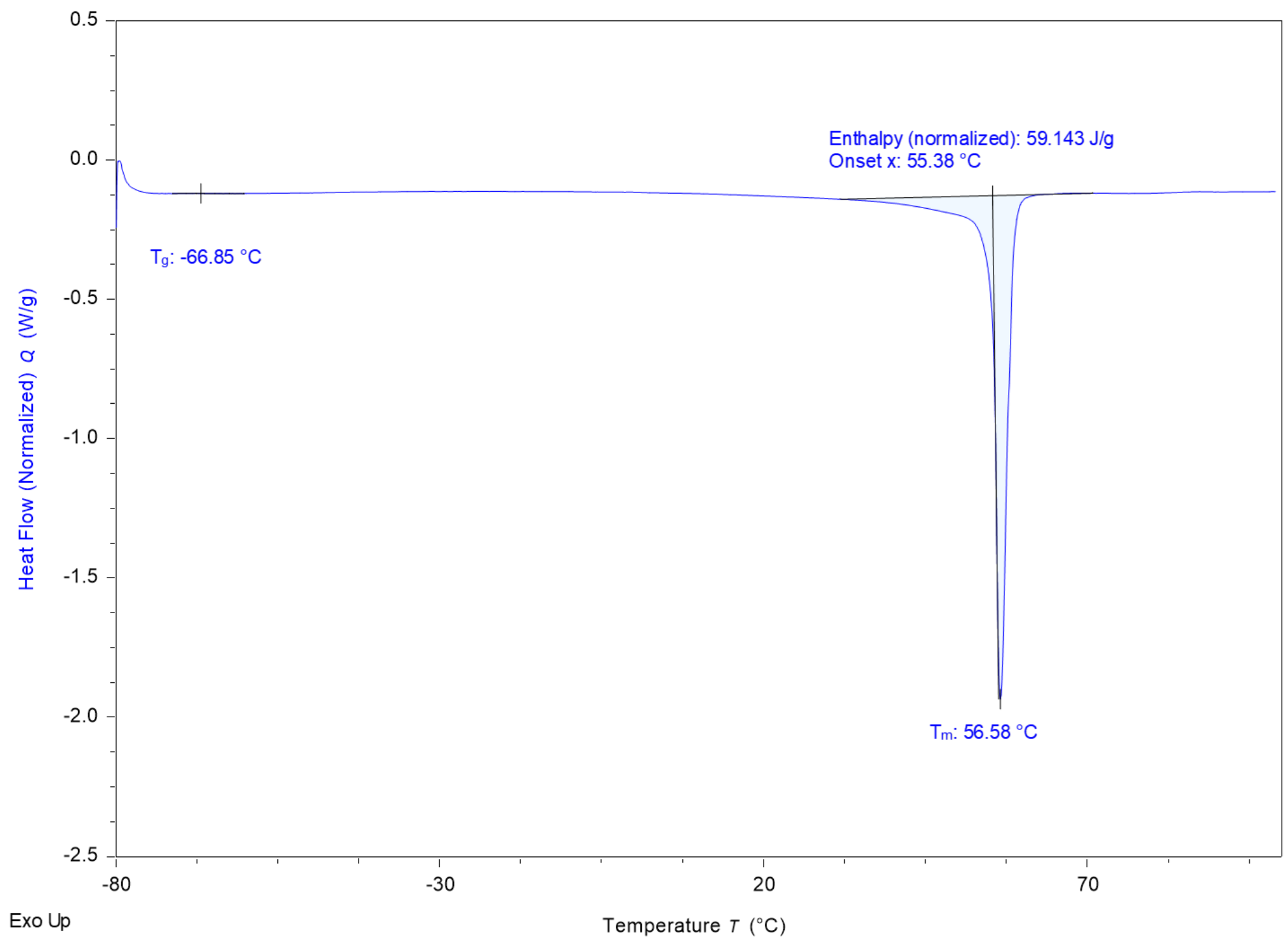

*Figure S71: DSC thermogram (second heating cycle) of PCL (Table 1, entry 9) produced from LAlOCH_2C≡CH **3**.*

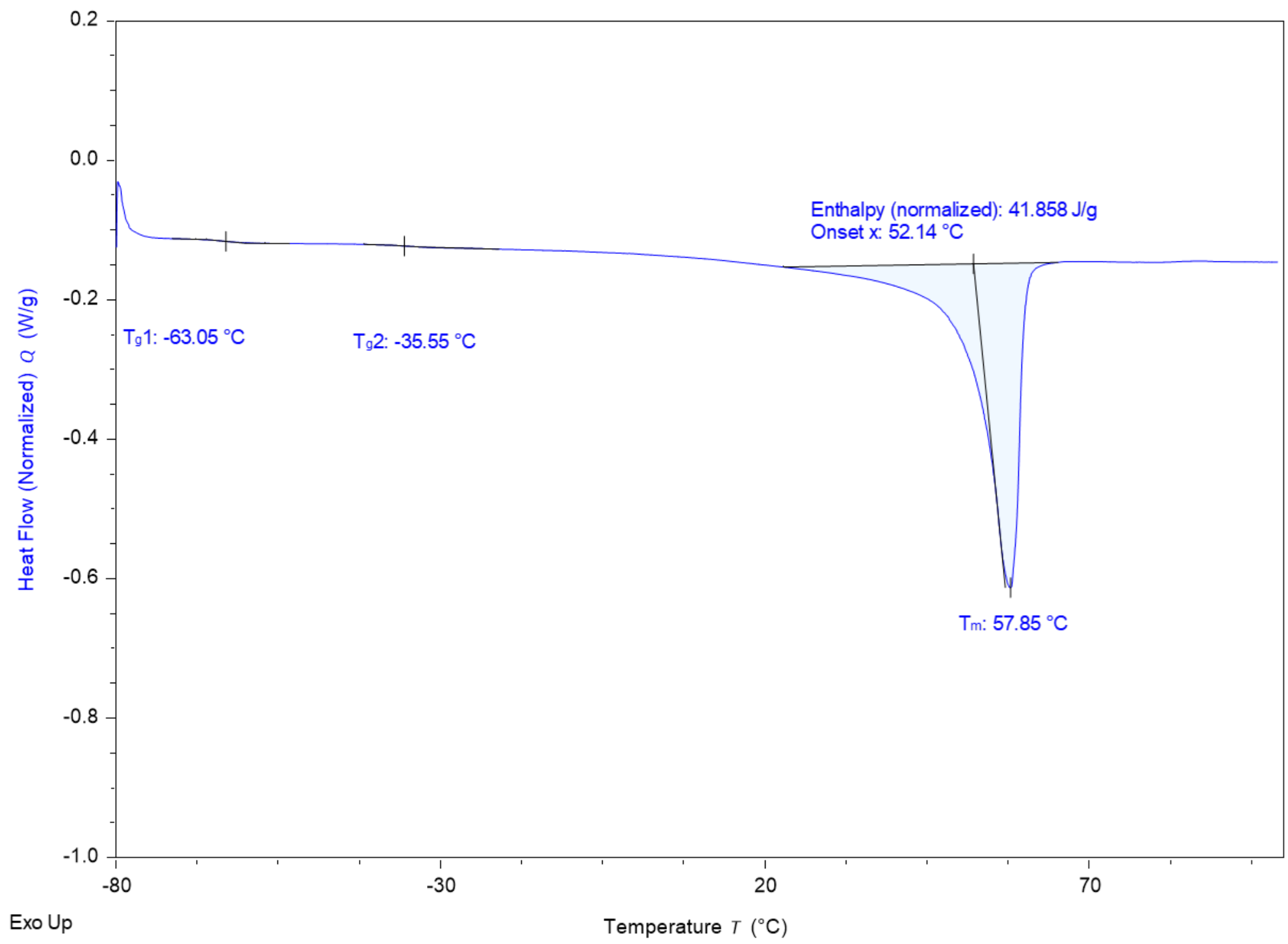

Figure S72: DSC thermogram (second heating cycle) of PH-b-PCL (Table 1, entry 12) produced from in situ generated [LTiCH=C(Bn)CH$_2$OAlL][B(C$_6$F$_5$)$_4$] ***4b****.*

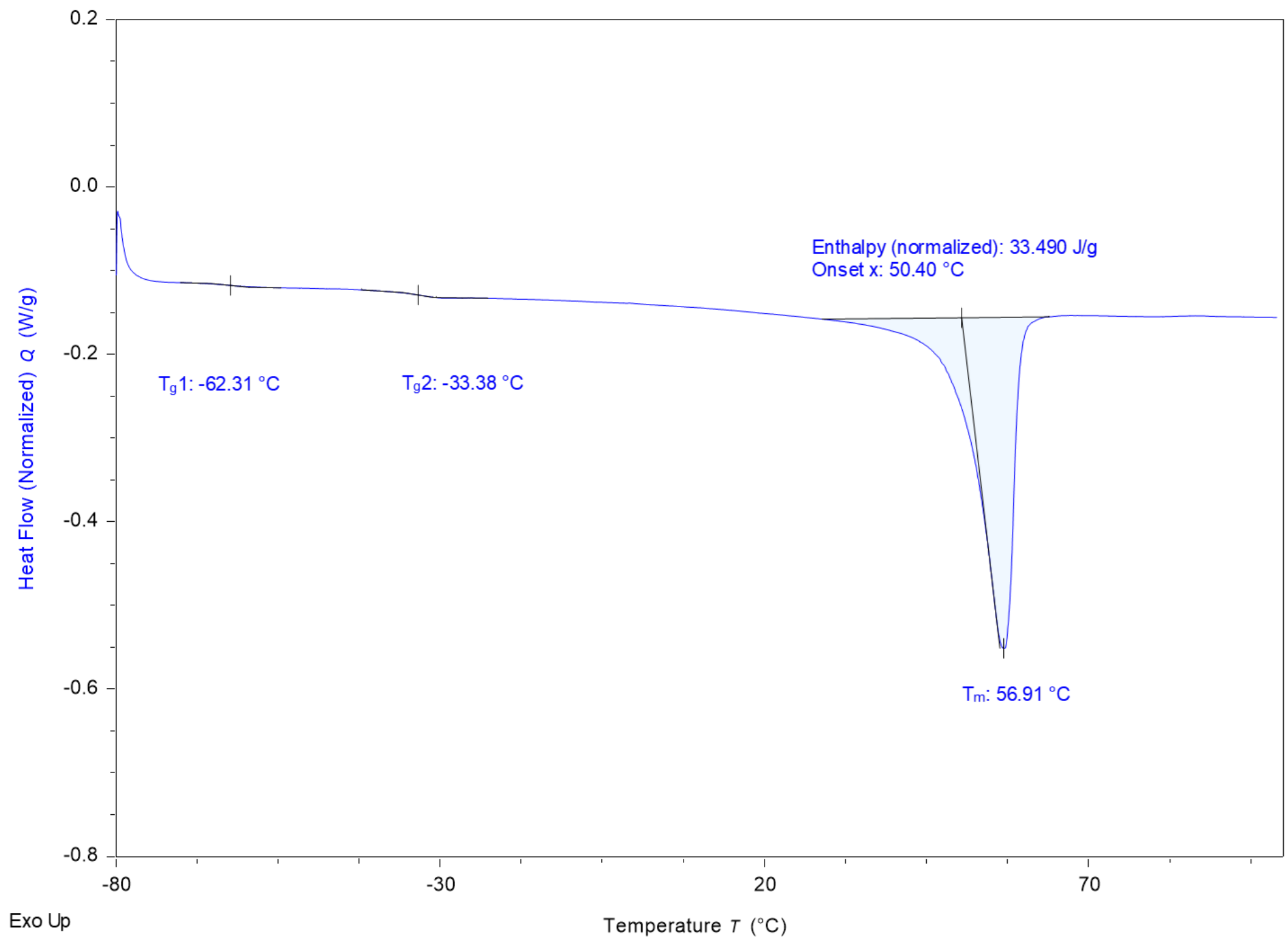

Figure S73: DSC thermogram (second heating cycle) of PH-b-PCL (Table 1, entry 13) produced from in situ generated [LTiCH=C(Bn)CH$_2$OAlL][B(C$_6$F$_5$)$_4$] ***4b.***

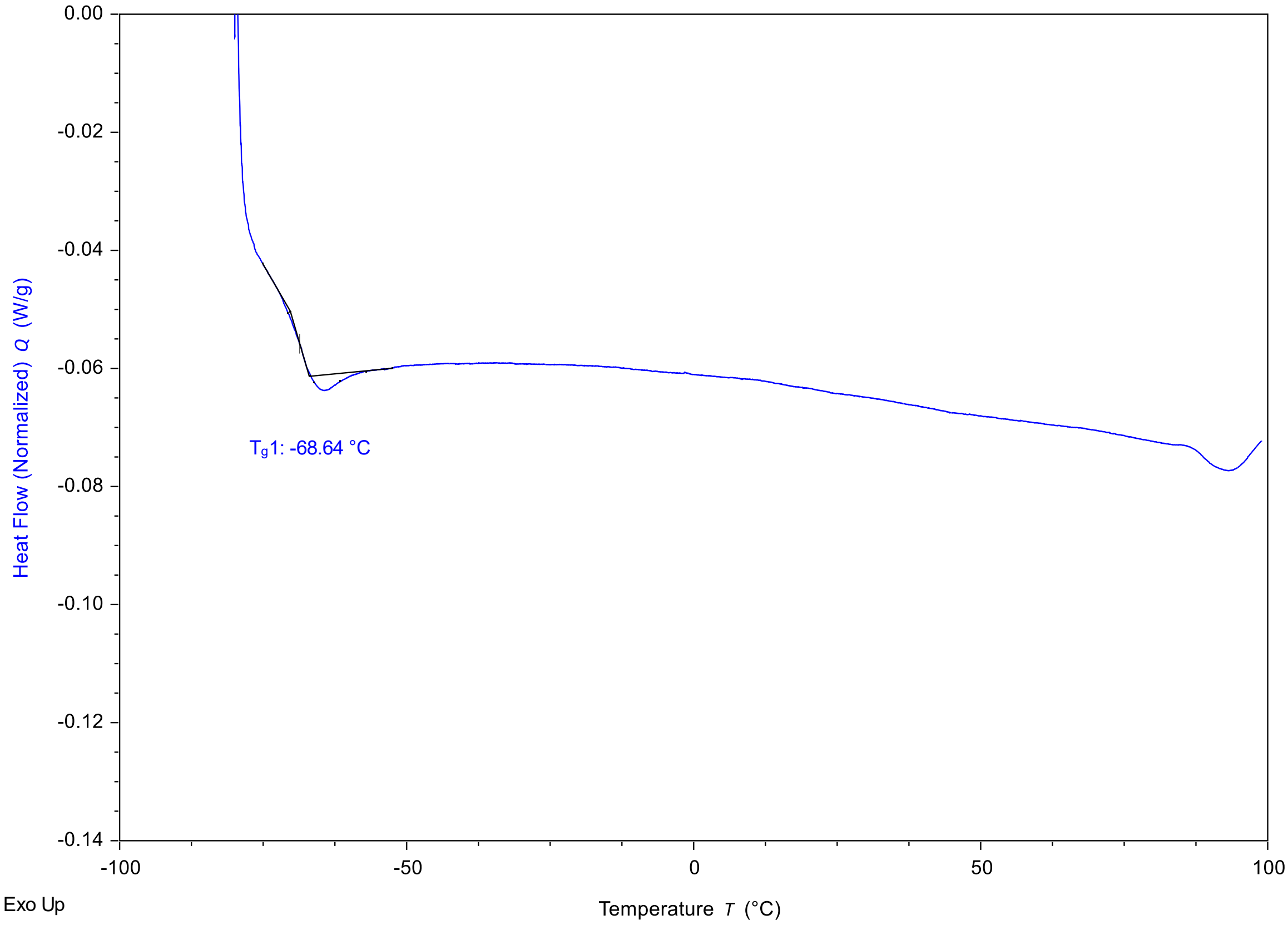

Figure S74: DSC thermogram (second heating cycle) of PO (Table 1, entry 15) produced from in situ generated [LTiCH=C(Bn)CH_2OAlL][B$(C_6F_5)_4$] ***4b.***

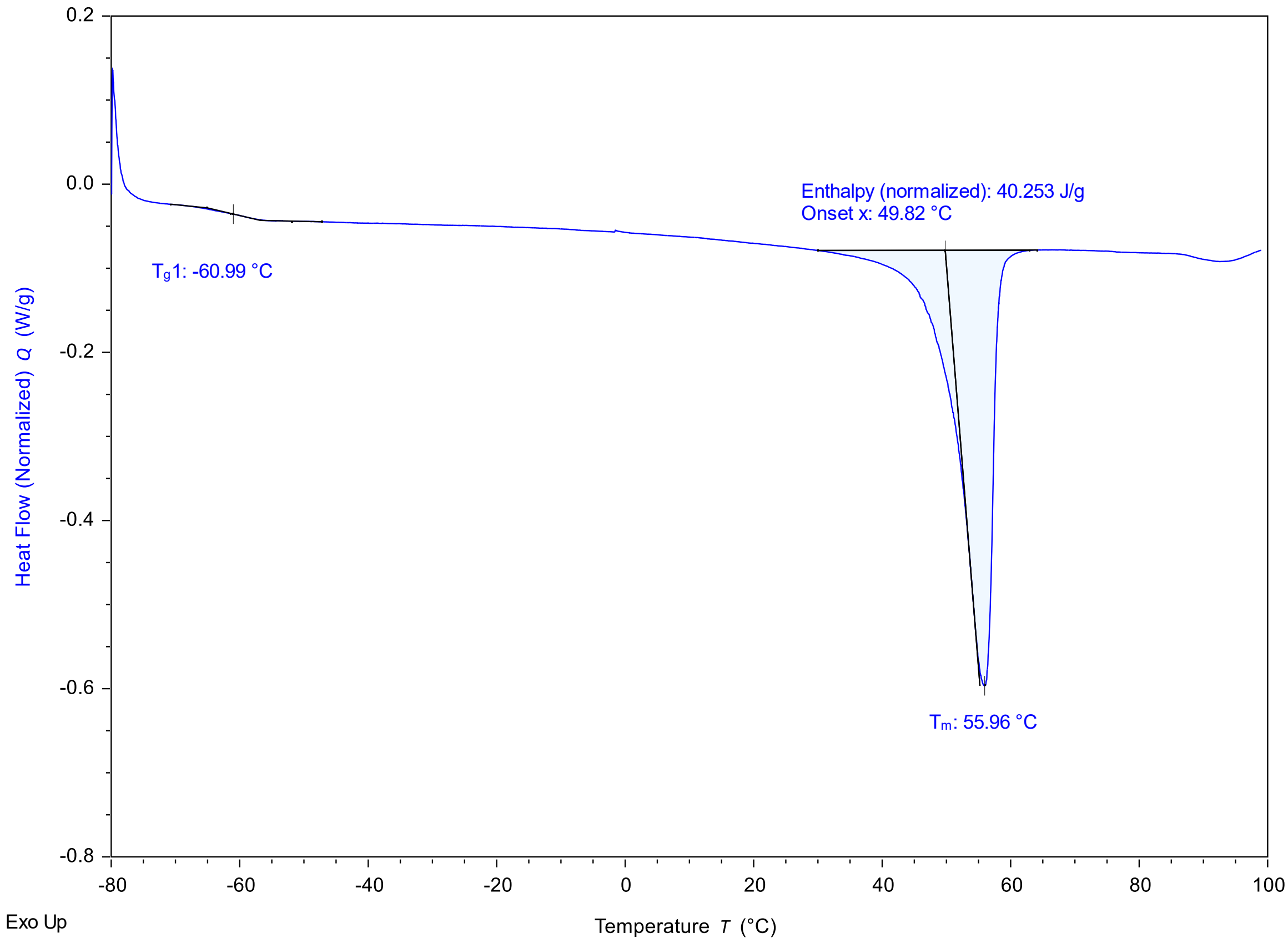

Figure S75: DSC thermogram (second heating cycle) of PO-b-PCL (Table 1, entry 16) produced from in situ generated [LTiCH=C(Bn)CH_2OAlL][B(C_6F_5)$_4$] ***4b.***

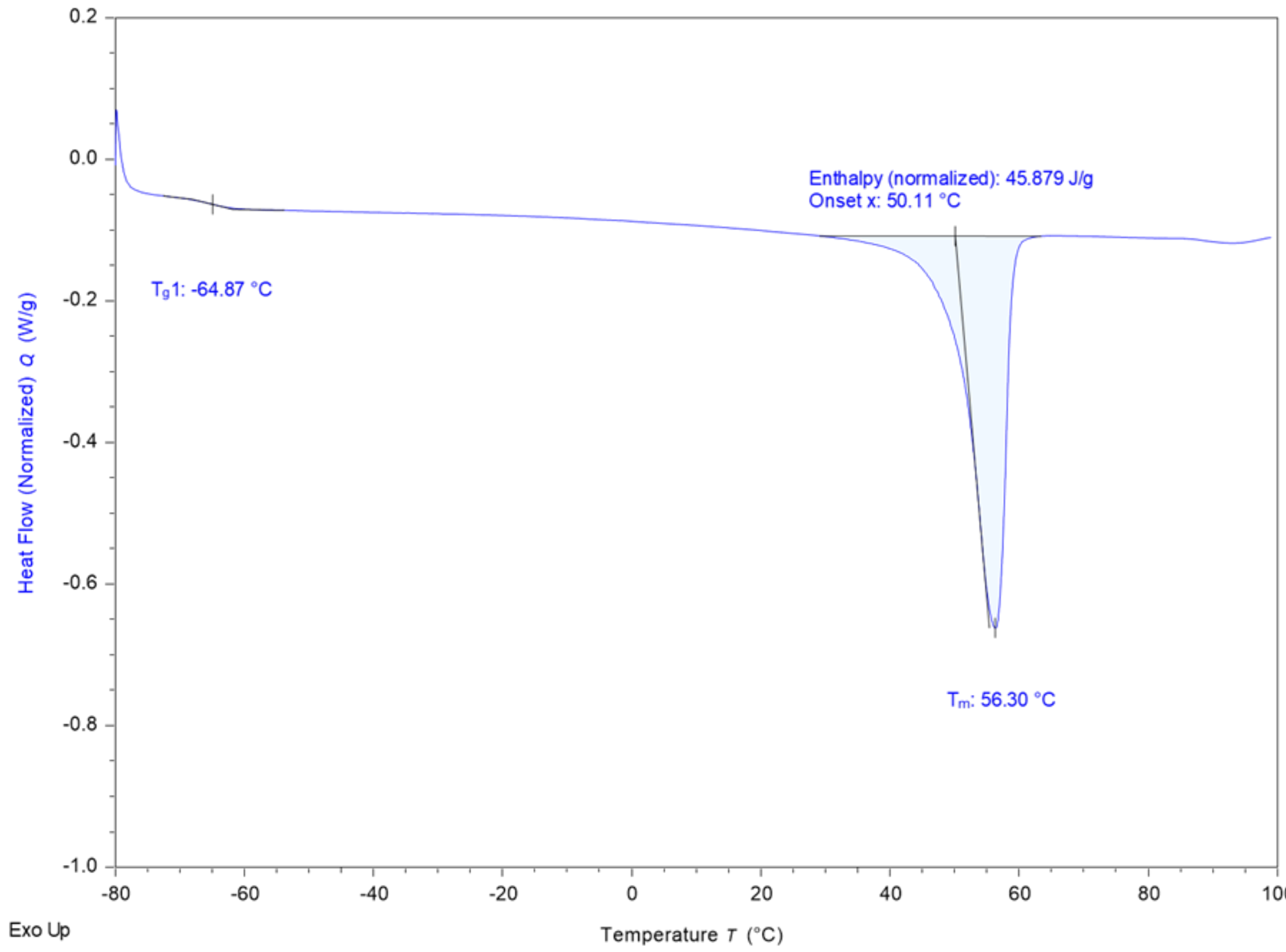

Figure S76: DSC thermogram (second heating cycle) of PD-b-PCL (Table 1, entry 18) produced from in situ generated [LTiCH=C(Bn)CH_2OAlL][B(C_6F_5)$_4$] ***4b.***

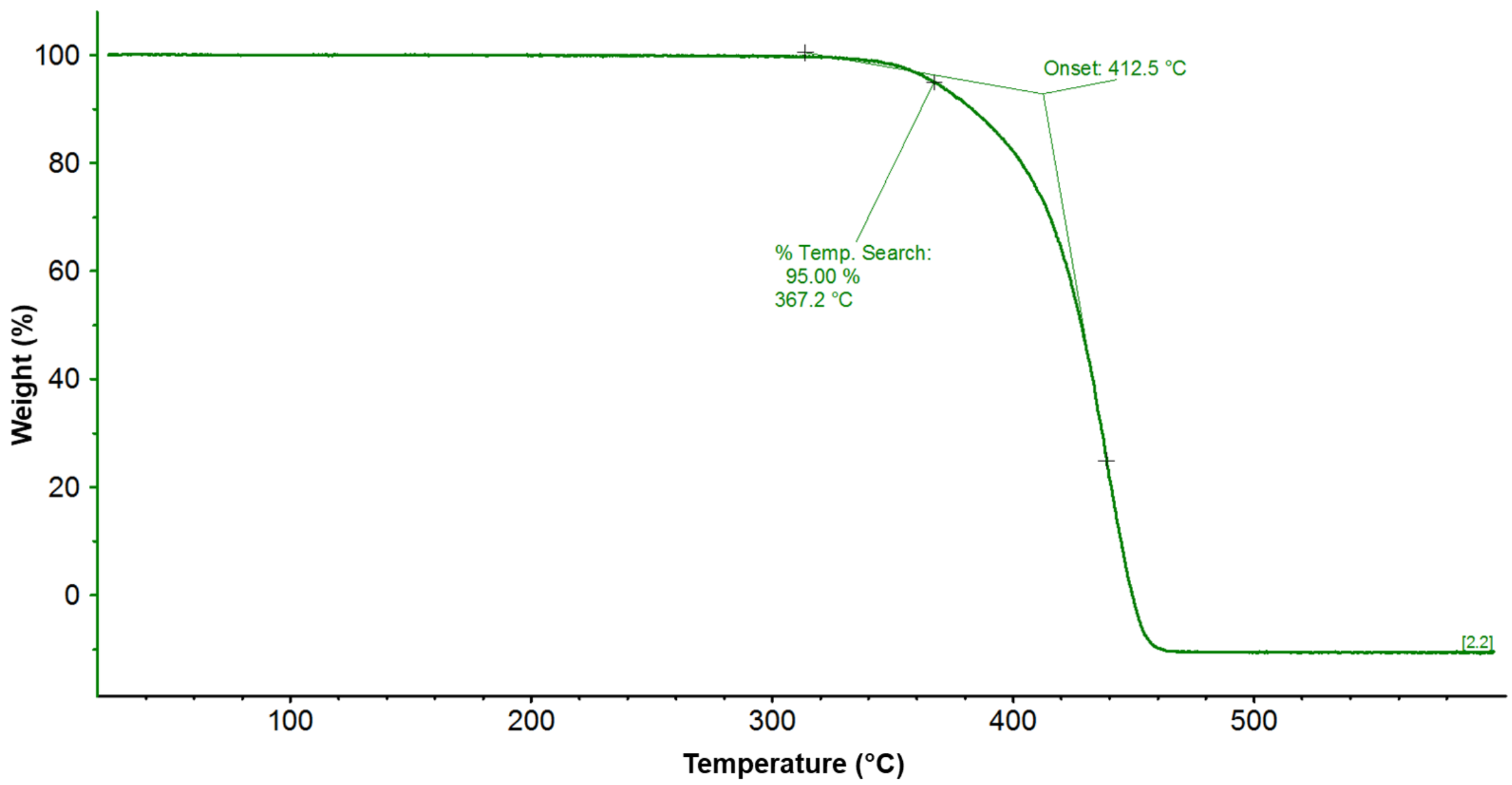

Figure S77: TGA thermogram of PH (Table 1, entry 7) produced from in situ generated [LTiCH=C(Bn)CH_2OAlL][B(C_6F_5)$_4$] ***4b.***

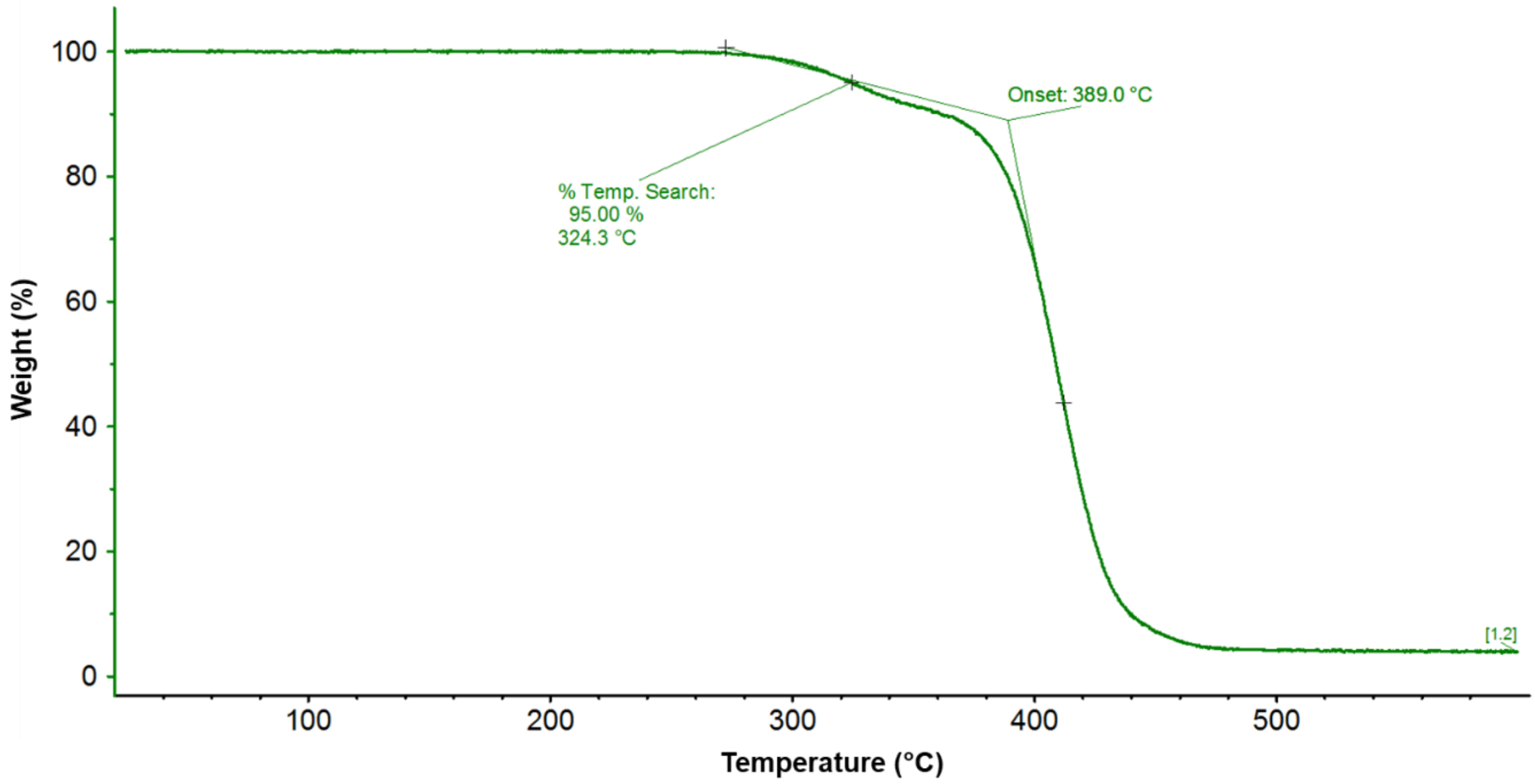

*Figure S78: TGA thermogram of PCL (Table 1, entry 9) produced from LAlOCH$_2$C≡CH **3**.*

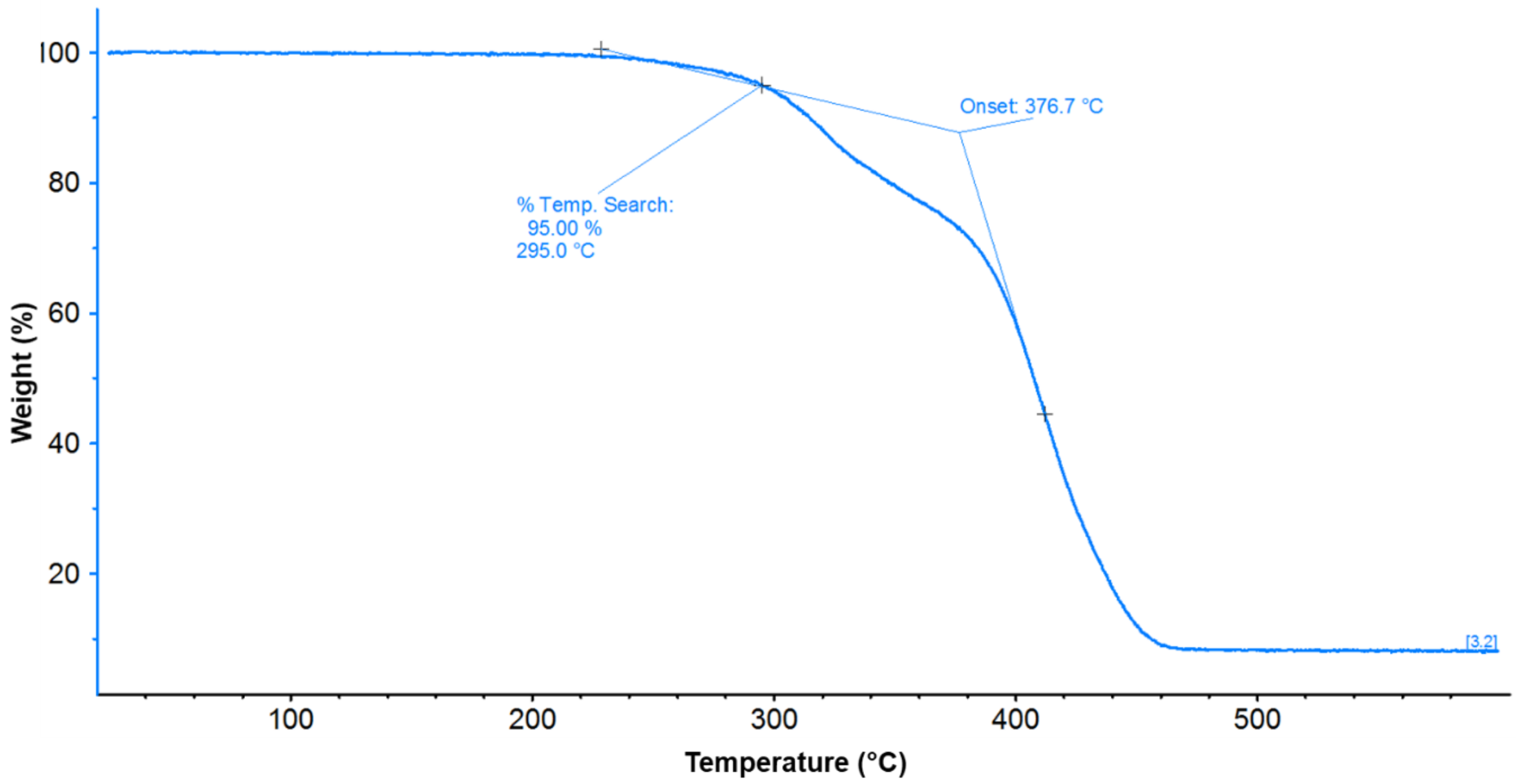

*Figure S79: TGA thermogram of PH-b-PCL (Table 1, entry 13) produced from in situ generated [LTiCH=C(Bn)CH$_2$OAlL][B(C$_6$F$_5$)$_4$] **4b.***

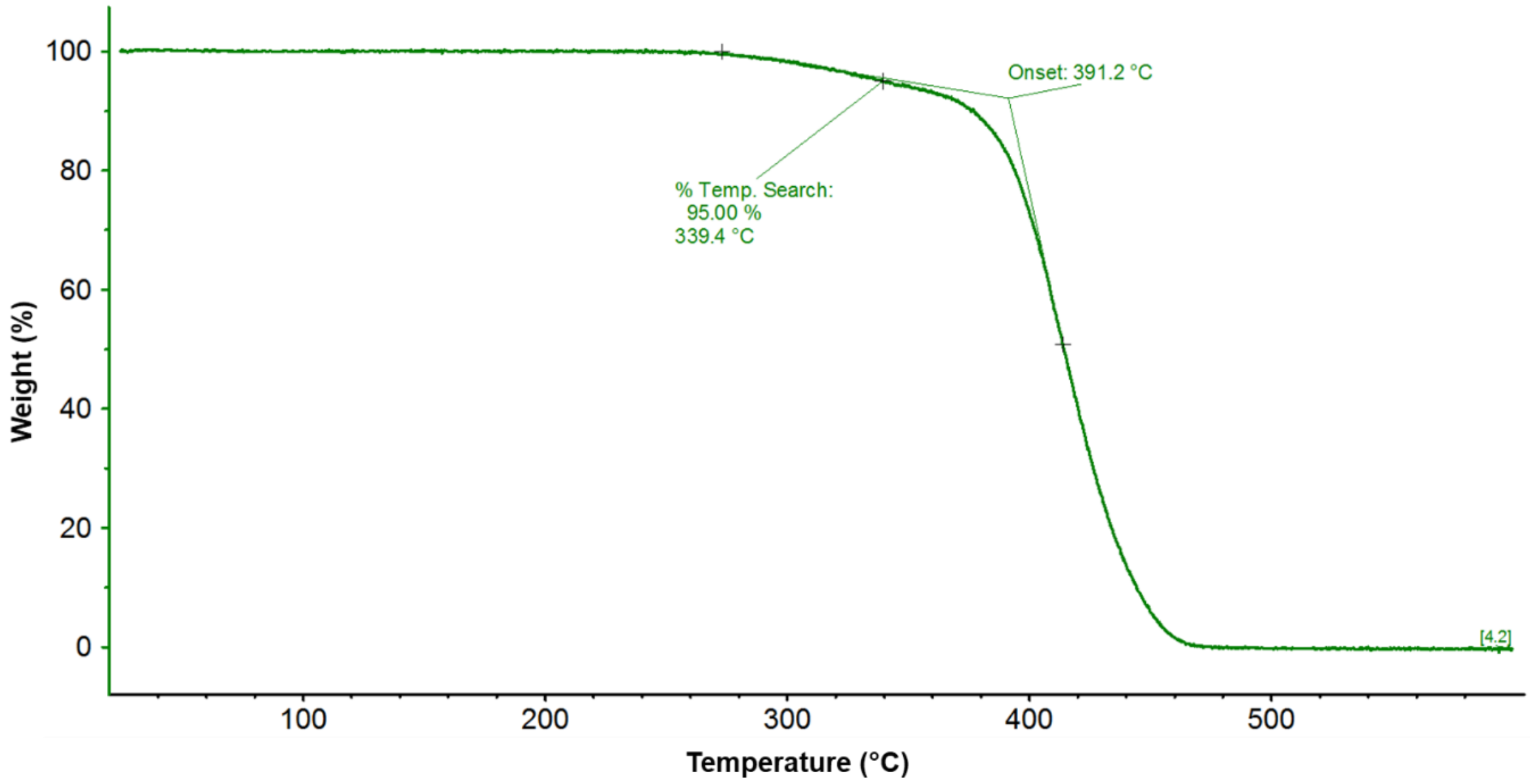

Figure S80: TGA thermogram of PO-b-PCL (Table 1, entry 16) produced from in situ generated [LTiCH=C(Bn)CH_2OAlL][B(C_6F_5)$_4$] ***4b.***

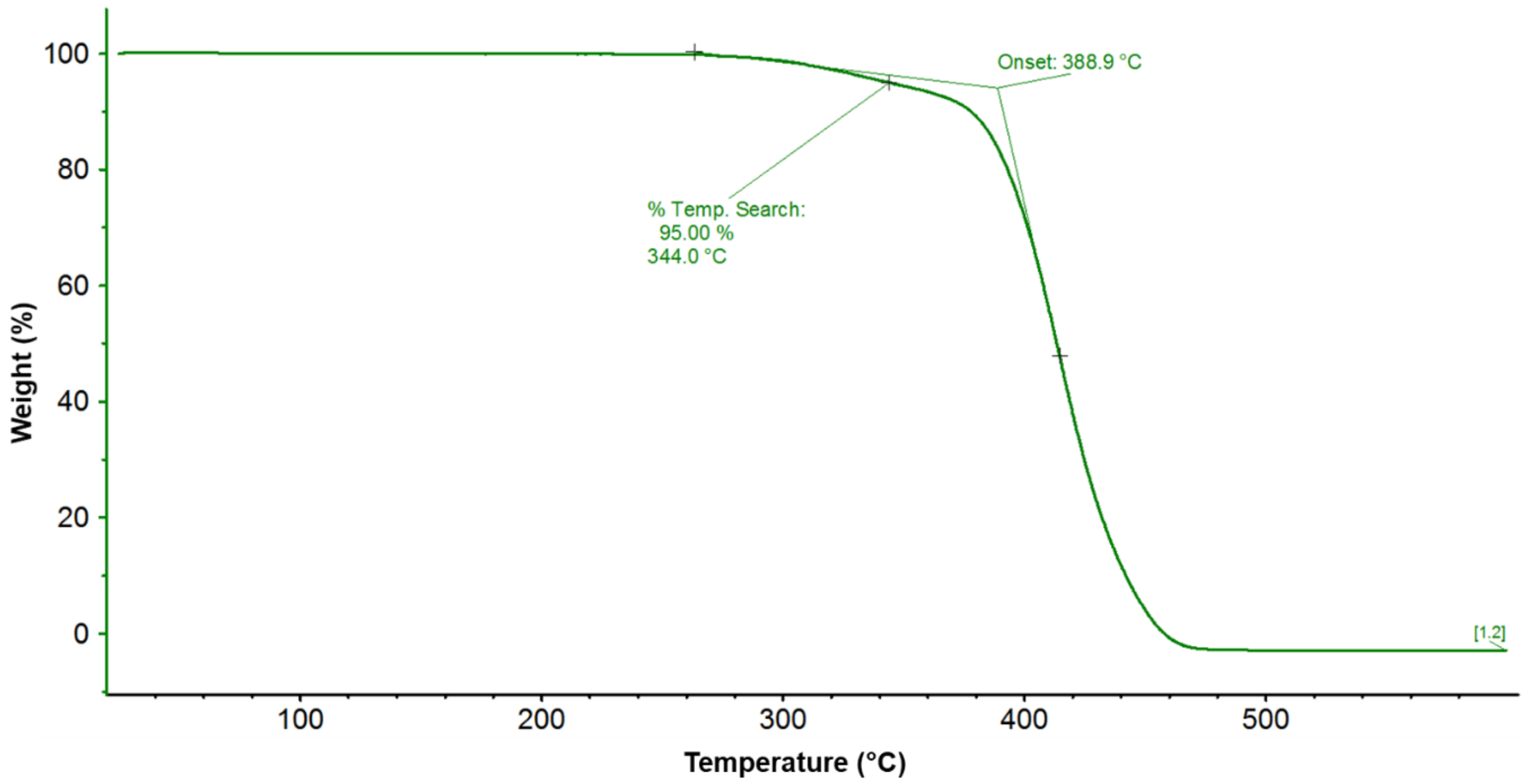

Figure S81: TGA thermogram of PD-b-PCL (Table 1, entry 18) produced from in situ generated [LTiCH=C(Bn)CH_2OAlL][B(C_6F_5)$_4$] ***4b****.*

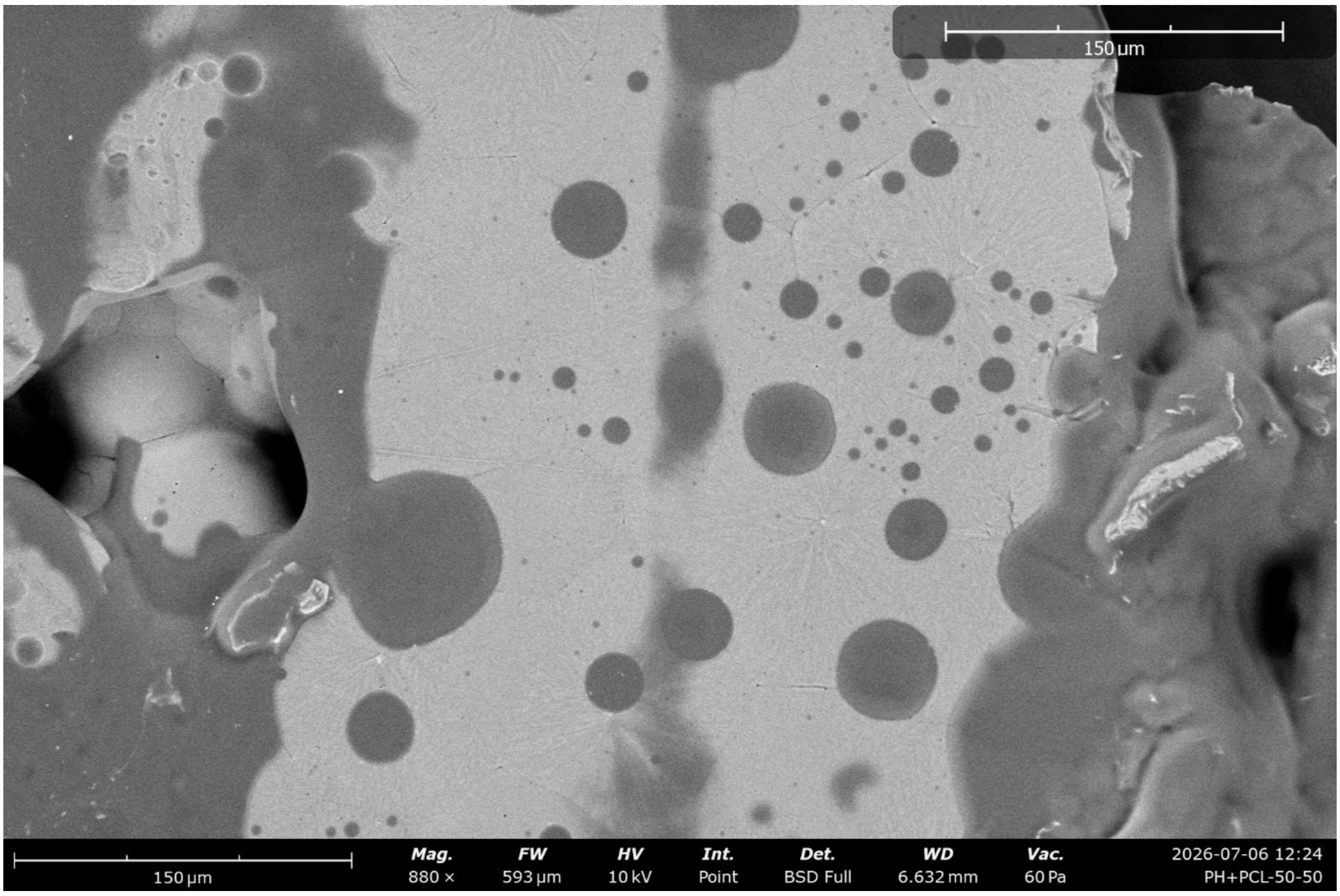

Figure S82: SEM image of a 50:50 PH:PCL blend (using PH and PCL from Table 1, entries 7 and 9).

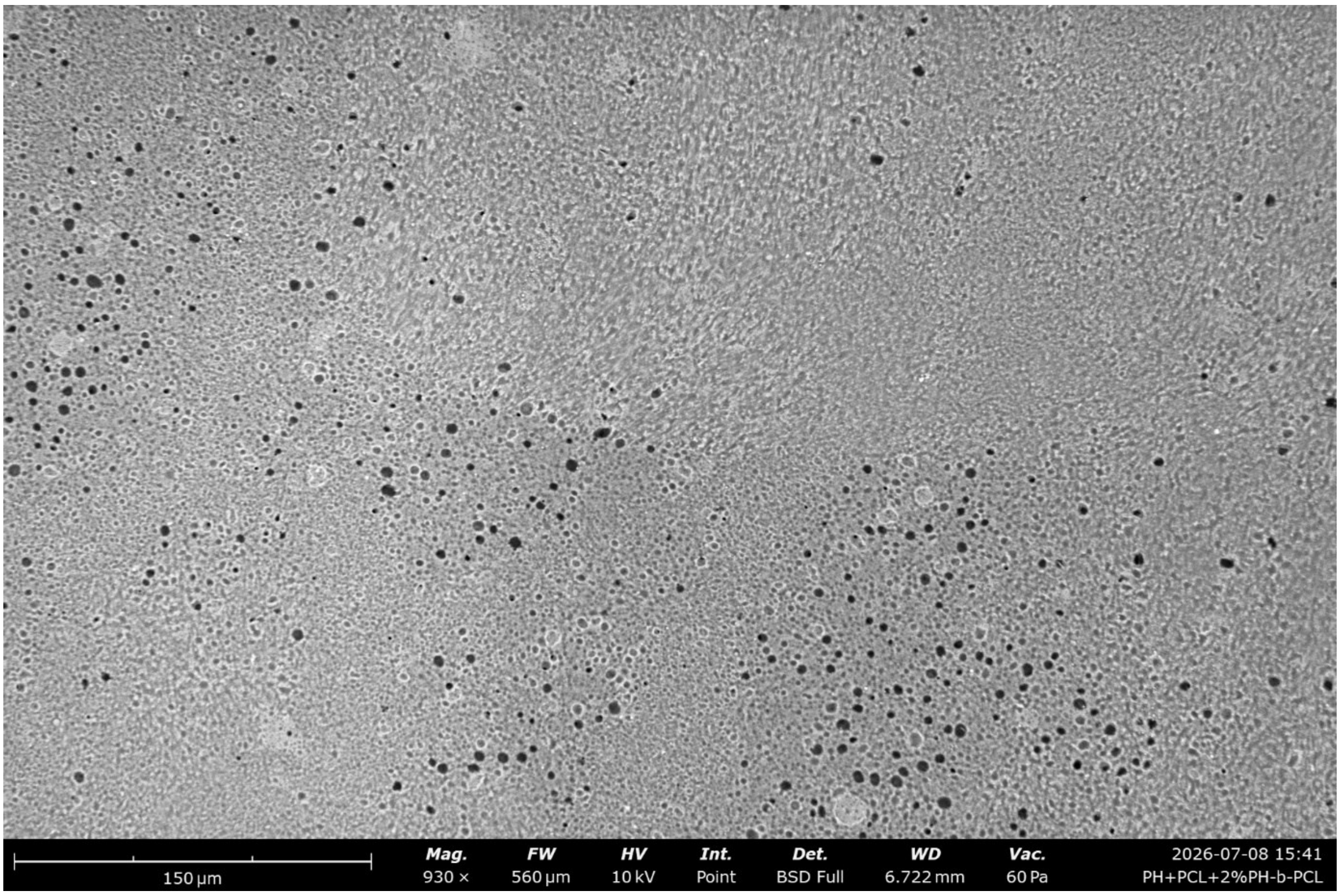

Figure S83: SEM image of a 50:50 PH:PCL blend (using PH and PCL from Table 1, entries 7 and 9)) with 2% of PH-b-PCL (Table 1, entry 13) added as a compatibiliser.

Crystallography data

Crystals for single-crystal X-ray diffraction experiments were first coated in Paratone oil, then a suitable sample was selected on a Mitigen tip and mounted on the diffractometer. Data for **1a**, **1b**, **2b** and **3** was collected using either a Bruker D8 VENTURE diffractometer or Rigaku SuperNova or Rigaku XtaLAB Synergy-R, diffractometer, with Mo or Cu K-alpha radiation and a crystal temperature of 100 or 120 K. The structures were solved by direct or dual-space methods using ShelXS[7] or ShelXT 2018/2.[8] The model was refined with ShelXL[9] or olex2.refine[10] using full matrix least squares minimisation on F^2. Olex2 1.5-beta[11] was used as the graphical interface to both programs.

*Table S3: Crystallographic data and refinement detail for compound **1a, 2a, 2b** and **3**.*

**Compounds**	**1a**	**2a**	**2b**	**3**
Formula	$C_{36}H_{60}N_2O_2Ti$	$C_{123}H_{156}B_2F_{30}N_4O_4Ti_2$	$C_{70}H_{73}BF_{20}N_2O_2Ti$	$C_{37}H_{57}AlN_2O_3$
μ/mm^{-1}	0.268	1.940	0.221	0.748
Formula Weight	600.757	2441.93	1413.01	604.858
Size/mm^3	0.37×0.33×0.23	0.21×0.03×0.02	0.40×0.07×0.04	0.15×0.03×0.03
T/K	120.00(10)	100.0	100.00	120.00(12)
Crystal System	monoclinic	triclinic	orthorhombic	tetragonal
Space Group	$P2_1/n$	*P*-1	*Pbca*	*I*-4
a/Å	11.6803(3)	13.5469(3)	20.703(3)	28.2480(6)
b/Å	20.8984(5)	17.3670(3)	17.783(3)	28.2480(6)
c/Å	14.7749(3)	27.1155(3)	37.948(6)	9.1807(3)
α/°	90	93.7977(12)	90	90
β/°	90.942(2)	99.9260(13)	90	90
γ/°	90	99.1221(16)	90	90
V/Å^3	3606.06(15)	6175.69(19)	13971(4)	7325.7(3)
Z	4	2	8	8
Measured Refl's.	48351	83400	158073	21076
Indep't Refl's	12339	22444	12763	7211
Parameters	910	1524	883	767
Restraints	0	75	346	0.0360
GooF	1.0736	1.027	1.064	1.0578
wR_2 (all data)	0.0531	0.1973	0.1313	0.1525
wR_2	0.0489	0.1792	0.1248	0.1456
R_1 (all data)	0.0533	0.1051	0.0678	0.0645
R_1	0.0357	0.0730	0.0564	0.0552

$R_1 = \Sigma|F_o| - |F_c|/\Sigma|F_o|$, $wR_2 = [\Sigma(F_o^2 - F_c^2)^2/\Sigma w(F_o^2)^2]^{1/2}$.

*Table S4: Selected bond lengths (Å) for compound $LTiMe_2$ **1a**, $[LTiMe]^+[MeB(C_6F_5)_3]^-$ **2a** and of $[LTiBn]^+[B(C_6F_5)_4]^-$ **2b**.*

Bond	1a	2a	2b
Ti—C1	2.1119(8)	2.032(3)/ 2.081(4)	2.136(3)
Ti—C2	2.1087(9)	-	-
Ti—O1	1.8674(5)	1.788(3)/1.786(3)	1.7992(18)
Ti—O2	1.8649(5)	1.791(3)/1.778(3)	1.7879(18)
Ti—N1	2.3731(6)	2.247(3)/2.282(3)	2.242(2)
Ti—N2	2.5222(7)	2.232(3)/2.224(3)	2.296(2)

*Table S5: Selected bond angles (°) for compound $LTiMe_2$ **1a**, $[LTiMe]^+[MeB(C_6F_5)_3]^-$ **2a** and of $[LTiBn]^+[B(C_6F_5)_4]^-$ **2b**.*

	1a	2a	2b
O1-Ti-O2	163.35(2)	115.78(12)	119.56(8)
N1-Ti-O2	80.54(2)	88.41(12)	86.38(8)
N1-Ti-O1	82.85(2)	85.50(11)	85.08(8)
N2-Ti-O2	91.26(2)	111.16(13)	106.46(8)
N2-Ti-O1	85.11(2)	128.81(13)	128.77(8)
N2-Ti-N1	74.06(2)	76.37(12)	76.16(8)
C1-Ti-O2	96.13(3)	104.09(15)	107.38(11)
C1-Ti-O1	99.71(3)	99.52(15)	102.03(9)
C1-Ti-N1	158.55(3)	162.55(15)	157.91(10)
C1-Ti-N2	84.90(3)	87.65(15)	83.26(9)
C2-Ti-O2	91.58(3)	-	-
C2-Ti-O1	99.76(3)	-	-
C2-Ti-N1	105.99(3)	-	-
C2-Ti-N2	176.66(3)	-	-
C1-Ti-C2	95.26(4)	-	-

*Table S6: Selected bond lengths (Å) and angles (°) for $LAlOCH_2C{\equiv}CH$ **3**.*

Bond	Length (in Å)	X-Al-Y	Angle (°)
Al—O1	1.763(2)	O2-Al-O1	120.79(11)
Al—O2	1.774(2)	O3-Al-O1	96.28(12)
Al—O3	1.777(3)	O3-Al-O2	97.03(14)
Al—N1	2.182(3)	N1-Al1-O1	90.00(10)
Al—N2	2.089(3)	N1-Al1-O2	89.26(10)
-	-	N1-Al1-O3	167.18(16)
-	-	N2-Al1-O1	124.08(12)
-	-	N2-Al1-O2	114.03(11)
-	-	N2-Al1-O3	87.06(17)
-	-	N2-Al1-N1	80.18(12)

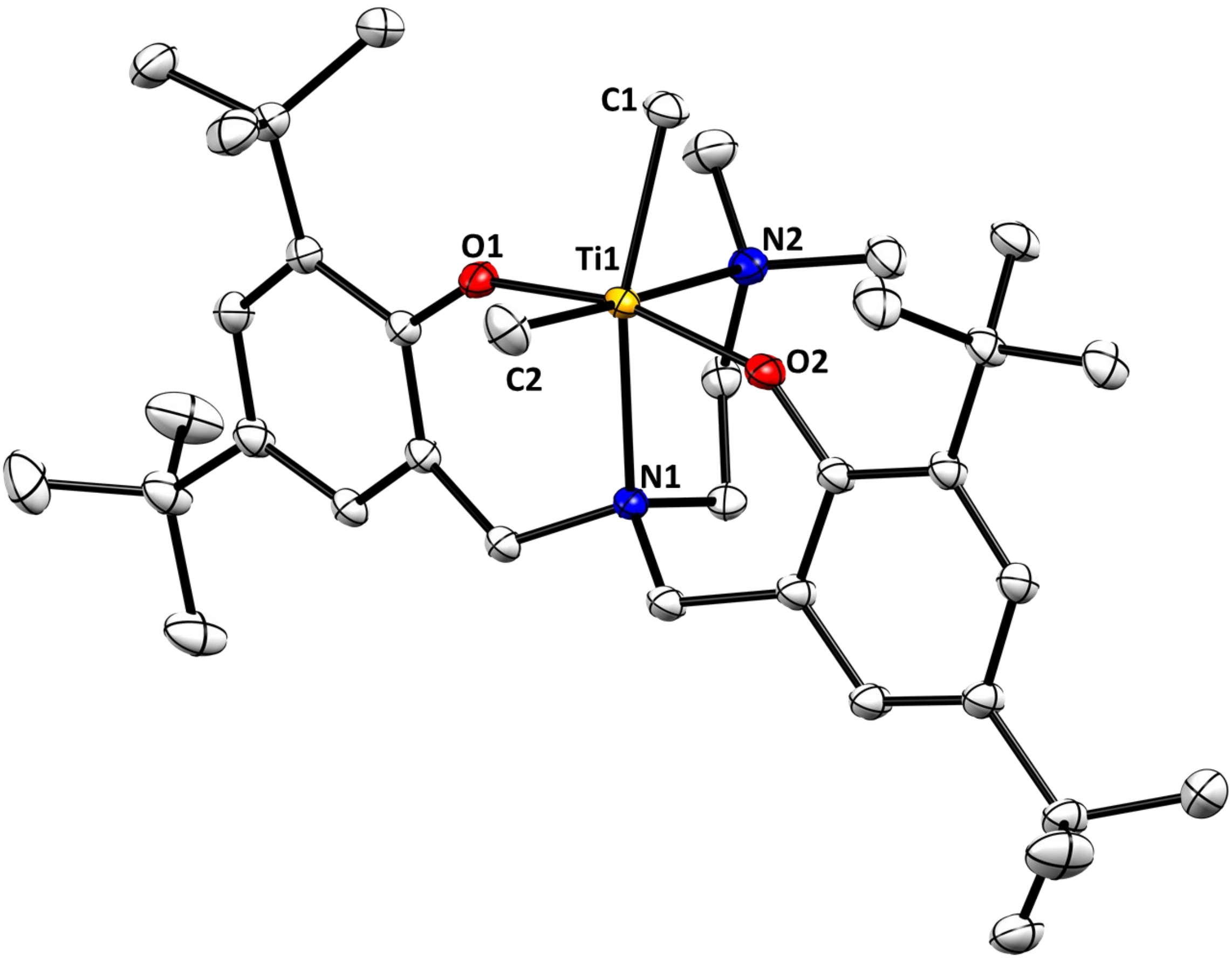

*Figure S84: Molecular structure of $LTiMe_2$ **1a**. Hydrogen atoms are omitted for clarity; thermal ellipsoids shown at 50%.*

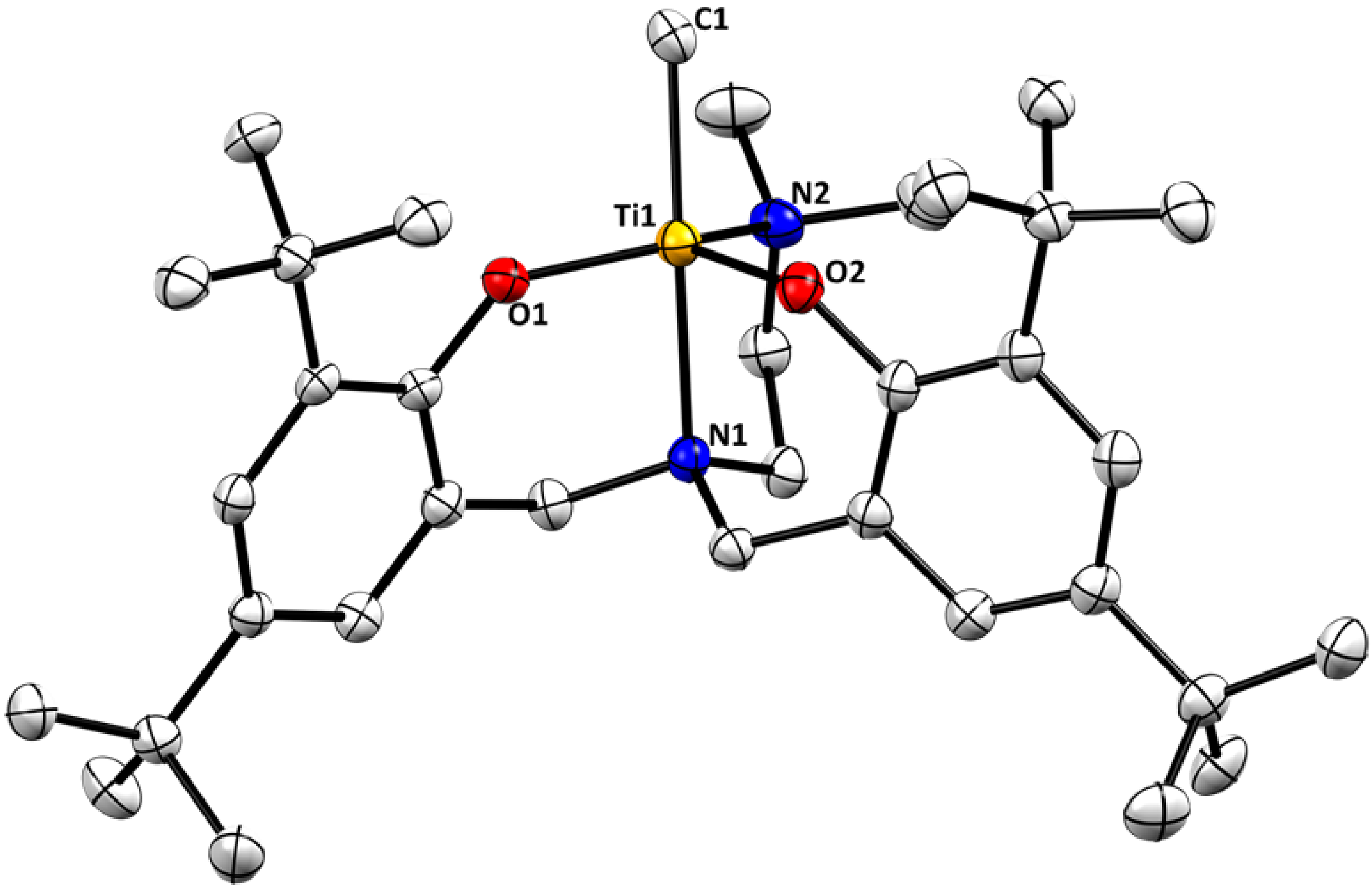

*Figure S85: Molecular structure of $[LTiMe]^+[MeB(C_6F_5)_3]^-$ **2a**. Hydrogen atoms, solvent and counter ion are omitted for clarity; thermal ellipsoids shown at 50%.*

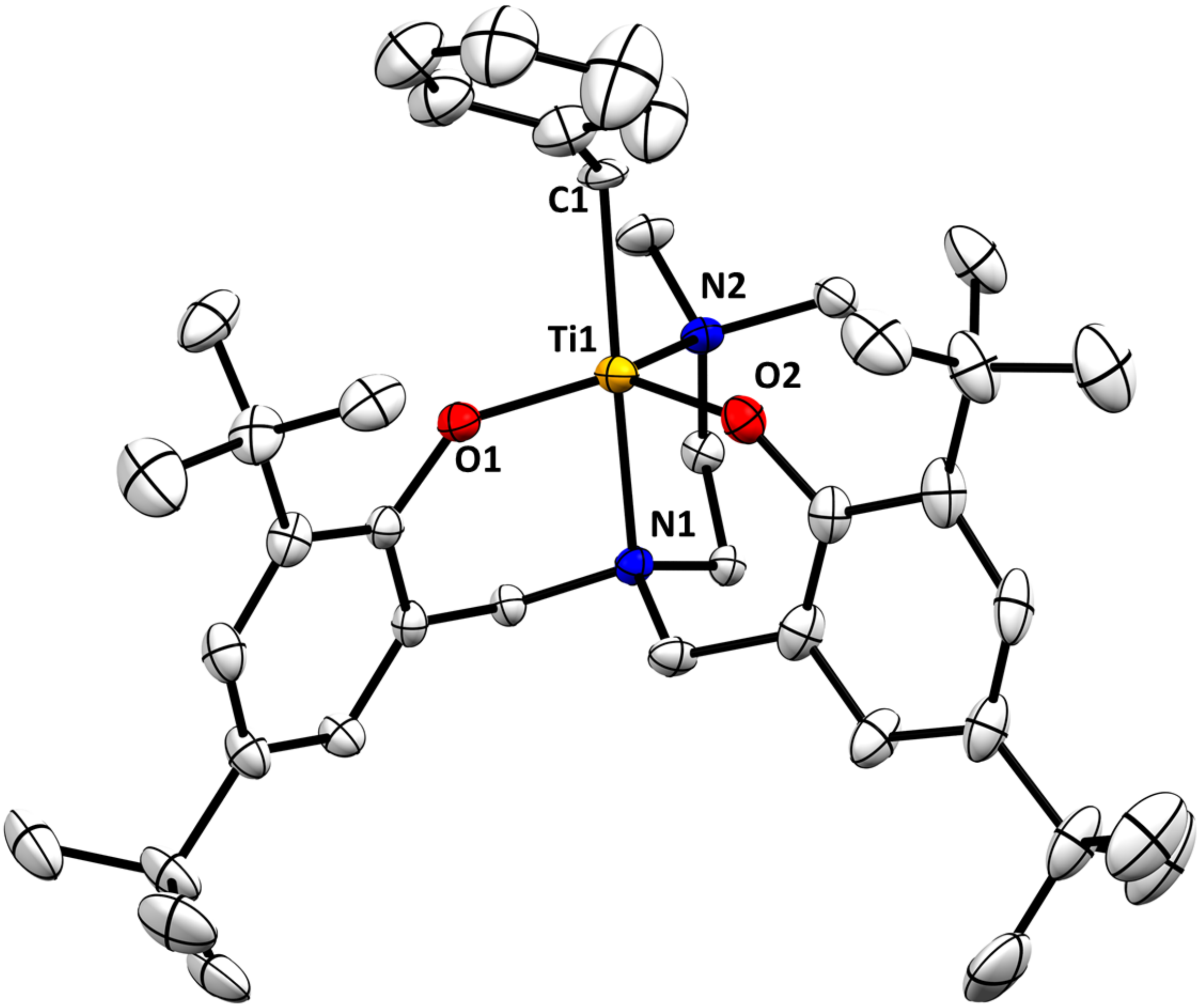

*Figure S86: Molecular structure of the cationic part of [LTiBn]$^{+}$[B(C_6F_5)$_4$]$^{-}$ **2b**. Hydrogen atoms are omitted for clarity.*

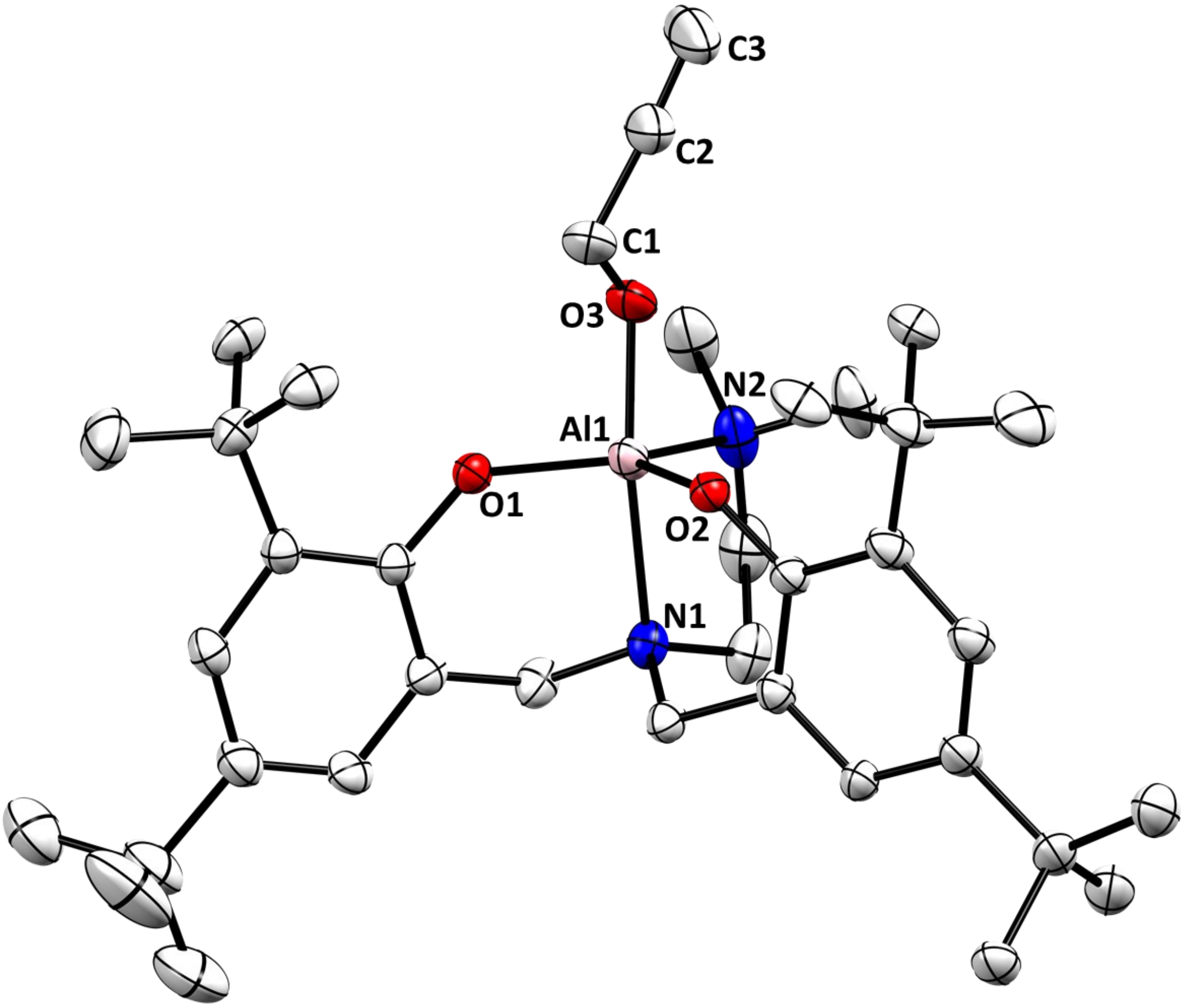

*Figure S87: Molecular structure of LAlOCH$_2$C≡CH **3.** Thermal ellipsoids shown at 30% probability. Hydrogen atoms are omitted for clarity.*

References

[1] E. Y. Tshuva, I. Goldberg, M. Kol, Z. Goldschmidt, "Coordination Chemistry Of Amine Bis(Phenolate) Titanium Complexes: Tuning Complex Type and Structure by Ligand Modification" *Inorg. Chem.* **2001**, *40*, 4263–4270, https://doi.org/10.1021/ic010210s.

[2] S. Barroso, A. M. Coelho, S. Gómez-Ruiz, M. J. Calhorda, Ž. Žižak, G. N. Kaluerović, A. M. Martins, "Synthesis, Cytotoxic and Hydrolytic Studies of Titanium Complexes Anchored by a Tripodal Diamine Bis(Phenolate) Ligand" *Dalton Trans.* **2014**, *43*, 17422–17433, https://doi.org/10.1039/C4DT00975D.

[3] E. Y. Tshuva, I. Goldberg, M. Kol, Z. Goldschmidt, "Living Polymerization of 1-Hexene due to an Extra Donor Arm on a Novel Amine Bis(Phenolate) Titanium Catalyst" *Inorg. Chem. Commun.* **2000**, *3*, 611–614, https://doi.org/10.1016/S1387-7003(00)00151-9.

[4] C. T. Chen, C. A. Huang, B. H. Huang, "Aluminium Metal Complexes Supported by Amine Bis-Phenolate Ligands as Catalysts for Ring-Opening Polymerization of ε-Caprolactone" *Dalton Trans.* **2003**, *3*, 3799–3803, https://doi.org/10.1039/B307365C.

[5] D. H. Wu, A. Chen, C. S. Johnson, "An Improved Diffusion-Ordered Spectroscopy Experiment Incorporating Bipolar-Gradient Pulses" *J. Magn. Reson. A* **1995**, *115*, 260–264, https://doi.org/10.1006/jmra.1995.1176.

[6] A. D. Horton, "Unprecedented Isolation of Alk-1-En-3-Yn-1-Yl Intermediates in the Catalytic Oligomerisation of Alk-1-Ynes by Cationic Lewis Base-Free Zirconocene Complexes" *J. Chem. Soc. Chem. Commun.* **1992**, 185–187, https://doi.org/10.1039/C39920000185.

[7] G. M. Sheldrick, "A Short History of *SHELX*" *Acta Crystallogr. A* **2008**, *64*, 112–122, https://doi.org/10.1107/S0108767307043930.

[8] G. M. Sheldrick, "*SHELXT* – Integrated Space-group and Crystal-structure Determination" *Acta Crystallogr. A Found. Adv.* **2015**, *71*, 3–8, https://doi.org/10.1107/S2053273314026370.

[9] G. M. Sheldrick, "Crystal Structure Refinement with *SHELXL*" *Acta Crystallogr. C Struct. Chem.* **2015**, *71*, 3–8, https://doi.org/10.1107/S2053229614024218.

[10] L. J. Bourhis, O. V. Dolomanov, R. J. Gildea, J. A. K. Howard, H. Puschmann, "The Anatomy of a Comprehensive Constrained, Restrained Refinement Program for the Modern Computing Environment – *Olex2* Dissected" *Acta Crystallogr. A Found. Adv.* **2015**, *71*, 59–75, doi:10.1107/S2053273314022207.

[11] O. V. Dolomanov, L. J. Bourhis, R. J. Gildea, J. A. K. Howard, H. Puschmann, "*OLEX2* : A Complete Structure Solution, Refinement and Analysis Program" *J. Appl. Crystallogr.* **2009**, *42*, 339–341, https://doi.org/10.1107/S0021889808042726.
